# Functionalized spirolactones by photoinduced dearomatization of biaryl compounds†
†Electronic supplementary information (ESI) available. See DOI: 10.1039/c8sc05476b


**DOI:** 10.1039/c8sc05476b

**Published:** 2019-02-19

**Authors:** Hongji Li, Elena Subbotina, Anon Bunrit, Feng Wang, Joseph S. M. Samec

**Affiliations:** a State Key Laboratory of Catalysis (SKLC) , Dalian National Laboratory for Clean Energy (DNL) , Dalian Institute of Chemical Physics (DICP) , Dalian 116023 , China . Email: wangfeng@dicp.ac.cn; b University of Chinese Academy of Sciences , Beijing 100049 , China; c Department of Organic Chemistry , Stockholm University , SE-106 91 , Stockholm , Sweden . Email: joseph.samec@su.se

## Abstract

The idea of using biaryl structures to generate synthetic building blocks such as spirolactones is attractive because biaryl structures are abundant in biomass waste streams. However, the inertness of aromatic rings of biaryls makes it challenging to transform them into functionalized structures. In this work, we developed photoinduced dearomatization of nonphenolic biaryl compounds to generate spirolactones. We demonstrate that dearomatization can be performed *via* either aerobic photocatalysis or anaerobic photooxidation to tolerate specific synthetic conditions. In both pathways, dearomatization is induced by electrophilic attack of the carboxyl radical. The resulting spirodiene radical is captured by either oxygen or water in aerobic and anaerobic systems, respectively, to generate the spirodienone. These methods represent novel routes to synthesize spirolactones from the biaryl motif.

## Abstract 2

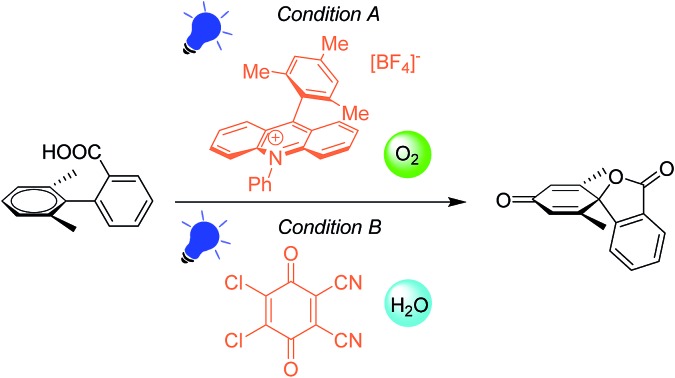
Visible-light-induced dearomatization of non-phenolic 1,1′-biaryl-2-carboxylic acids has been developed by either aerobic photocatalytic or anaerobic photooxidative pathways.

## Introduction

**Fig. 1:**
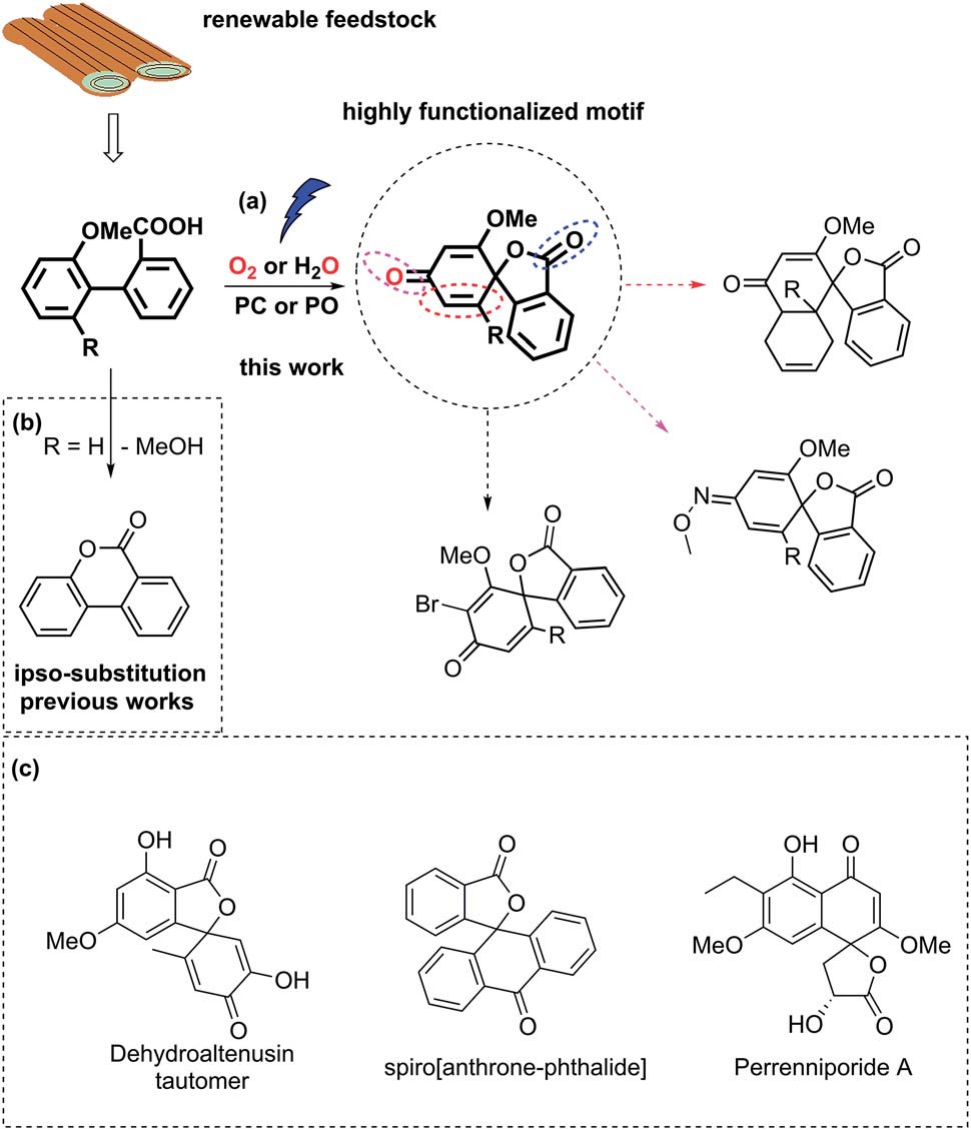
(a) Carboxyl radical-induced dearomative spirolactonization of biaryl carboxylic acids. PC denotes a photocatalyst, and PO denotes a photooxidant. Potential transformations of the highly functionalized motif. (b) Previous reports regarding transformations of biaryl carboxylic acids, where *ipso*-substitution of the methoxy group was observed. (c) Examples of relevant compounds containing a spirolactone or dearomatized biaryl functionality.^14^–^16^

Biaryl compounds are abundant in biomass waste streams from pulping of lignocellulose.^1^–^4^ In the lignin structure, the biphenyl linkage is known as the 5–5′ bond which is inert and preserved during the pulping of biomass (Fig. 1). Thus, this motif is generated in high amounts as a by-product in bio-refineries and can therefore be considered as a potential future green feedstock.^4^–^11^ The biaryl motifs are also found as intermediates and products in, for example, the pharmaceutical industry and thus have synthetic relevance.^12^,^13^ A potential application of this synthon is dearomative spirolactonization, where one of the aryls is dearomatized by an *o*-carboxylic acid to produce a spirolactone. The spirolactone structure is found for example in the dehydroaltenusin tautomer isolated from mycelium extracts, which could act as selective DNA polymerase α inhibitor.^14^ Spirolactones comprising the anthracene moiety are reported in the patent literature as substrates for recording materials.^15^ Such a spirolactone possess many functional groups that can each be further converted, which makes it potentially a highly valuable intermediate (Fig. 1, ESI†).^16^

Dearomative spirolactonization has previously been realized with phenolic compounds *via* a facile phenol oxidation reaction followed by nucleophilic attack.^17^–^23^ Regarding the dearomatization of nonphenolic biaryls, no examples of a carboxyl-radical-induced transformation have been reported. A few protocols in which nitrogen-based radical or nitrenium ion dearomatization led to spiro-formation have been developed.^24^–^27^ Those transformations were driven by the low activation entropy of the N-centered radicals for the cyclization to 5-membered products.^28^ In comparison, the carboxyl motif is readily available, however, strong preferences for carboxyl radicals to form 6-membered products have been reported by Gonzales and co-workers, who found that the blockage of the *ortho*-position of the aryl ring by a methoxy group led to *ipso* substitution instead of spirolactonization.^29^

Herein, we report the first photocatalyzed dearomatization of nonphenolic biaryls mediated by a carboxyl radical. The reaction can be performed on substrates blocked in the *ortho*-position without *ipso* substitution using an acridinium catalyst under aerobic conditions. Taking into account the feasibility of generating such biaryls from lignin, this is a sustainable methodology to produce highly functionalized motifs. Importantly, due to the suppressed *ipso* substitution, spirolactones with labile groups (OMe) can be generated (Fig. 1). In addition, a complementary methodology in which commercial 2,3-dichloro-5,6-dicyano-1,4-benzoquinone (DDQ) is used as a photooxidant to generate the spirolactones under aqueous conditions is disclosed to tolerate anaerobic synthetic conditions.

## Results and discussion

### Condition screening for the dearomative spirolactonization of a biaryl acid

Initially model compound **1**, in which the *ortho*-position of attacked arene was methyl substituted, was chosen for the optimization of reaction conditions (Table 1). Acridinium catalyst i was chosen as the photocatalyst for oxidation of the carboxylic group in view of its excellent performance in many organic oxidation reactions.^30^–^33^ Screening of the catalyst and solvents showed that product **2** could be generated in 19% yield in the presence of 20 mol% of catalyst i under aerobic conditions (Table 1, entry 2). To facilitate the formation of the carboxyl radical *via* the deprotonation of the carboxyl group, several amines as well as inorganic bases were tested (see Table S1†). The addition of 1 equivalent of 1,4-diazabicyclo[2.2.2]octane (DABCO) resulted in the formation of the product in 65% yield. When the catalyst loading was decreased to 5 mol%, a significant decrease in the yield was observed (Table 1, entry 4). Several additives, such as 2,2,6,6-tetramethylpiperidine-1-oxyl (TEMPO) and DDQ, were tested with low catalyst loading. The use of both TEMPO and DABCO together allowed us to obtain the desired product in 89% yield after only 4 h (68% isolated yield, Table 1, entry 6), which could be ascribed to the scavenging of the reactive oxygen species by TEMPO, which suppresses overoxidation of the substrate.^34^ We name this set of aerobic conditions condition A.

**Table 1:** Optimization of reaction conditions ^*a*^

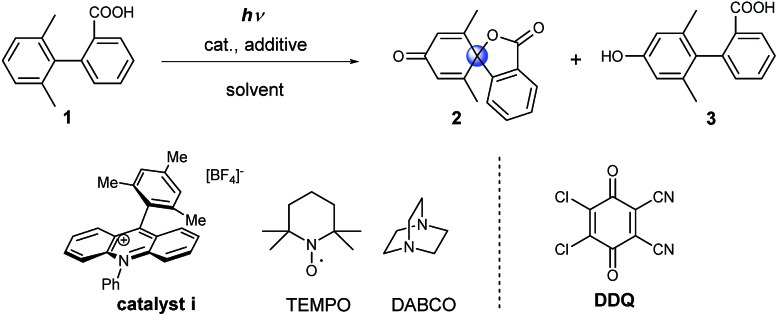					
Entry	Cat. (equiv.)	Additive (equiv.)	Solvent	Yield, **2**, %	Yield, **3**, %
1	**i** (0.2)	None	CH_3_CN	5	Trace
2	**i** (0.2)	None	Acetone	19	Trace
3	**i** (0.2)	DABCO (1)	Acetone	65	Trace
4	**i** (0.05)	DABCO (1)	Acetone	15	Trace
5	**i** (0.05)	TEMPO (1)	Acetone	37	Trace
6^*b*^	**i** (0.08)	DABCO (1), TEMPO (1)	Acetone	**89 (68)**	Trace
7	**i** (0.08)	DDQ (1)	Acetone	Trace	48
8^*c*^	None	DDQ (1)	CH_3_CN	Trace	33
9^*d*^	None	DDQ (1)	CH_3_CN	Trace	63
10^*c*^	None	DDQ (2)	CH_3_CN	44	21
11^*c*^	None	DDQ (4)	CH_3_CN	72	16
12^*c*^	None	DDQ (6)	CH_3_CN	**93**	6

^*a*^Reactions conditions: 0.05 mmol scale, solvent (1 mL) under LED lamps (427 nm) for 12 h, air, at room temperature. NMR yields *vs.* 1,3,5-trimethoxybenzene as the internal standard.

^*b*^Reaction time: 4 h, (isolated yield).

^*c*^LED lamps (440 nm) for 20 min, Ar atmosphere, 22 equiv. H_2_O.

^*d*^Reaction time: 15 h.

DDQ may participate in hydrogen atom abstraction or serve as a terminal oxidant and thus facilitate the regeneration of the catalyst.^35^–^38^ When 1 equivalent of DDQ was used as the only additive, we observed the formation of phenolic compound **3** (Table 1, entry 7). Fukuzumi and co-workers reported that the triplet excited state of DDQ could oxidize benzene to phenol using water as the oxygen source.^35^ Thus, we carried out the reaction using DDQ without an acridinium catalyst but with the addition of water. Under these reaction conditions, phenol **3** was obtained as the main product after 15 h (Table 1, entry 9). Interestingly, when the amount of DDQ was increased from 2 to 6 equivalents (Table 1, entries 10–12), the spiro product **2** was obtained in excellent yield after only 20 minutes under inert reaction conditions (Table 1, entry 12). We name this set of anaerobic conditions condition B. Attempts to use DDQ in catalytic amounts in the presence of co-catalysts such as nitrates and *tert*-butyl nitrite failed (see ESI Table 2†).^39^

### Substrate scope

**Fig. 2:**
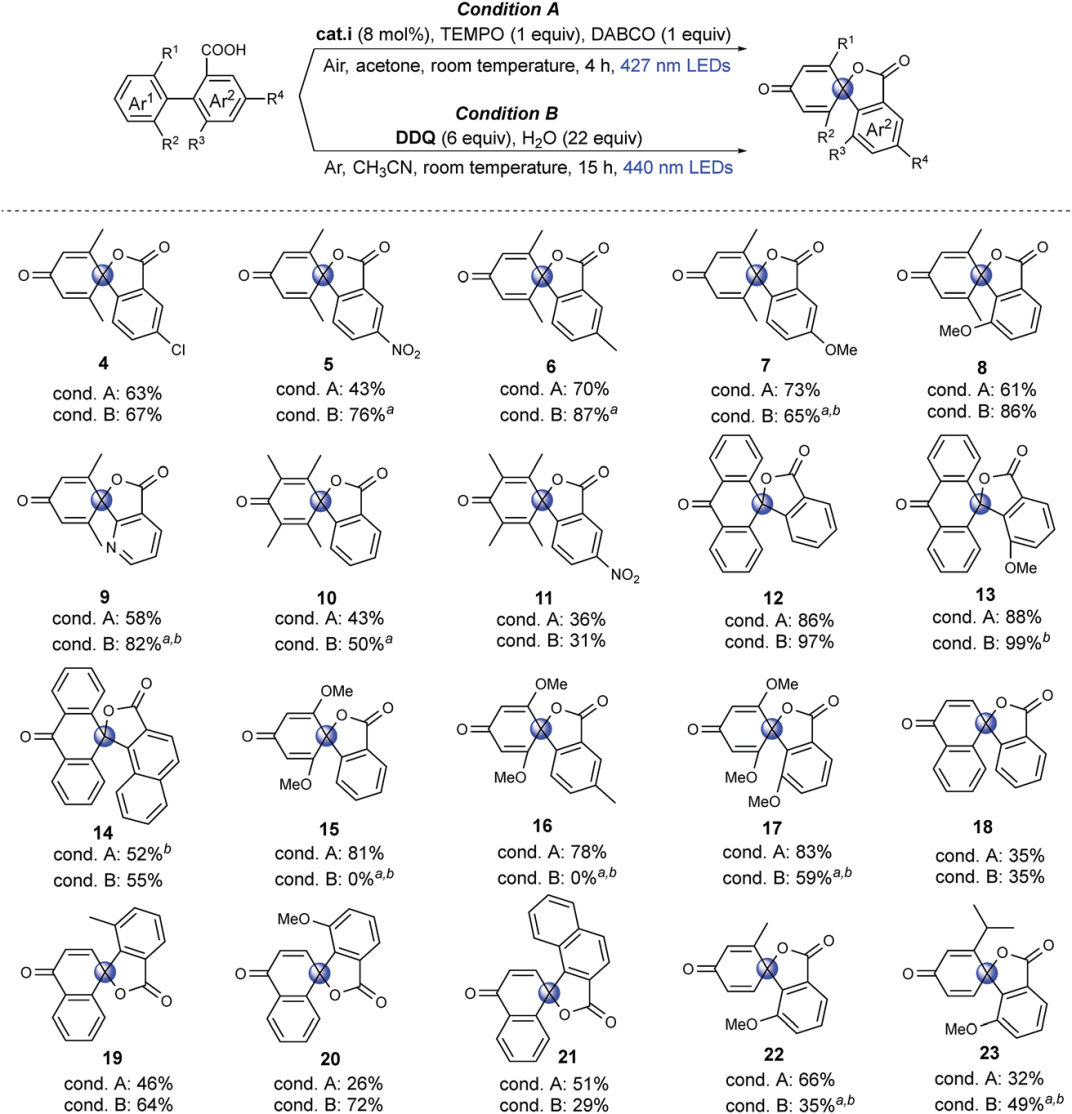
Substrate scope of dearomative spirolactonization under aerobic and anaerobic conditions. Substrate: 0.1 mmol, isolated yields. ^*a*^Reaction time: 0.5 h. ^*b*^Substrate: 0.05 mmol, NMR yields *vs.* 1,3,5-trimethoxybenzene internal standard, the blue circles were used to label the spiro carbon.

To investigate the feasibility of our two methods and reveal the differences between the aerobic and anaerobic systems, a range of different 1,1′-biaryl-2-carboxylic acids were tested using the two reaction conditions (Fig. 2). Initially, *meta*-xylene substrates on the Ar^1^ ring were evaluated. It is worth mentioning that, under aerobic conditions, oxidation of the dimethyl-substituted aromatic rings can occur, leading to decomposition.^37^,^40^,^41^ Yet, when using our optimized reaction conditions, no over-oxidation was observed and, remarkably, the products were even obtained in good yields. Introduction of a nitro group in the *meta*-position to the carboxyl group (Ar^2^) resulted in a lower yield of product **5**, in which the higher oxidation potential of aryl carboxylic acid may limit the carboxylic radical formation.^36^ Under anaerobic conditions (condition B), good to excellent yields of products **4–8** were achieved. When the phenyl group was exchanged for a pyridine moiety (Ar^2^), moderate to good yields of product **9** were obtained. Tetramethyl arenes are extremely challenging substrates because they are highly activated for oxidation.^37^,^41^ To our delight, moderate yields of **10** and **11** could be obtained using both methodologies. With an anthracene substituent (Ar^1^), good to excellent yields of spiro-products were obtained using both methodologies (**12**, **13**). However, when Ar^2^ was exchanged for a naphthyl group, a moderate yield of the product (**14**) was obtained. Dimethoxy-substituted arenes (Ar^1^) are another type of challenging substrates, as *ipso* substitution of the methoxy group can occur.^28^ In this case, moderate to excellent yields of spirolactones were obtained using condition A (**15–17**). Under condition B, spiro products **15** and **16** were not formed, and instead, *ipso* substitution of the methoxy group occurred, affording the six-membered lactones. Interestingly, when the *o*-MeO group was introduced onto the Ar^2^ ring to further promote the twisted conformation, *ipso* substitution was suppressed, and product **17** was formed in a moderate yield under condition B. Then, challenging naphthyl (Ar^1^) substrates with exposed *o*-H were tested. The exposed *o*-H could give the lactone as a six-membered product. Under condition A, low to moderate yields of products **18–21** were obtained, which may result from the formation of the endoperoxide structure under an oxygen atmosphere, as previously reported.^42^ Using condition B resulted in moderate to good yields of the desired products (**19**, **20**). Finally, very challenging aryls that were mono-substituted on both the Ar^1^ and Ar^2^ rings were tested. Remarkably, products **22** and **23** were obtained in moderate to good yields. In particular, compound **23** is noteworthy, as the isopropyl group is prone to undergo benzylic oxidation or *ipso* substitution. In summary, we found both methods to be feasible; moreover, the two systems are complementary to meet required synthetic conditions, aerobic or anaerobic conditions, dry or aqueous conditions, in that most of the substrates tested can be transformed into the spirolactones in moderate to high yields using at least one of the two different systems.

### Mechanistic studies: control experiments

To gain insight into the mechanism of the reaction, several control experiments were conducted.

### Reactions in the dark and under an inert atmosphere

Performing the reactions under dark conditions led to no conversion in either system, indicating that both processes are photoinduced (see ESI Table 1†). Furthermore, no product formation occurred under an argon atmosphere for condition A.

### Labelling experiments to determine the origin of the dienone oxygen

**Fig. 3:**
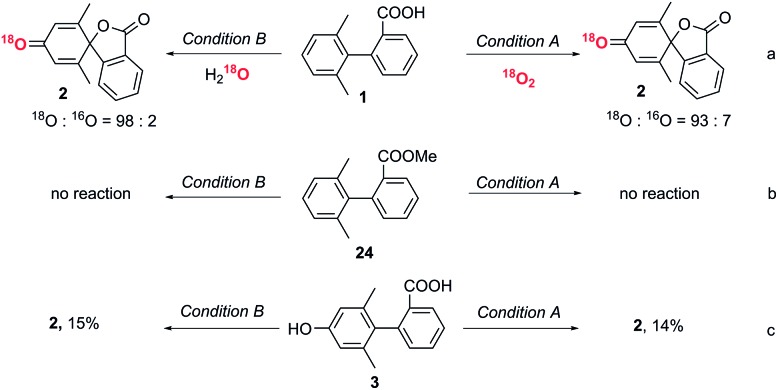
Mechanistic studies to reveal the oxygen source, the roles of the carboxyl group and phenolic compounds during the dearomative spirolactonization. NMR yields *vs.* 1,3,5-trimethoxybenzene internal standard.

An isotopic labelling experiment using ^18^O_2_ showed that the oxygen in the dienone originated from oxygen in the air in condition A (Fig. 3a). An isotopic labelling experiment using H_2_^18^O showed that the oxygen in the dienone originated from water in condition B. These labelling experiments demonstrate that there are two distinct reaction mechanisms in the spirolactone formation.

### The role of the carboxyl group

To exclude direct oxidation of the aromatic ring (Ar^1^) instead of the carboxyl group, electrochemical measurements were performed (see ESI, Fig. S1†). The oxidation potential of the carboxyl group is lower than that of Ar^1^ showing oxidation of the carboxyl group to generate a carboxylic radical is easier than oxidation of the aromatic ring. When the methyl ester derivative of the biphenyl carboxylic acid (**24**) was subjected to reaction conditions A and B, the starting material was recovered (Fig. 3b), which shows the essential role of carboxyl to trigger this dearomatization. The generation of an aryl carboxyl radical could be further confirmed by the conversion of a biphenyl acid to a six-membered lactone product under both conditions (control experiment, ESI, page S13†).^29^

### The role of phenolic compound **3**

Since phenolic compound **3** was formed under condition B when only one equivalent of DDQ was used, we wanted to determine if this was an intermediate in the formation of **2** or a by-product. To this end, phenol substrate **3** was subjected to both sets of optimized reaction conditions. Interestingly, only a small amount of the desired product, **2**, was observed in each case, implying that phenol **3** is not an intermediate to the conversion to the final spiro product (Fig. 3c).

### Proposed mechanism

**Fig. 4:**
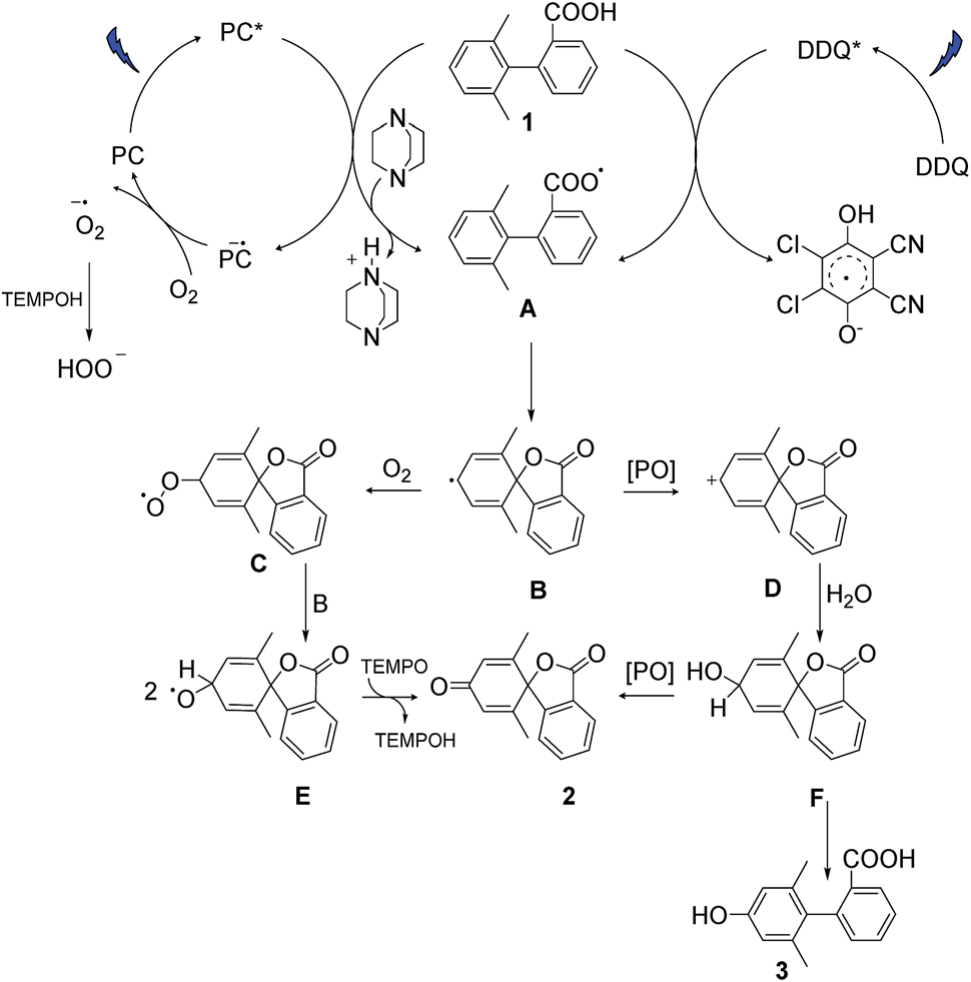
Proposed reaction pathways. PC and PO are the abbreviation of the photocatalyst and photooxidant.

Based on our experimental data and previous literature reports, we propose the following reaction pathways under aerobic and anaerobic conditions (Fig. 4). The formation of key intermediate **A**, the carboxyl radical, occurs as a first step in both reaction mechanisms.^29^ Electrochemical measurements, the reaction using ester analogue **24** (Fig. 3b), and the formation of the lactone from non-substituted biaryls (control experiments, ESI, page S13†) support this proposal. Carboxyl radical intermediate **A** induces dearomatization by intramolecular cyclization to form intermediate **B** in both systems, where the twisted conformation promotes the cyclization.^28^ From this point, the two pathways diverge. Under aerobic condition A, nucleophilic attack of intermediate **B** by oxygen could deliver peroxy radical **C**, and then reaction between **C** and **B** gives radical **E**,^43^,^44^ which could further deliver product **2***via* hydrogen atom transfer in the presence of TEMPO.^34^ This pathway was confirmed by using labelled oxygen (^18^O_2_) (Fig. 3a) and by the lack of any reaction under argon. The addition of TEMPO could quench the harmful superoxide radical anion and realize mild oxidative conditions.^45^ Meanwhile, the DABCO additive has been proposed to facilitate carboxyl radical formation by deprotonation.^46^ In the case of condition B, intermediate **B** undergoes oxidation to give cation **D**, which then traps a molecule of water to deliver hydroxylated intermediate **F**. This pathway was confirmed by using labelled water (H_2_^18^O) (Fig. 3a) and by the low conversion that occurred under dry conditions with oxygen (entry 17, Table S1†). Intermediate **F** can then either undergo further oxidation to give final product **2** or be rearomatized to furnish by-product **3**. When higher concentrations of DDQ were used, the oxidation rate increased, and the side reaction was suppressed. When lower concentrations of DDQ were used, rearomatization of intermediate **F** occurred and phenol **3** was generated.^36^ This pathway is supported by the reaction of the methyl ester (Fig. 3b), which shows that very low conversion to phenol was observed in the absence of a carboxyl radical, in contrast to the reaction of the carboxylic acid generating the phenol product in 63% yield (Table 1, entry 9).

## Conclusions

In conclusion, our carboxyl radical-induced dearomatization of non-phenolic arenes provides a sustainable methodology for generating highly functionalized spirolactones from lignin-derived biaryl compounds. These methods show the possibility to utilize the inert 5–5′ linkage in lignin to generate useful intermediates. The reaction can be performed *via* either aerobic photocatalytic or anaerobic photooxidative pathways. Both methods formed the carboxyl radical. This radical then attacks the neighboring aryl in the *ipso* position to generate a spirodiene radical, which is captured by reactive oxygen species or water in aerobic and anaerobic systems, respectively, to produce spirolactone products. Through this strategy, a number of spirolactones can be directly synthesized using nonphenolic arenes as starting materials. Especially, due to the suppressed *ipso* substitution, spirolactones with labile groups (OMe) can be generated, and such a spirolactone possess many functional groups that can each be further transformed, which makes it potentially a highly valuable intermediate. Thus, the developed methods can be applied to the syntheses of complex molecules where either inert or dry reaction conditions are required.

## Conflicts of interest

There are no conflicts to declare.

## Supplementary Material

Supplementary information
