# Supplementary material for: Functionalized spirolactones by photoinduced dearomatization of biaryl compounds
Source: Chem Sci. 2019 Feb 19;10(12):3681–6. doi: 10.1039/c8sc05476b (PMC6438148; doi:10.1039/c8sc05476b)
Supplement: Supplementary file 1 [file SC-010-C8SC05476B-s001.pdf]

Electronic Supplementary Material (ESI) for Chemical Science.
This journal is © The Royal Society of Chemistry 2019

Supplementary Materials for

Functionalized spirolactones by photoinduced dearomatization of biaryl compounds

Hongji Li,[ab†] Elena Subbotina,[c†] Anon Bunrit,[a] Feng Wang,*[a] and Joseph S. M. Samec*[c]

[a] State Key Laboratory of Catalysis (SKLC), Dalian National Laboratory for Clean Energy (DNL), Dalian Institute of Chemical Physics (DICP), Dalian 116023, China. [b] University of Chinese Academy of Sciences, Beijing 100049, China. [c] Department of Organic Chemistry, Stockholm University, SE-106 91, Stockholm, Sweden.† These authors contributed equally to this work.

Email: wangfeng@dicp.ac.cn

Email: joseph.samec@su.se

Table of Contents:

1	Checklist of characterization data of all compounds	S3
2	General	S8
3	All starting acids and spiro products	S8
4	The synthesis of 4'-hydroxy-2',6'-dimethyl-[1,1'-biphenyl]-2-carboxylic acid (**3**)	S9
5	The synthesis of Methyl 2',6'-dimethyl-[1,1'-biphenyl]-2-carboxylate (**24**)	S9
6	General procedure conditions **A** and **B**	S10
7	Optimization of reaction condition	S11
8	Attempts to use DDQ as a catalyst	S12
9	Electrochemical measurements	S13
10	Control experiments	S14
11	Isotope labelling expetiments	S15
12	Further transformations of spirolactone	S16
13	General method A to synthesize starting biaryl acid **1**	S16
14	General method B to synthesize starting biaryl acid **A13**	S17
15	General method C to synthesize starting hindered biaryl acid **A6**	S17
16	Characterization data of all starting acids	S19
17	Characterization data of all products	S26
18	Spectra of all starting acids	S35
19	Spectra of all products	S56
20	References	S82

1. Checklist of characterization data of all compounds:

1.1 Checklist of characterization data of synthesized acids:

Code	Compound	Known/ Unknown	^{1}H-NMR	^{13}C-NMR	IR	HRMS
**1**	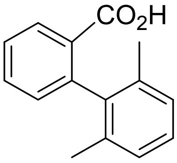	**Known**[1]	√	√	-	-
**A2**	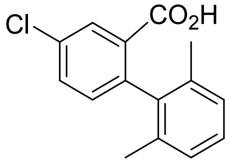	**Unknown**	√	√	-	-
**A3**	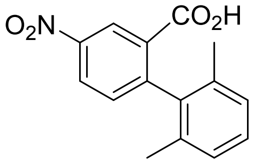	**Unknown**	√	√	-	-
**A4**	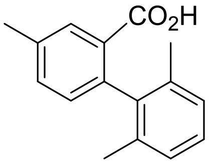	**Unknown**	√	√	-	-
**A5**	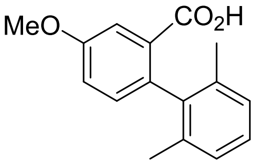	**Unknown**	√	√	-	-
**A6**	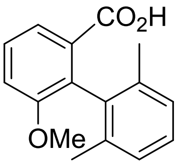	**Unknown**	√	√	-	-
**A7**	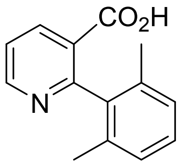	**Unknown**	√	√	-	-
**A8**	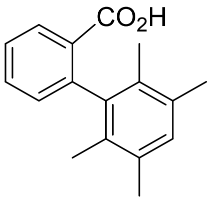	**Unknown**	√	√	-	-

**A9**	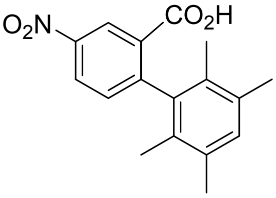	**Unknown**	√	√	-	-
**A10**	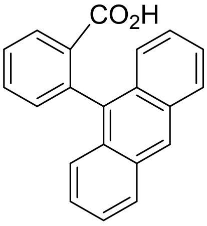	**Known**[2]	√	√	-	-
**A11**	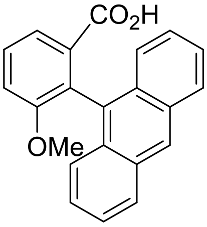	**Unknown**	√	√	-	-
**A12**	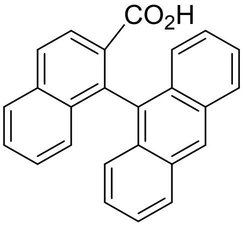	**Unknown**	√	√	-	-
**A13**	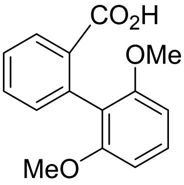	**Known**[3]	√	√	-	-
**A14**	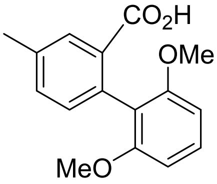	**Unknown**	√	√	-	-
**A15**	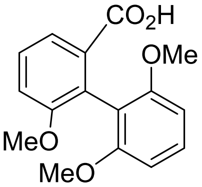	**Unknown**	√	√	-	-
**A16**	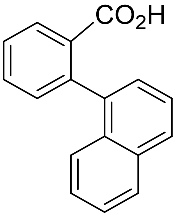	**Known**[4]	√	√	-	-

**A17**	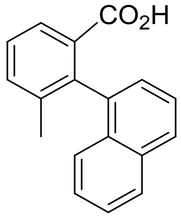	**Unknown**	√	√	-	-
**A18**	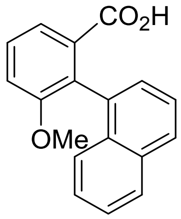	**Known**[5]	√	√	-	-
**A19**	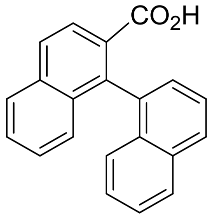	**Known**[6]	√	√	-	-
**A20**	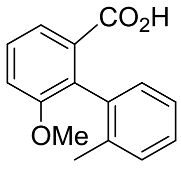	**Unknown**	√	√	-	-
**A21**	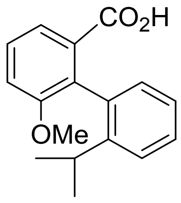	**Unknown**	√	√	-	-

1.2 Checklist of characterization data of all products:

**Code**	**Compound**	**Known/ Unknown**	**^{1}H-NMR**	**^{13}C-NMR**	**IR**	**HRMS**
**2**	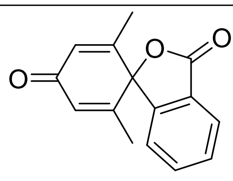	**Unknown**	√	√	√	√
**3**	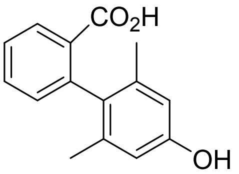	**Unknown**	√	√	√	√
**4**	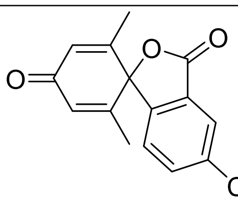	**Unknown**	√	√	√	√

**5**	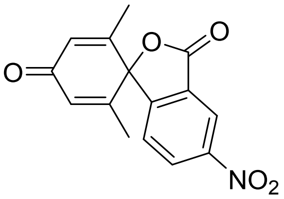	**Unknown**	√	√	√	√
**6**	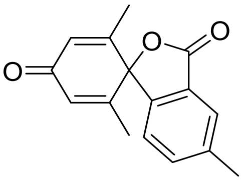	**Unknown**	√	√	√	√
**7**	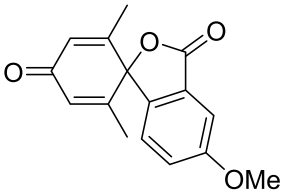	**Unknown**	√	√	√	√
**8**	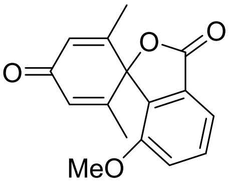	**Unknown**	√	√	√	√
**9**	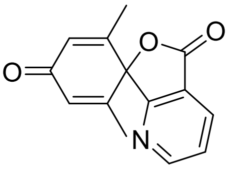	**Unknown**	√	√	-	√
**10**	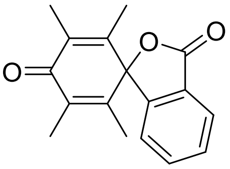	**Unknown**	√	√	√	√
**11**	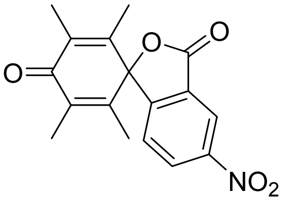	**Unknown**	√	√	√	√
**12**	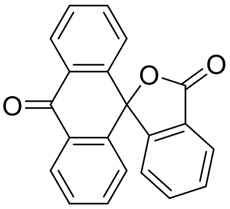	**Known**[7]	√	√	-	√
**13**	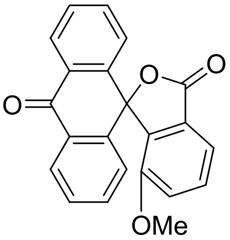	**Unknown**	√	√	√	√

**14**	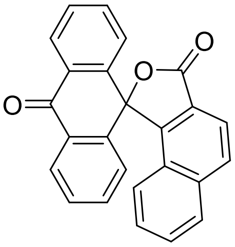	**Unknown**	√	√	√	√
**15**	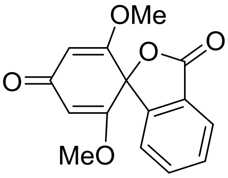	**Unknown**	√	√	√	√
**16**	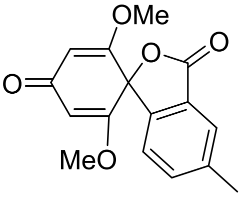	**Unknown**	√	√	√	√
**17**	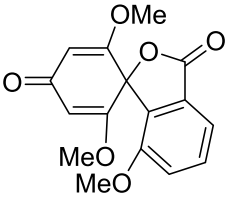	**Unknown**	√	√	-	√
**18**	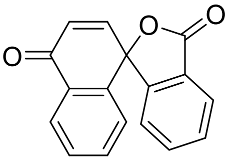	**Unknown**	√	√	√	√
**19**	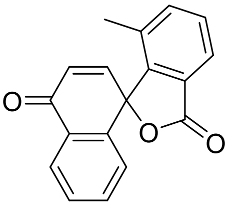	**Unknown**	√	√	√	√
**20**	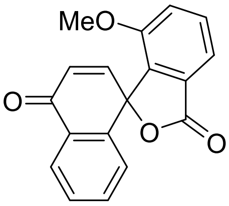	**Unknown**	√	√	√	√
**21**	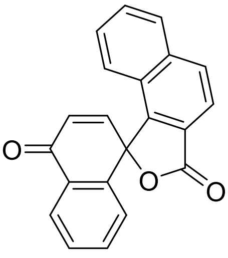	**Unknown**	√	√	√	√

**22**	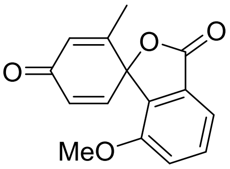	**Unknown**	√	√	√	√
**23**	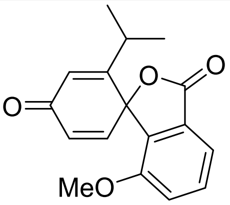	**Unknown**	√	√	-	√
**24**	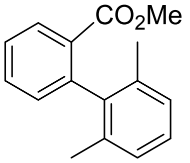	**Known**[8]	√	√	-	-

2. General

^{1}H-NMR spectra were recorded with a Bruker 400 (400 MHz) spectrometer as solutions in $CDCl_3$. Chemical shifts are expressed in parts per million (ppm, δ) and are referenced to $CHCl_3$ (δ = 7.26 ppm) as an internal standard. All coupling constants are absolute values and are expressed in Hz. The description of the signals include: s = singlet, d = doublet, t = triplet, m = multiplet and dd = doublet of doublets, at = apparent triplet. ^{13}C -NMR spectra were recorded with a Bruker 400 (101 MHz) spectrometer as solutions in $CDCl_3$ with complete proton decoupling. Chemical shifts are expressed in parts per million (ppm, δ) and are referenced to $CDCl_3$ (δ = 77.0 ppm) as an internal standard. IR spectra were recorded by a Perkin Elmer FT-IR Spectrometer. High-Resolution Mass Spectra (HRMS) were performed with a micrOTOF (Bruker) spectrometer. The molecular fragments are quoted as the relation between mass and charge (m/z). The routine monitoring of reactions was performed by crude ^{1}H NMR and/or with silica gel pre-coated Al plate, which was analyzed with iodine and/or uv light. Solvents, reagents and chemicals were purchased from Aldrich. All reactions were executed with oven-dried glassware. The LED lamps (PR160 LED Photoredox Lights) are commercial products from Kessil, and the average intensity is 288 mW/cm^2 (measured from 1 cm distance). The range of LED lamp (λ_{max} = 440 nm) is from 410 nm to 480 nm, and the range of LED lamp (λ_{max} = 427 nm) is from 395 nm to 470 nm.

3. All starting acids and spiro products

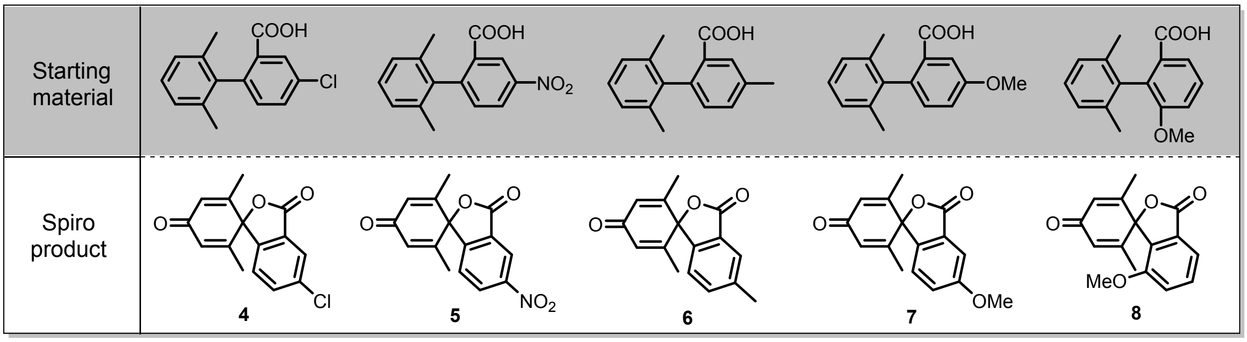

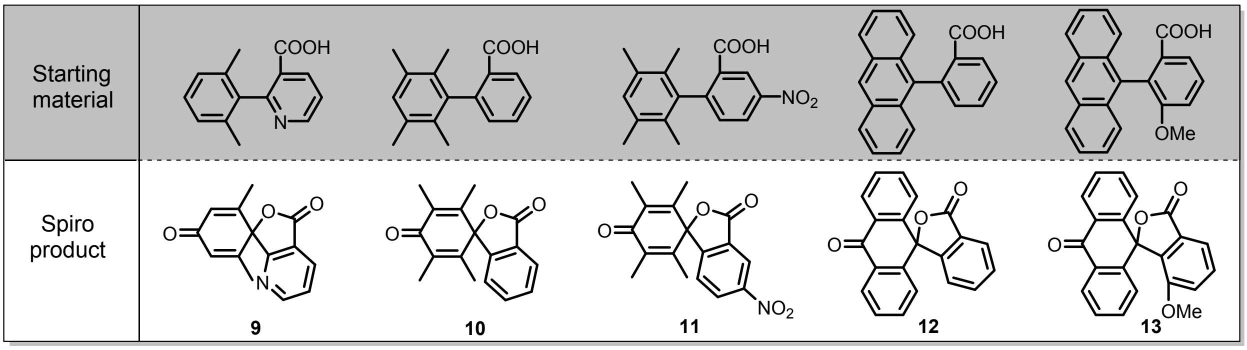

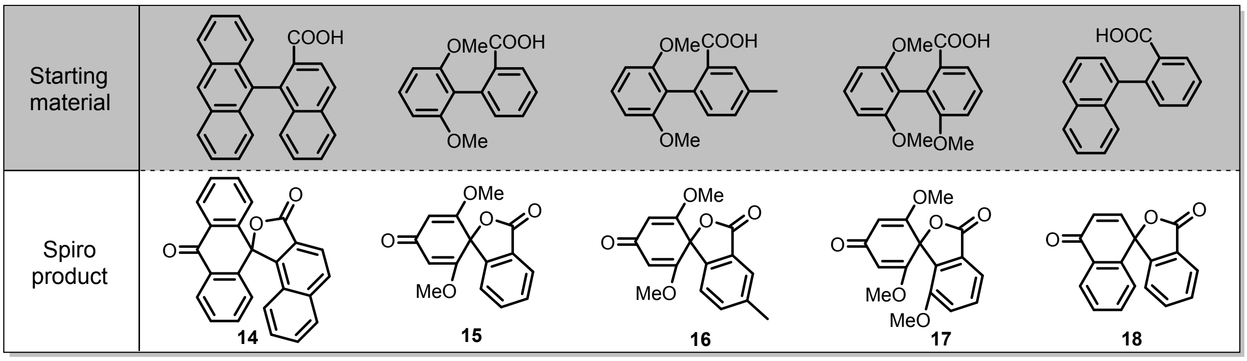

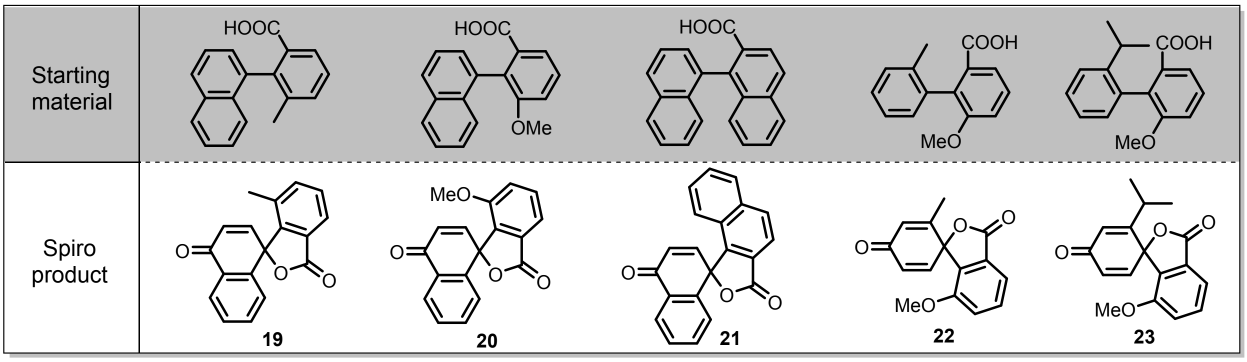

4. The synthesis of 4'-hydroxy-2',6'-dimethyl-[1,1'-biphenyl]-2-carboxylic acid (3)

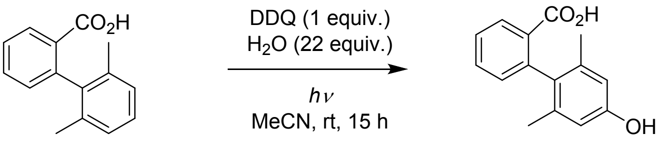

A 50 mL flask equipped with a stir bar was loaded with substrate **1** (1 mmol) and DDQ (1 mmol). After sealing the flask, the atmosphere was switched to argon, then acetonitrile (20 mL) with water (22 mmol) was added via syringe. The reaction mixture was irradiated at r.t. with 2 LED lamps (440 nm) for 15 h. After the completing of the reaction, reaction mixture was concentrated in vacuo and purified by flash chromatography over silica gel.

5. The synthesis of Methyl 2',6'-dimethyl-[1,1'-biphenyl]-2-carboxylate (24)

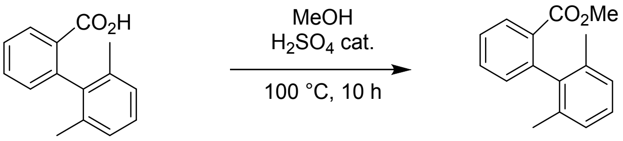

A 25 mL microwave vial (Biotage) equipped with a stir bar was loaded with substrate **1** (1 mmol), then the H_2SO_4 methanol solution (5%, 6 mL) was added. After sealing the vial, the reaction mixture was heated at 100 °C for 10 h. After the completing of the reaction, reaction mixture was concentrated in vacuo and purified by flash chromatography over silica gel.

6. General procedure conditions A and B

General procedure condition A: A 2-5 mL microwave vial (Biotage) equipped with a stir bar was loaded with the corresponding biarylacid (0.1 mmol), catalyst, and additives. Solvent was added and the vial was sealed. The reaction mixture was irradiated at r.t. with 2 LED lamps (427 nm) for specified time. After the completing of the reaction, reaction mixture was concentrated in vacuo and purified by flash chromatography over silica gel.

Procedure for the reaction under oxygen atmosphere condition A: A 2-5 mL microwave vial (Biotage) equipped with a stir bar was loaded with the corresponding biarylacid (0.1 mmol), catalyst, and additives. Solvent was added, the vial was sealed and flashed with oxygen for 5 mins. The reaction mixture was irradiated at r.t. with 2 LED lamps (427 nm) for specified time. After the completing of the reaction, reaction mixture was concentrated in vacuo and purified by flash chromatography over silica gel.

General procedure condition B: A 2-5 mL microwave vial (Biotage) equipped with a stir bar was loaded with the corresponding biarylacid (0.1 mmol) and DDQ (0.6 mmol). After sealing the vial, the atmosphere was switched to argon, then acetonitrile (4 mL) with water (2.2 mmol) was added using needle. The reaction mixture was irradiated at r.t. with 2 LED lamps (440 nm) for specified time. After the completing of the reaction, reaction mixture was concentrated in vacuo and purified by flash chromatography over silica gel.

General procedure for determination of the NMR yields of the products: For NMR yields, after the solvent was removed from the reaction mixture, 1, 3, 5-trimethoxybenzene was added as the standard. Then DMSO-d_6 or $CDCl_3$ was added to the residue and proton spectra was used to quantify to yields of products.

7. Optimization of Reaction Condition

Supplementary Table 1 Optimization of Reaction Condition[a]

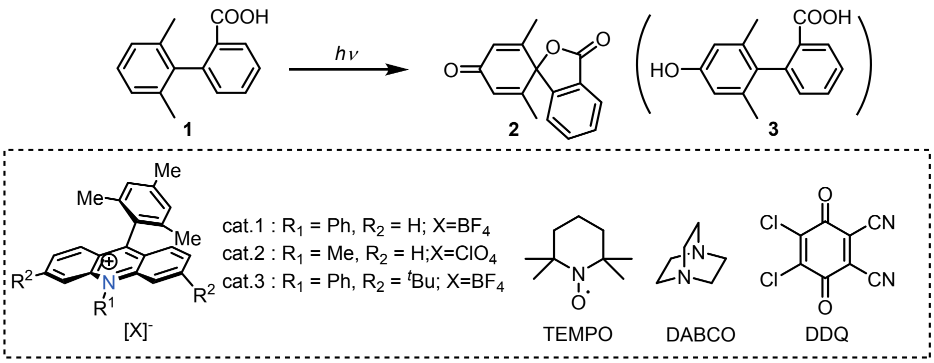

Entry	catalyst	amount of cat. (mol %)	Additive (equiv)	solvent	Yield **2**/**3**, %
1	cat.2	20	-	CH_3CN	trace/trace
2	cat.3	20	-	CH_3CN	5/trace
3	cat.1	20	-	DCE	trace/trace
4	cat.1	20	-	MeOH	10/trace
5	cat.1	20	Et_3N	Acetone	20/trace
6	cat.1	20	Py	Acetone	26/trace
7	cat.1	20	DBU	Acetone	35/trace
8	cat.1	20	KOH	Acetone	55/trace
9	cat.1	0	DABCO (1)/TEMPO (1)	Acetone	trace/trace
10[b]	cat.1	8	DABCO (1)/TEMPO (1)	Acetone	80/trace
11[c]	cat.1	8	DABCO (1)/TEMPO (1)	Acetone	trace/trace
12[d]	cat.1	8	DABCO (1)/TEMPO (1)	Acetone	trace/trace
13[e]	-	-	DDQ (3)	CH_3CN	49/21
14[e]	-	-	DDQ (5)	CH_3CN	77/12
15[e]	-	-	DDQ (6)	CH_3CN	89 (72)/6
16[f]	-	-	DDQ (6)	CH_3CN	trace/trace
17[g]	-	-	DDQ (6)	CH_3CN	26/trace

[a] Reactions were carried out at 0.05 mmol scale, with each additive in solvent (1 mL) under two LED lamps (λ_{max} = 427 nm) for 12 h. Air atmosphere. Yields were obtained by NMR relative to 1,3,5-trimethoxybenzene internal standard. [b] Oxygen atmosphere, 4 h. [c] Argon atmosphere, 4 h. [d] Dark reaction, 4 h. [e] LED lamps (λ_{max} = 440 nm) 15 h. Argon atmosphere, with addition of 22 equiv H_2O. [f] Dark reaction under argon atmosphere for 15 h with addition of 22 equiv H_2O. [g] LED lamps (λ_{max} = 440 nm) for 15 h. Oxygen atmosphere. Without addition of H_2O.

8. Attempts to use DDQ as a catalyst

Supplementary Table 2 Attempts to use DDQ as a catalyst

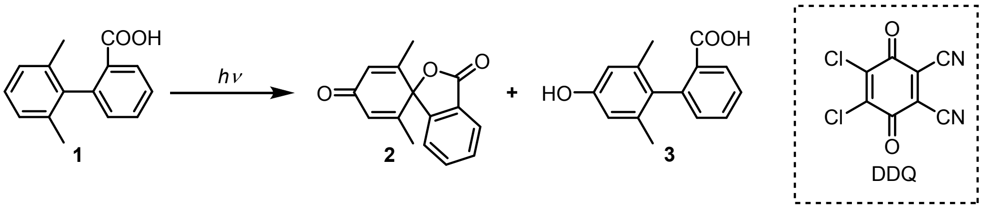

Entry	amount of DDQ (mol %)	Additive (equiv)	solvent	Atmosphere	Yield **2**/**3**, %
1	20	$NaNO_2$ (30%)	AcOH : CH_3CN (1:10)	O_2	2/21
2	20	$NaNO_2$ (30%)	AcOH : CH_2Cl_2 (1:10)	O_2	3/33
3	20	$NaNO_2$ (30%)	AcOH	O_2	2/30
4	100	$NaNO_2$ (30%)	AcOH : CH_2Cl_2 (1:10)	O_2	17/21
5	100	$NaNO_2$ (200%)	AcOH : CH_2Cl_2 (1:10)	O_2	29/trace
6	20	$Fe(NO_3)_3$ (26%)	CH_3CN	O_2	29/15
7	120	$Fe(NO_3)_3$ (100%)	CH_3CN	Ar	6/trace
8	20	$_t$BuONO (40%)	CH_3CN	O_2	28/trace
9	20	$_t$BuONO (200%)	CH_3CN	O_2	28/trace
10	100	$_t$BuONO (200%)	CH_3CN	O_2	26/trace

Reactions were carried out at 0.05 mmol scale, with each additive in solvent (1 mL) and 22 equiv H_2O under two LED lamps (λ_{max} = 440 nm) for 6-15 h. Yields were obtained by NMR relative to 1,3,5-trimethoxybenzene internal standard.

9. Electrochemical measurements

Electrochemical half peak redox potentials ($E_{p/2}$) were estimated from cyclic voltammograms. Measurements were performed in Ar-sparged acetonitrile with 0.1 M tetrabutylammonium hexafluorophosphate ($TBAPF_6$) as the electrolyte and a three electrode setup (working: glassy carbon, reference: saturated calomel electrode, counter: platinum). The potential was scanned from 0.5 V to a vertex potential of 2.8 V at a sweep rate of 20 mV/s. The half-wave potential for irreversible oxidation is estimated at $E_{p/2}$ the potential where the current is equal to one-half the peak current of the oxidation event.

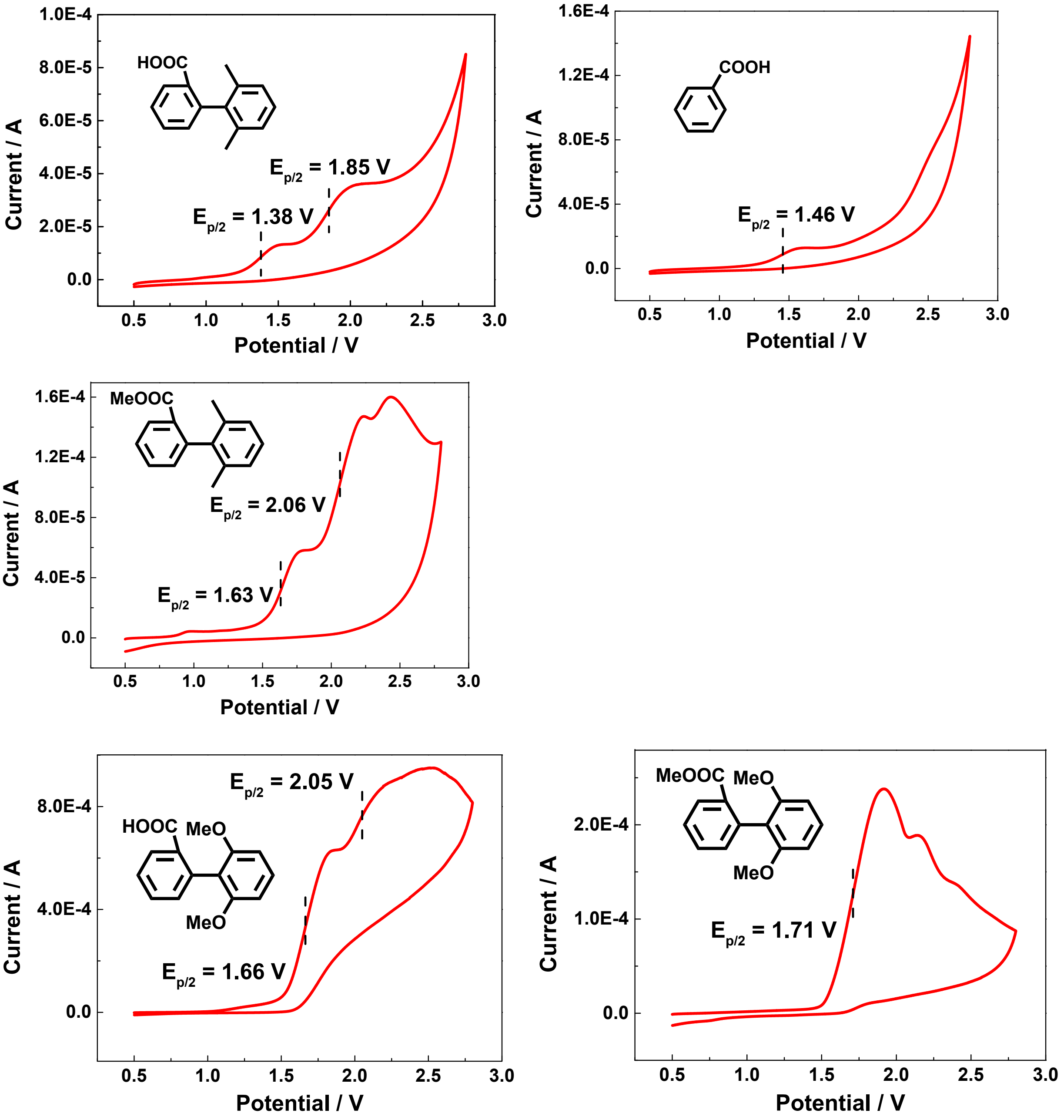

Figure S1 Cyclic voltammograms for selected compounds. The $E_{p/2}$ values shown on each plot referenced to SCE.

10. Control experiments

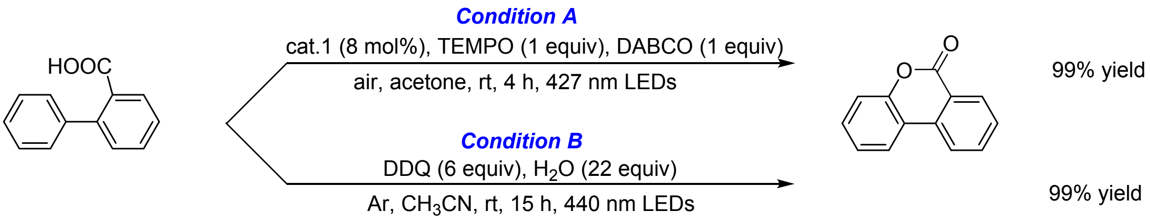

11. Isotope labelling experiments

11.1 Control experiment with standard reaction condition B (H_2O)

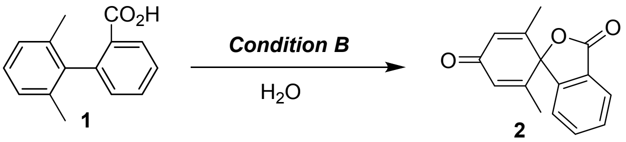

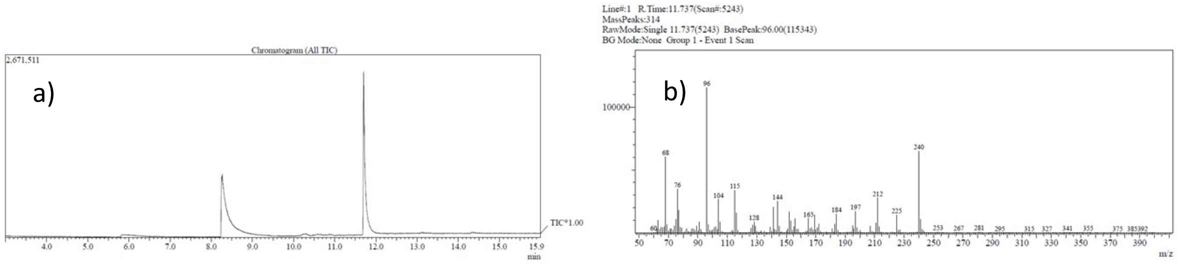

Figure S2. a) GS-MS chromatogram of experiment with standard reaction condition B (H_2O). b) Mass peaks at t = 11.7 min; MS of spiro product found *m/z* 240 (^{16}O).

11.2 Experiment using $^{18}O_2$ with reaction condition A

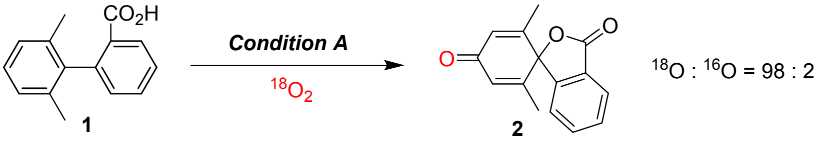

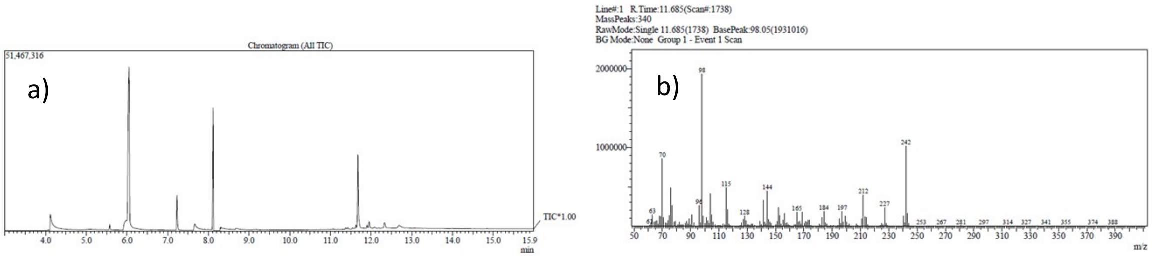

Figure S3. a) GS-MS chromatogram of experiment using $^{18}O_2$ with reaction condition A. b) Mass peaks at t = 11.7 min; MS of spiro products found *m/z* 242 : 240 (^{18}O : ^{16}O = 98 : 2).

11.3 Experiment using $H_2{}^{18}O$ with reaction condition B

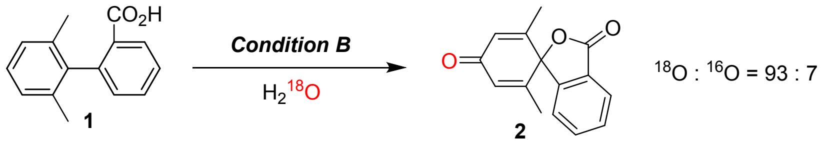

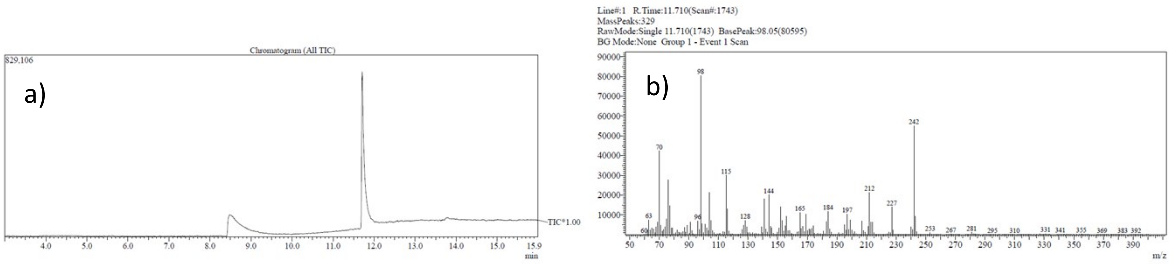

Figure S4. a) GS-MS chromatogram of experiment using $H_2{}^{18}O$ with reaction condition B. b) Mass peaks at t = 11.7 min; MS of spiro products found *m/z* 242 : 240 (^{18}O : ^{16}O = 93 : 7).

12. Further transformations of spirolactone

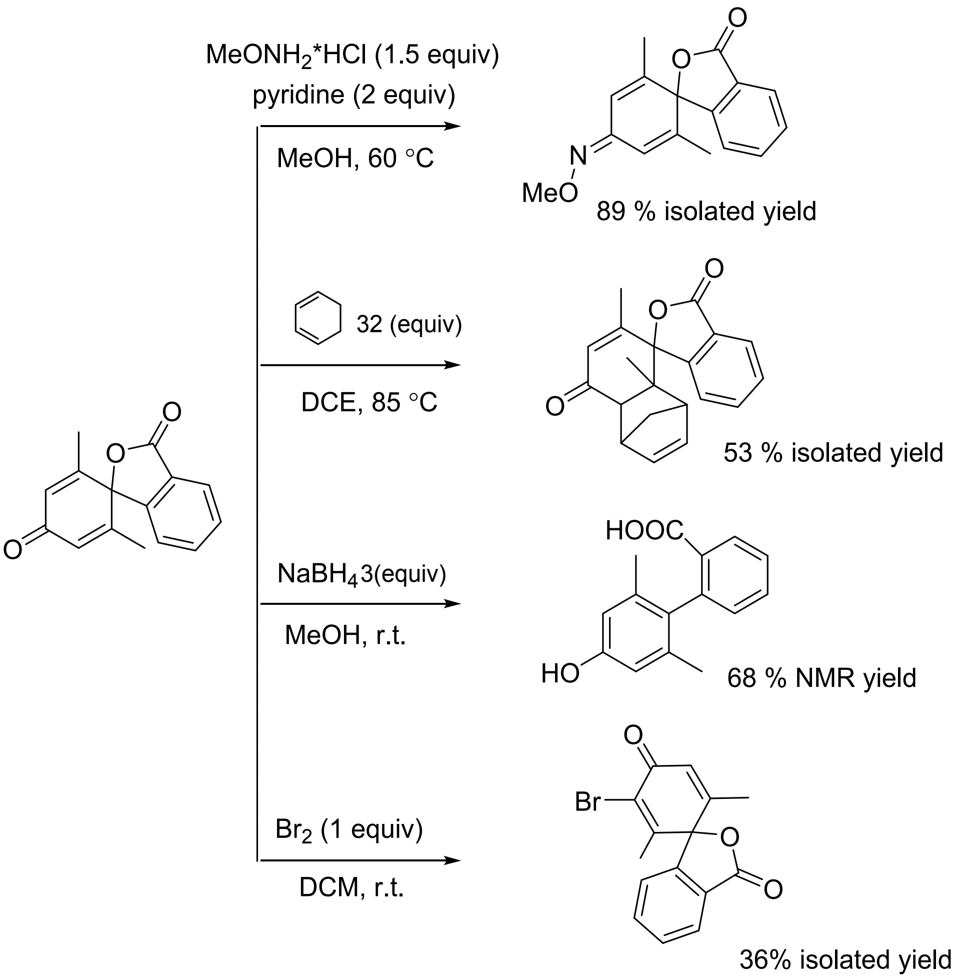

Figure S5. Further transformations of spirolactone. These reactions were run on 0.1 mmol scale.

13. General method A to synthesize starting biaryl acid 1

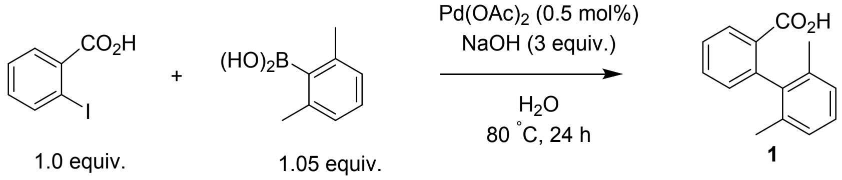

A 250 mL flask (Biotage) equipped with a stir bar was loaded with 2-iodobenzoic acid (2480 mg, 10 mmol, 1.0 equiv.), boronic acid (1580 mg, 10.5 mmol. 1.05 equiv.), NaOH (1200 mg, 30 mmol, 3.0 equiv.), $Pd(OAc)_2$ (11.2 mg, 0.5 mol%), and water (100mL). After sealing the vial and flashed with argon, the reaction mixture was heated at 80 °C for 24 h. After completion monitored by TLC, the reaction mixture was then cooled down to room temperature. Water was added and the reaction mixture was extracted with CH_2Cl_2 (3×20 mL). The organic layer was separated, washed with brine, dried over sodium sulfate, concentrated, and purified by silica gel column chromatography to provide biaryl acid **1** (1700 mg, 75%).

14. General method B to synthesize starting biaryl acid A13

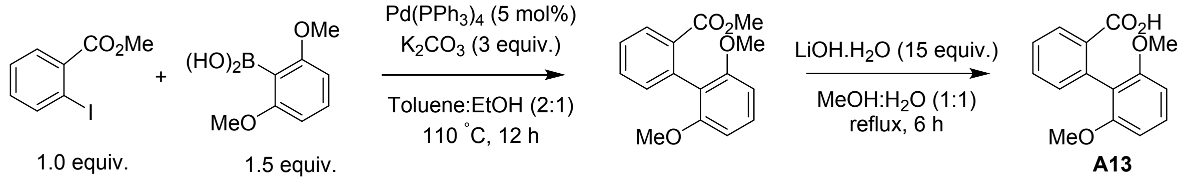

A 25 mL microwave vial (Biotage) equipped with a stir bar was loaded with methyl 2-iodobenzoate (500 mg, 1.9 mmol, 1.0 equiv.), boronic acid (520 mg, 2.9 mmol. 1.5 equiv.), K_2CO_3 (790 mg, 5.7 mmol, 3.0 equiv.), $Pd(PPh_3)_4$ (110 mg, 5 mol%), and solvents (toluene: EtOH, 6+3 mL, 2:1). After sealing the vial and flashed with argon, the reaction mixture was heated at 110 °C for 12 h. After completion monitored by TLC, the reaction mixture was then cooled down to room temperature. Water was added and the reaction mixture was extracted by CH_2Cl_2 (3×20 mL). The organic layer was separated, washed with brine, dried over sodium sulfate, concentrated, and purified by silica gel column chromatography to provide biaryl ester which was used directly for the next synthetic step without further purification.

A mixture of ester suspended in methanol (30 mL), water (10 mL) and $LiOH{\cdot}H_2O$ (1.196 g, 28.5 mmol, 15.0 equiv.) was refluxed for 6 hr. After completion monitored by TLC, the resulting mixture was then acidified with 30 ml of 30 % aq. solution of HCl, and extracted with CH_2Cl_2 (3×20 mL). The organic layer was separated, washed with brine, dried over sodium sulfate, concentrated, and purified by silica gel column chromatography to provide biaryl acid **A13** (319 mg, 65% overall yield).

15. General method C to synthesize starting hindered biaryl acid A6

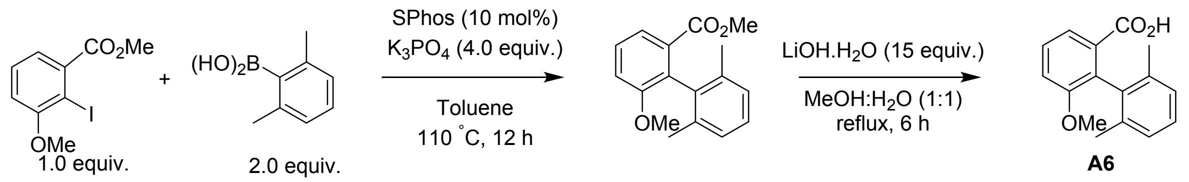

An oven dried 25 mL microwave vial (Biotage) equipped with a stir bar was loaded with methyl 2-iodo-3-methoxybenzoate (500 mg, 1.7 mmol, 1.0 equiv.), boronic acid (513 mg, 3.4 mmol. 2.0 equiv.), anhydrous K_3PO_4 (1.453 g, 6.8 mmol, 4.0 equiv.), $Pd(OAc)_2$ (19 mg, 5.0 mol%), ligand (SPhos, 2-Dicyclohexylphosphino-2′,6′-dimethoxybipheny) (70 mg, 10 mol%), and dry toluene (10 mL). After sealing the vial and flashed with argon, the reaction mixture was heated at 110 °C for 24 h. After completion monitored by TLC, the reaction mixture was then cooled down to room temperature. Water was added and the reaction mixture was extracted by CH_2Cl_2 (3×20 mL). The organic layer was separated, washed with brine, dried over sodium sulfate, concentrated, and purified by silica gel column chromatography to provide biaryl ester which was used directly for the next synthetic step without further purification.

A mixture of ester suspended in methanol (30 mL), water (10 mL) and $LiOH{\cdot}H_2O$ (1.070 g, 25.5 mmol, 15.0 equiv.) was refluxed for 6 hr. After completion monitored by TLC, the resulting mixture was then acidified with 30 ml of 30 % aq. solution of HCl, and extracted with CH_2Cl_2 (3×20 mL). The organic layer was separated, washed with brine, dried over sodium sulfate, concentrated, and purified by silica gel column chromatography to provide biaryl acid **A6** (152 mg, 35% overall yield).

16. Characterization data of all starting acids

2',6'-dimethyl-[1,1'-biphenyl]-2-carboxylic acid (1)[1]

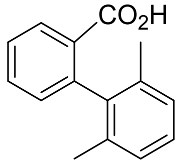

Using the general method A, acid **1** was obtained in 170 mg (75%) as a white solid.

^{1}H NMR (400 MHz, $CDCl_3$) δ = 11.15 (s, 1H), 8.12 (dd, *J* = 7.9, 1.4 Hz, 1H), 7.63 (td, *J* = 7.5, 1.4 Hz, 1H), 7.46 (td, *J* = 7.6, 1.3 Hz, 1H), 7.23 – 7.13 (m, 2H), 7.13 – 7.05 (m, 2H), 1.96 (s, 6H).

^{13}C NMR (101 MHz, $CDCl_3$) δ = 171.8, 142.8, 140.8, 135.1, 133.0, 131.2, 130.8, 128.8, 127.1, 126.9, 20.5.

4-chloro-2',6'-dimethyl-[1,1'-biphenyl]-2-carboxylic acid (A2)

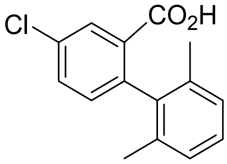

Using the general method B, acid **A2** was obtained in 285 mg (65%) as a white solid.

^{1}H NMR (400 MHz, $CDCl_3$) δ = 10.98 (s, 1H), 8.10 (d, *J* = 2.3 Hz, 1H), 7.60 (dd, *J* = 8.2, 2.3 Hz, 1H), 7.19 (dd, *J* = 8.2, 6.8 Hz, 1H), 7.11 (dd, *J* = 11.0, 7.9 Hz, 3H), 1.95 (s, 6H).

^{13}C NMR (101 MHz, $CDCl_3$) δ = 170.6, 141.3, 139.5, 135.1, 133.1, 132.3, 131.2, 130.3, 127.3, 127.1, 20.5.

2',6'-dimethyl-4-nitro-[1,1'-biphenyl]-2-carboxylic acid (A3)

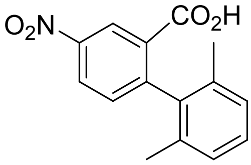

Using the general method B, acid **A3** was obtained in 313 mg (60%) as a light yellow solid.

^{1}H NMR (400 MHz, $CDCl_3$) δ = 10.50 (s, 1H), 8.96 (d, *J* = 2.4 Hz, 1H), 8.48 (dd, *J* = 8.4, 2.5 Hz, 1H), 7.41 (d, *J* = 8.4 Hz, 1H), 7.25 – 7.19 (m, 1H), 7.17 – 7.08 (m, 2H), 1.95 (s, 6H).

^{13}C NMR (101 MHz, $CDCl_3$) δ = 169.8, 169.8, 149.7, 146.9, 138.6, 134.4, 132.6, 130.4, 127.9, 127.3, 126.4, 20.5.

2',4,6'-trimethyl-[1,1'-biphenyl]-2-carboxylic acid (A4)

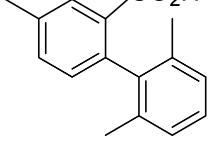

Using the general method B, acid **A4** was obtained in 313 mg (68%) as a white solid.

^{1}H NMR (400 MHz, $CDCl_3$) δ = 7.91 – 7.87 (m, 1H), 7.40 (ddd, *J* = 7.8, 2.0, 0.9 Hz, 1H), 7.14 (dd, *J* = 8.4, 6.6 Hz, 1H), 7.06 (d, *J* = 7.8 Hz, 2H), 7.01 (d, *J* = 7.7 Hz, 1H), 2.45 (s, 3H), 1.95 (s, 6H).

^{13}C NMR (101 MHz, $CDCl_3$) δ = 171.1, 141.2, 139.2, 136.8, 135.7, 133.3, 131.4, 130.5, 129.6, 126.9, 126.7, 21.0, 20.6.

4-methoxy-2',6'-dimethyl-[1,1'-biphenyl]-2-carboxylic acid (A5)

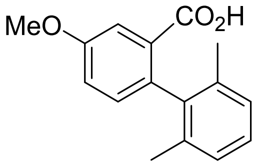

Using the general method B, acid **A5** was obtained in 287 mg (55%) as a white solid.

^{1}H NMR (400 MHz, $CDCl_3$) δ = 10.54 (s, 1H), 7.63 (d, *J* = 2.8 Hz, 1H), 7.21 – 7.13 (m, 2H), 7.09 (dq, *J* = 7.2, 0.7 Hz, 2H), 7.06 (d, *J* = 8.4 Hz, 1H), 3.91 (s, 3H), 1.97 (s, 6H).

^{13}C NMR (101 MHz, $CDCl_3$) δ = 170.8, 158.4, 140.4, 135.8, 135.0, 131.9, 129.5, 127.0, 126.9, 119.8, 115.2, 55.5, 20.6.

6-methoxy-2',6'-dimethyl-[1,1'-biphenyl]-2-carboxylic acid (A6)

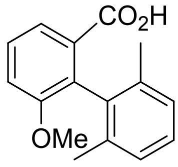

Using the general method C, acid **A6** was obtained in 152 mg (35%) as a white solid.

^{1}H NMR (400 MHz, $CDCl_3$) δ = 7.67 (dd, *J* = 7.9, 1.1 Hz, 1H), 7.44 (t, *J* = 8.1 Hz, 1H), 7.24 – 7.14 (m, 2H), 7.10 (d, *J* = 7.2 Hz, 2H), 3.75 (s, 3H), 1.95 (s, 6H).

^{13}C NMR (101 MHz, $CDCl_3$) δ = 170.8, 158.4, 140.4, 135.8, 135.0, 131.9, 129.6, 127.0, 126.9, 119.8, 115.2, 55.5, 20.6.

2-(2,6-dimethylphenyl)nicotinic acid (A7)

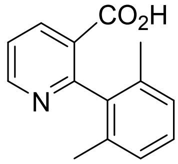

Using the general method C, acid **A7** was obtained in 200 mg (38%) as a yellow solid.

^{1}H NMR (400 MHz, $CDCl_3$) δ 8.49 (d, *J* = 2.0 Hz, 1H), 8.47 (d, *J* = 2.0 Hz, 1H), 8.41 (dd, *J* = 4.9, 2.0 Hz, 2H), 7.15 (dd, *J* = 7.6, 4.9 Hz, 2H), 4.22 (s, 6H).

^{13}C NMR (101 MHz, $CDCl_3$) δ = 164.6, 161.2, 151.8, 143.1, 127.3, 118.4, 112.4, 54.8.

2',3',5',6'-tetramethyl-[1,1'-biphenyl]-2-carboxylic acid (A8)

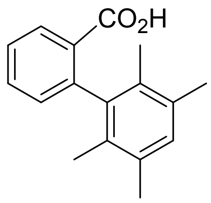

Using the general method B, acid **A8** was obtained in 340 mg (70%) as a white solid.

^{1}H NMR (400 MHz, $CDCl_3$) δ = 10.43 (s, 1H), 8.15 (dd, *J* = 7.9, 1.5 Hz, 1H), 7.62 (td, *J* = 7.5, 1.4 Hz, 1H), 7.47 (td, *J* = 7.6, 1.3 Hz, 1H), 7.14 (dd, *J* = 7.6, 1.3 Hz, 1H), 7.01 (s, 1H), 2.28 (s, 6H), 1.83 (s, 6H).

^{13}C NMR (101 MHz, $CDCl_3$) δ = 170.9, 144.0, 140.4, 133.2, 132.9, 131.3, 131.2, 130.6, 128.9, 126.9, 20.1, 17.0.

2',3',5',6'-tetramethyl-4-nitro-[1,1'-biphenyl]-2-carboxylic acid (A9)

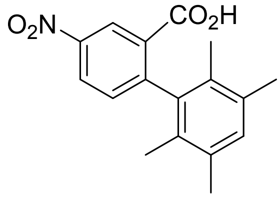

Using the general method B, acid **A19** was obtained in 322 mg (56%) as a white solid.

^{1}H NMR (400 MHz, $CDCl_3$) δ = 8.96 (d, *J* = 2.5 Hz, 1H), 8.45 (dd, *J* = 8.4, 2.5 Hz, 1H), 7.36 (d, *J* = 8.4 Hz, 1H), 7.05 (s, 1H), 2.28 (s, 6H), 1.80 (s, 6H).

^{13}C NMR (101 MHz, $CDCl_3$) δ = 167.8, 150.9, 146.8, 138.5, 133.7, 132.8, 131.4, 130.6, 130.5, 127.0, 126.3, 20.0, 17.0.

2-(anthracen-9-yl)benzoic acid (A10)[2]

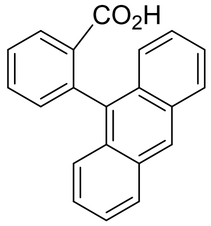

Using the general method C, acid **A10** was obtained in 227 mg (40%) as a light yellow solid.

^{1}H NMR (400 MHz, $CDCl_3$) δ = 8.50 (s, 1H), 8.23 (ddd, *J* = 7.9, 1.5, 0.5 Hz, 1H), 8.05 (ddt, *J* = 8.5, 1.3, 0.7 Hz, 2H), 7.73 (td, *J* = 7.5, 1.5 Hz, 1H), 7.67 – 7.57 (m, 1H), 7.45 (ddd, *J* = 8.5, 6.4, 1.2 Hz, 2H), 7.42 – 7.39 (m, 2H), 7.37 (ddd, *J* = 7.6, 1.4, 0.5 Hz, 1H), 7.32 (dd, *J* = 6.4, 1.3 Hz, 1H), 7.30 (dd, *J* = 6.4, 1.3 Hz, 1H).

^{13}C NMR (101 MHz, $CDCl_3$) δ = 168.8, 140.5, 135.8, 133.0, 132.7, 131.4, 131.1, 130.5, 129.9, 128.4, 128.0, 126.6, 126.1, 125.4, 125.0.

2-(anthracen-9-yl)-3-methoxybenzoic acid (A11)

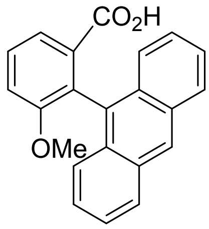

Using the general method C, acid **A11** was obtained in 157 mg (28%) as a light yellow solid.

^{1}H NMR (400 MHz, $CDCl_3$) δ = 8.49 (s, 1H), 8.08 – 8.02 (m, 2H), 7.83 (dd, *J* = 7.9, 1.1 Hz, 1H), 7.63 (s, 1H), 7.47 – 7.41 (m, 4H), 7.36 – 7.28 (m, 5H), 3.57 (s, 3H).

^{13}C NMR (101 MHz, $CDCl_3$) δ = 169.0, 158.4, 132.2, 131.6, 131.3, 130.2, 129.2, 128.7, 128.5, 126.5, 125.8, 125.3, 124.9, 123.2, 115.4, 56.2.

1-(anthracen-9-yl)-2-naphthoic acid (A12)

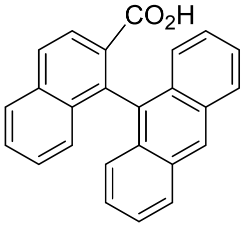

Using the general method C, acid **A12** was obtained in 144 mg (22%) as a yellow solid.

^{1}H NMR (400 MHz, $CDCl_3$) δ = 8.60 (s, 1H), 8.26 (d, *J* = 8.7 Hz, 1H), 8.15 – 8.07 (m, 3H), 8.02 (d, *J* = 8.2 Hz, 1H), 7.56 (ddd, *J* = 8.1, 6.8, 1.2 Hz, 1H), 7.45 (ddd, *J* = 8.5, 5.4, 2.2 Hz, 2H), 7.27 – 7.15 (m, 5H), 6.95 (dd, *J* = 8.6, 1.0 Hz, 1H).

^{13}C NMR (101 MHz, $CDCl_3$) δ = 169.8, 139.9, 135.4, 133.4, 133.1, 131.2, 130.7, 128.5, 128.4, 128.2, 128.1, 127.9, 127.0, 126.9, 26.5, 126.1, 125.7, 125.1.

2',6'-dimethoxy-[1,1'-biphenyl]-2-carboxylic acid (A13)[3]

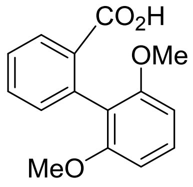

Using the general method B, acid **A13** was obtained in 384 mg (78%) as a white solid.

^{1}H NMR (400 MHz, $CDCl_3$) δ = 8.03 (dd, *J* = 7.8, 1.4 Hz, 1H), 7.58 (td, *J* = 7.5, 1.5 Hz, 1H), 7.41 (td, *J* = 7.6, 1.3 Hz, 1H), 7.36 (dd, *J* = 7.7, 1.3 Hz, 1H), 7.33 – 7.26 (m, 1H), 6.64 (d, *J* = 8.4 Hz, 2H), 3.71 (s, 6H).

^{13}C NMR (101 MHz, $CDCl_3$) δ = 172.4, 157.0, 135.3, 132.7, 131.8, 130.7, 130.2, 128.9, 127.0, 118.6, 104.2, 55.8.

2',6'-dimethoxy-4-methyl-[1,1'-biphenyl]-2-carboxylic acid (A14)

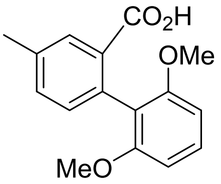

Using the general method B, acid **A14** was obtained in 415 mg (70%) as a white solid.

^{1}H NMR (400 MHz, $CDCl_3$) δ = 11.01 (s, 1H), 7.87 – 7.81 (m, 1H), 7.40 (ddd, *J* = 7.8, 1.9, 0.8 Hz, 1H), 7.32 – 7.26 (m, 1H), 7.25 (d, *J* = 7.8 Hz, 1H), 6.64 (d, *J* = 8.4 Hz, 2H), 3.71 (s, 6H), 2.44 (s, 3H).

^{13}C NMR (101 MHz, $CDCl_3$) δ = 172.6, 157.1, 136.8, 132.8, 132.5, 132.2, 130.8, 130.4, 128.7, 118.5, 104.2, 104.2, 55.8, 21.1.

2',6,6'-trimethoxy-[1,1'-biphenyl]-2-carboxylic acid (A15)

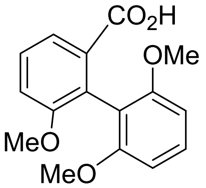

Using the general method C, acid **A15** was obtained in 148 mg (30%) as a white solid.

^{1}H NMR (400 MHz, $CDCl_3$) δ = 10.21 (s, 2H), 7.62 (dd, *J* = 7.8, 1.1 Hz, 1H), 7.41 (t, *J* = 8.0 Hz, 1H), 7.32 (t, *J* = 8.4 Hz, 1H), 7.18 (dd, *J* = 8.3, 1.2 Hz, 1H), 6.65 (d, *J* = 8.4 Hz, 2H), 3.76 (s, 3H), 3.71 (s, 6H).

^{13}C NMR (101 MHz, $CDCl_3$) δ = 170.9, 157.7, 157.6, 132.0, 129.1, 128.2, 124.6, 122.5, 115.2, 114.1, 104.3, 56.3, 55.9.

2-(naphthalen-1-yl)benzoic acid (A16)[4]

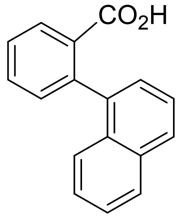

Using the general method B, acid **A16** was obtained in 379 mg (80%) as a white solid.

^{1}H NMR (400 MHz, $CDCl_3$) δ = 9.59 (s, 1H), 8.06 (dd, *J* = 7.9, 1.4 Hz, 1H), 7.91 (d, *J* = 8.4 Hz, 1H), 7.87 (d, *J* = 8.3 Hz, 1H), 7.63 (td, *J* = 7.5, 1.4 Hz, 1H), 7.56 – 7.44 (m, 4H), 7.42 – 7.33 (m, 2H), 7.33 – 7.27 (m, 1H).

^{13}C NMR (101 MHz, $CDCl_3$) δ = 172.1, 141.9, 139.2, 133.2, 132.2, 132.1, 131.9, 130.8, 130.1, 128.1, 127.6, 127.5, 125.9, 125.5, 125.5, 125.0.

3-methyl-2-(naphthalen-1-yl)benzoic acid (A17)

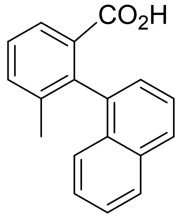

Using the general method C, acid **A17** was obtained in 257 mg (45%) as a white solid.

^{1}H NMR (400 MHz, $CDCl_3$) δ = 10.08 (s, 1H), 7.94 – 7.88 (m, 2H), 7.86 (dd, *J* = 8.2, 1.1 Hz, 1H), 7.49 (dddd, *J* = 16.1, 8.1, 6.5, 1.1 Hz, 3H), 7.42 (t, *J* = 7.7 Hz, 1H), 7.35 (ddd, *J* = 8.0, 6.6, 1.3 Hz, 1H), 7.32 – 7.26 (m, 2H), 7.20 (dd, *J* = 7.0, 1.2 Hz, 1H), 1.92 (s, 3H).

^{13}C NMR (101 MHz, $CDCl_3$) δ = 171.2, 140.9, 138.6, 137.8, 134.1, 133.4, 131.9, 130.4, 128.4, 128.2, 127.5, 127.4, 126.0, 125.8, 125.6, 125.3, 125.3, 20.3.

3-methoxy-2-(naphthalen-1-yl)benzoic acid (A18)[5]

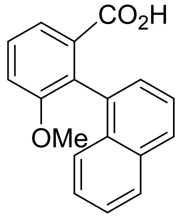

Using the general method C, acid **A18** was obtained in 187 mg (33%) as a light yellow solid.

^{1}H NMR (400 MHz, $CDCl_3$) δ = 9.84 (s, 1H), 7.91 – 7.84 (m, 2H), 7.62 (dd, *J* = 7.9, 1.1 Hz, 1H), 7.52 – 7.48 (m, 2H), 7.46 (d, *J* = 4.7 Hz, 2H), 7.37 – 7.34 (m, 2H), 7.25 (dd, *J* = 7.0, 1.2 Hz, 1H), 7.21 (dd, *J* = 8.3, 1.2 Hz, 1H), 3.65 (s, 3H).

^{13}C NMR (101 MHz, $CDCl_3$) δ = 170.8, 157.8, 134.6, 133.2, 132.4, 131.9, 130.3, 128.8, 128.2, 127.7, 126.6, 125.7, 125.5, 125.4, 125.2, 122.6, 114.8, 56.1.

[1,1'-binaphthalene]-2-carboxylic acid (A19)[6]

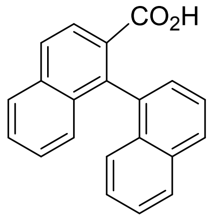

Using the general method C, acid **A19** was obtained in 315 mg (56%) as a white solid.

^{1}H NMR (400 MHz, $CDCl_3$) δ 8.10 (d, *J* = 8.7 Hz, 1H), 8.00 (d, *J* = 8.7 Hz, 1H), 7.98 – 7.93 (m, 3H), 7.56 (ddd, *J* = 8.1, 6.5, 1.3 Hz, 2H), 7.47 (ddd, *J* = 8.1, 6.7, 1.2 Hz, 1H), 7.36 – 7.32 (m, 1H), 7.31 (dd, *J* = 8.6, 1.3 Hz, 1H), 7.28 – 7.22 (m, 2H), 7.18 (s, 1H).

^{13}C NMR (101 MHz, $CDCl_3$) δ = 171.1, 141.1, 136.5, 135.2, 133.3, 133.2, 132.9, 128.2, 128.2, 128.1, 127.9, 127.8, 127.0, 126.7, 126.2, 126.1, 125.9, 125.7, 125.2.

6-methoxy-2'-methyl-[1,1'-biphenyl]-2-carboxylic acid (A20)

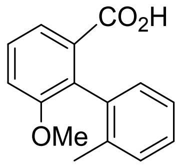

Using the general method B, acid **A20** was obtained in 282 mg (68%) as a white solid.

^{1}H NMR (400 MHz, $CDCl_3$) δ = 7.59 (dd, *J* = 7.8, 1.1 Hz, 1H), 7.42 (t, *J* = 8.1 Hz, 1H), 7.28 – 7.24 (m, 2H), 7.16 (dd, *J* = 8.3, 1.1 Hz, 1H), 7.03 (dd, *J* = 7.2, 1.2 Hz, 1H), 3.76 (s, 3H), 2.07 (s, 3H).

^{13}C NMR (101 MHz, $CDCl_3$) δ = 171.7, 157.1, 136.6, 136.2, 131.7, 131.1, 129.2, 128.9, 128.3, 127.4, 125.2, 122.3, 114.6, 56.0, 19.8.

2'-isopropyl-6-methoxy-[1,1'-biphenyl]-2-carboxylic acid (A21)

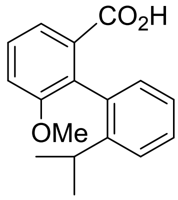

Using the general method B, acid **A21** was obtained in 259 mg (56%) as a light yellow solid.

^{1}H NMR (400 MHz, $CDCl_3$) δ = 7.59 (dd, *J* = 7.9, 1.1 Hz, 1H), 7.42 (t, *J* = 8.0 Hz, 1H), 7.38 – 7.34 (m, 2H), 7.18 (ddd, *J* = 7.5, 4.9, 3.6 Hz, 1H), 7.14 (dd, *J* = 8.3, 1.1 Hz, 1H), 6.98 (dt, *J* = 7.5, 1.0 Hz, 1H), 3.74 (s, 3H), 2.59 (p, *J* = 6.9 Hz, 1H), 1.15 (d, *J* = 6.9 Hz, 3H), 1.08 (d, *J* = 6.9 Hz, 3H).

^{13}C NMR (101 MHz, $CDCl_3$) δ = 171.8, 157.3, 147.2, 134.8, 131.8, 129.0, 128.2, 127.8, 125.0, 124.9, 122.2, 114.2, 55.7, 30.7, 23.8, 23.7.

17. Characterization data of all products

2,6-dimethyl-3'*H*-spiro[cyclohexane-1,1'-isobenzofuran]-2,5-diene-3',4-dione (2)

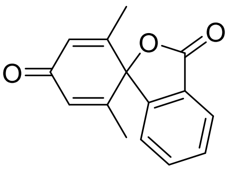

^{1}H NMR (400 MHz, $CDCl_3$) δ = 8.01 (d, J = 7.6 Hz, 1H), 7.73 (t, J = 8.1 Hz, 1H), 7.65 (t, J = 7.5 Hz, 1H), 7.21 (d, J = 7.7 Hz, 1H), 6.28 (s, 2H), 1.66 (s, 6H).

^{13}C NMR (101 MHz, $CDCl_3$) δ = 184.9, 169.8, 154.5, 148.0, 135.4, 130.6, 128.2, 126.3, 126.1, 121.4, 85.0, 17.1.

IR (cm^{-1}) ν 2978, 1482, 1270, 1245, 1210, 760, 730

HRMS (ESI) calcd. for $C_{15}H_{12}NaO_3$ [M+Na] *m/z* 263.0684 found *m/z* 263.0679.

4'-hydroxy-2',6'-dimethyl-[1,1'-biphenyl]-2-carboxylic acid (3)

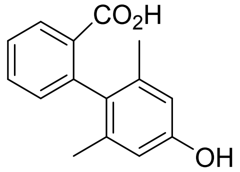

^{1}H NMR (400 MHz, $CDCl_3$) δ = 8.04 (d, J = 7.8 Hz, 1H), 7.56 (t, J = 8.2 Hz, 1H), 7.42 (t, J = 7.6 Hz, 1H), 7.14 (d, J = 7.6 Hz, 1H), 6.58 (s, 2H), 1.89 (s, 6H).

^{13}C NMR (101 MHz, $CDCl_3$) δ = 170.6, 155.0, 142.1, 136.8, 132.7, 132.2, 131.4, 130.7, 126.9, 114.1, 20.7.

HRMS (ESI) calcd. for $C_{15}H_{13}O_3$ [M-H] *m/z* 241.0863 found *m/z* 241.0870.

5'-chloro-2,6-dimethyl-3'*H*-spiro[cyclohexane-1,1'-isobenzofuran]-2,5-diene-3',4-dione (4)

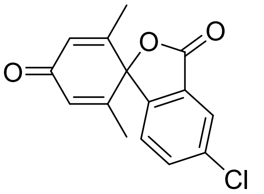

^{1}H NMR (400 MHz, $CDCl_3$) δ = 7.97 (s, 1H), 7.69 (d, J = 8.2 Hz, 1H), 7.16 (d, J = 8.2 Hz, 1H), 6.29 (s, 2H), 1.68 (s, 6H).

^{13}C NMR (101 MHz, $CDCl_3$) δ = 184.6, 168.3, 153.8, 146.2, 137.0, 135.7, 128.5, 127.9, 126.2, 122.7, 84.9, 17.1.

IR (cm^{-1}) ν 1710, 1674, 1637, 1594, 1460, 1093, 1066, 1007, 945, 833, 782, 754

HRMS (ESI) calcd. for $C_{15}H_{12}ClO_3$ [M+H] *m/z* 275.0469 found *m/z* 275.0464.

2,6-dimethyl-5'-nitro-3'*H*-spiro[cyclohexane-1,1'-isobenzofuran]-2,5-diene-3',4-dione (5)

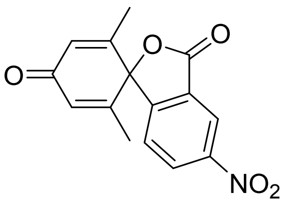

^{1}H NMR (400 MHz, $CDCl_3$) δ = 8.84 (s, 1H), 8.60 (d, *J* = 8.4 Hz, 1H), 7.43 (d, *J* = 7.8 Hz, 1H), 6.35 (s, 2H), 1.70 (s, 6H).

^{13}C NMR (101 MHz, $CDCl_3$) δ = 184.2, 167.2, 153.3, 152.7, 149.9, 130.2, 129.0, 127.9, 123.0, 121.9, 85.2, 17.2.

IR (cm^{-1}) ν 1675, 1640, 1614, 1536, 1435, 1384, 1108, 1004, 950, 907, 813, 747, 662

HRMS (ESI) calcd. for $C_{15}H_{12}NO_5$ [M+H] *m/z* 286.0710 found *m/z* 286.0706.

2,5',6-trimethyl-3'*H*-spiro[cyclohexane-1,1'-isobenzofuran]-2,5-diene-3',4-dione (6)

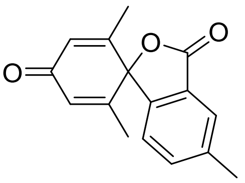

^{1}H NMR (400 MHz, $CDCl_3$) δ = 7.79 (s, 1H), 7.53 (d, *J* = 7.8 Hz, 1H), 7.08 (d, *J* = 7.9 Hz, 1H), 6.26 (s, 2H), 2.52 (s, 3H), 1.66 (s, 6H).

^{13}C NMR (101 MHz, $CDCl_3$) δ = 185.0, 170.1, 154.8, 145.4, 141.2, 136.5, 128.1, 126.3, 121.1, 84.9, 21.4, 17.2.

IR (cm^{-1}) ν 1711, 1673, 1642, 1595, 1489, 1285, 1162, 1081, 1036, 1001, 946, 888, 837, 790, 705

HRMS (ESI) calcd. for $C_{16}H_{14}NaO_3$ [M+Na] *m/z* 277.0835 found *m/z* 277.0834.

5'-methoxy-2,6-dimethyl-3'*H*-spiro[cyclohexane-1,1'-isobenzofuran]-2,5-diene-3',4-dione (7)

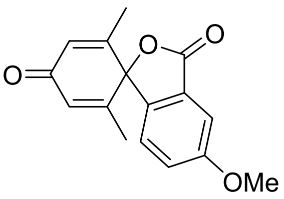

^{1}H NMR (500 MHz, $CDCl_3$) δ = 7.43 (d, *J* = 2.2 Hz, 1H), 7.28 (dd, *J* = 8.5, 2.3 Hz, 1H), 7.10 (d, *J* = 8.5 Hz, 1H), 6.28 (s, 2H), 3.95 (s, 3H), 1.69 (s, 6H).

^{13}C NMR (101 MHz, $CDCl_3$) δ = 184.9, 169.9, 161.7, 154.7, 139.9, 128.1, 127.6, 124.1, 122.3, 108.1, 84.8, 55.9, 17.1.

IR (cm-1) ν 2970, 1738, 1711, 1674, 1641, 1490, 1435, 1283, 1079, 1036, 1000, 948, 903, 838, 773, 681

HRMS (ESI) calcd. for $C_{16}H_{14}NaO_4$ [M+Na] *m/z* 293.0784 found *m/z* 293.0778.

7'-methoxy-2,6-dimethyl-3'*H*-spiro[cyclohexane-1,1'-isobenzofuran]-2,5-diene-3',4-dione (8)

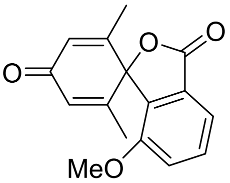

^{1}H NMR (400 MHz, CDCl$_3$) δ = 7.64 – 7.55 (m, 2H), 7.13 (d, *J* = 8.9 Hz, 1H), 6.28 (s, 2H), 3.77 (s, 3H), 1.66 (s, 6H).

^{13}C NMR (101 MHz, CDCl$_3$) δ = 185.3, 169.9, 154.3, 152.0, 134.4, 132.5, 128.9, 128.4, 117.6, 116.4, 83.5, 55.9, 17.2.

IR (cm-1) ν 2970, 1738, 1555, 1490, 1435, 1301, 1284, 1046, 970, 939, 770, 757

HRMS (ESI) calcd. for $C_{16}H_{14}NaO_{34}$ [M+Na] *m/z* 293.0784 found *m/z* 293.0783.

2,6-dimethyl-5'*H*-spiro[cyclohexane-1,7'-furo[3,4-b]pyridine]-2,5-diene-4,5'-dione (9)

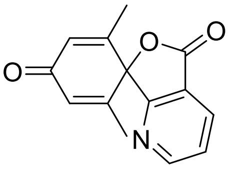

^{1}H NMR (400 MHz, CDCl$_3$) δ = 8.92 (d, *J* = 6.4 Hz, 1H), 8.33 (d, *J* = 7.8 Hz, 1H), 7.60 (dd, *J* = 7.8, 4.8 Hz, 1H), 6.37 (s, 2H), 1.66 (s, 6H).

^{13}C NMR (101 MHz, CDCl$_3$) δ = 184.7, 167.8, 166.9, 156.5, 151.5, 134.7, 129.8, 124.9, 120.5, 86.0, 17.2.

HRMS (ESI) calcd. for $C_{14}H_{11}NaO_3$ [M+Na] *m/z* 264.0631 found *m/z* 264.0635.

2,3,5,6-tetramethyl-3'*H*-spiro[cyclohexane-1,1'-isobenzofuran]-2,5-diene-3',4-dione (10)

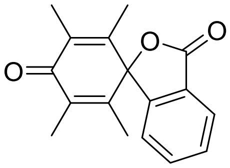

^{1}H NMR (400 MHz, CDCl$_3$) δ = 7.99 (d, *J* = 7.5 Hz, 1H), 7.67 (t, *J* = 7.5 Hz, 1H), 7.60 (t, *J* = 7.5 Hz, 1H), 7.10 (d, *J* = 7.6 Hz, 1H), 1.99 (s, 6H), 1.56 (s, 6H).

^{13}C NMR (101 MHz, CDCl$_3$) δ = 184.6, 170.5, 149.2, 146.2, 135.0, 133.1, 130.0, 126.9, 126.0, 121.4, 85.7, 14.0, 11.9.

IR (cm-1) ν 2978, 2960, 1652, 1626, 1482, 1270, 1212, 1041, 760, 730

HRMS (ESI) calcd. for $C_{17}H_{16}NaO_3$ [M+Na] *m/z* 291.0992 found *m/z* 291.0984.

2,3,5,6-tetramethyl-5'-nitro-3'*H*-spiro[cyclohexane-1,1'-isobenzofuran]-2,5-diene-3',4-dione (11)

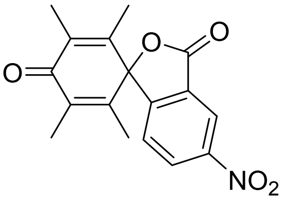

^{1}H NMR (400 MHz, $CDCl_3$) δ = 8.82 (s, 1H), 8.53 (d, *J* = 10.5 Hz, 1H), 7.32 (d, *J* = 8.4 Hz, 1H), 2.02 (s, 6H), 1.60 (s, 6H).

^{13}C NMR (101 MHz, $CDCl_3$) δ = 184.0, 167.8, 154.6, 149.6, 144.3, 134.3, 129.9, 128.6, 122.9, 121.6, 85.9, 14.0, 12.0.

IR (cm-1) ν 1769, 1731, 1687, 1652, 1625, 1543, 1481, 1311, 1270, 1247, 1152, 1112, 1099, 1070, 1041, 988, 966, 923, 843, 806, 791, 760, 701, 668

HRMS (ESI) calcd. for $C_{17}H_{15}NNaO_5$ [M+Na] *m/z* 336.0842 found *m/z* 336.0845.

3'*H*,10*H*-spiro[anthracene-9,1'-isobenzofuran]-3',10-dione (12)[7]

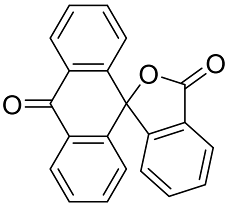

^{1}H NMR (400 MHz, $CDCl_3$) δ = 8.43 – 8.38 (m, 2H), 8.05 (ddd, *J* = 7.4, 3.4, 2.1 Hz, 1H), 7.62 – 7.55 (m, 4H), 7.55 – 7.49 (m, 2H), 7.27 – 7.21 (m, 2H), 6.91 (ddd, *J* = 7.9, 3.4, 2.2 Hz, 1H).

^{13}C NMR (101 MHz, $CDCl_3$) δ = 182.8, 170.6, 154.0, 139.7, 135.4, 134.1, 130.5, 129.6, 129.4, 127.9, 126.2, 125.9, 123.7, 122.4, 83.1.

HRMS (ESI) calcd. for $C_{21}H_{12}NaO_3$ [M+Na] *m/z* 335.0679 found *m/z* 335.0678.

7'-methoxy-3'*H*,10*H*-spiro[anthracene-9,1'-isobenzofuran]-3',10-dione (13)

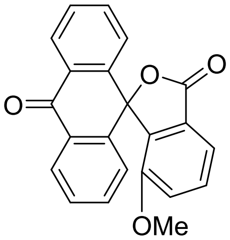

^{1}H NMR (400 MHz, $CDCl_3$) δ = 8.36 (d, *J* = 9.0 Hz, 2H), 7.65 (d, *J* = 7.6 Hz, 1H), 7.56 (dd, *J* = 13.9, 10.2 Hz, 5H), 7.31 (s, 2H), 6.94 (d, *J* = 8.0 Hz, 1H), 3.46 (s, 3H).

^{13}C NMR (101 MHz, $CDCl_3$) δ = 183.3, 170.8, 153.3, 141.2, 138.4, 133.3, 131.9, 131.5, 129.1, 127.1, 125.4, 117.4, 116.6, 82.1, 55.4.

IR (cm-1) ν 2978, 1731, 1723, 1686, 1652, 1624, 1483, 1270, 1112, 1098, 1041, 760

HRMS (ESI) calcd. for $C_{22}H_{14}NaO_4$ [M+Na] *m/z* 365.0784 found *m/z* 365.0786.

3'*H*,10*H*-spiro[anthracene-9,1'-naphtho[1,2-c]furan]-3',10-dione (14)

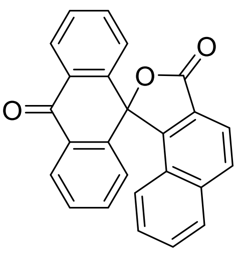

^{1}H NMR (400 MHz, $CDCl_3$) δ = 8.51 (d, *J* = 7.4 Hz, 2H), 8.11 (dd, *J* = 18.8, 8.5 Hz, 2H), 7.99 (d, *J* = 8.3 Hz, 1H), 7.63 – 7.52 (m, 3H), 7.49 (t, *J* = 7.6 Hz, 2H), 7.29 (t, *J* = 7.1 Hz, 1H), 7.16 (d, *J* = 8.1 Hz, 1H), 6.98 (d, *J* = 7.9 Hz, 2H).

^{13}C NMR (101 MHz, $CDCl_3$) δ = 182.4, 170.5, 150.9, 138.8, 137.0, 134.3, 131.9, 130.9, 129.9, 129.4, 129.2, 128.3, 128.0, 127.3, 125.8, 123.9, 123.8, 120.1, 82.7.

IR (cm-1) ν 1714, 1668, 1627, 1597, 1589, 1520, 1460, 1320, 1304, 1275, 1173, 1096, 977, 928, 786, 756, 715, 680

HRMS (ESI) calcd. for $C_{25}H_{14}NaO_3$ [M+Na] *m/z* 385.0835 found *m/z* 385.0836.

2,6-dimethoxy-3'*H*-spiro[cyclohexane-1,1'-isobenzofuran]-2,5-diene-3',4-dione (15)

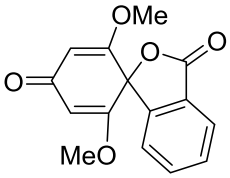

^{1}H NMR (400 MHz, $CDCl_3$) δ = 7.97 (d, *J* = 7.4 Hz, 1H), 7.70 (t, *J* = 7.5 Hz, 1H), 7.63 (t, *J* = 7.0 Hz, 1H), 7.28 (d, *J* = 7.6 Hz, 1H), 5.64 (s, 2H), 3.61 (s, 6H).

^{13}C NMR (101 MHz, $CDCl_3$) δ = 186.2, 169.6, 165.9, 146.8, 134.8, 130.4, 126.4, 126.1, 120.8, 102.2, 80.7, 56.6.

IR (cm^{-1}) ν 3005, 1710, 1666, 1567, 1504, 1417, 1359, 1275, 1261, 1084, 961, 870, 750, 764

HRMS (ESI) calcd. for $C_{15}H_{12}NaO_5$ [M+Na] *m/z* 295.0577 found *m/z* 295.0575.

2,6-dimethoxy-5'-methyl-3'*H*-spiro[cyclohexane-1,1'-isobenzofuran]-2,5-diene-3',4-dione (16)

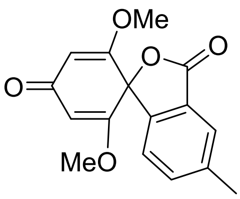

^{1}H NMR (400 MHz, $CDCl_3$) δ 7.77 (s, 1H), 7.50 (d, *J* = 7.9 Hz, 1H), 7.16 (d, *J* = 7.8 Hz, 1H), 5.63 (s, 2H), 3.62 (d, *J* = 2.4 Hz, 6H), 2.51 (s, 3H).

^{13}C NMR (101 MHz, $CDCl_3$) δ 186.24, 169.74, 166.14, 144.22, 140.90, 135.82, 126.61, 126.16, 120.53, 102.11, 80.58, 56.53, 21.33.

IR (cm^{-1}) ν 2990, 2970, 2707, 1738, 1314, 1564, 1500, 1364, 1275, 1261, 1092, 897, 764, 750

HRMS (ESI) calcd. for $C_{16}H_{14}NaO_5$ [M+Na] *m/z* 309.0733 found *m/z* 309.0748.

2,6,7'-trimethoxy-3'*H*-spiro[cyclohexane-1,1'-isobenzofuran]-2,5-diene-3',4-dione (17)

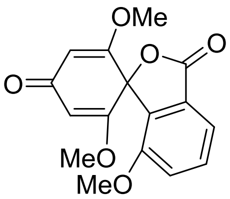

^{1}H NMR (400 MHz, $CDCl_3$) δ = 7.61 (t, *J* = 7.7 Hz, 1H), 7.56 (d, *J* = 7.0 Hz, 1H), 7.15 (d, *J* = 7.9 Hz, 1H), 5.67 (s, 2H), 3.83 (s, 3H), 3.65 (s, 6H).

^{13}C NMR (101 MHz, $CDCl_3$) δ = 186.7, 169.6, 164.8, 154.0, 133.6, 128.8, 117.4, 115.9, 102.6, 79.6, 56.5, 55.9.

HRMS (ESI) calcd. for $C_{16}H_{14}NaO_6$ [M+Na] *m/z* 325.0683 found *m/z* 325.0691.

3*H*,4'*H*-spiro[isobenzofuran-1,1'-naphthalene]-3,4'-dione (18)

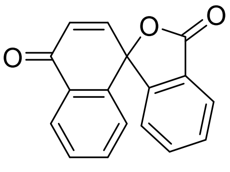

^{1}H NMR (400 MHz, $CDCl_3$) δ = 8.23 (dd, *J* = 6.2, 3.1 Hz, 1H), 8.05 (d, *J* = 6.1 Hz, 1H), 7.67 – 7.59 (m, 2H), 7.53 (dd, *J* = 5.9, 3.4 Hz, 2H), 7.10 (d, *J* = 6.5 Hz, 1H), 7.05 – 7.01 (m, 1H), 6.72 (dd, *J* = 68.3, 10.1 Hz, 2H).

^{13}C NMR (101 MHz, $CDCl_3$) δ = 183.6, 169.8, 150.2, 144.7, 138.9, 135.3, 133.6, 130.2, 130.1, 130.0, 129.5, 127.1, 126.5, 126.5, 125.0, 122.6, 81.6.

IR (cm^{-1}) ν 2970, 2948, 2707, 1738, 1673, 1599, 1500, 1456, 1276, 1261, 974, 940, 764, 750

HRMS (ESI) calcd. for $C_{17}H_{10}NaO_3$ [M+Na] *m/z* 285.0522 found *m/z* 285.0533.

7-methyl-3*H*,4'*H*-spiro[isobenzofuran-1,1'-naphthalene]-3,4'-dione (19)

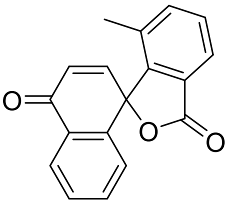

^{1}H NMR (400 MHz, $CDCl_3$) δ = 8.27 – 8.21 (m, 1H), 7.92 (d, *J* = 7.6 Hz, 1H), 7.60 – 7.49 (m, 3H), 7.42 (d, *J* = 7.5 Hz, 1H), 6.95 (dd, *J* = 5.7, 3.4 Hz, 1H), 6.76 – 6.65 (m, 2H), 1.87 (s, 3H).

^{13}C NMR (101 MHz, $CDCl_3$) δ = 183.5, 169.8, 147.4, 142.7, 137.6, 136.9, 133.8, 133.6, 131.1, 131.0, 130.8, 129.6, 127.0, 126.9, 125.8, 124.0, 80.9, 16.5.

IR (cm^{-1}) ν 2978, 1653, 1625, 1482, 1270, 1095, 1070, 1041, 988, 966, 921, 760, 731, 701

HRMS (ESI) calcd. for $C_{18}H_{12}NaO_3$ [M+Na] *m/z* 299.0679 found *m/z* 299.0682.

7-methoxy-3*H*,4'*H*-spiro[isobenzofuran-1,1'-naphthalene]-3,4'-dione (20)

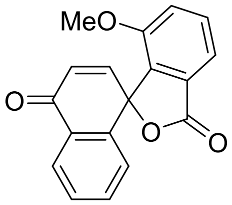

^{1}H NMR (400 MHz, CDCl$_3$) δ = 8.19 (dd, *J* = 5.9, 3.3 Hz, 1H), 7.59 (dt, *J* = 15.3, 7.6 Hz, 2H), 7.54 – 7.44 (m, 2H), 7.05 (t, *J* = 7.2 Hz, 2H), 6.65 (dd, *J* = 32.9, 10.1 Hz, 2H), 3.60 (s, 3H).

^{13}C NMR (101 MHz, CDCl$_3$) δ = 184.1, 169.9, 154.01, 142.8, 137.9, 136.9, 132.9, 132.4, 131.0, 130.5, 129.1, 126.9, 126.6, 125.8, 117.7, 116.5, 80.4, 55.7.

IR (cm^{-1}) ν 2978, 1731, 1686, 1652, 1625, 1485, 1265, 1114, 1099, 925, 760

HRMS (ESI) calcd. for $C_{18}H_{12}NaO_4$ [M+Na] *m/z* 315.0628 found *m/z* 315.0640.

3'*H*,4*H*-spiro[naphthalene-1,1'-naphtho[1,2-c]furan]-3',4-dione (21)

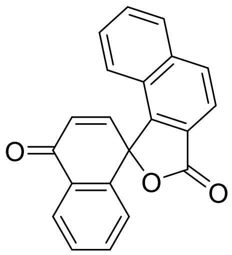

^{1}H NMR (400 MHz, CDCl$_3$) δ = 8.33 (d, *J* = 6.3 Hz, 1H), 8.12 (d, *J* = 8.4 Hz, 1H), 8.03 (d, *J* = 8.4 Hz, 2H), 7.64 (t, *J* = 7.6 Hz, 1H), 7.56 (d, *J* = 7.4 Hz, 1H), 7.43 (d, *J* = 8.1 Hz, 2H), 7.38 (d, *J* = 7.3 Hz, 1H), 6.95 (d, *J* = 8.6 Hz, 1H), 6.79 (q, *J* = 10.1 Hz, 2H).

^{13}C NMR (101 MHz, CDCl$_3$) δ = 183.6, 169.9, 148.0, 144.2, 138.4, 136.8, 133.8, 132.2, 131.1, 130.6, 129.8, 129.5, 129.4, 128.5, 127.4, 127.1, 126.1, 123.9, 123.2, 120.6, 80.9.

IR (cm^{-1}) ν 2978, 2960, 1731, 1687, 1653, 1625, 1484, 1303, 1270, 1099, 1041, 925, 760, 729, 713

HRMS (ESI) calcd. for $C_{21}H_{12}NaO_3$ [M+Na] *m/z* 335.0679 found *m/z* 335.0681.

7'-methoxy-2-methyl-3'*H*-spiro[cyclohexane-1,1'-isobenzofuran]-2,5-diene-3',4-dione (22)

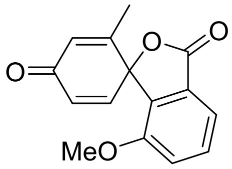

^{1}H NMR (400 MHz, CDCl$_3$) δ = 7.65 – 7.55 (m, 2H), 7.14 (d, *J* = 7.5 Hz, 1H), 6.52 (d, *J* = 9.9 Hz, 1H), 6.40 (d, *J* = 11.6 Hz, 1H), 6.31 (s, 1H), 3.78 (s, 3H), 1.67 (d, *J* = 1.4 Hz, 3H).

^{13}C NMR (101 MHz, CDCl$_3$) δ = 185.4, 169.4, 154.5, 151.9, 142.6, 133.8, 132.6, 130.2, 128.9, 128.0, 117.8, 116.3, 81.2, 55.8, 17.2.

IR (cm^{-1}) ν 2978, 1731, 1684, 1485, 1265, 1246, 1114, 1098, 1039, 925, 760

HRMS (ESI) calcd. for $C_{15}H_{12}NaO_4$ [M+Na] *m/z* 279.0628 found *m/z* 279.0634.

2-isopropyl-7'-methoxy-3'*H*-spiro[cyclohexane-1,1'-isobenzofuran]-2,5-diene-3',4-dione (23)

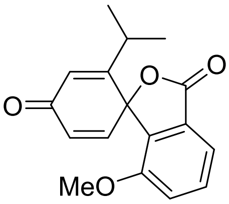

^{1}H NMR (400 MHz, $CDCl_3$) δ = 7.65 – 7.56 (m, 2H), 7.13 (d, *J* = 8.8 Hz, 1H), 6.48 (d, *J* = 9.8 Hz, 1H), 6.43 – 6.34 (m, 2H), 3.78 (s, 1H), 1.97 – 1.84 (m, 1H), 1.16 (d, *J* = 6.8 Hz, 3H), 0.77 (d, *J* = 6.8 Hz, 3H).

^{13}C NMR (101 MHz, $CDCl_3$) δ = 185.9, 169.8, 162.2, 142.9, 133.4, 132.7, 129.7, 128.3, 126.7, 117.9, 116.2, 81.4, 55.7, 28.2, 23.5, 23.0.

HRMS (ESI) calcd. for $C_{17}H_{16}NaO_4$ [M+Na] *m/z* 307.0941 found *m/z* 307.0943.

Methyl 2',6'-dimethyl-[1,1'-biphenyl]-2-carboxylate (24)[8]

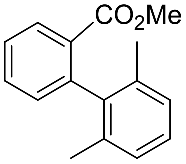

^{1}H NMR (400 MHz, $CDCl_3$) δ = 8.04 (ddd, *J* = 7.9, 1.5, 0.5 Hz, 1H), 7.60 (td, *J* = 7.5, 1.5 Hz, 1H), 7.46 (td, *J* = 7.6, 1.3 Hz, 1H), 7.21 – 7.15 (m, 2H), 7.11 (dq, *J* = 7.1, 0.7 Hz, 2H), 3.64 (s, 3H), 1.97 (s, 6H).

^{13}C NMR (101 MHz, $CDCl_3$) δ = 167.4, 142.2, 141.0, 135.2, 132.1, 130.6, 130.3, 130.2, 127.0, 126.9, 126.9, 51.8, 20.54.

4-(methoxyimino)-2,6-dimethyl-3'H-spiro[cyclohexane-1,1'-isobenzofuran]-2,5-dien-3'-one

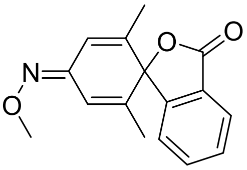

^{1}H NMR (400 MHz, $CDCl_3$) δ 7.94 (d, *J* = 7.6 Hz, 1H), 7.67 (t, *J* = 7.4 Hz, 1H), 7.57 (t, *J* = 7.5 Hz, 1H), 7.18 (d, *J* = 7.6 Hz, 1H), 6.93 (s, 1H), 6.30 (s, 1H), 3.99 (s, 3H), 1.56 (s, 3H), 1.52 (s, 3H).

^{13}C NMR (101 MHz, CDCl3) δ 170.3, 149.8, 147.7, 143.1, 138.6, 134.9, 129.9, 126.3, 125.58, 123.2, 121.8, 115.5, 87.0, 62.3, 17.3, 16.8.

4a',6'-dimethyl-1',4',4a',8a'-tetrahydro-3H,8'H-spiro[isobenzofuran-1,5'-[1,4]ethanonaphthalene]-3,8'-dione

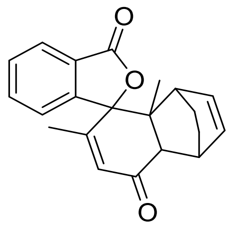

^{1}H NMR (400 MHz, $CDCl_3$) δ 7.78 (d, *J* = 7.6 Hz, 1H), 7.53 (t, *J* = 7.6 Hz, 1H), 7.43 (t, *J* = 7.5 Hz, 1H), 7.04 (d, *J* = 7.8 Hz, 1H), 6.52 (t, *J* = 7.2 Hz, 1H), 5.92 (t, *J* = 7.2 Hz, 1H), 5.84 (s, 1H), 3.03 – 2.95 (m, 1H), 2.92 (s, 1H), 2.53 (d, *J* = 2.7 Hz, 1H), 1.80 (dd, *J* = 12.6, 9.8 Hz, 1H), 1.67 – 1.54 (m, 4H), 1.32 (dd, *J* = 17.2, 7.8 Hz, 1H), 1.06 – 0.98 (m, 1H), 0.67 (s, 3H).

^{13}C NMR (101 MHz, CDCl3) δ 199.5, 168.9, 159.1, 151.1, 135.3, 133.6, 131.2, 128.6, 128.3, 124.5, 124.0, 122.2, 90.5, 57.9, 41.6, 38.2, 36.2, 23.6, 21.9, 21.8, 17.3.

2,2'',6,6''-tetramethyl-3,3''-bispiro[cyclohexane-1,1'-isobenzofuran]-2,2'',5,5''-tetraene-3',3''',4,4''-tetraone

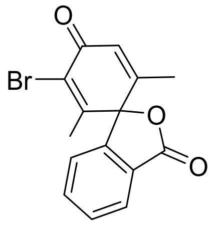

^{1}H NMR (400 MHz, $CDCl_3$) δ 8.03 (dt, *J* = 7.5, 1.0 Hz, 1H), 7.71 (m, 2H), 7.21 (dt, *J* = 7.6, 1.0 Hz, 1H), 6.42 (q, *J* = 1.4 Hz, 1H), 1.82 (s, 3H), 1.67 (d, *J* = 1.4 Hz, 3H).

^{13}C NMR (101 MHz, CDCl3) δ 177.4, 169.3, 154.6, 152.3, 147.2, 135.63, 130.9, 126.7, 126.6, 126.5, 126.1, 121.5, 85.9, 18.8, 17.1.

HRMS (ESI) calcd. for $C_{15}H_{12}BrO_3$ [M+H] *m/z* 319.9968 found *m/z* 319.9964.

18. Spectra of all starting acids

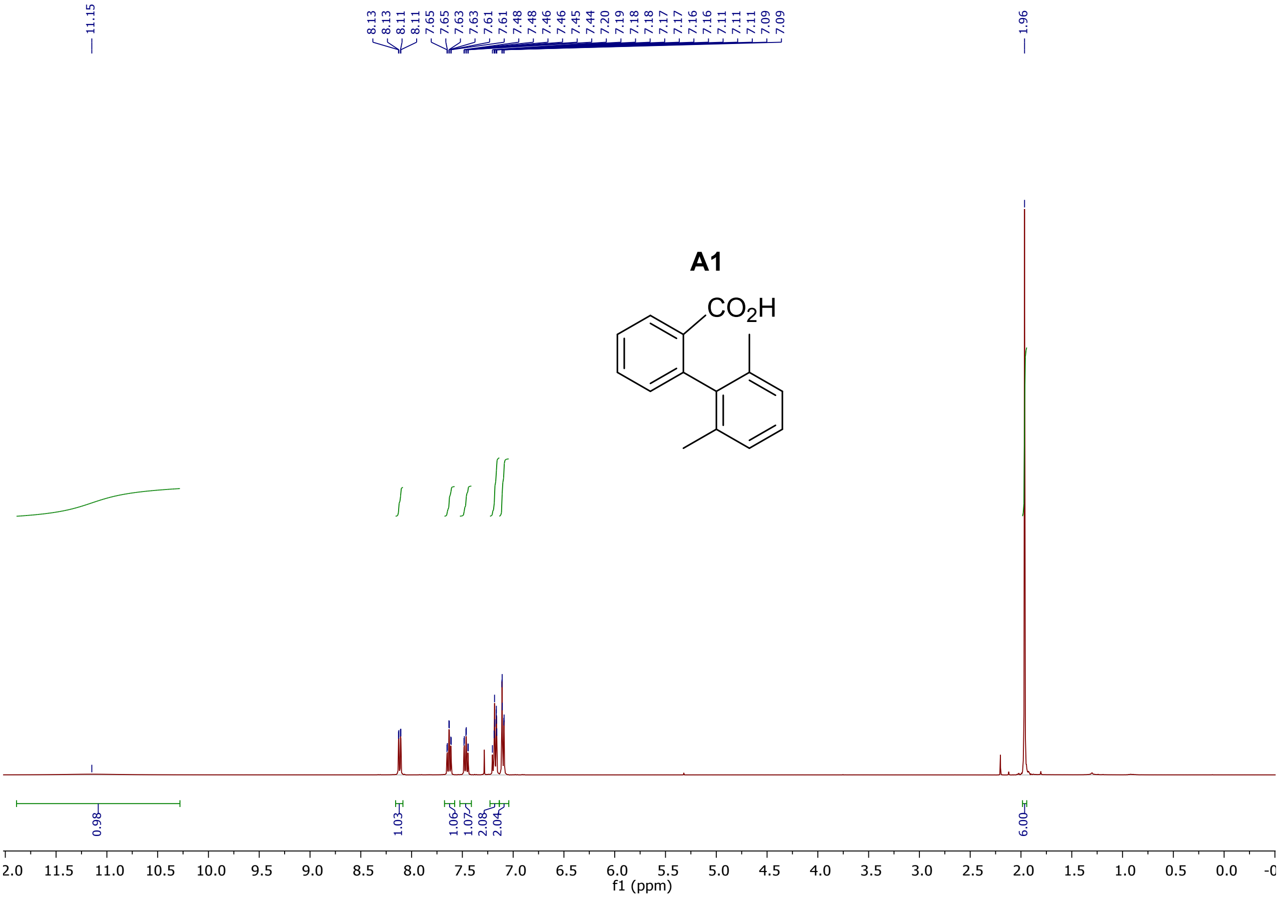

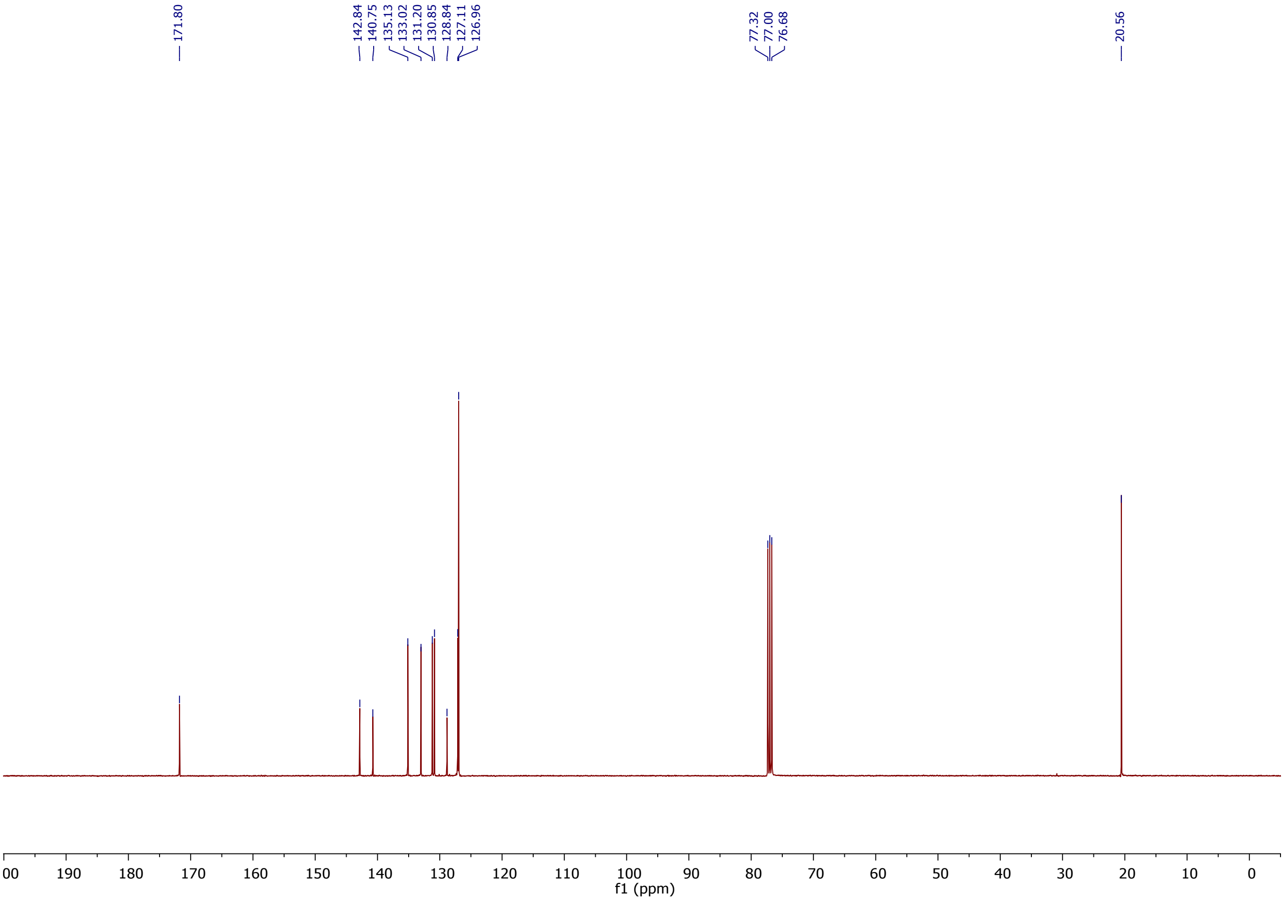

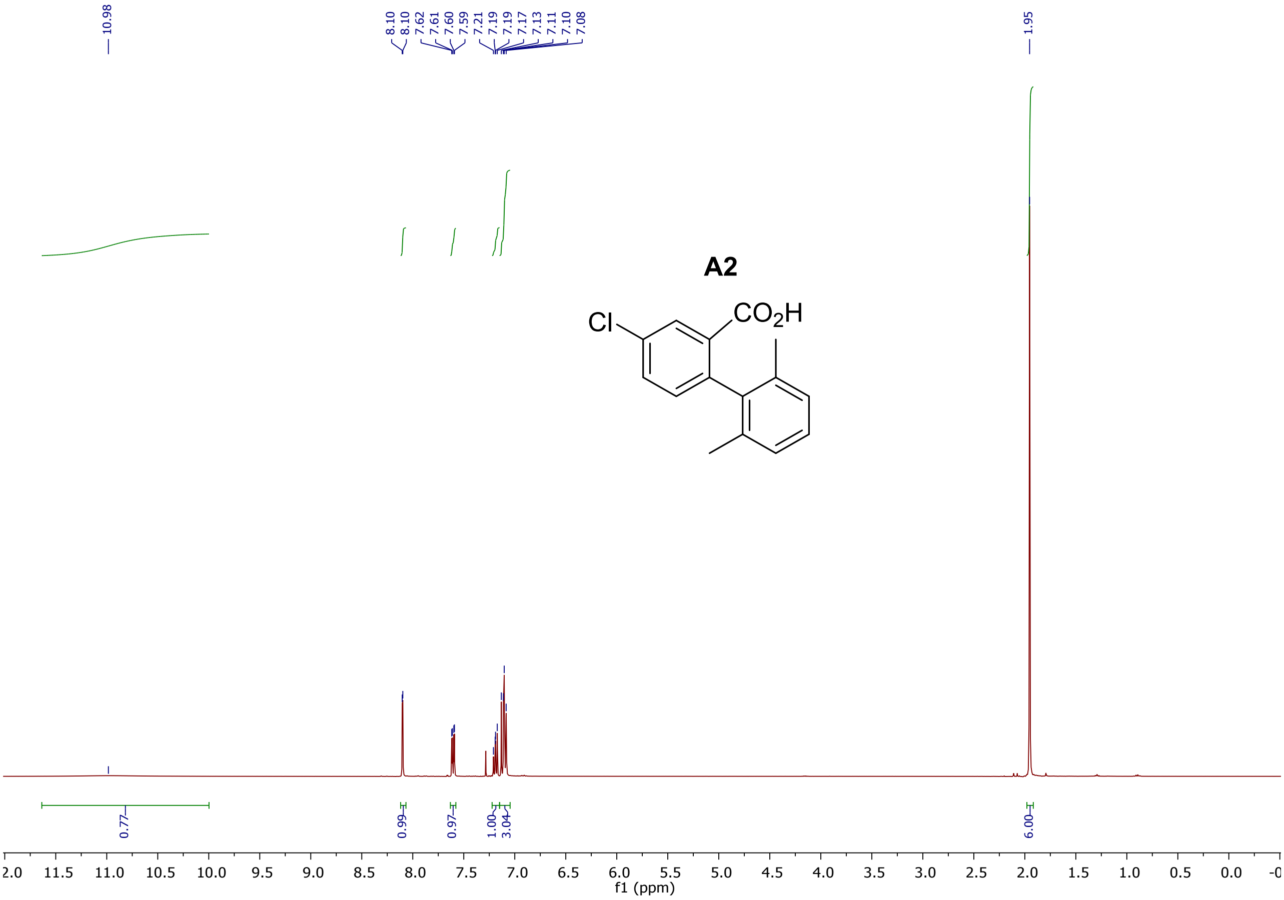
10.98
8.10
8.10
7.62
7.61
7.60
7.59
7.21
7.19
7.19
7.17
7.13
7.11
7.10
7.08
1.95
A2
Cl
CO2H
0.77
0.99
0.97
1.00
3.04
6.00
f1 (ppm)

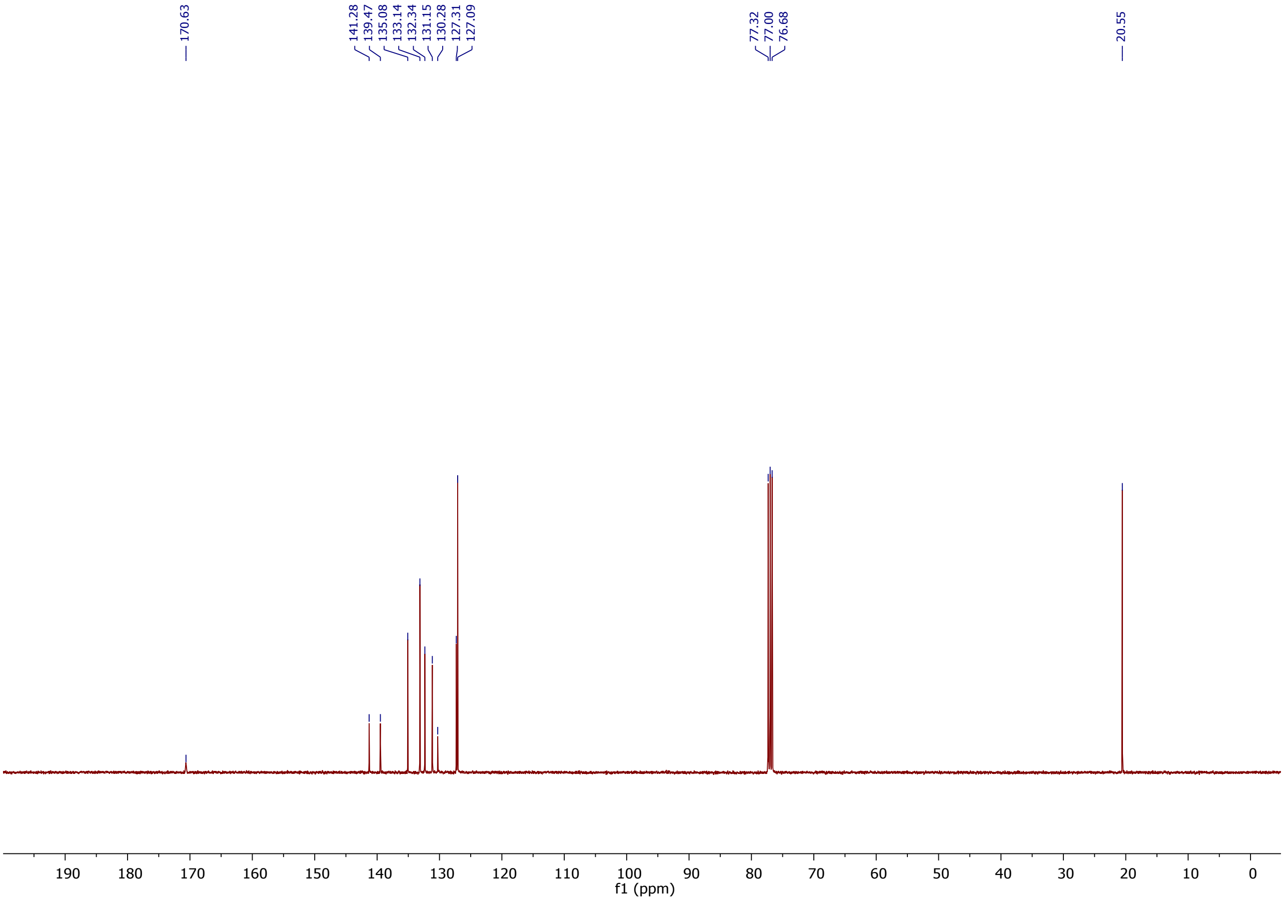
170.63
141.28
139.47
135.08
133.14
132.34
131.15
130.28
127.31
127.09
77.32
77.00
76.68
20.55
f1 (ppm)

A3

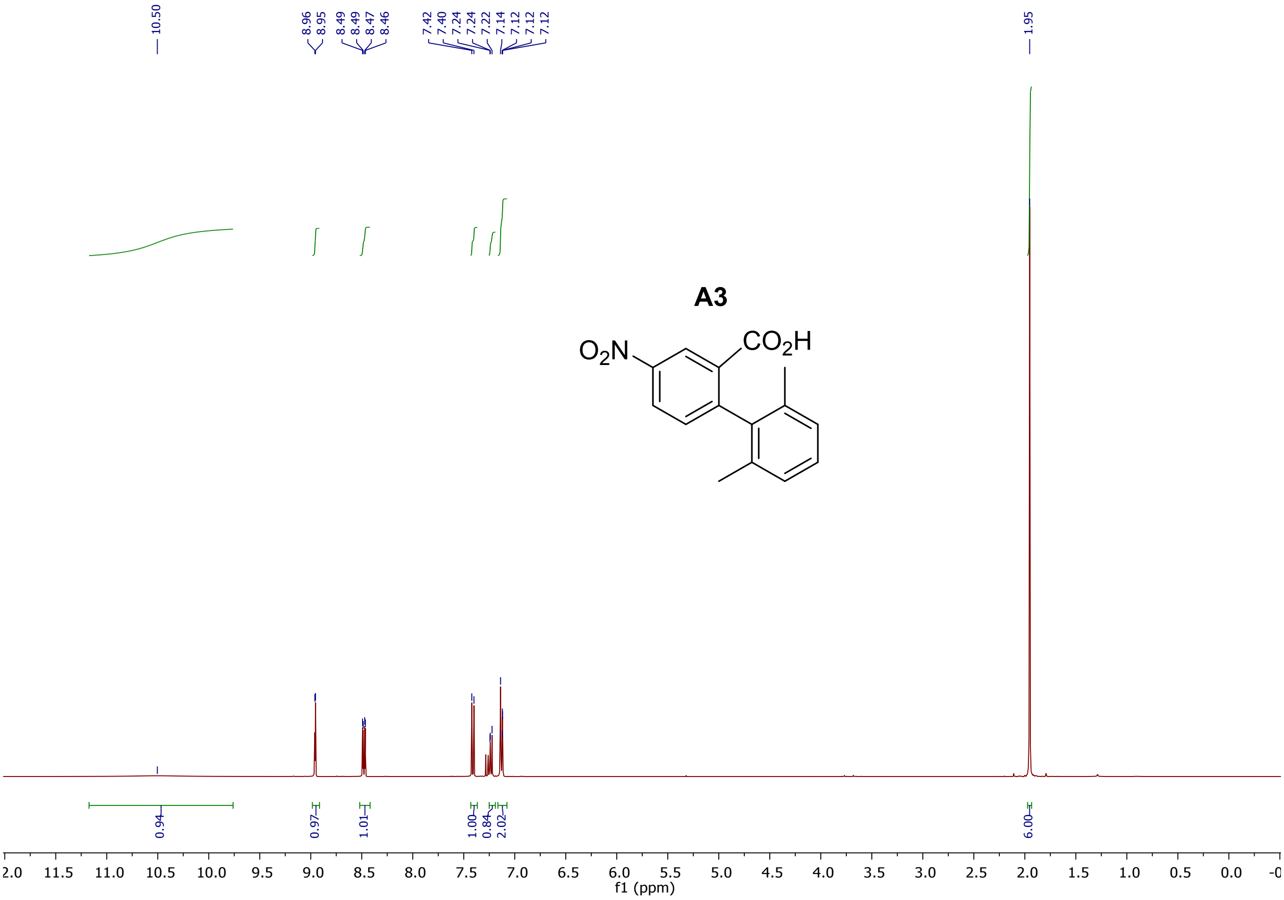
O2N
CO2H
10.50
8.96
8.95
8.49
8.49
8.47
8.46
7.42
7.40
7.24
7.24
7.22
7.14
7.12
7.12
7.12
1.95
0.94
0.97
1.01
1.00
0.84
2.02
6.00
f1 (ppm)

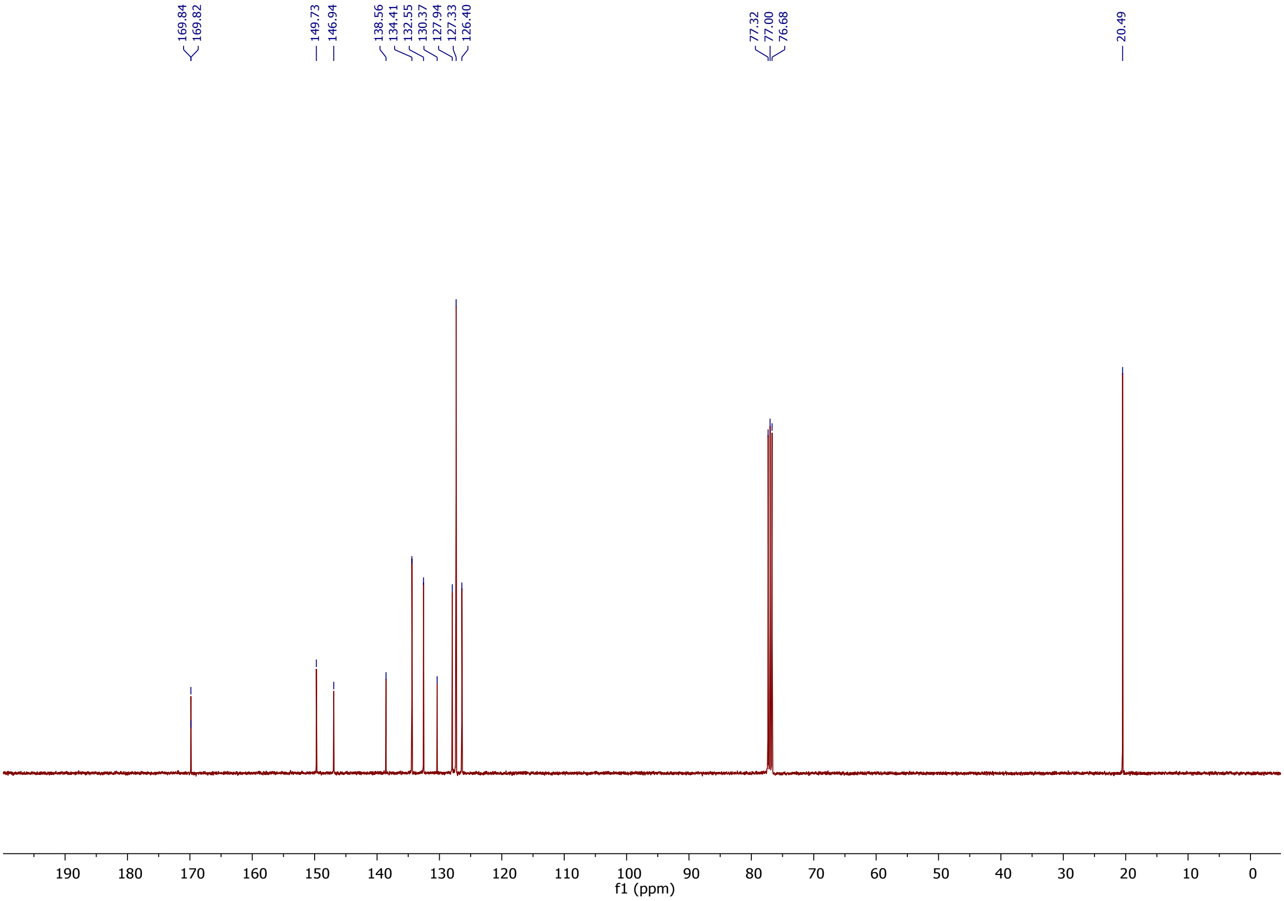
169.84
169.82
149.73
146.94
138.56
134.41
132.55
130.37
127.94
127.33
126.40
77.32
77.00
76.68
20.49
f1 (ppm)

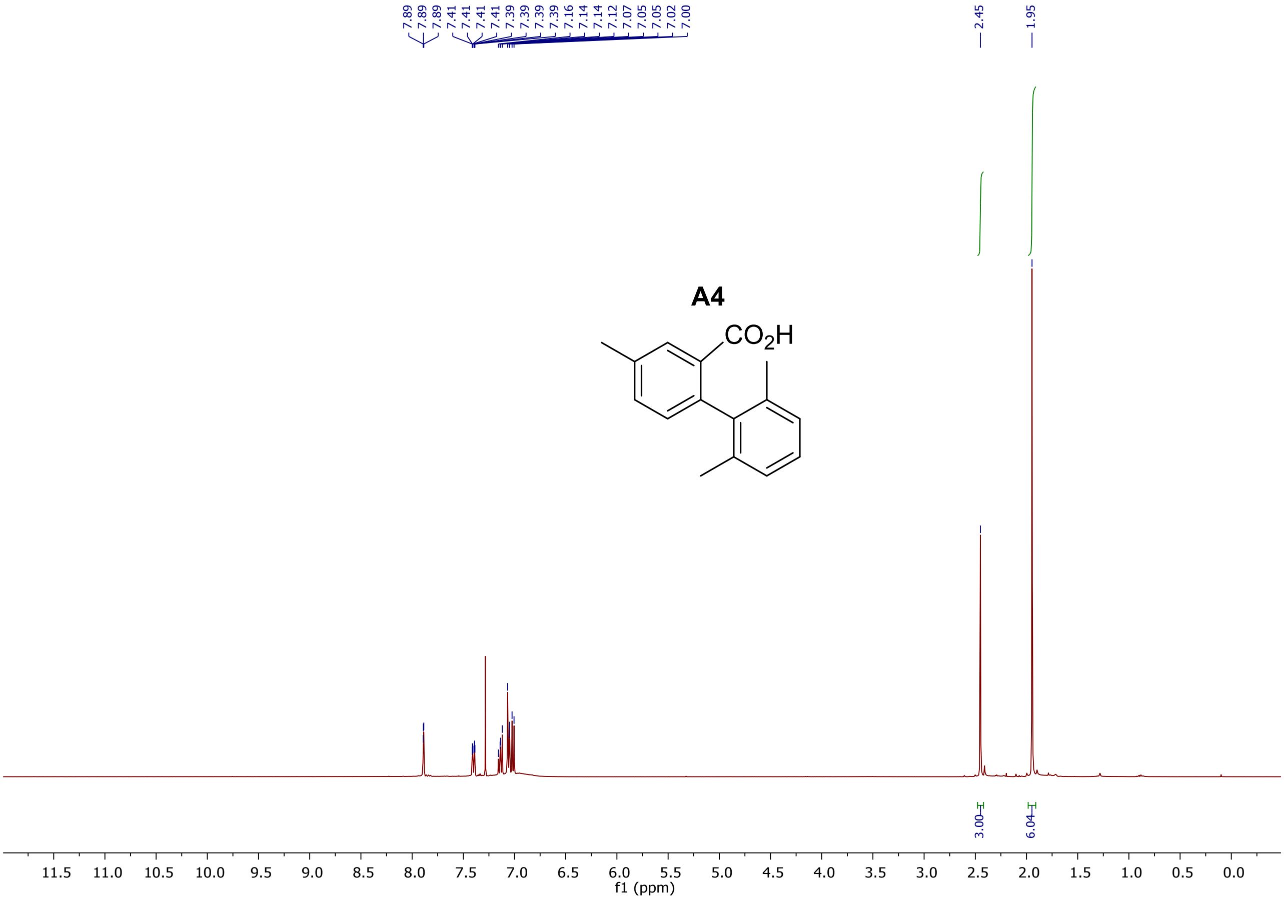
7.89
7.89
7.89
7.41
7.41
7.41
7.41
7.39
7.39
7.39
7.39
7.16
7.14
7.12
7.07
7.05
7.05
7.02
7.00
2.45
1.95
A4
CO_2H
3.00
6.04
11.5 11.0 10.5 10.0 9.5 9.0 8.5 8.0 7.5 7.0 6.5 6.0 5.5 5.0 4.5 4.0 3.5 3.0 2.5 2.0 1.5 1.0 0.5 0.0
f1 (ppm)

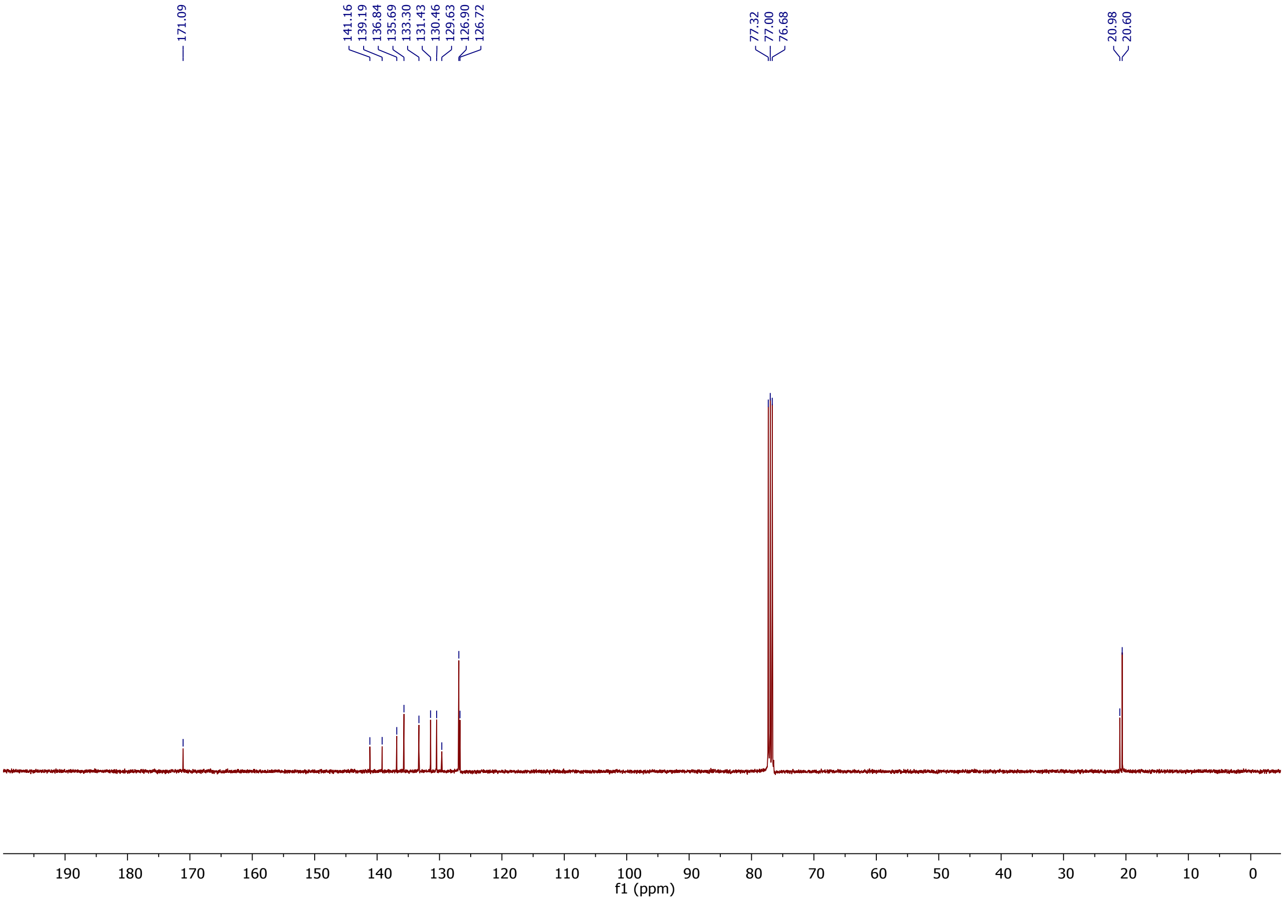
171.09
141.16
139.19
136.84
135.69
133.30
131.43
130.46
129.63
126.90
126.72
77.32
77.00
76.68
20.98
20.60
190 180 170 160 150 140 130 120 110 100 90 80 70 60 50 40 30 20 10 0
f1 (ppm)

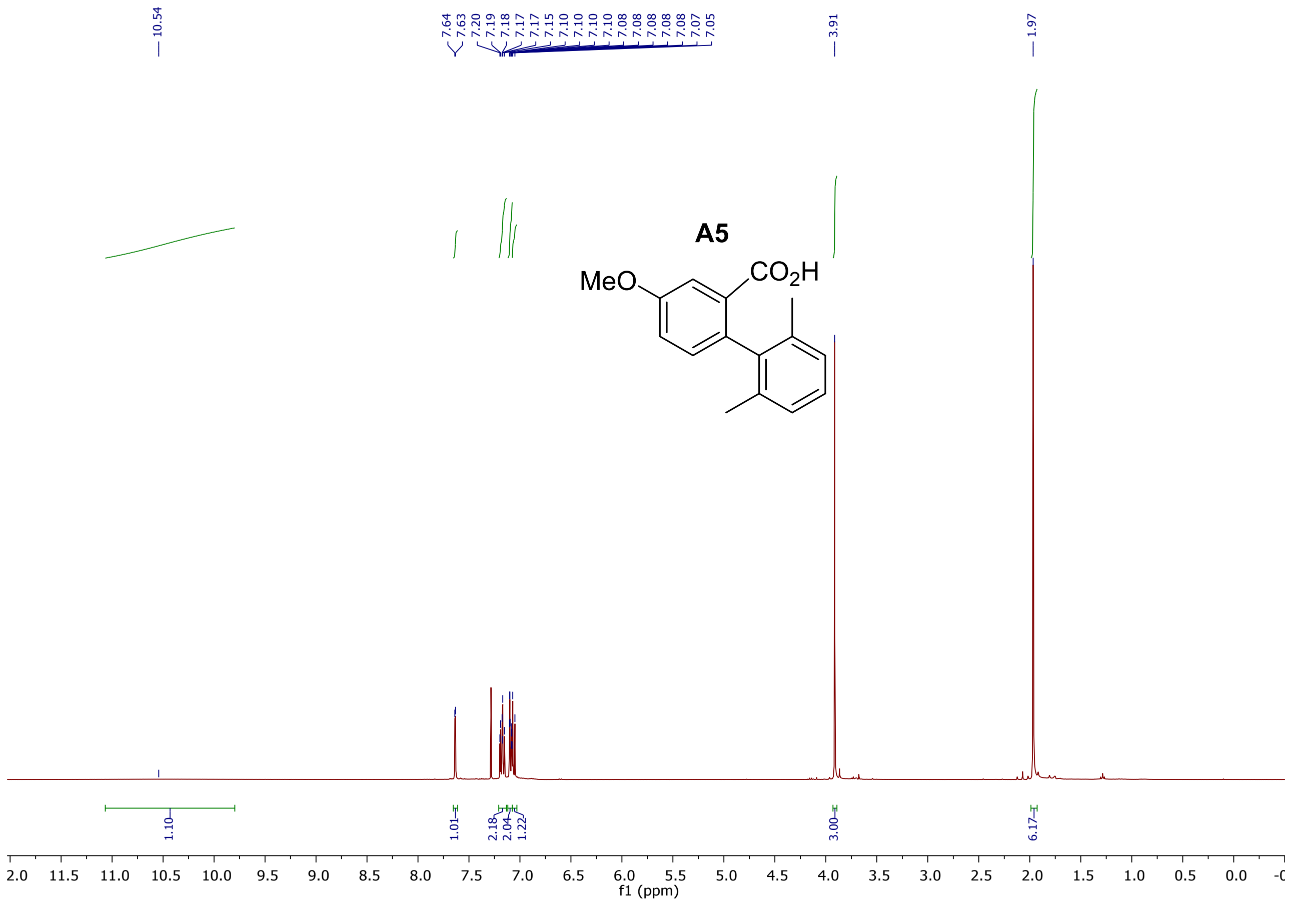
10.54
7.64 7.63 7.20 7.19 7.18 7.17 7.17 7.15 7.10 7.10 7.10 7.10 7.08 7.08 7.08 7.08 7.08 7.07 7.05
3.91
1.97
A5
MeO
CO2H
1.10
1.01
2.18
2.04
1.22
3.00
6.17
f1 (ppm)

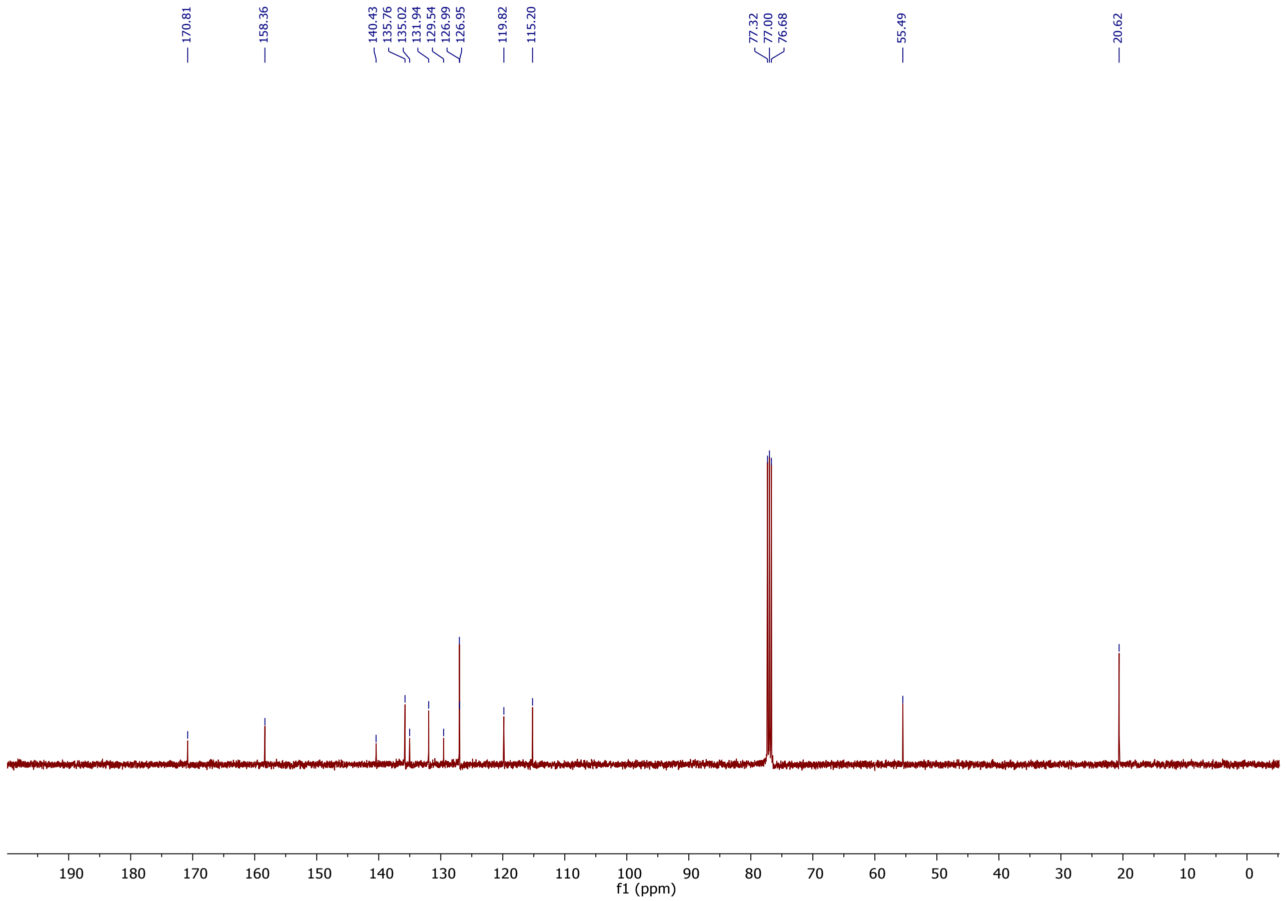
170.81
158.36
140.43 135.76 135.02 131.94 129.54 126.99 126.95
119.82
115.20
77.32 77.00 76.68
55.49
20.62
f1 (ppm)

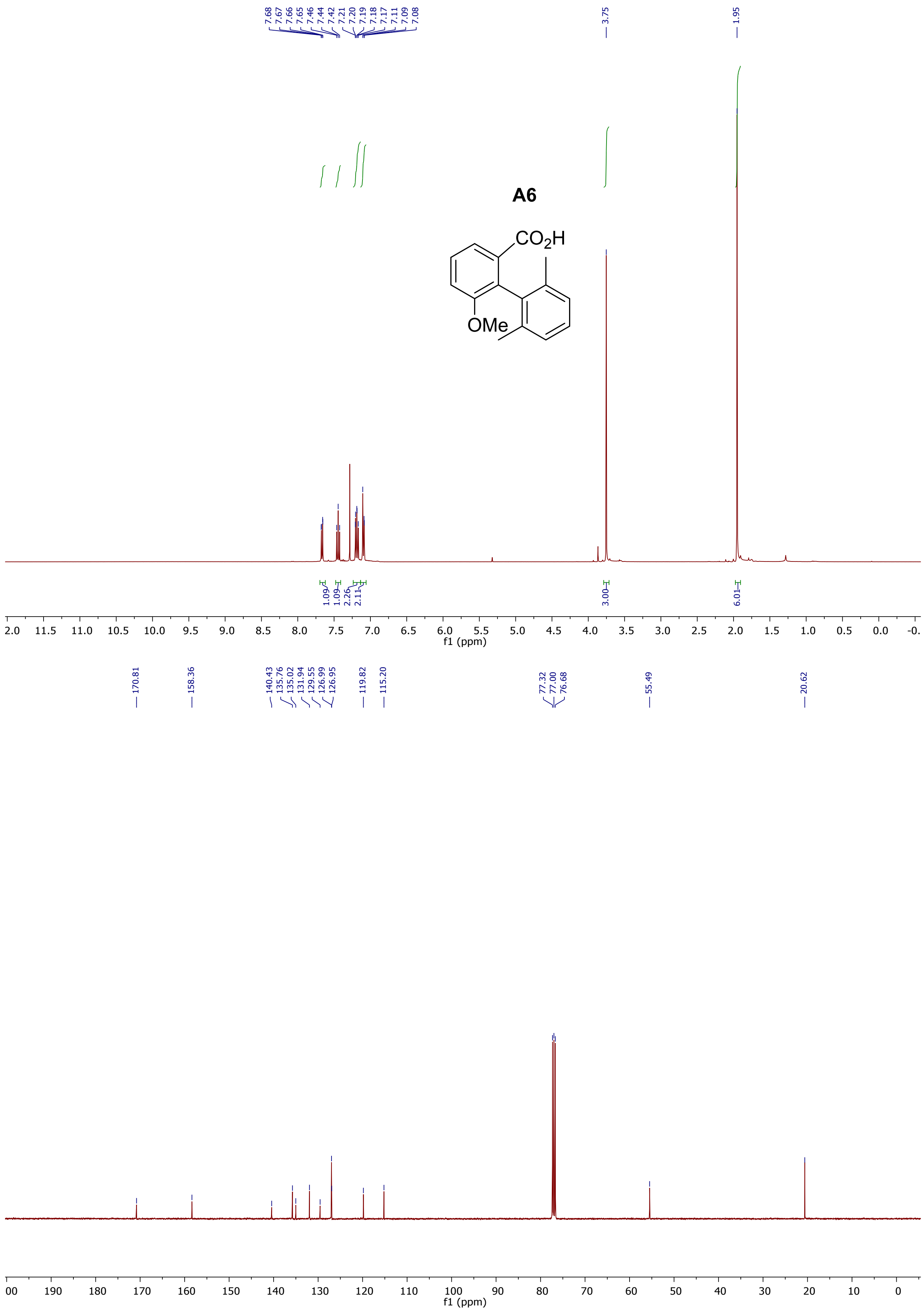
7.68
7.67
7.66
7.65
7.46
7.44
7.42
7.21
7.20
7.19
7.18
7.17
7.11
7.09
7.08
3.75
1.95
A6
CO2H
OMe
1.09
1.09
2.26
2.11
3.00
6.01
f1 (ppm)
170.81
158.36
140.43
135.76
135.02
131.94
129.55
126.99
126.95
119.82
115.20
77.32
77.00
76.68
55.49
20.62
f1 (ppm)

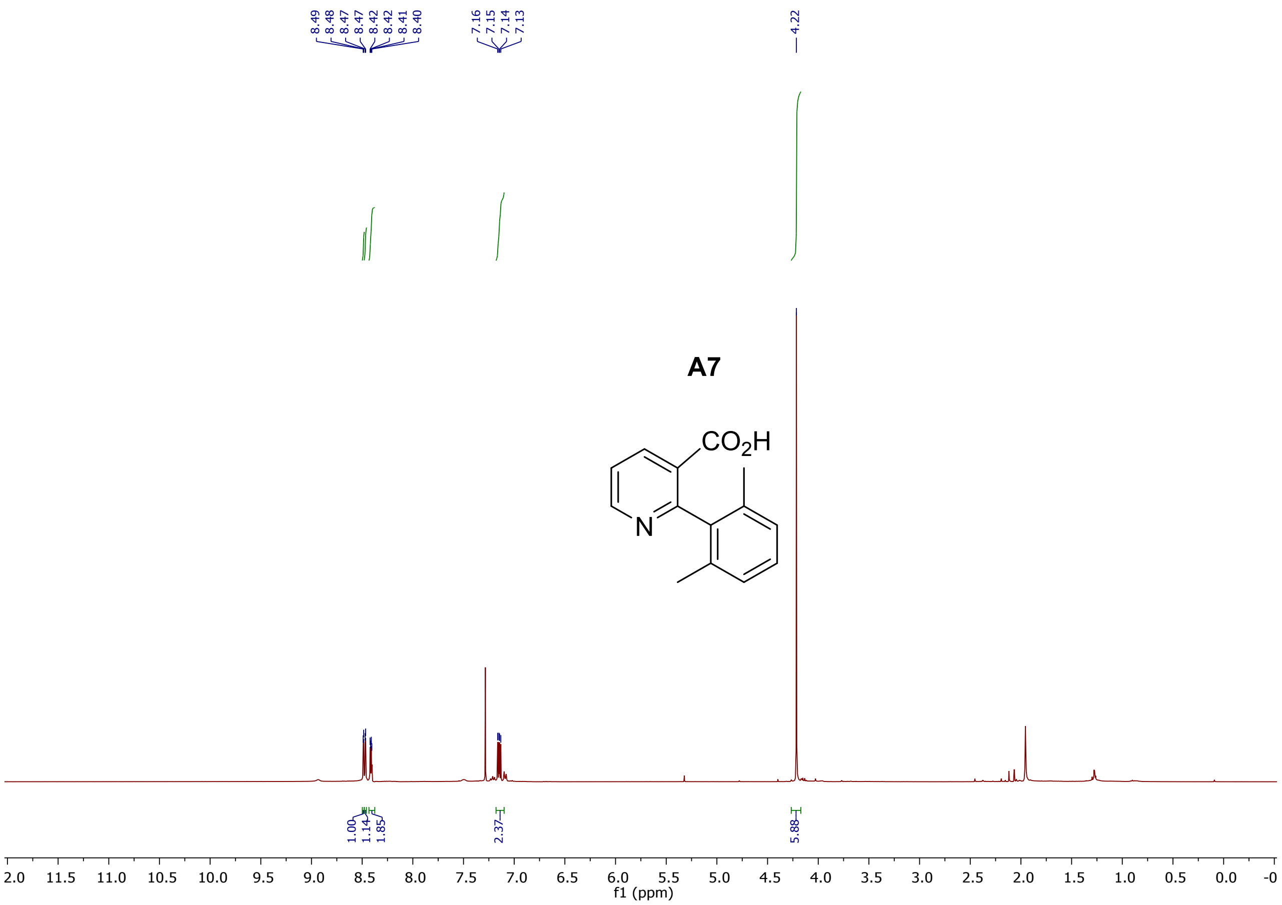
8.49
8.48
8.47
8.47
8.42
8.42
8.41
8.40
7.16
7.15
7.14
7.13
4.22
A7
CO2H
N
1.00
1.14
1.85
2.37
5.88
f1 (ppm)

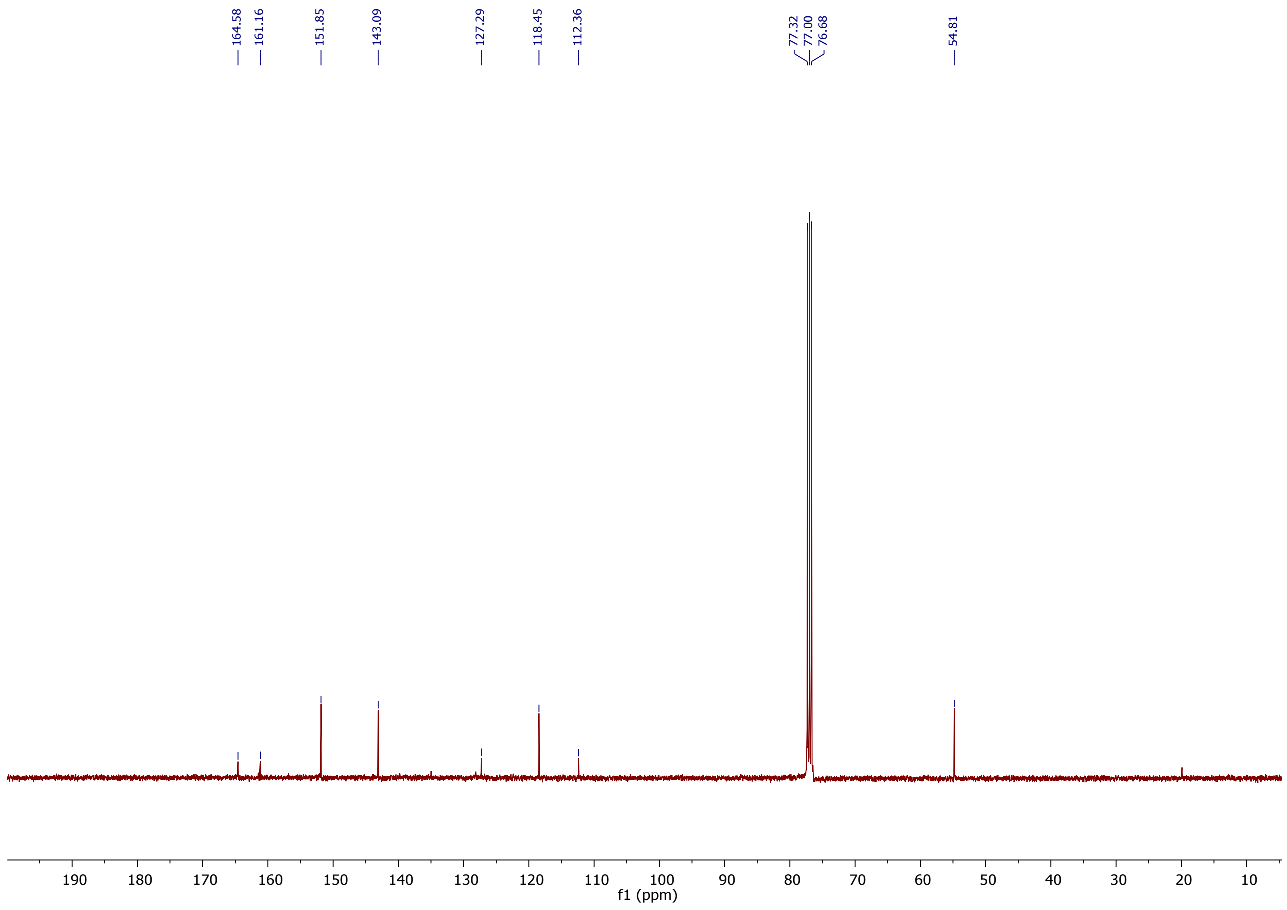
164.58
161.16
151.85
143.09
127.29
118.45
112.36
77.32
77.00
76.68
54.81
f1 (ppm)

A8

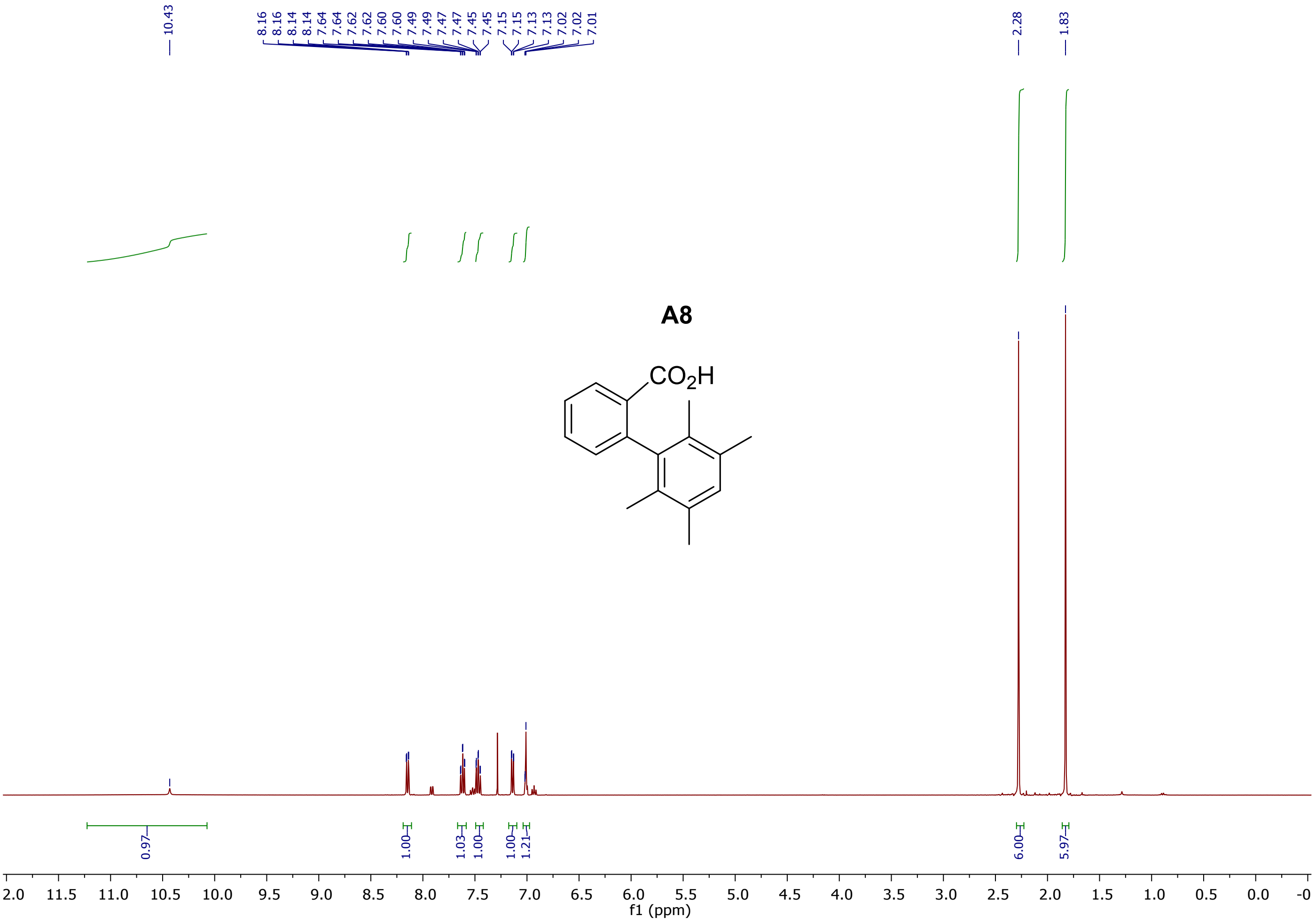

CO_2H
10.43
8.16 8.16 8.14 8.14 7.64 7.64 7.62 7.62 7.60 7.49 7.49 7.47 7.47 7.45 7.45 7.15 7.15 7.13 7.13 7.02 7.02 7.01
2.28
1.83
0.97
1.00
1.03
1.00
1.00
1.21
6.00
5.97
f1 (ppm)

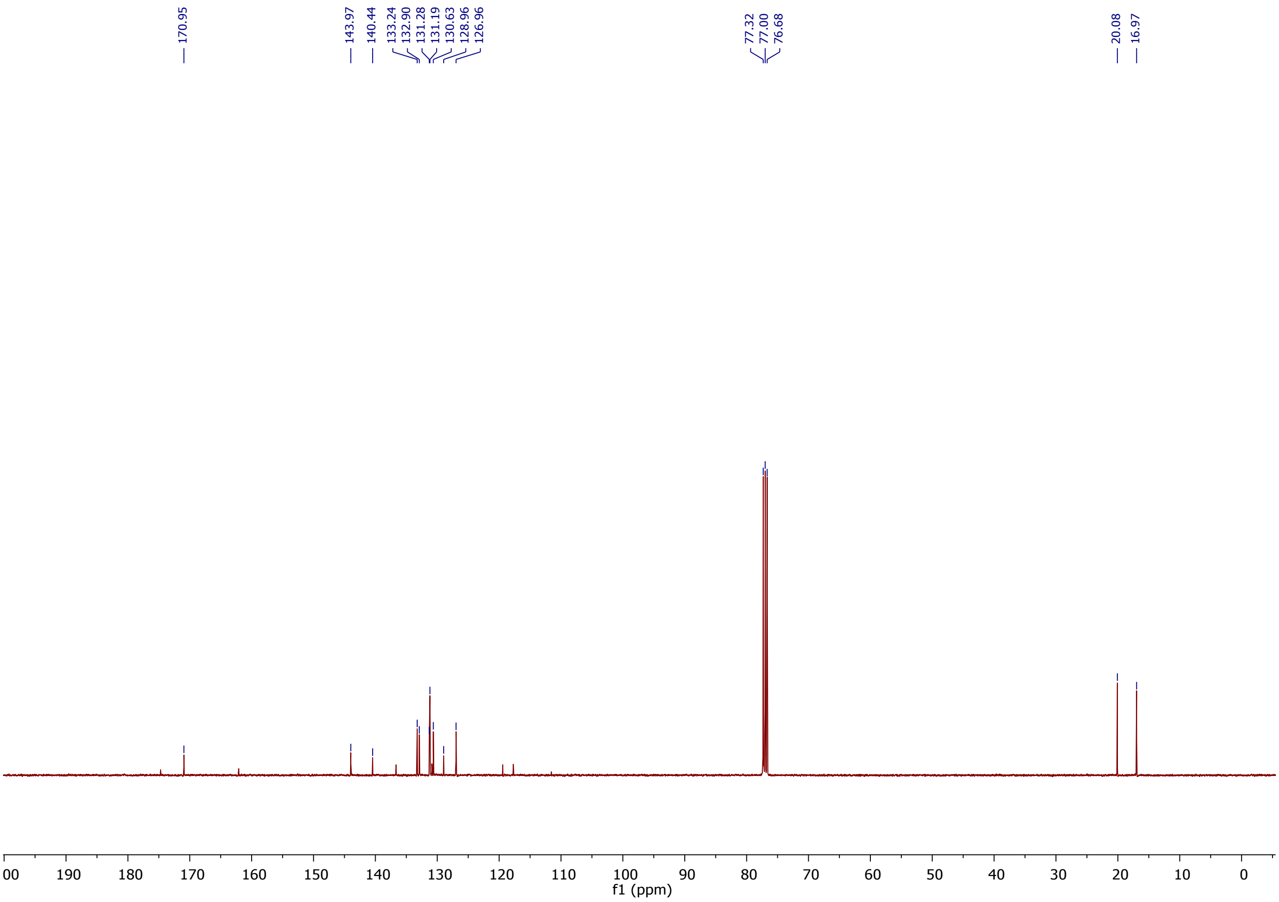

170.95
143.97 140.44 133.24 132.90 131.28 131.19 130.63 128.96 126.96
77.32 77.00 76.68
20.08 16.97
f1 (ppm)

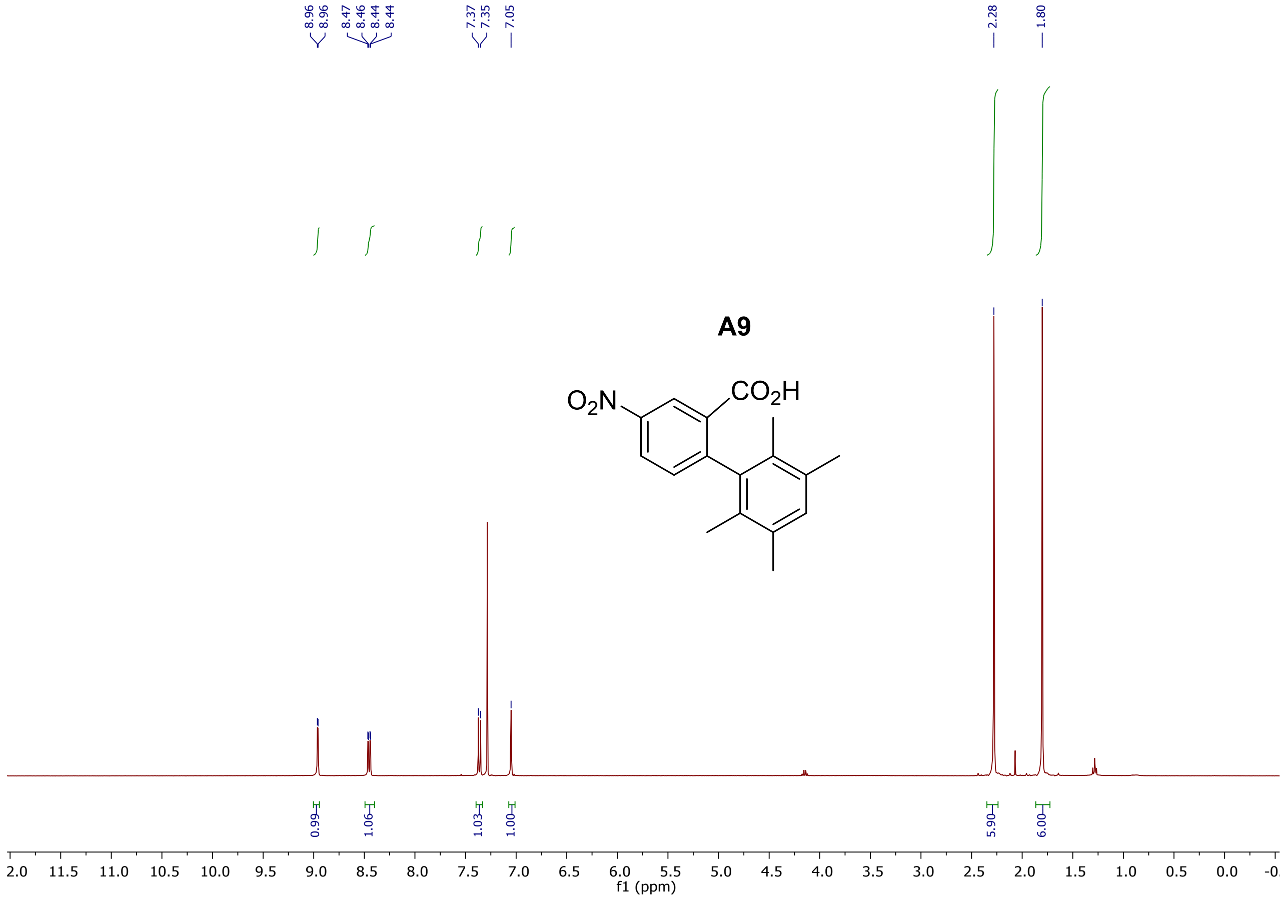

8.96
8.96
8.47
8.46
8.44
8.44
7.37
7.35
7.05
2.28
1.80
A9
O_2N
CO_2H
0.99
1.06
1.03
1.00
5.90
6.00
f1 (ppm)

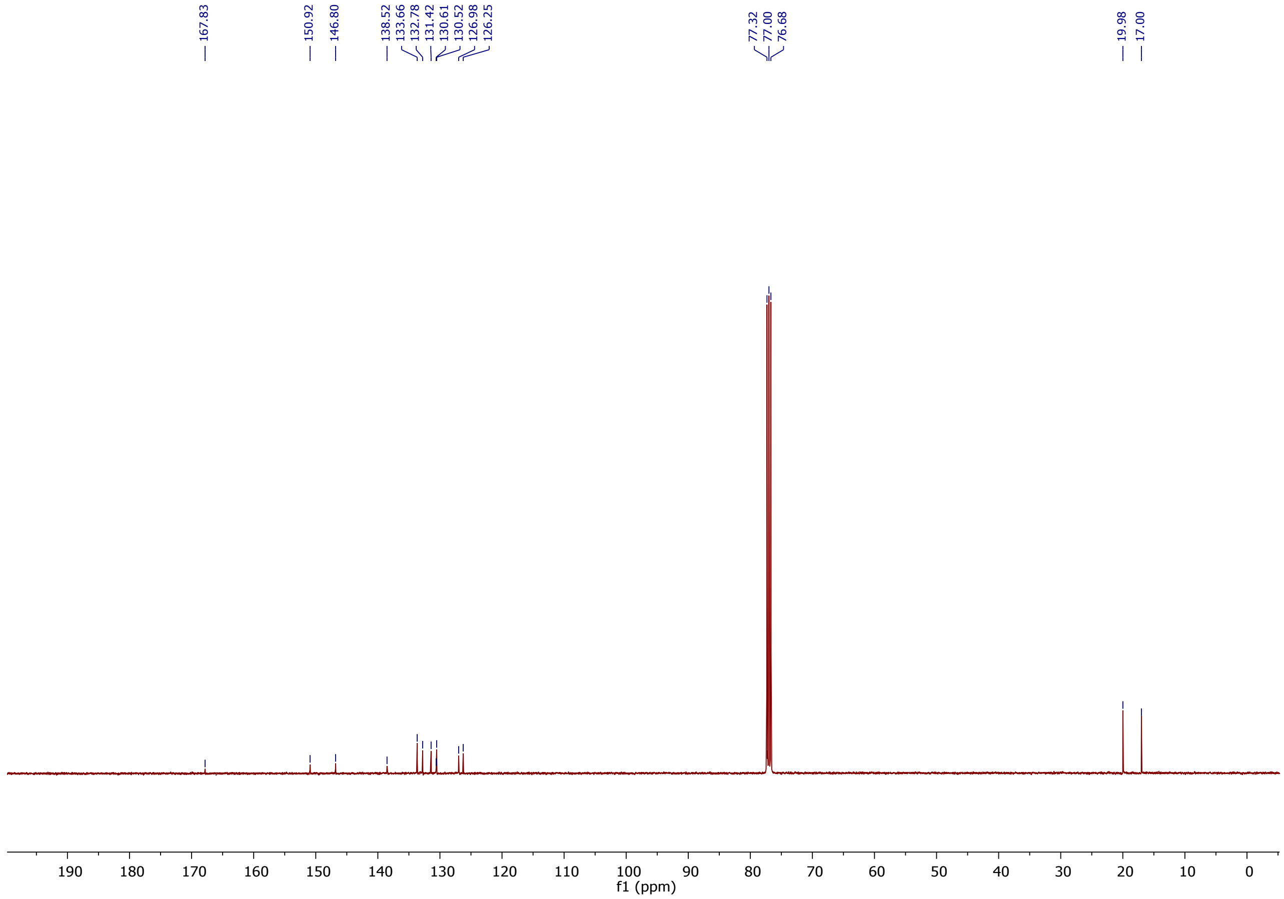

167.83
150.92
146.80
138.52
133.66
132.78
131.42
130.61
130.52
126.98
126.25
77.32
77.00
76.68
19.98
17.00
f1 (ppm)

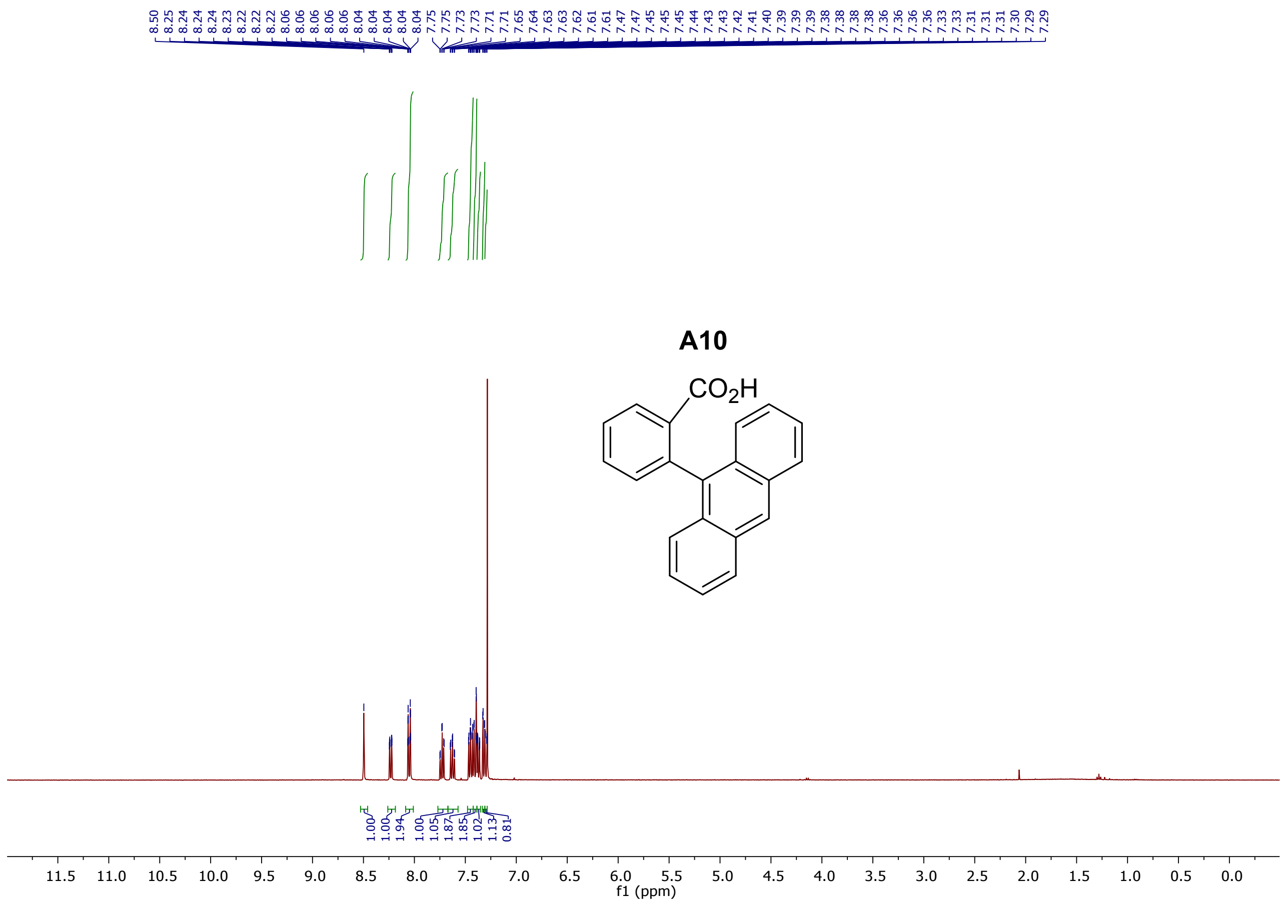
8.50
8.25
8.24
8.24
8.23
8.22
8.22
8.22
8.06
8.06
8.06
8.06
8.04
8.04
8.04
8.04
8.04
7.75
7.75
7.73
7.73
7.71
7.71
7.65
7.64
7.63
7.63
7.62
7.61
7.61
7.47
7.45
7.45
7.45
7.44
7.43
7.43
7.42
7.41
7.40
7.39
7.39
7.39
7.38
7.38
7.38
7.38
7.36
7.36
7.36
7.33
7.33
7.31
7.31
7.30
7.29
7.29
A10
CO2H
1.00
1.00
1.94
1.00
1.05
1.87
1.85
1.02
1.13
0.81
11.5 11.0 10.5 10.0 9.5 9.0 8.5 8.0 7.5 7.0 6.5 6.0 5.5 5.0 4.5 4.0 3.5 3.0 2.5 2.0 1.5 1.0 0.5 0.0
f1 (ppm)

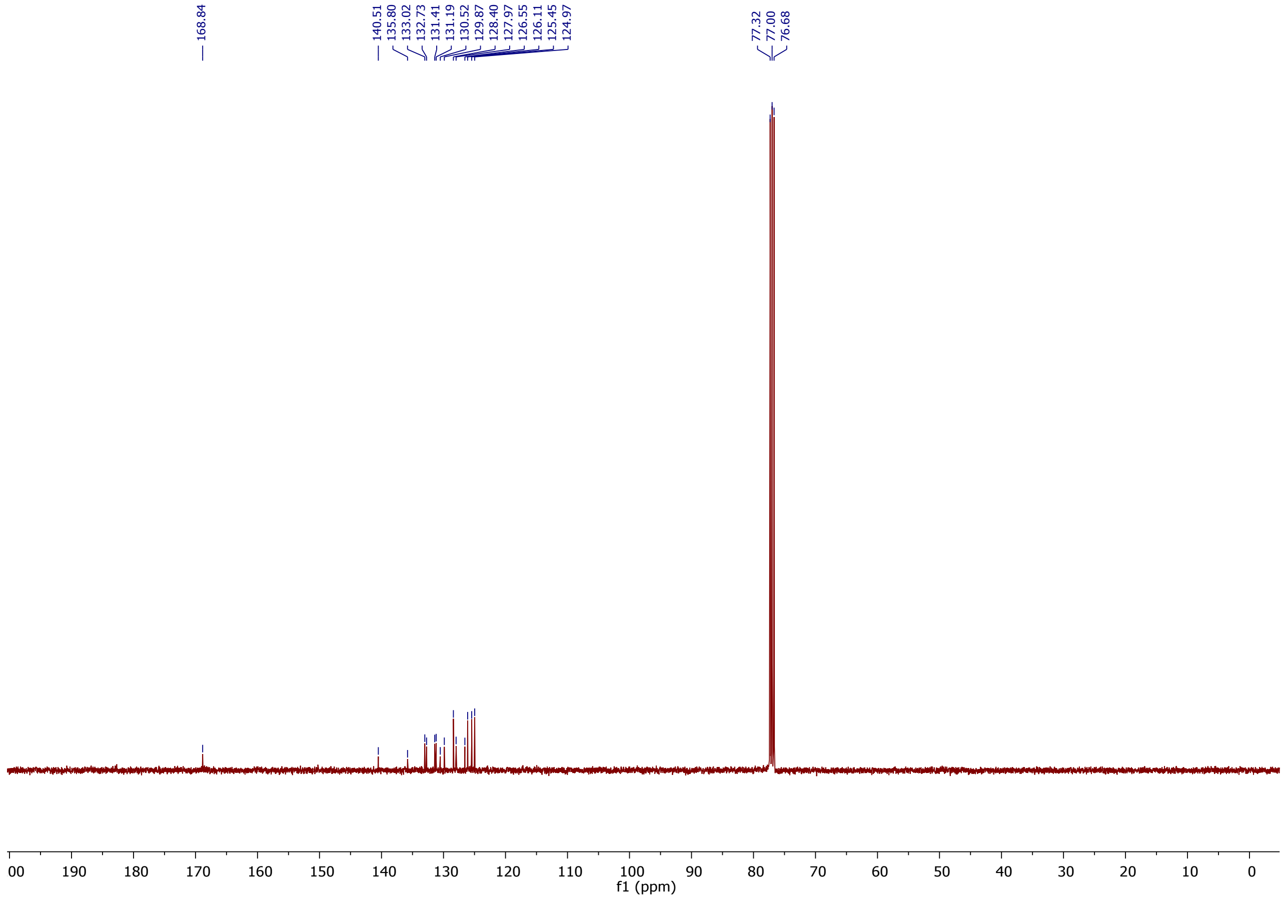
168.84
140.51
135.80
133.02
132.73
131.41
131.19
130.52
129.87
128.40
127.97
126.55
126.11
125.45
124.97
77.32
77.00
76.68
00 190 180 170 160 150 140 130 120 110 100 90 80 70 60 50 40 30 20 10 0
f1 (ppm)

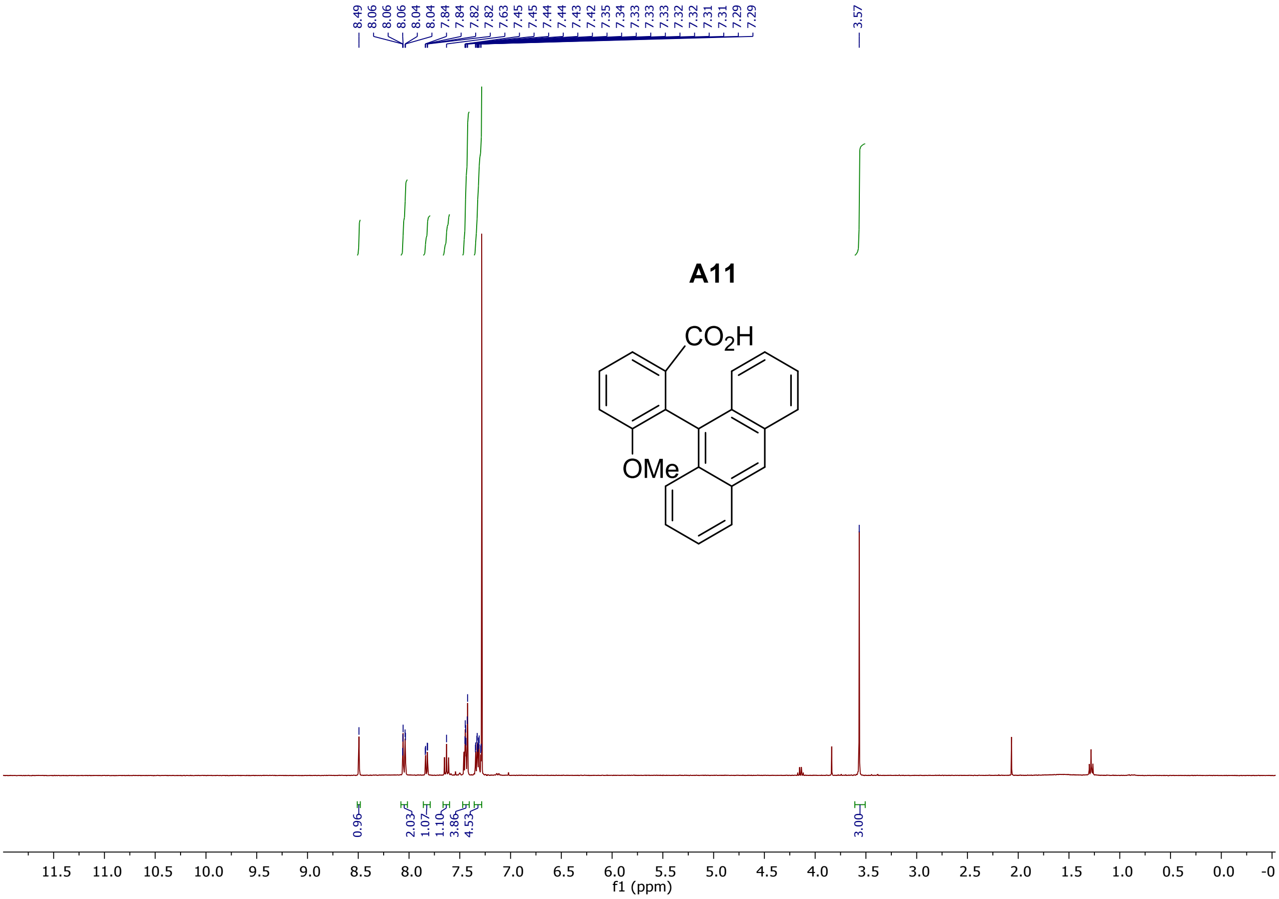
8.49 8.06 8.06 8.06 8.04 8.04 7.84 7.84 7.82 7.82 7.63 7.45 7.45 7.44 7.44 7.43 7.42 7.35 7.34 7.33 7.33 7.32 7.32 7.31 7.31 7.29 7.29
3.57
A11
CO2H
OMe
0.96 2.03 1.07 1.10 3.86 4.53
3.00
11.5 11.0 10.5 10.0 9.5 9.0 8.5 8.0 7.5 7.0 6.5 6.0 5.5 5.0 4.5 4.0 3.5 3.0 2.5 2.0 1.5 1.0 0.5 0.0 -0
f1 (ppm)
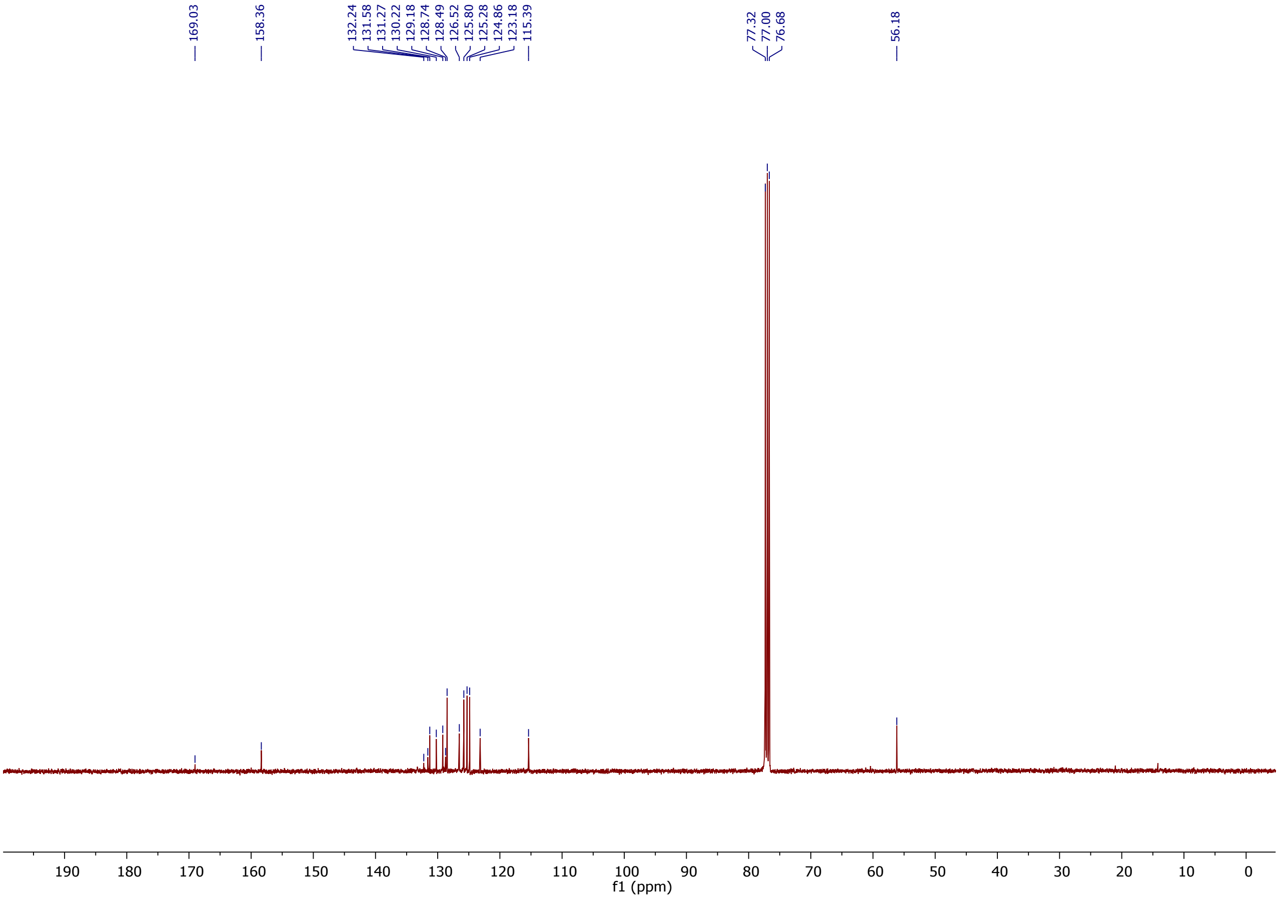
169.03
158.36
132.24 131.58 131.27 130.22 129.18 128.74 128.49 126.52 125.80 125.28 124.86 123.18 115.39
77.32 77.00 76.68
56.18
190 180 170 160 150 140 130 120 110 100 90 80 70 60 50 40 30 20 10 0
f1 (ppm)

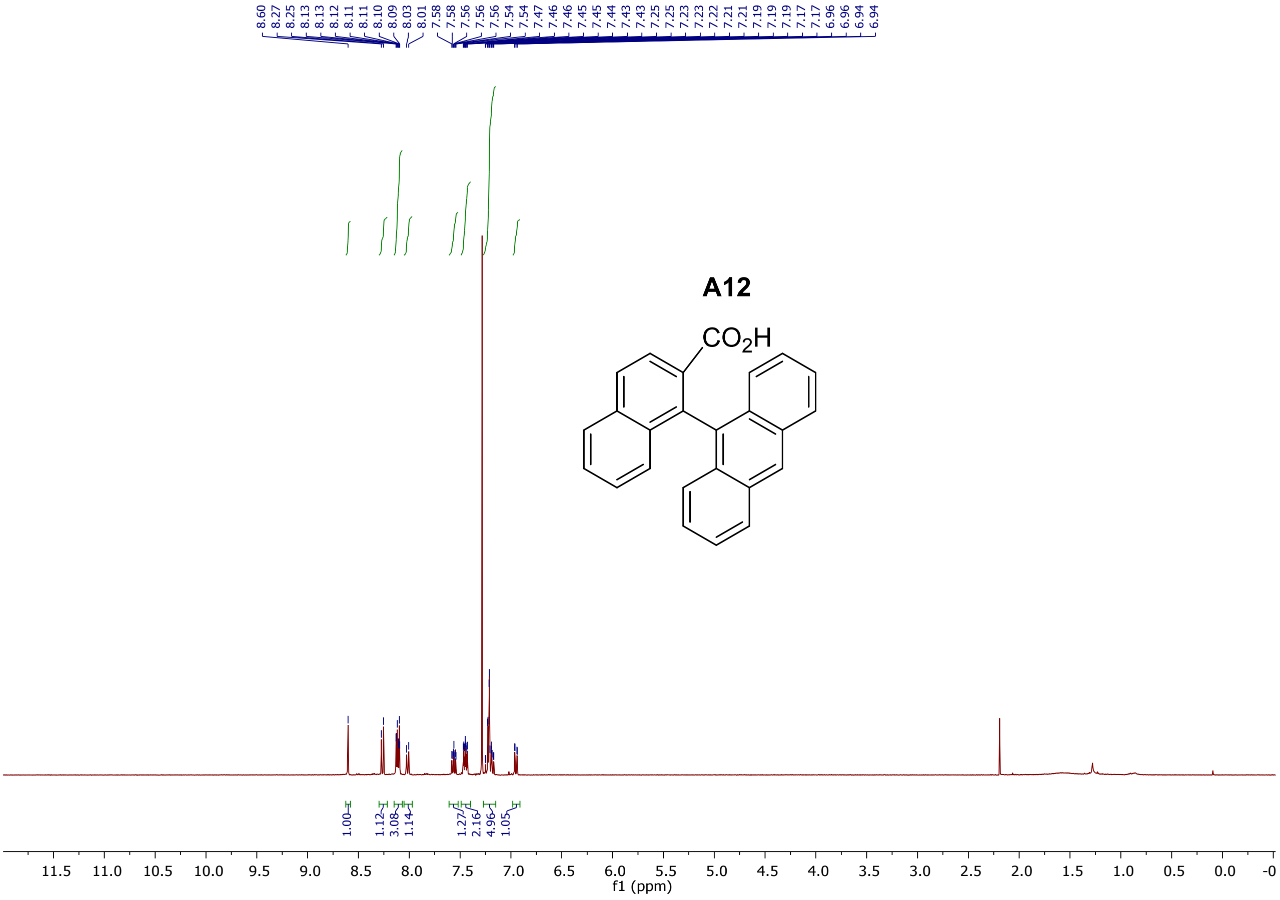
8.60 8.27 8.25 8.13 8.13 8.12 8.11 8.11 8.10 8.09 8.03 8.01 7.58 7.58 7.56 7.56 7.56 7.54 7.54 7.47 7.46 7.46 7.45 7.45 7.44 7.43 7.43 7.25 7.25 7.23 7.23 7.22 7.21 7.21 7.19 7.19 7.19 7.17 6.96 6.96 6.94 6.94
A12
CO_2H
1.00 1.12 3.08 1.14 1.27 2.16 4.96 1.05
11.5 11.0 10.5 10.0 9.5 9.0 8.5 8.0 7.5 7.0 6.5 6.0 5.5 5.0 4.5 4.0 3.5 3.0 2.5 2.0 1.5 1.0 0.5 0.0 -0
f1 (ppm)

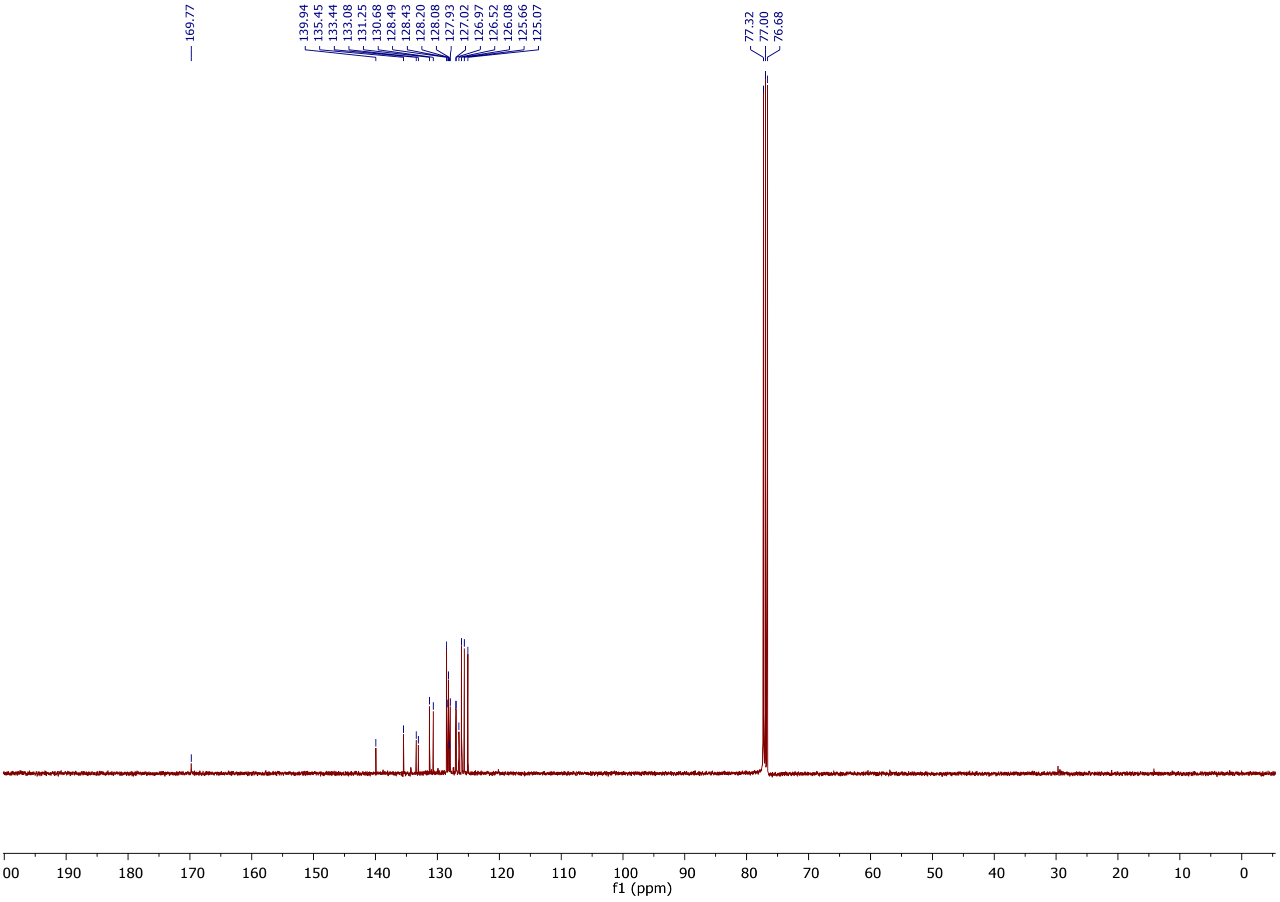
169.77
139.94 135.45 133.44 133.08 131.25 130.68 128.49 128.43 128.20 128.08 127.93 127.02 126.97 126.52 126.08 125.66 125.07
77.32 77.00 76.68
00 190 180 170 160 150 140 130 120 110 100 90 80 70 60 50 40 30 20 10 0
f1 (ppm)

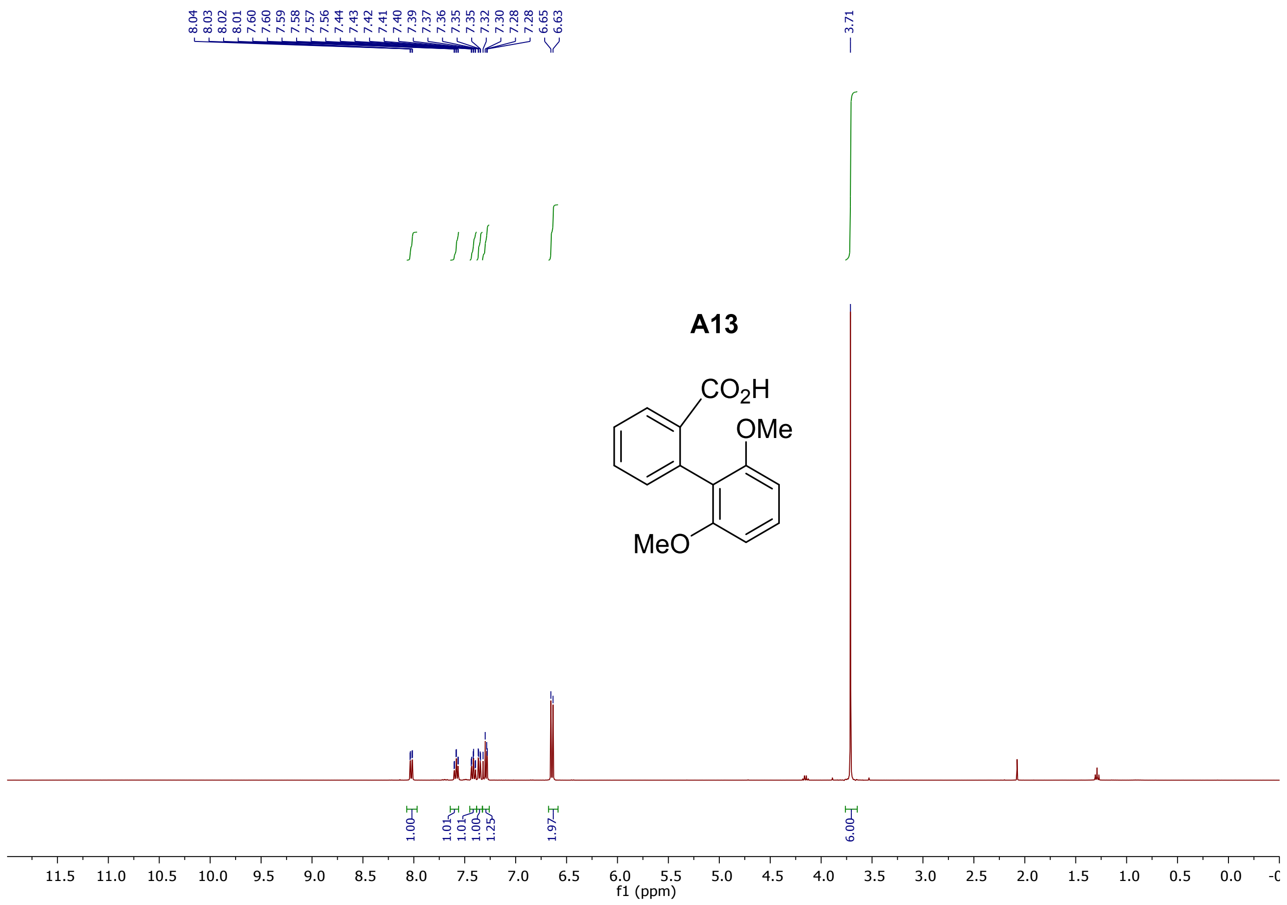
8.04
8.03
8.02
8.01
7.60
7.59
7.58
7.57
7.56
7.44
7.43
7.42
7.41
7.40
7.39
7.37
7.36
7.35
7.35
7.32
7.30
7.28
6.65
6.63
3.71
A13
CO2H
OMe
MeO
1.00
1.01
1.01
1.00
1.25
1.97
6.00
11.5 11.0 10.5 10.0 9.5 9.0 8.5 8.0 7.5 7.0 6.5 6.0 5.5 5.0 4.5 4.0 3.5 3.0 2.5 2.0 1.5 1.0 0.5 0.0
f1 (ppm)

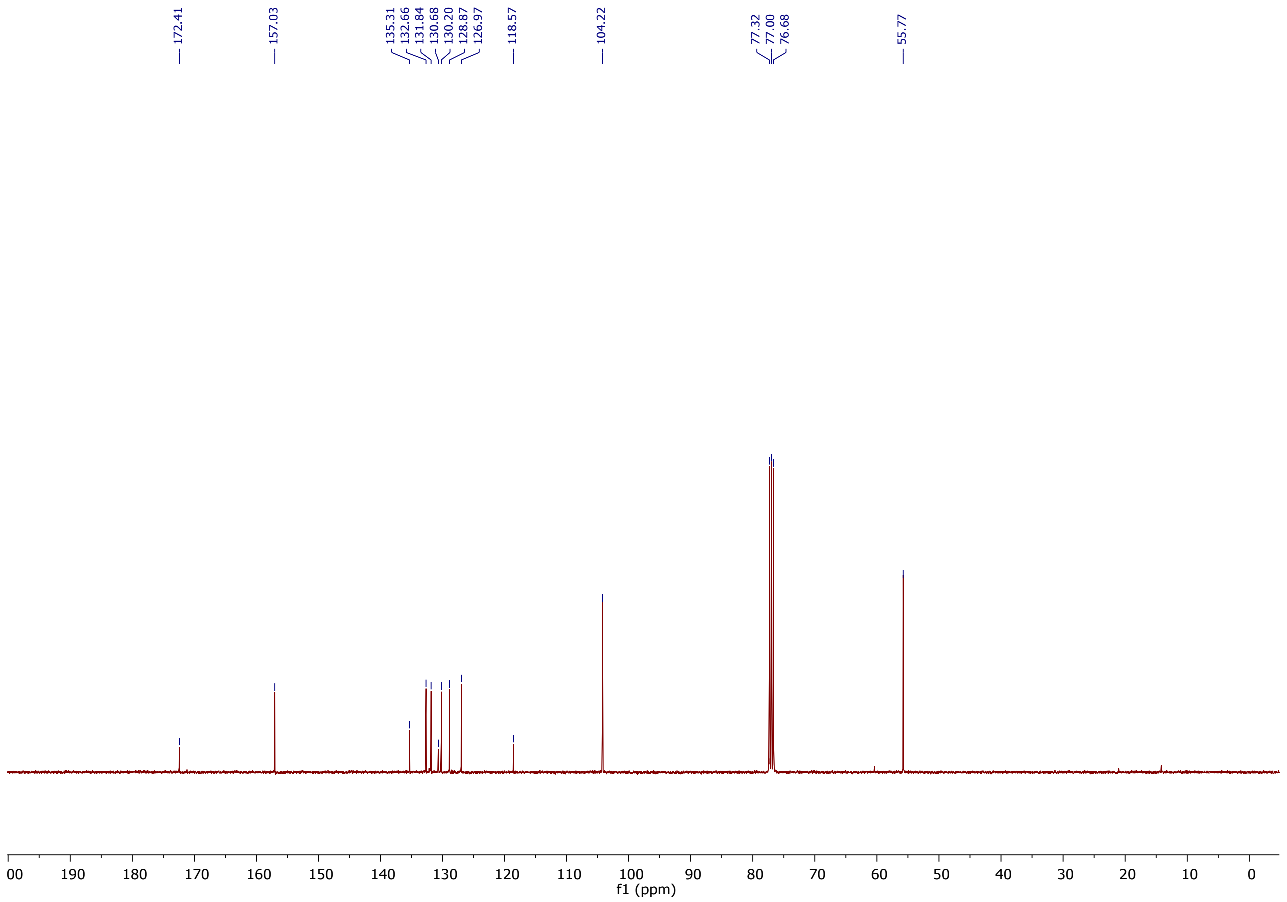
172.41
157.03
135.31
132.66
131.84
130.68
130.20
128.87
126.97
118.57
104.22
77.32
77.00
76.68
55.77
190 180 170 160 150 140 130 120 110 100 90 80 70 60 50 40 30 20 10 0
f1 (ppm)

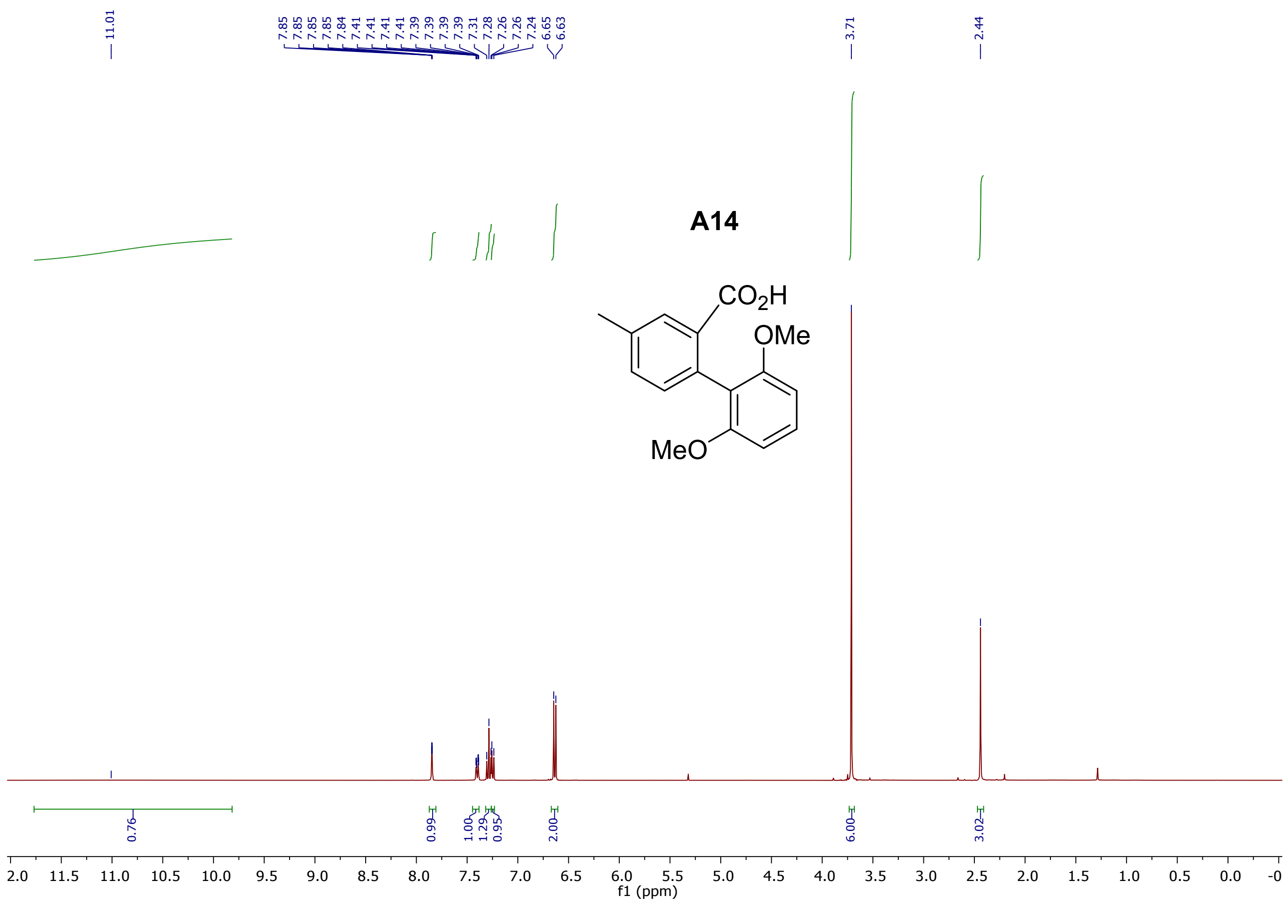
A14
CO2H
OMe
MeO
f1 (ppm)

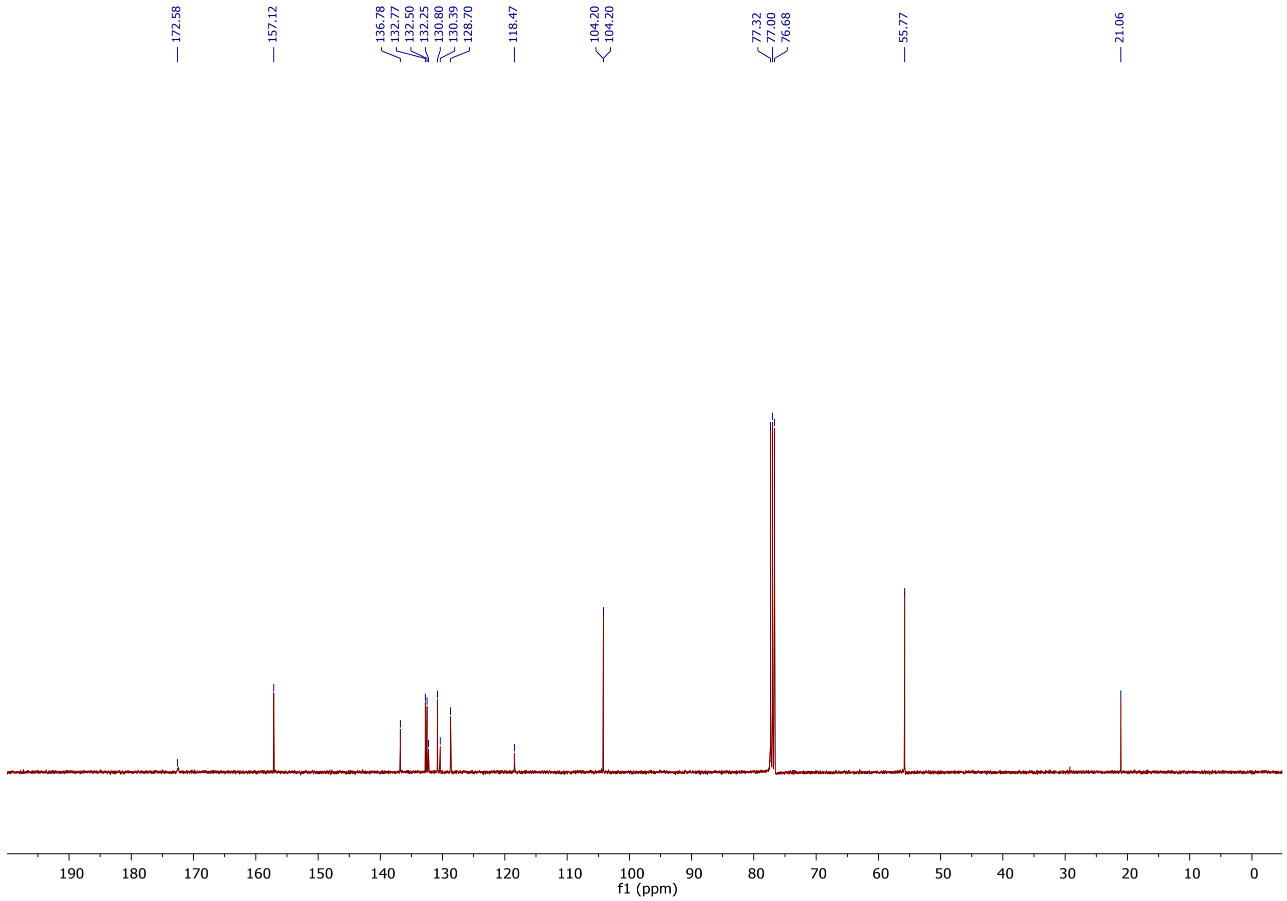
f1 (ppm)

A15

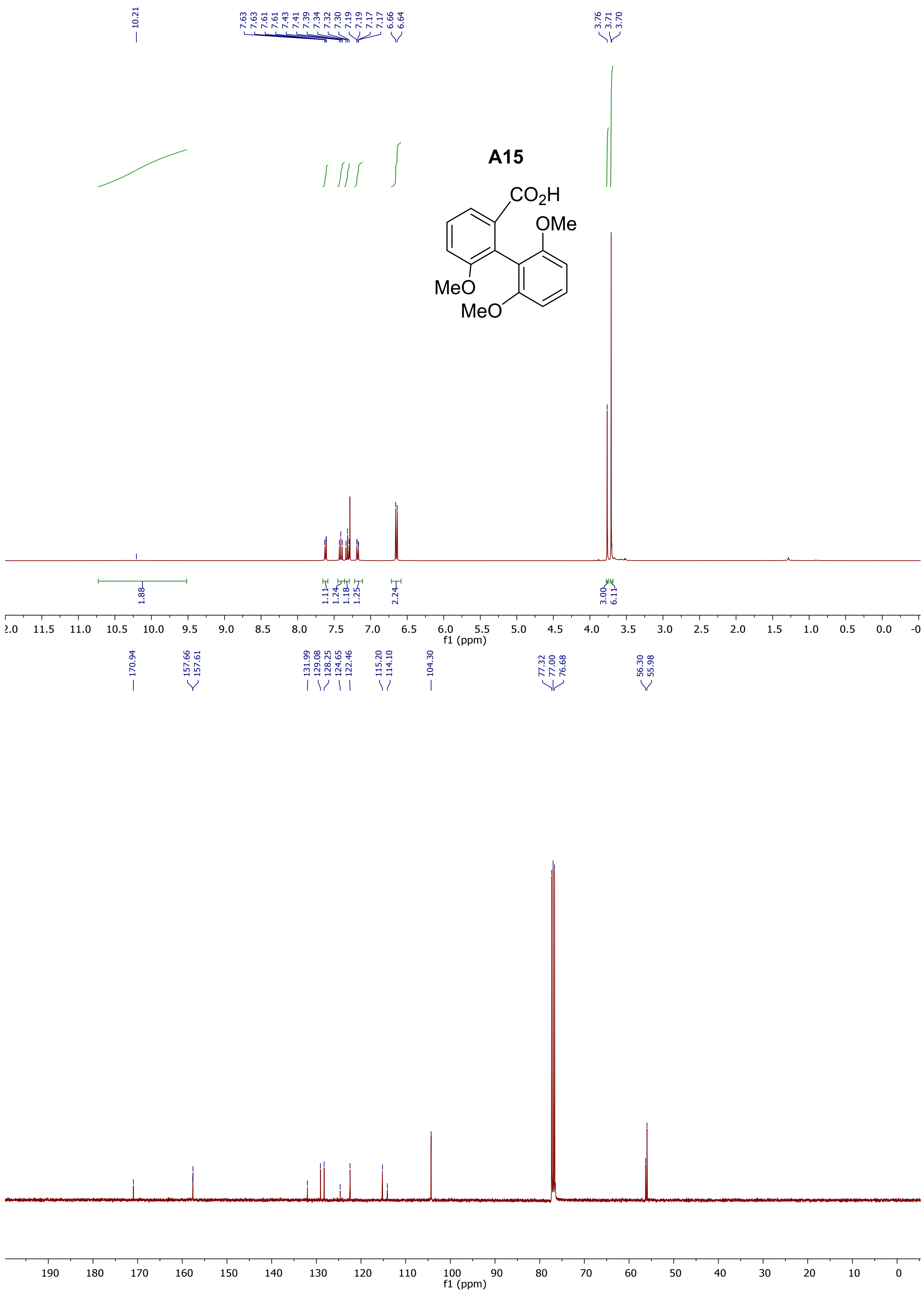
10.21
7.63
7.63
7.61
7.61
7.43
7.41
7.39
7.34
7.32
7.30
7.19
7.19
7.17
7.17
6.66
6.64
3.76
3.71
3.70
CO2H
OMe
MeO
MeO
1.88
1.11
1.24
1.18
1.25
2.24
3.00
6.11
f1 (ppm)
170.94
157.66
157.61
131.99
129.08
128.25
124.65
122.46
115.20
114.10
104.30
77.32
77.00
76.68
56.30
55.98
f1 (ppm)

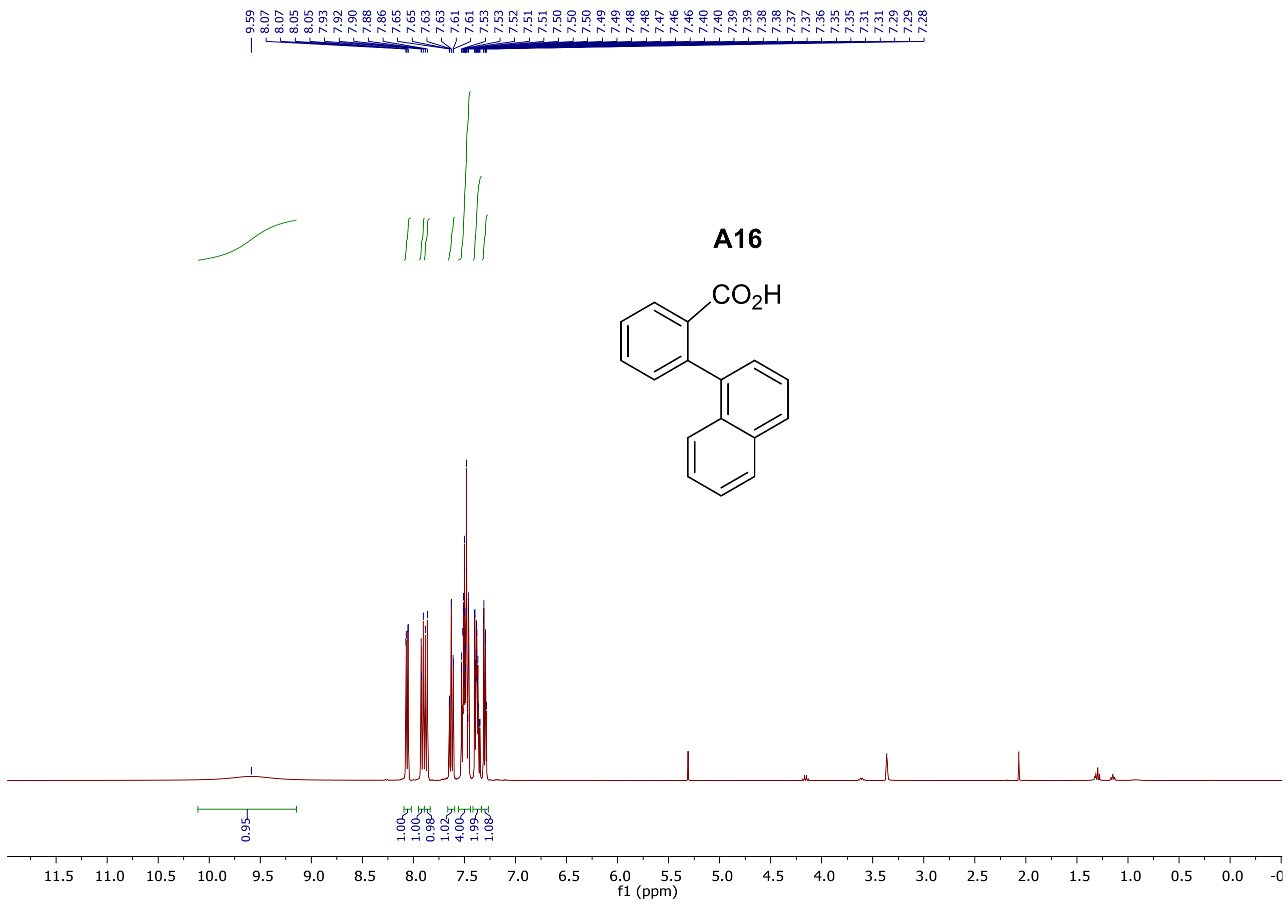

9.59 8.07 8.07 8.05 8.05 7.93 7.92 7.90 7.88 7.86 7.65 7.65 7.63 7.63 7.61 7.61 7.53 7.53 7.52 7.51 7.51 7.50 7.50 7.50 7.49 7.49 7.48 7.48 7.47 7.46 7.46 7.40 7.40 7.39 7.39 7.38 7.38 7.37 7.37 7.36 7.35 7.35 7.31 7.29 7.29 7.28
A16
CO2H
0.95 1.00 1.00 0.98 1.02 4.00 1.99 1.08
f1 (ppm)

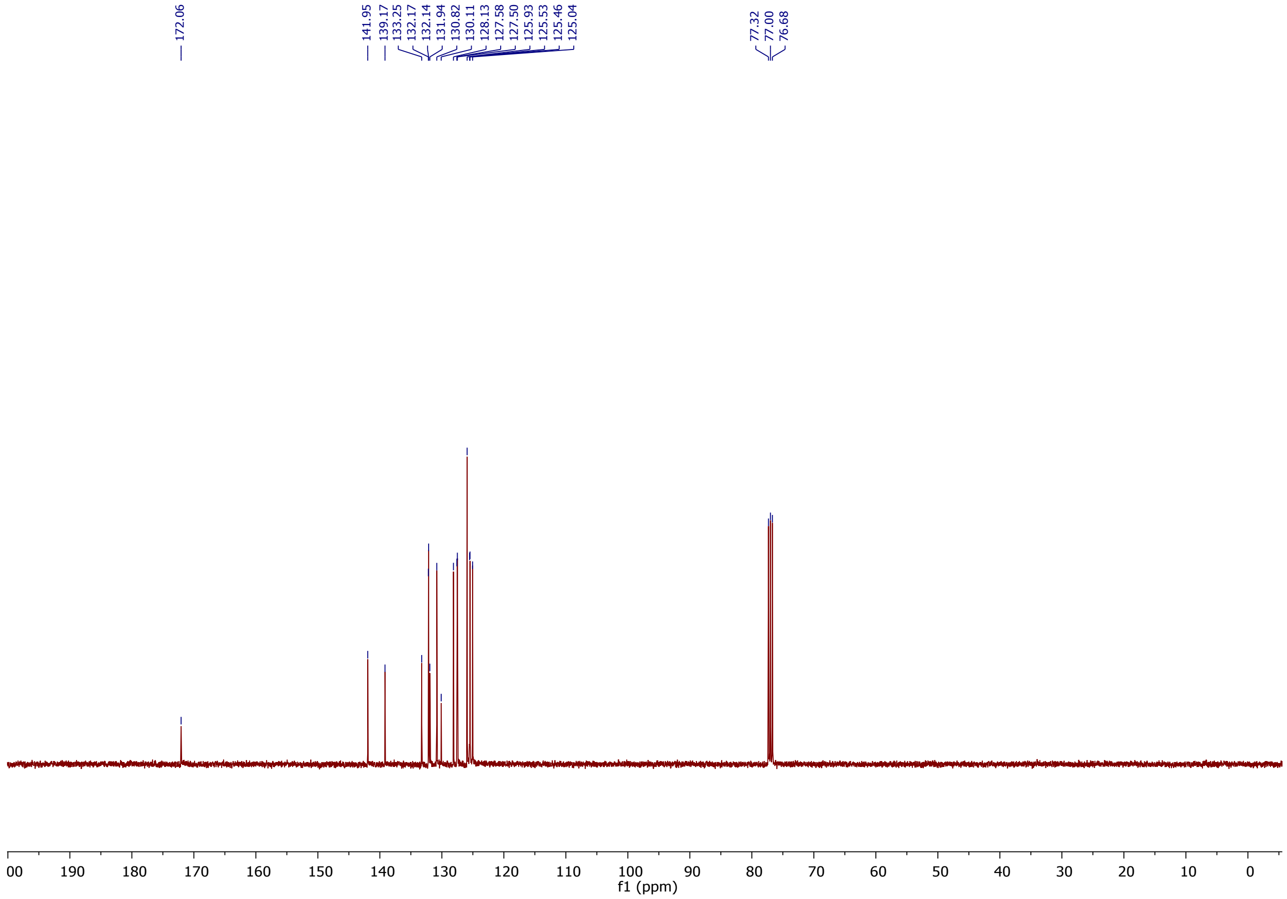

172.06 141.95 139.17 133.25 132.17 132.14 131.94 130.82 130.11 128.13 127.58 127.50 125.93 125.53 125.46 125.04 77.32 77.00 76.68
f1 (ppm)

A17

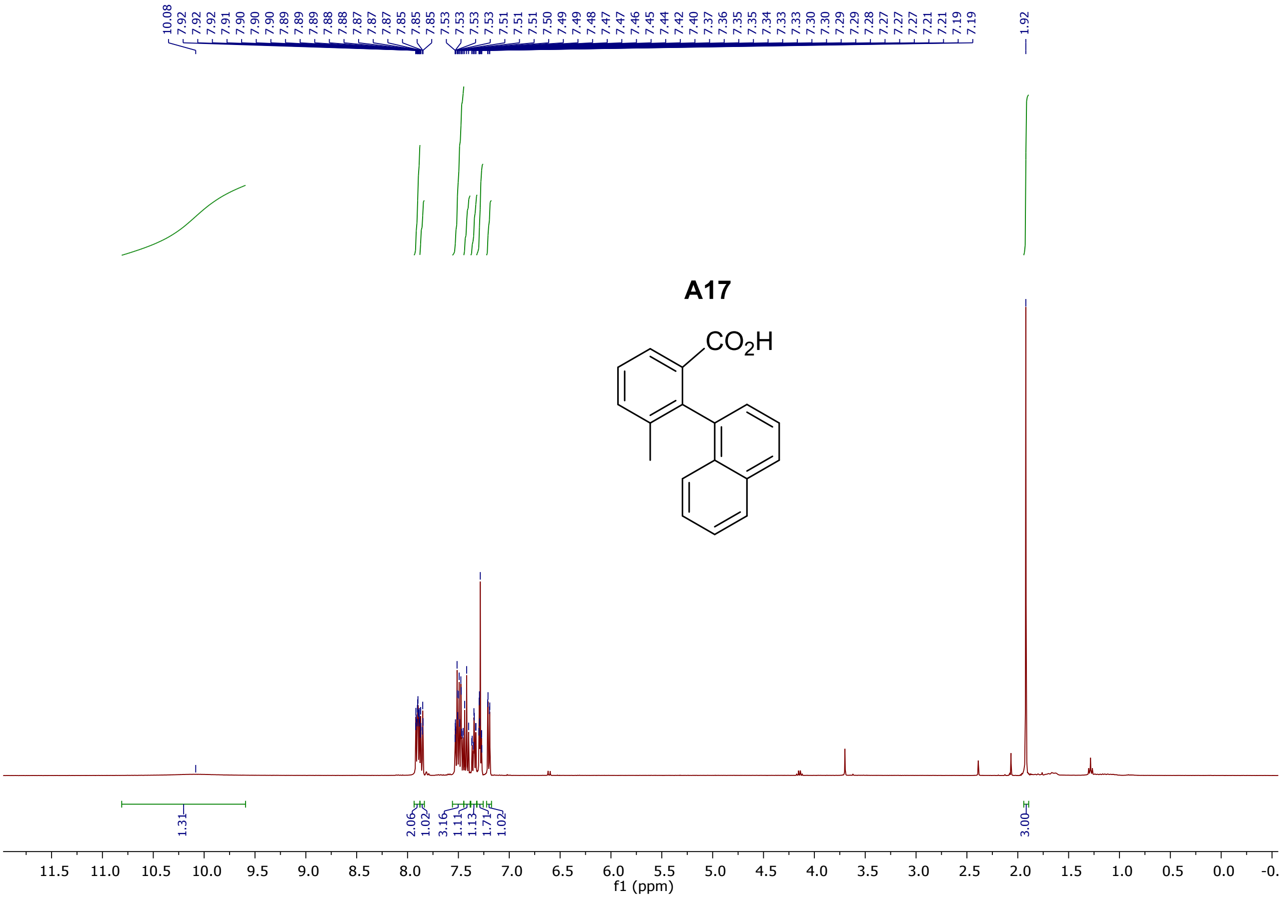
CO2H
10.08
7.92
7.92
7.91
7.90
7.90
7.89
7.89
7.88
7.88
7.87
7.87
7.85
7.85
7.53
7.53
7.53
7.51
7.51
7.50
7.49
7.49
7.48
7.47
7.46
7.45
7.44
7.42
7.40
7.37
7.36
7.35
7.35
7.34
7.33
7.33
7.30
7.30
7.29
7.29
7.28
7.27
7.27
7.27
7.21
7.21
7.19
7.19
1.92
1.31
2.06
1.02
3.16
1.11
1.13
1.71
1.02
3.00
11.5 11.0 10.5 10.0 9.5 9.0 8.5 8.0 7.5 7.0 6.5 6.0 5.5 5.0 4.5 4.0 3.5 3.0 2.5 2.0 1.5 1.0 0.5 0.0 -0.
f1 (ppm)

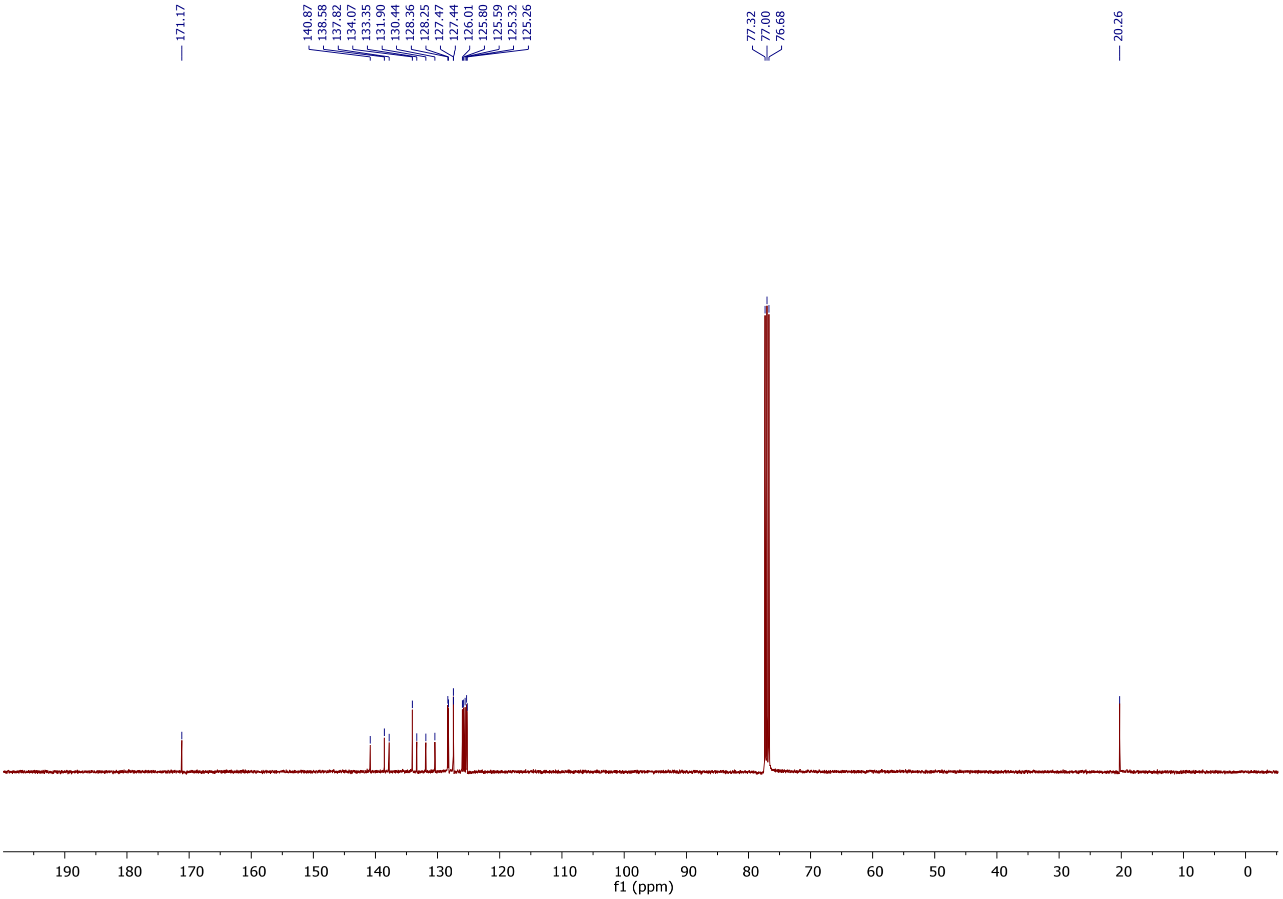
171.17
140.87
138.58
137.82
134.07
133.35
131.90
130.44
128.36
128.25
127.47
127.44
126.01
125.80
125.59
125.32
125.26
77.32
77.00
76.68
20.26
190 180 170 160 150 140 130 120 110 100 90 80 70 60 50 40 30 20 10 0
f1 (ppm)

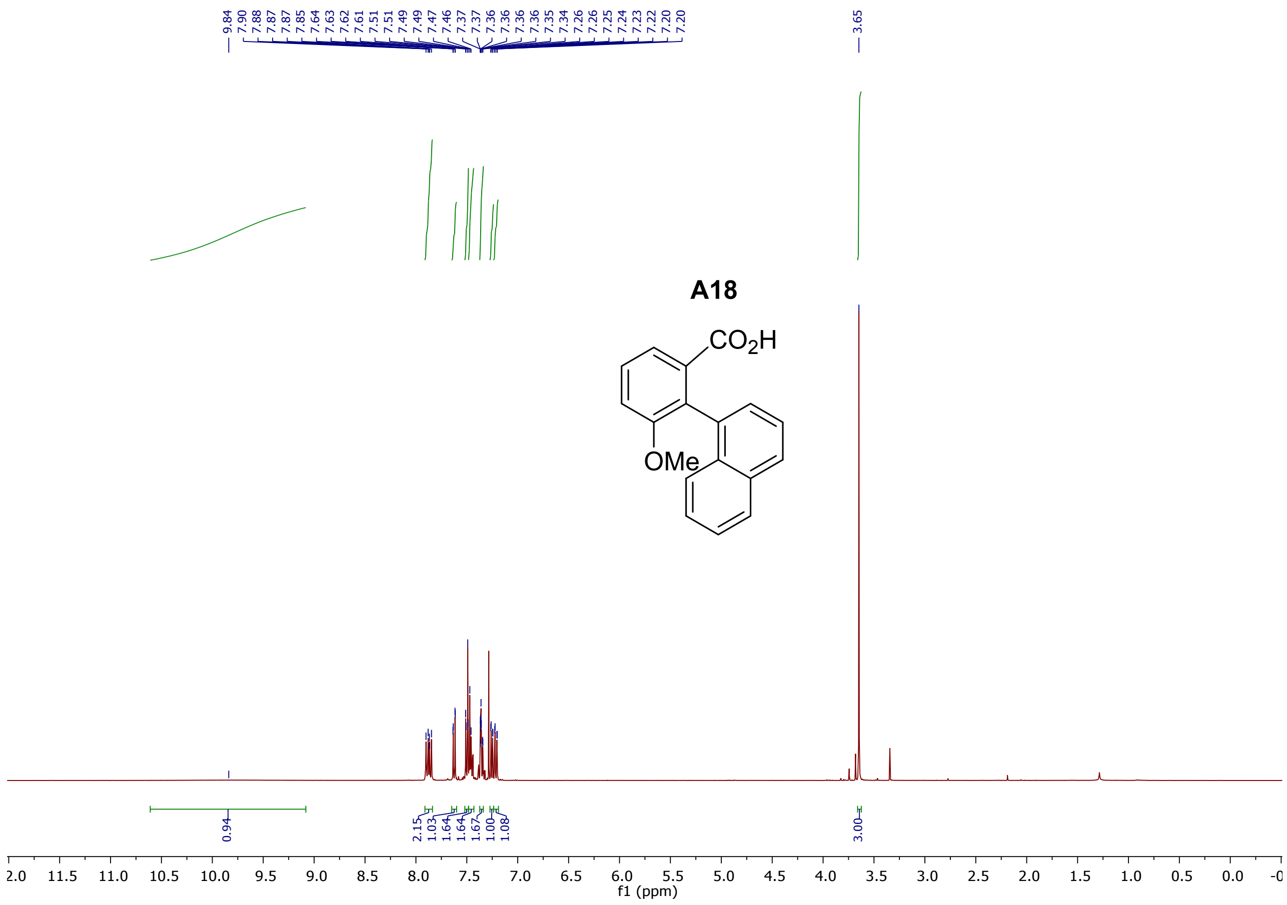
A18
CO2H
OMe
f1 (ppm)

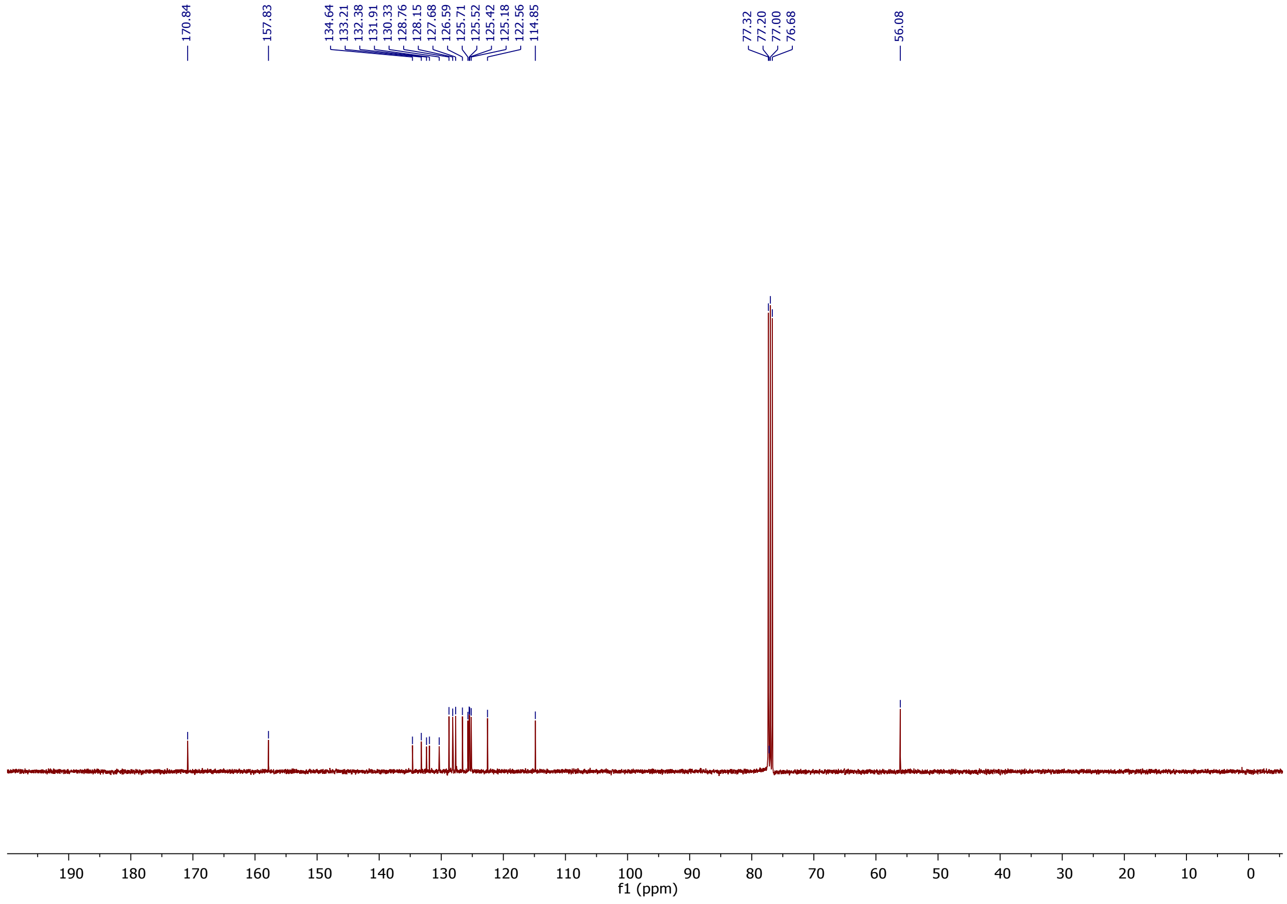
f1 (ppm)

A19

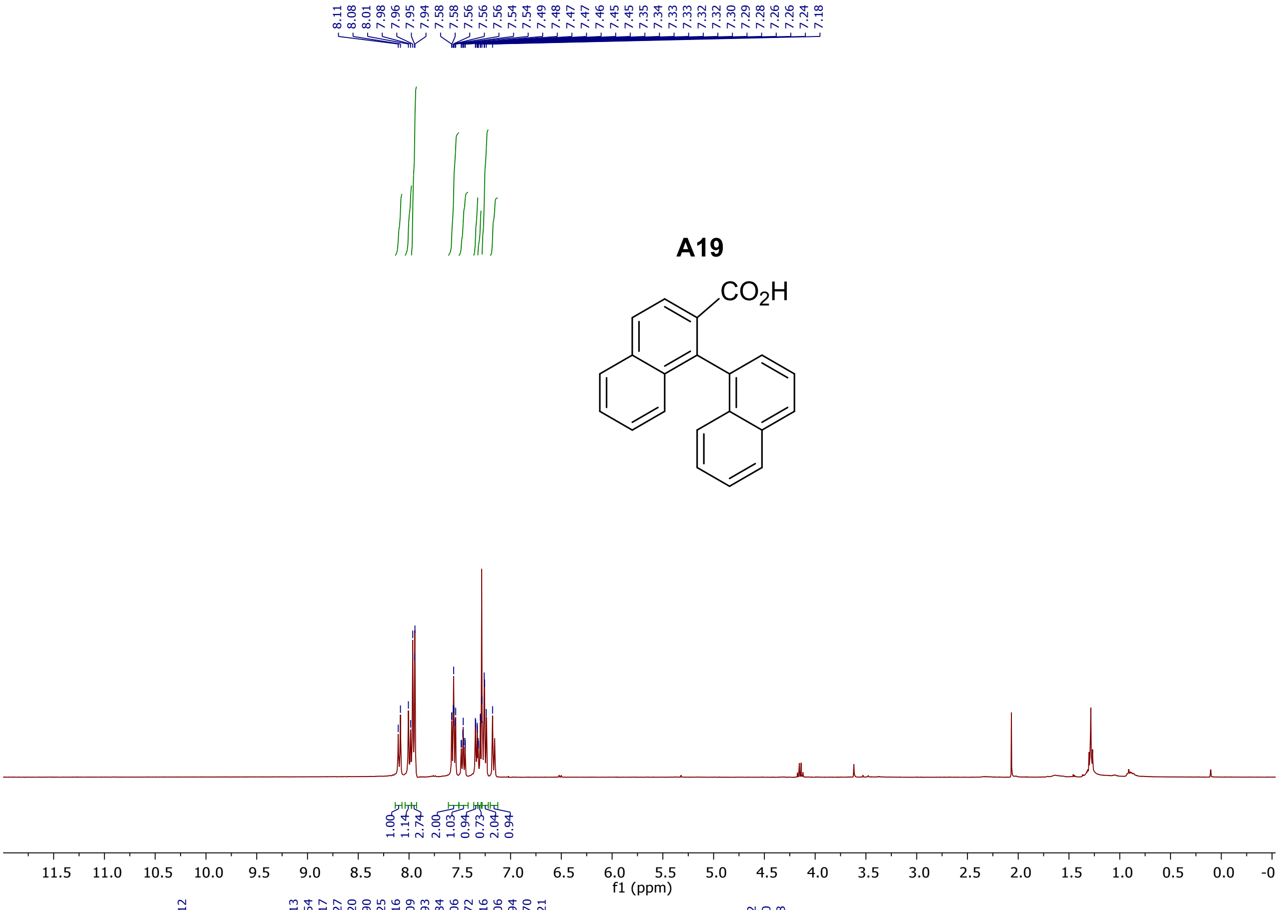
CO2H
8.11
8.08
8.01
7.98
7.96
7.95
7.94
7.58
7.58
7.56
7.56
7.56
7.54
7.54
7.49
7.48
7.47
7.47
7.46
7.45
7.45
7.35
7.34
7.33
7.33
7.32
7.32
7.30
7.29
7.28
7.26
7.24
7.18
1.00
1.14
2.74
2.00
1.03
0.94
0.73
2.04
0.94
11.5 11.0 10.5 10.0 9.5 9.0 8.5 8.0 7.5 7.0 6.5 6.0 5.5 5.0 4.5 4.0 3.5 3.0 2.5 2.0 1.5 1.0 0.5 0.0 -0
f1 (ppm)

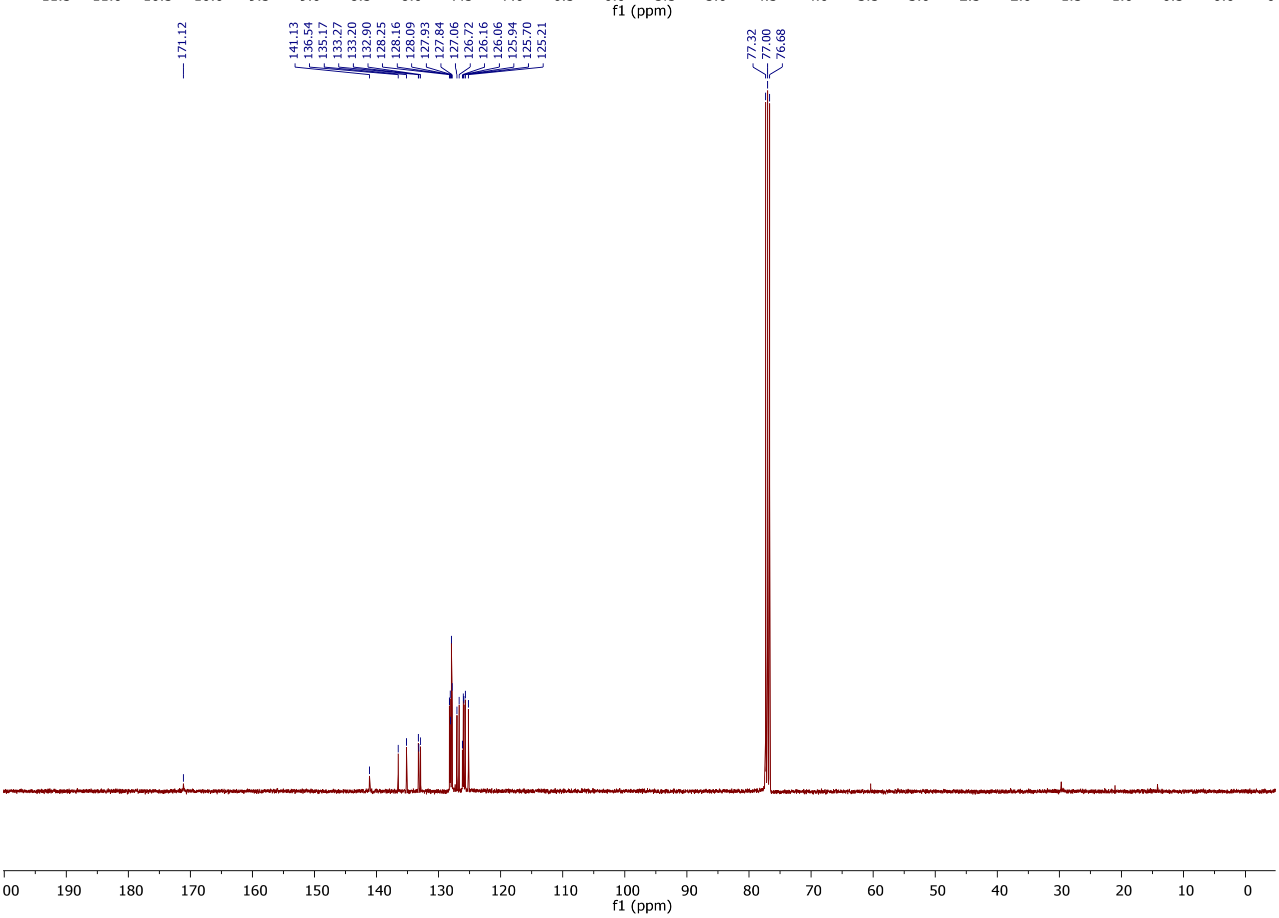
171.12
141.13
136.54
135.17
133.27
133.20
132.90
128.25
128.16
128.09
127.93
127.84
127.06
126.72
126.16
126.06
125.94
125.70
125.21
77.32
77.00
76.68
00 190 180 170 160 150 140 130 120 110 100 90 80 70 60 50 40 30 20 10 0
f1 (ppm)

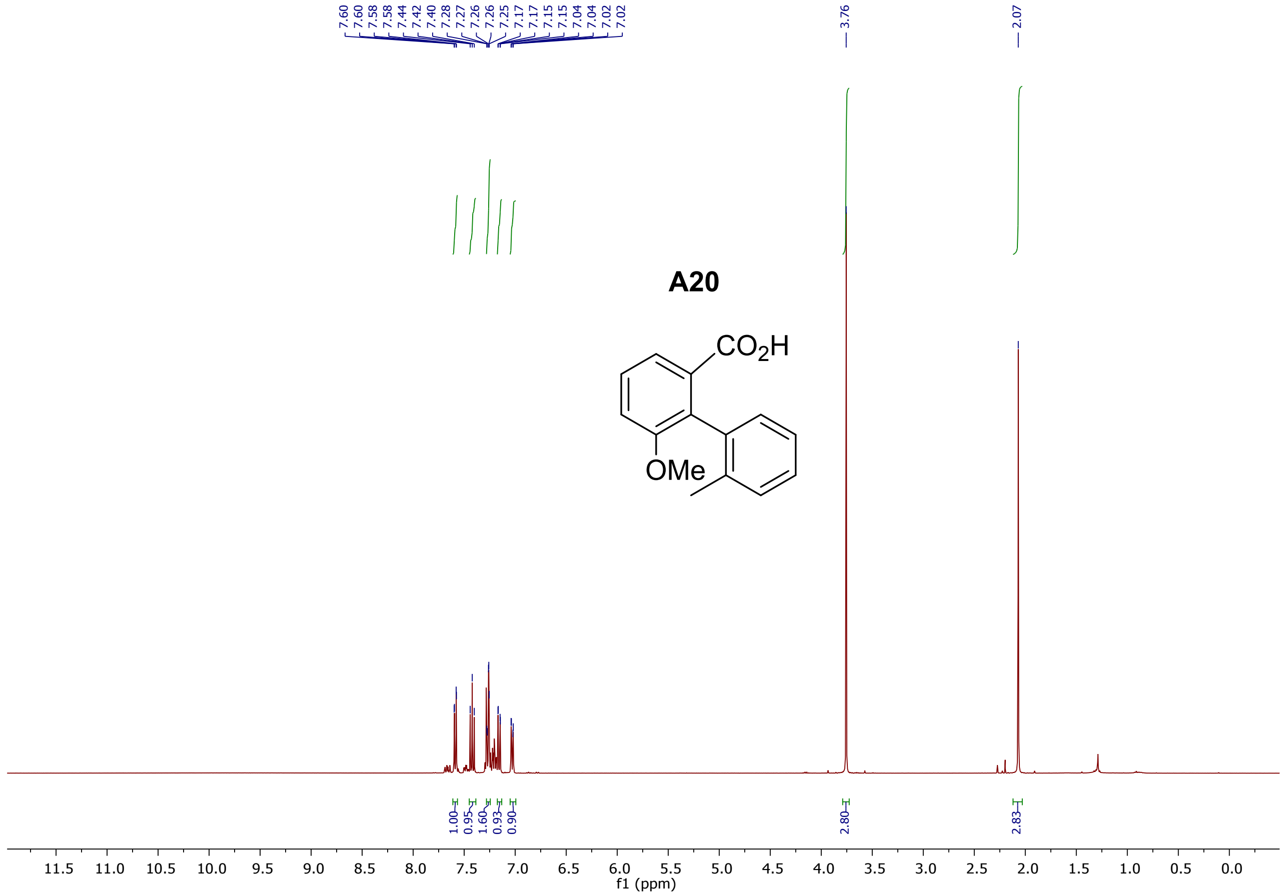
7.60
7.60
7.58
7.58
7.44
7.42
7.40
7.28
7.27
7.26
7.26
7.25
7.17
7.17
7.15
7.15
7.04
7.04
7.02
7.02
3.76
2.07
A20
CO_2H
OMe
1.00
0.95
1.60
0.93
0.90
2.80
2.83
f1 (ppm)

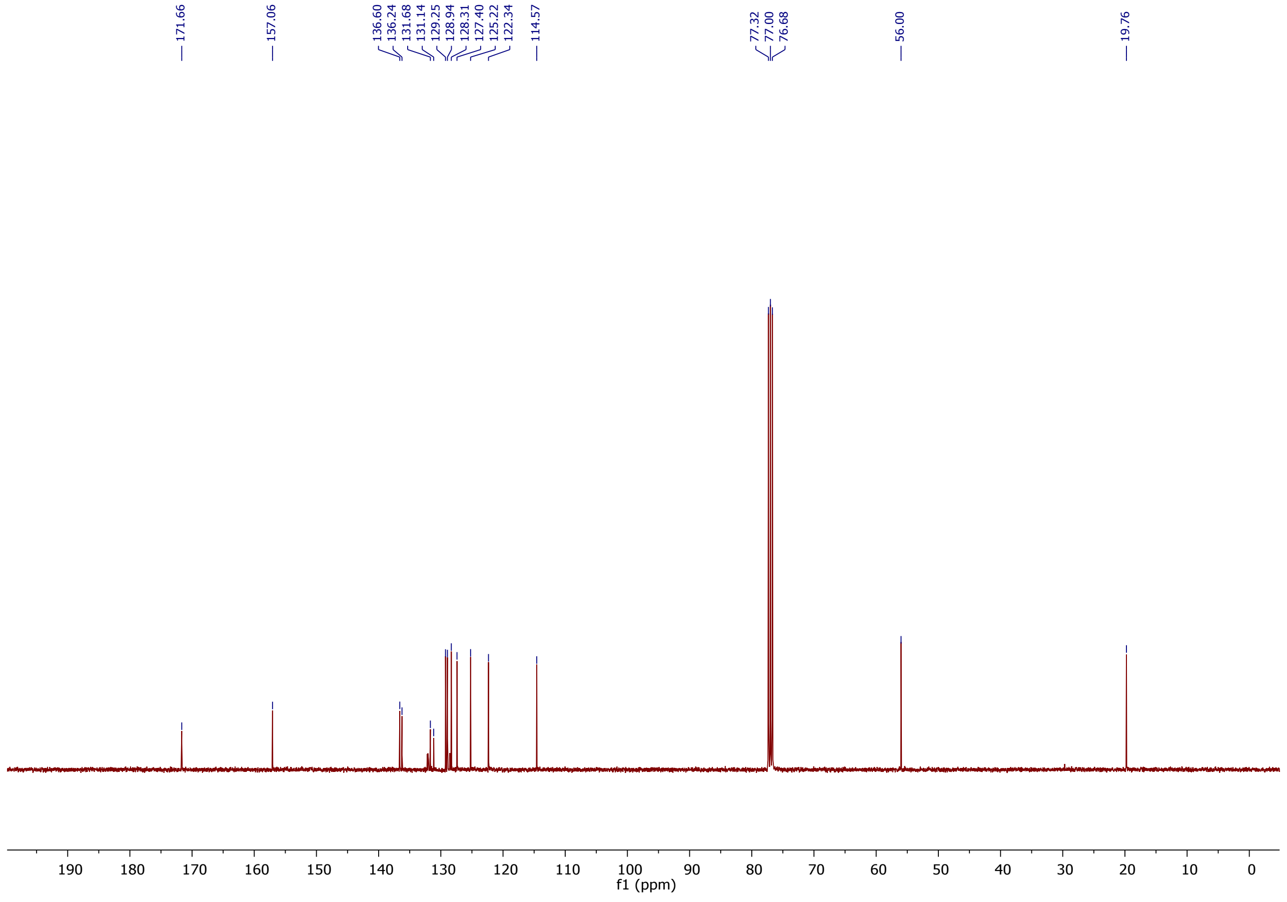
171.66
157.06
136.60
136.24
131.68
131.14
129.25
128.94
128.31
127.40
125.22
122.34
114.57
77.32
77.00
76.68
56.00
19.76
f1 (ppm)

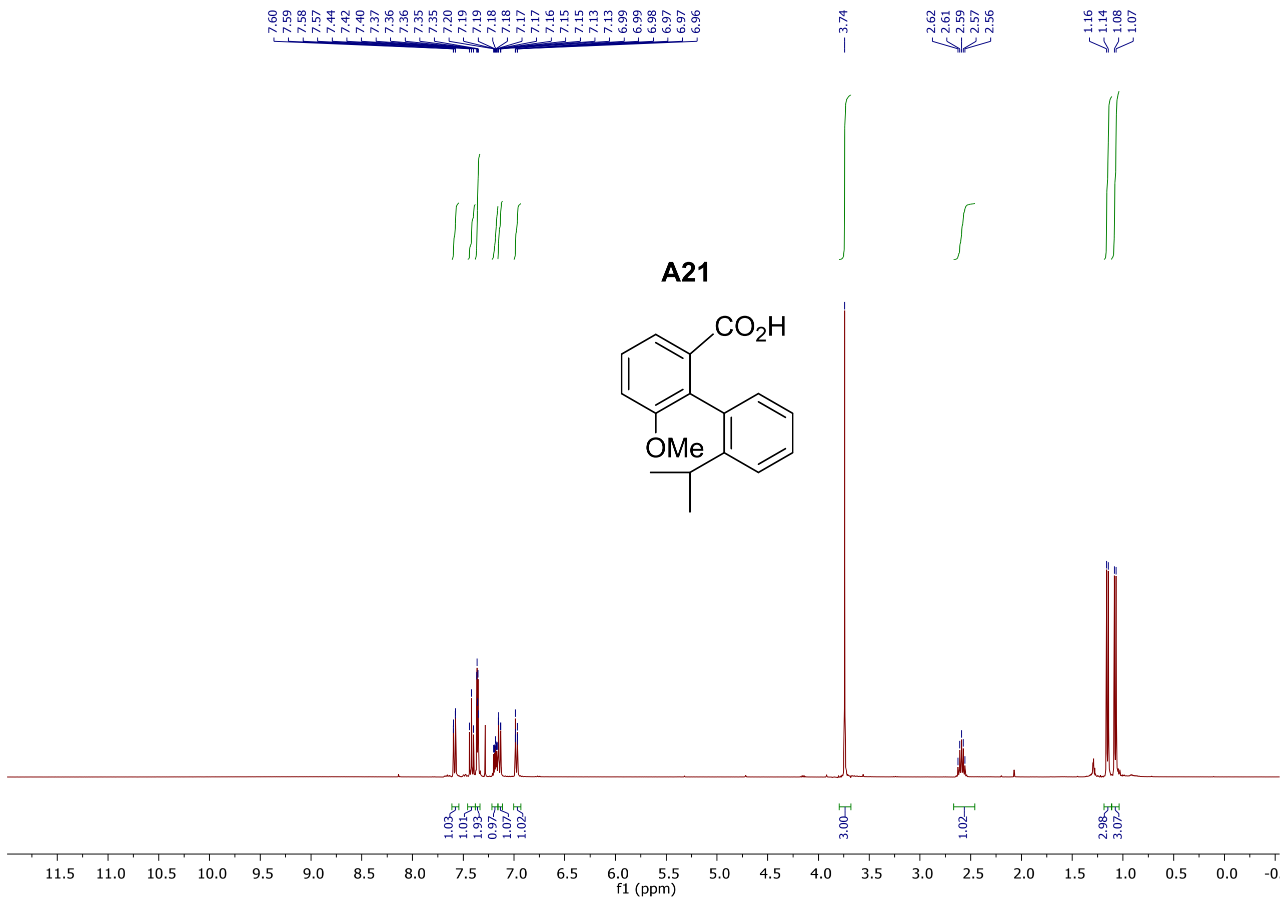
7.60 7.59 7.58 7.57 7.44 7.42 7.40 7.37 7.36 7.36 7.35 7.35 7.20 7.19 7.19 7.18 7.18 7.17 7.17 7.16 7.15 7.15 7.13 6.99 6.99 6.98 6.97 6.97 6.96
3.74
2.62 2.61 2.59 2.57 2.56
1.16 1.14 1.08 1.07
A21
CO_2H
OMe
1.03 1.01 1.93 0.97 1.07 1.02
3.00
1.02
2.98 3.07
11.5 11.0 10.5 10.0 9.5 9.0 8.5 8.0 7.5 7.0 6.5 6.0 5.5 5.0 4.5 4.0 3.5 3.0 2.5 2.0 1.5 1.0 0.5 0.0
f1 (ppm)

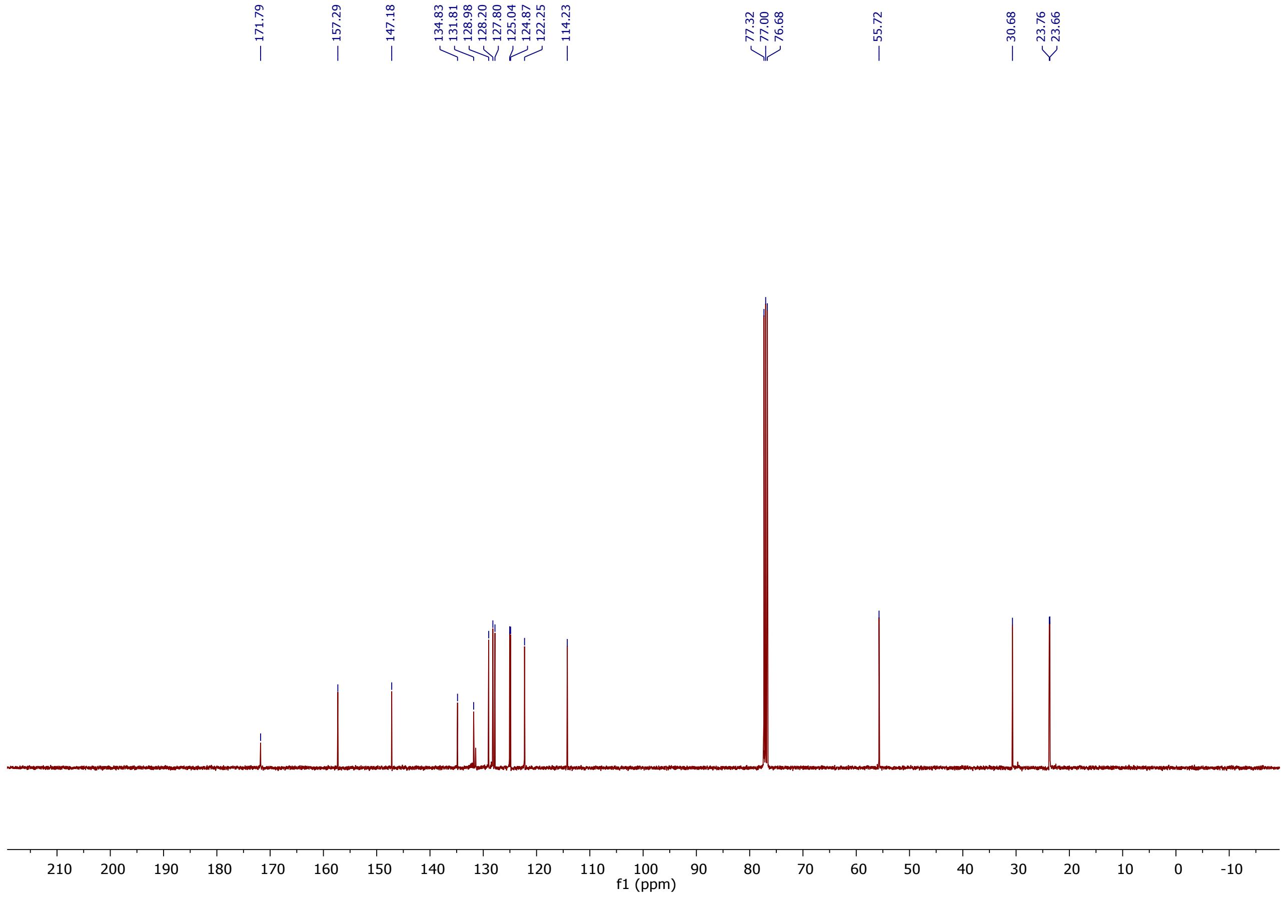
171.79
157.29
147.18
134.83 131.81 128.98 128.20 127.80 125.04 124.87 122.25
114.23
77.32 77.00 76.68
55.72
30.68
23.76 23.66
210 200 190 180 170 160 150 140 130 120 110 100 90 80 70 60 50 40 30 20 10 0 -10
f1 (ppm)

19. Spectra of all products

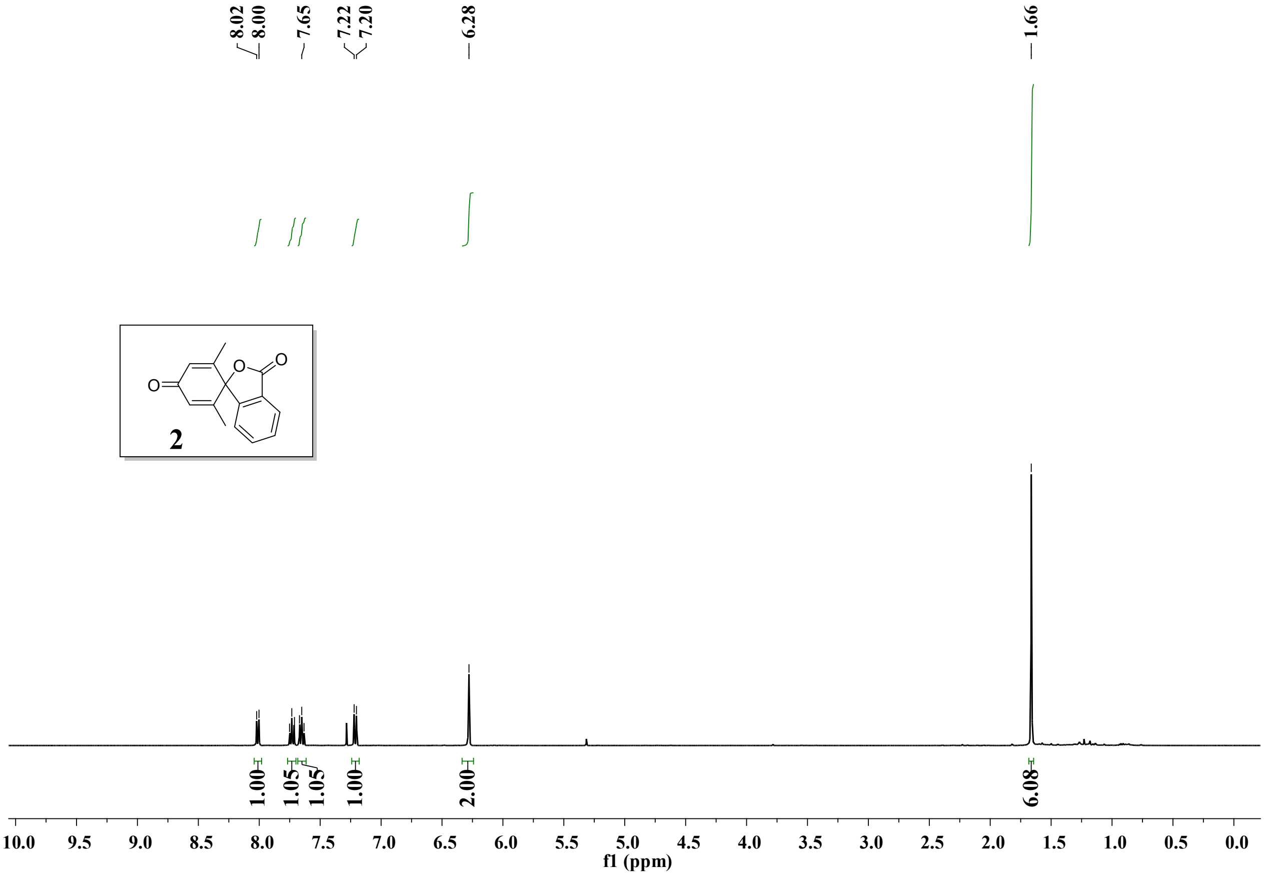

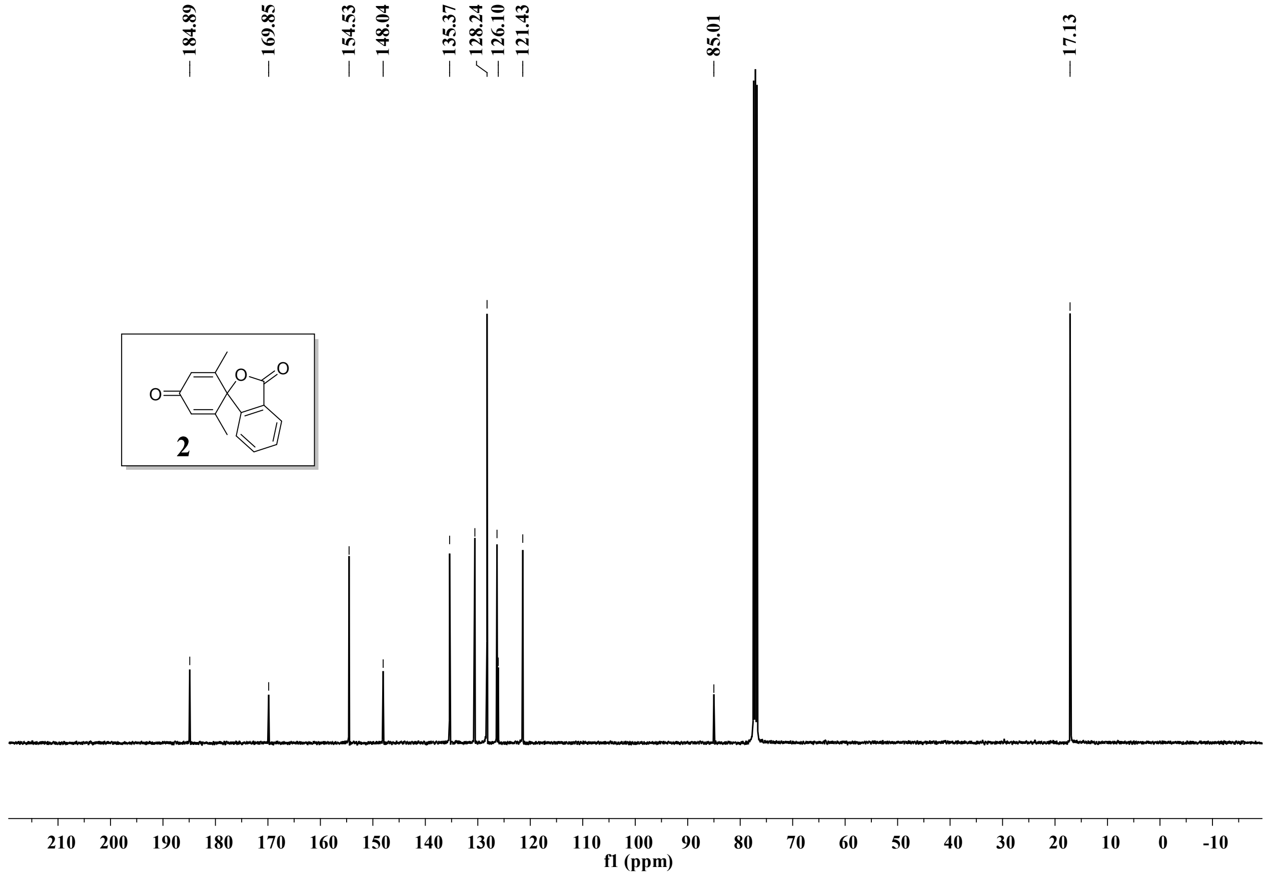

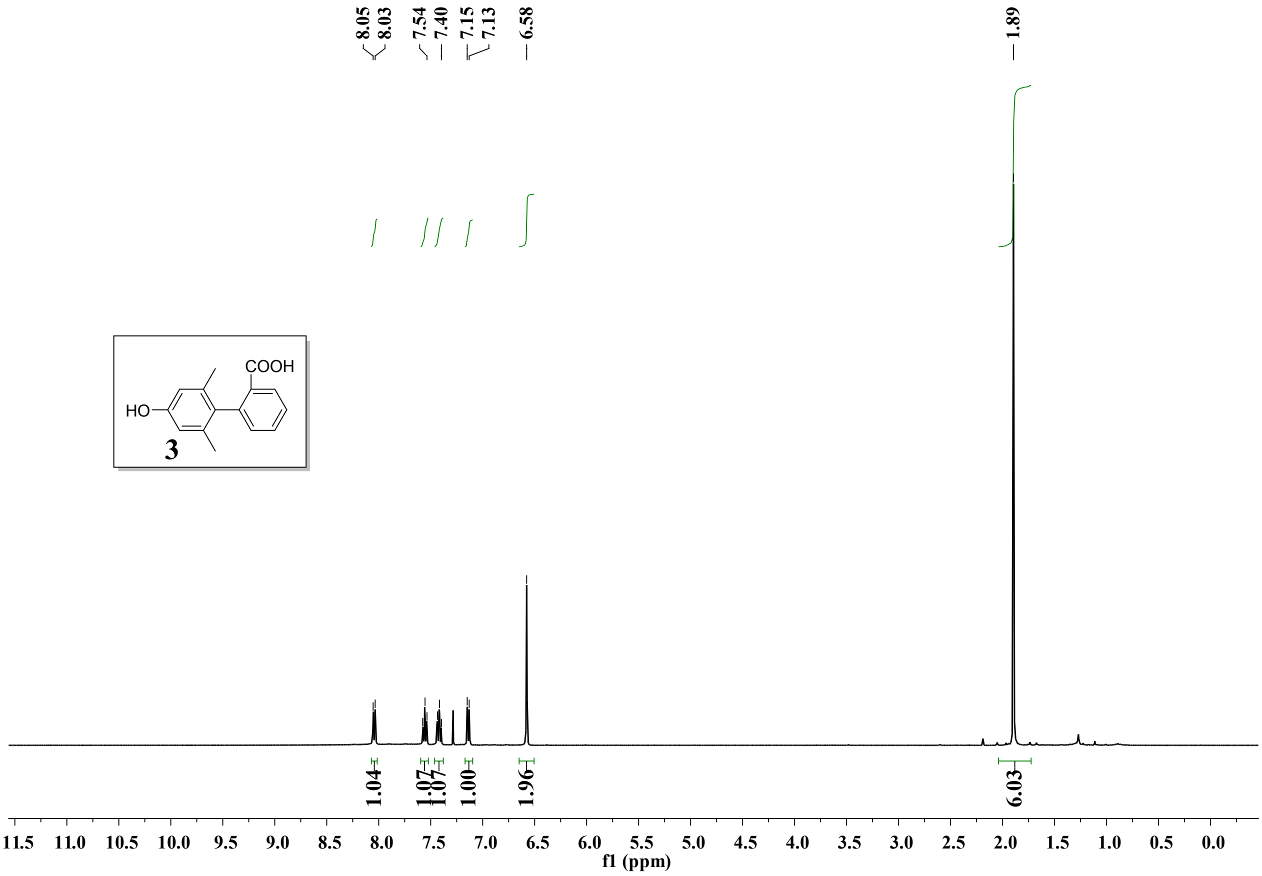
8.05
8.03
7.54
7.40
7.15
7.13
6.58
1.89
COOH
HO
3
1.04
1.07
1.07
1.00
1.96
6.03
11.5 11.0 10.5 10.0 9.5 9.0 8.5 8.0 7.5 7.0 6.5 6.0 5.5 5.0 4.5 4.0 3.5 3.0 2.5 2.0 1.5 1.0 0.5 0.0
f1 (ppm)

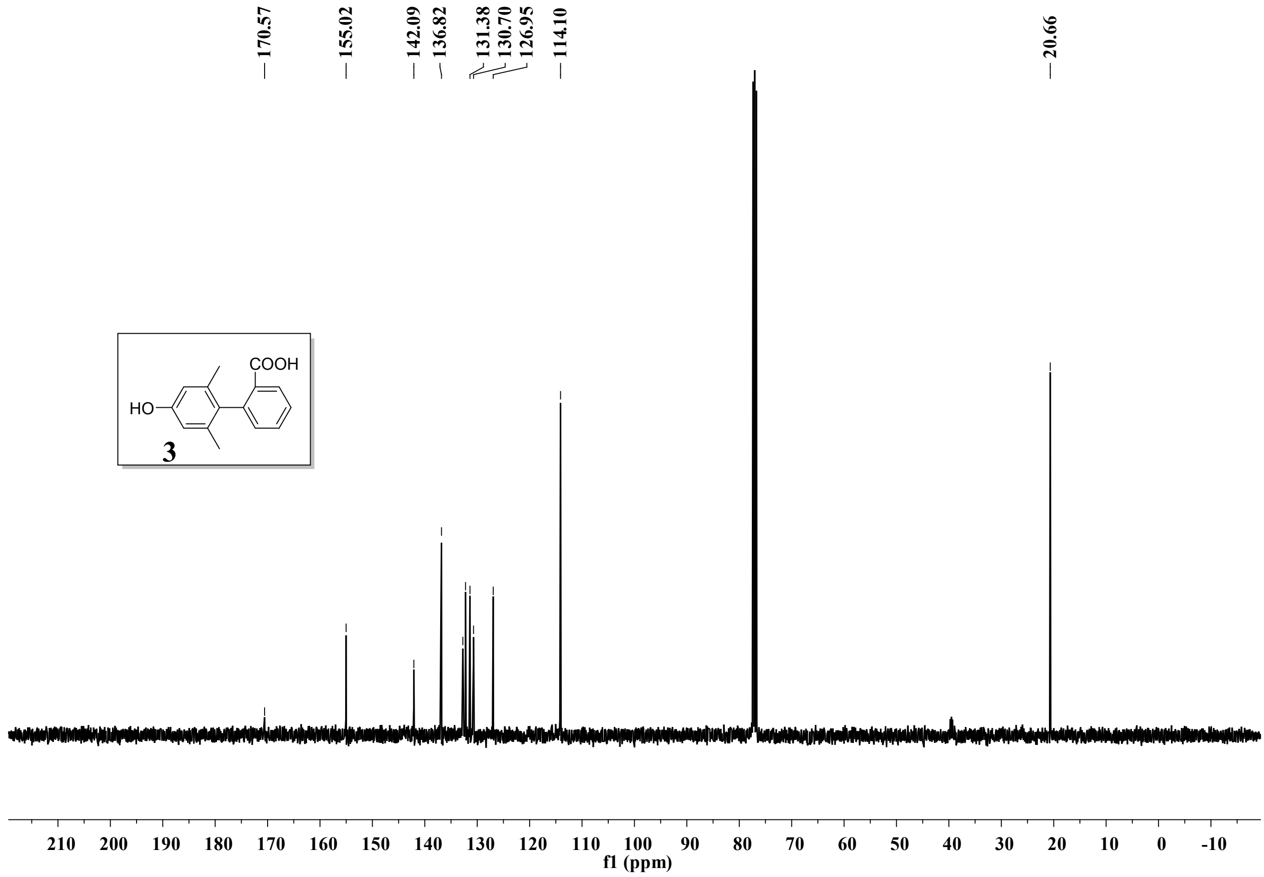
170.57
155.02
142.09
136.82
131.38
130.70
126.95
114.10
20.66
COOH
HO
3
210 200 190 180 170 160 150 140 130 120 110 100 90 80 70 60 50 40 30 20 10 0 -10
f1 (ppm)

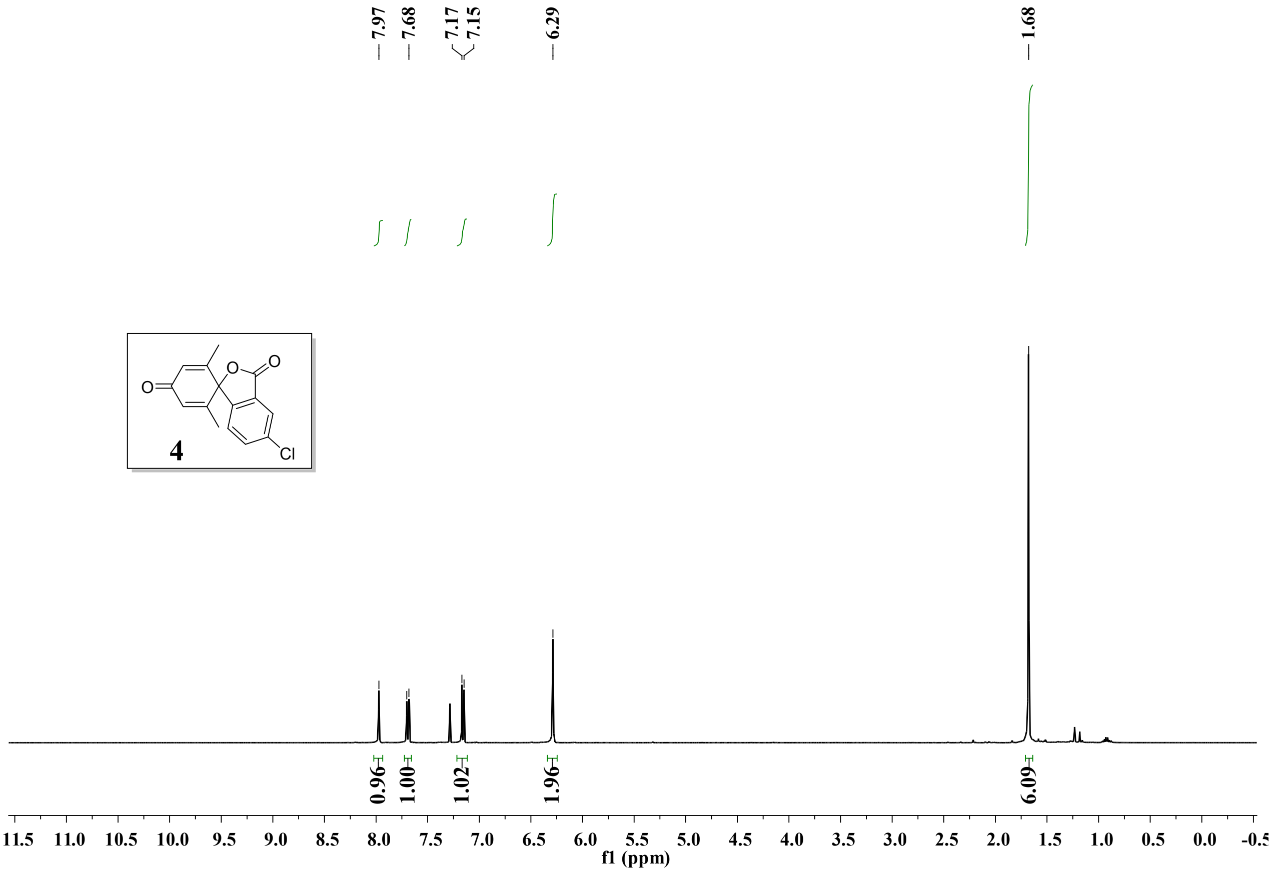
7.97
7.68
7.17
7.15
6.29
1.68
0.96
1.00
1.02
1.96
6.09
4
O
O
O
Cl
11.5 11.0 10.5 10.0 9.5 9.0 8.5 8.0 7.5 7.0 6.5 6.0 5.5 5.0 4.5 4.0 3.5 3.0 2.5 2.0 1.5 1.0 0.5 0.0
f1 (ppm)

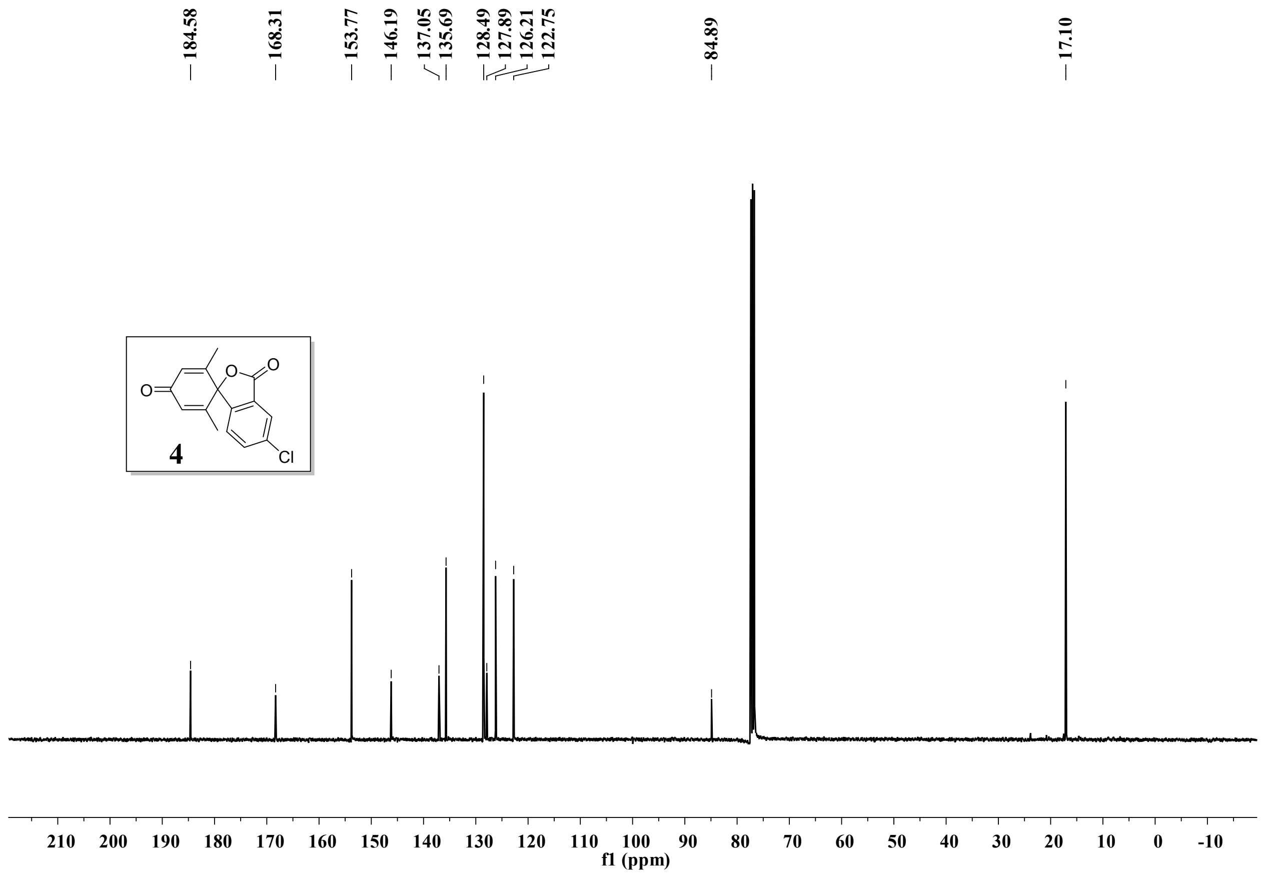
184.58
168.31
153.77
146.19
137.05
135.69
128.49
127.89
126.21
122.75
84.89
17.10
4
O
O
O
Cl
210 200 190 180 170 160 150 140 130 120 110 100 90 80 70 60 50 40 30 20 10 0 -10
f1 (ppm)

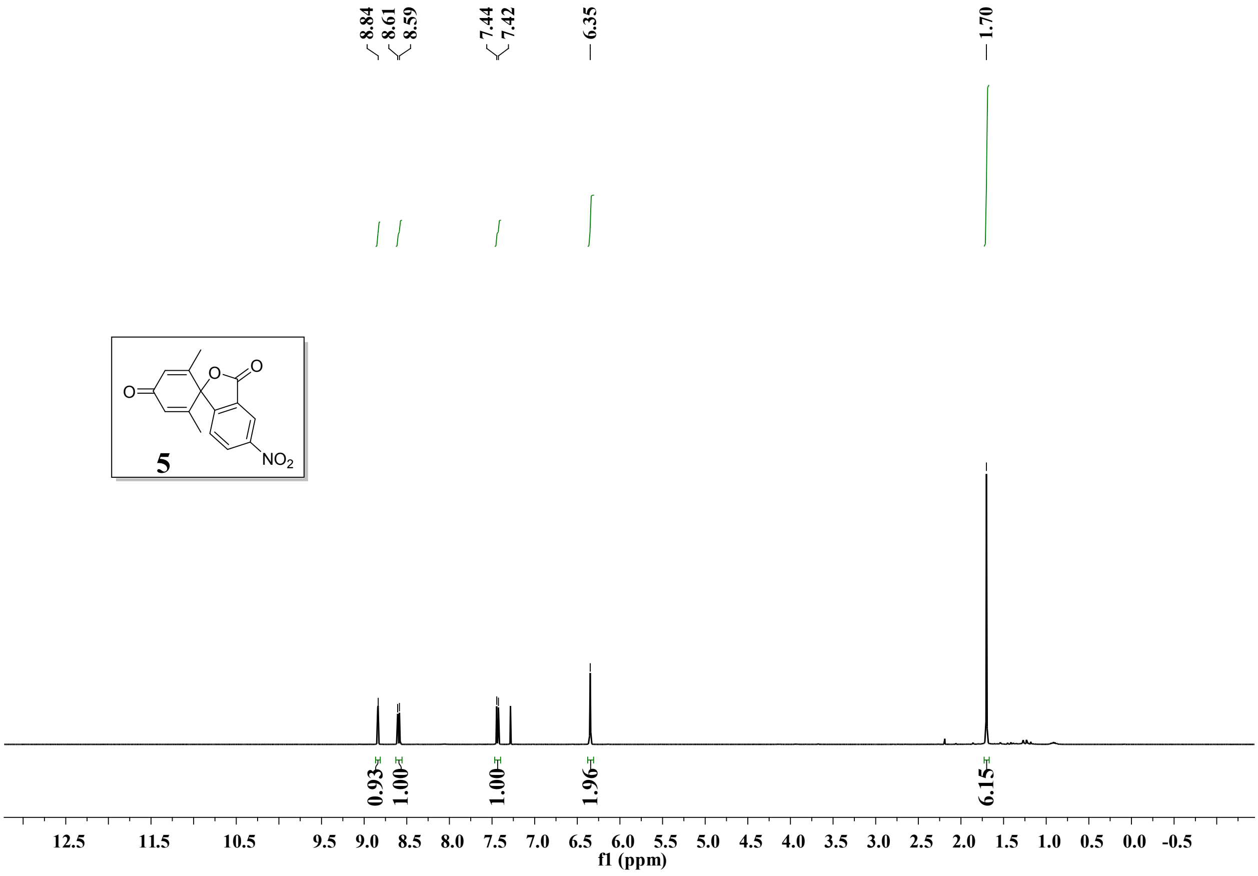
8.84
8.61
8.59
7.44
7.42
6.35
1.70
5
NO_2
0.93
1.00
1.00
1.96
6.15
12.5 11.5 10.5 9.5 9.0 8.5 8.0 7.5 7.0 6.5 6.0 5.5 5.0 4.5 4.0 3.5 3.0 2.5 2.0 1.5 1.0 0.5 0.0 -0.5
f1 (ppm)

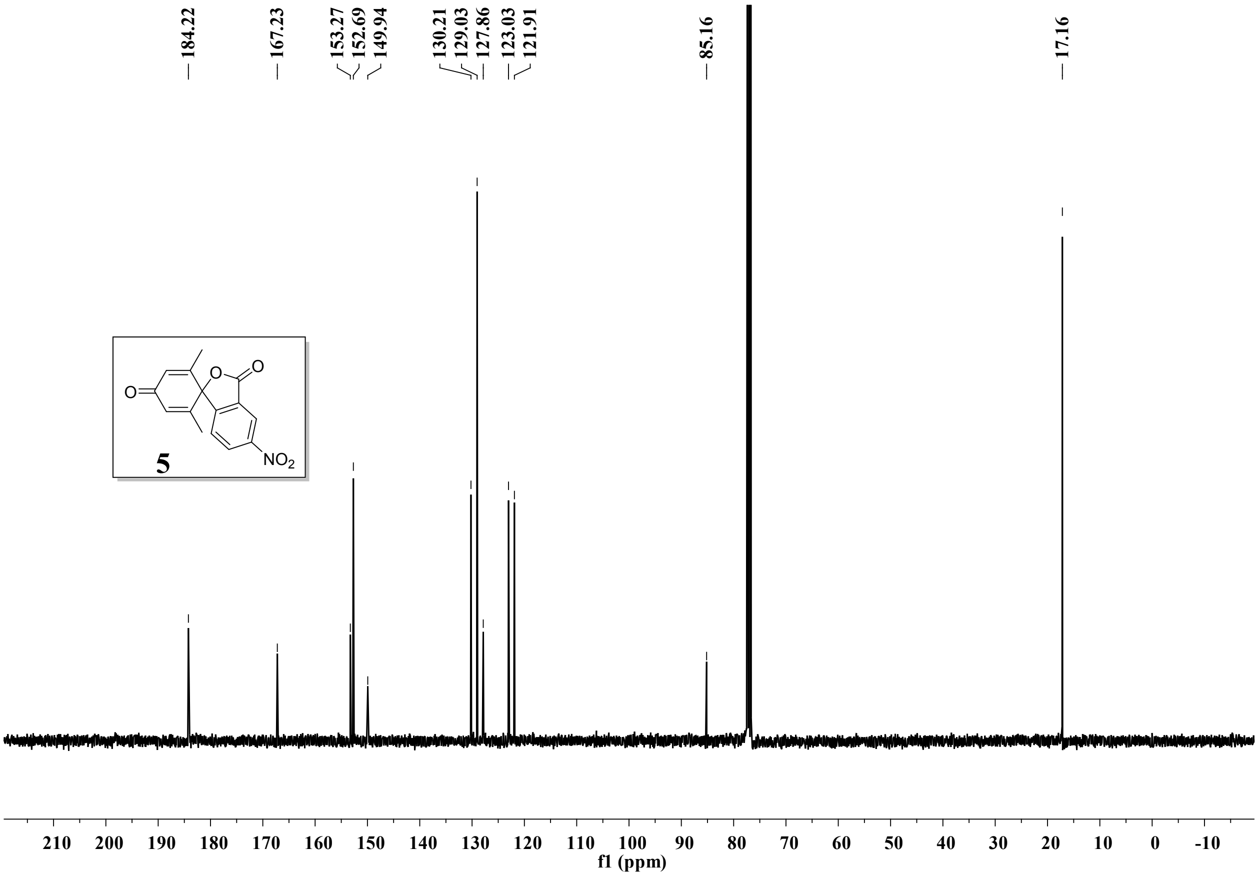
184.22
167.23
153.27
152.69
149.94
130.21
129.03
127.86
123.03
121.91
85.16
17.16
5
NO_2
210 200 190 180 170 160 150 140 130 120 110 100 90 80 70 60 50 40 30 20 10 0 -10
f1 (ppm)

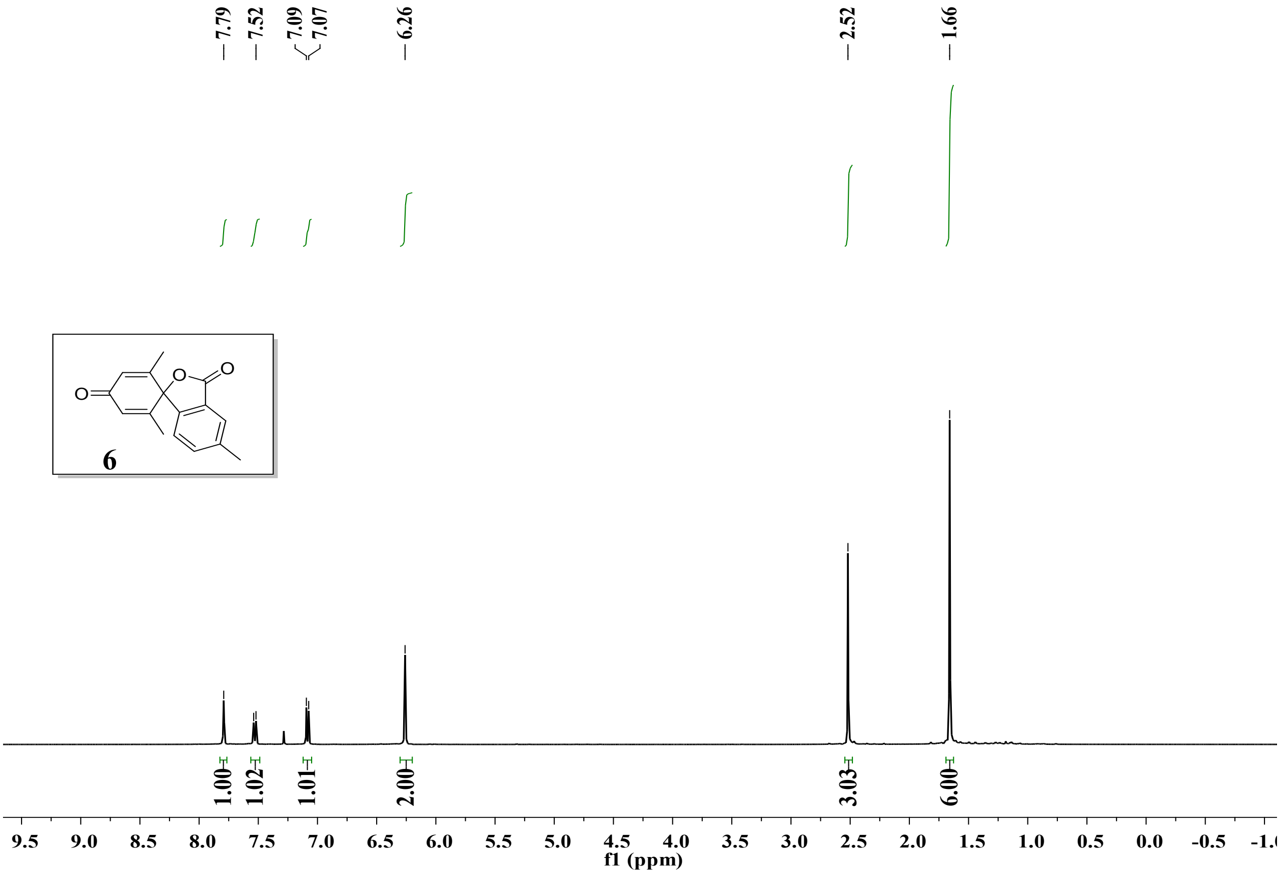
7.79
7.52
7.09
7.07
6.26
2.52
1.66
6
1.00
1.02
1.01
2.00
3.03
6.00
9.5 9.0 8.5 8.0 7.5 7.0 6.5 6.0 5.5 5.0 4.5 4.0 3.5 3.0 2.5 2.0 1.5 1.0 0.5 0.0 -0.5
f1 (ppm)

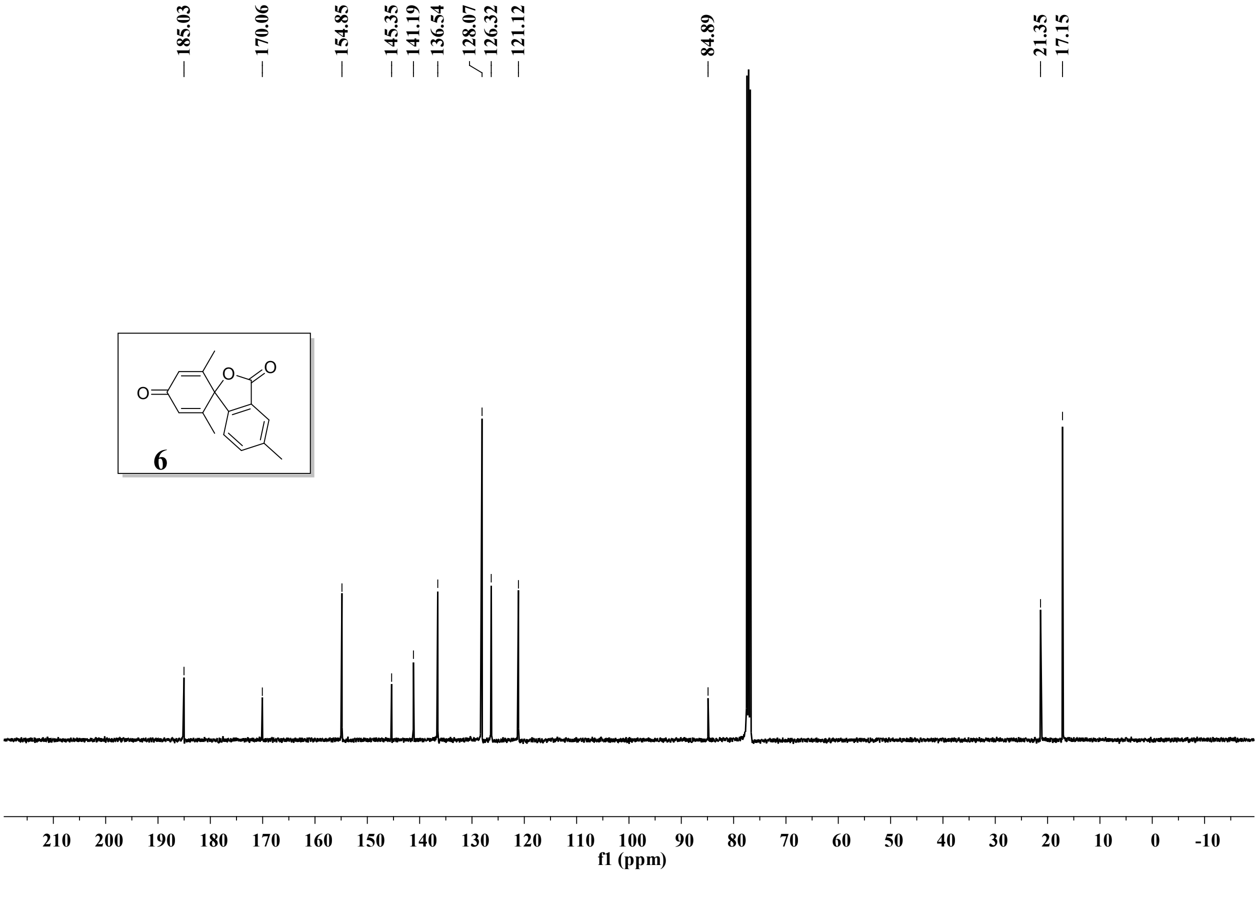
185.03
170.06
154.85
145.35
141.19
136.54
128.07
126.32
121.12
84.89
21.35
17.15
6
210 200 190 180 170 160 150 140 130 120 110 100 90 80 70 60 50 40 30 20 10 0 -10
f1 (ppm)

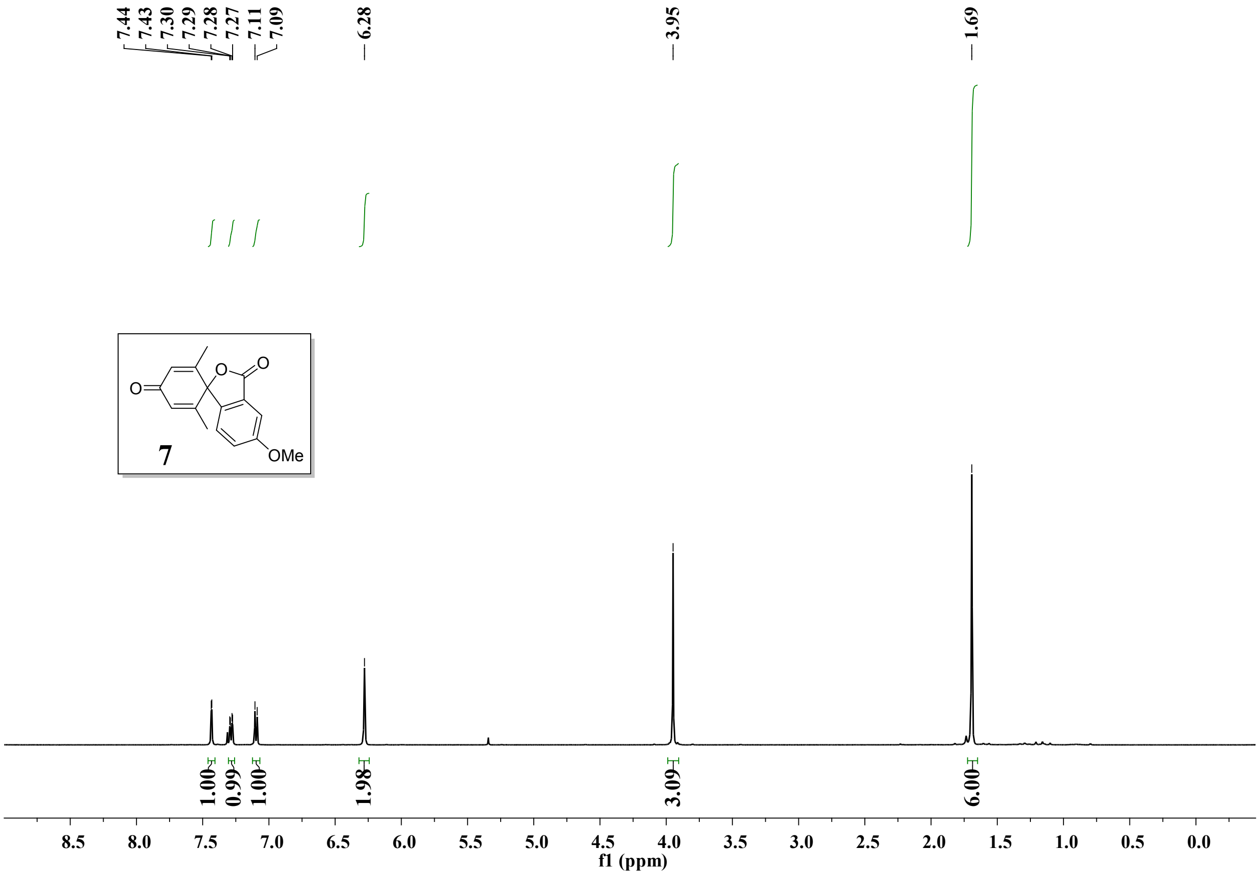
7.44
7.43
7.30
7.29
7.28
7.27
7.11
7.09
6.28
3.95
1.69
O
O
O
OMe
7
1.00
0.99
1.00
1.98
3.09
6.00
8.5 8.0 7.5 7.0 6.5 6.0 5.5 5.0 4.5 4.0 3.5 3.0 2.5 2.0 1.5 1.0 0.5 0.0
f1 (ppm)

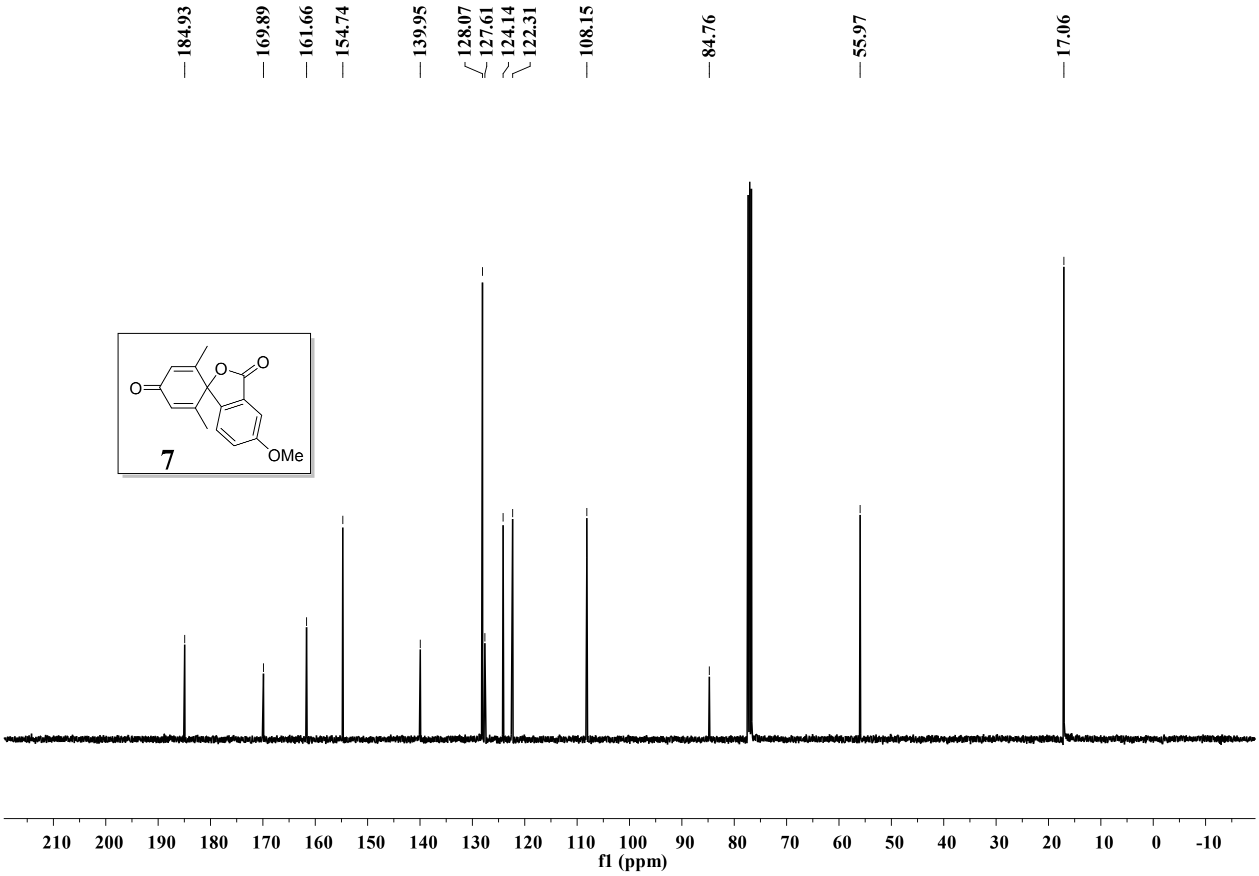
184.93
169.89
161.66
154.74
139.95
128.07
127.61
124.14
122.31
108.15
84.76
55.97
17.06
O
O
O
OMe
7
210 200 190 180 170 160 150 140 130 120 110 100 90 80 70 60 50 40 30 20 10 0 -10
f1 (ppm)

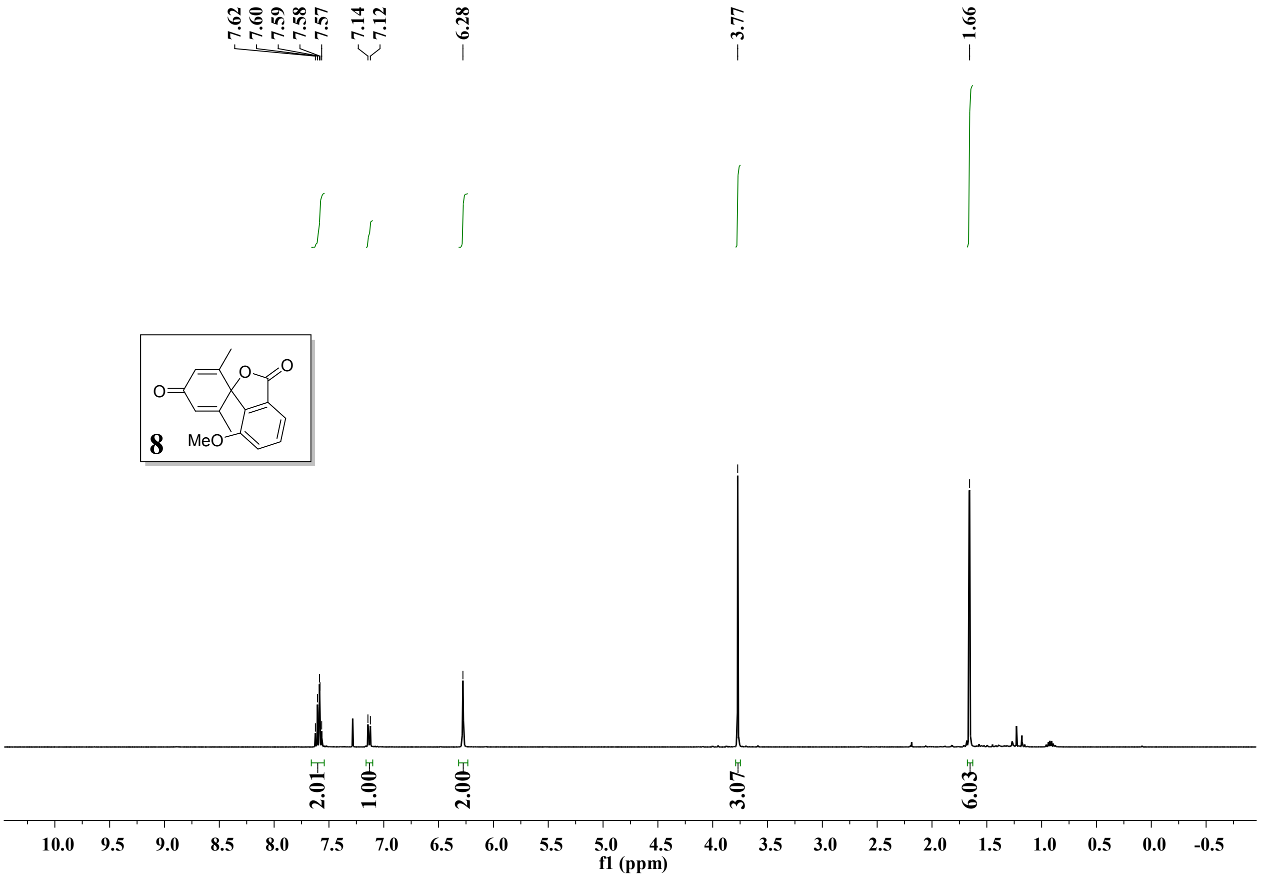
7.62
7.60
7.59
7.58
7.57
7.14
7.12
6.28
3.77
1.66
8
MeO
O
2.01
1.00
2.00
3.07
6.03
10.0 9.5 9.0 8.5 8.0 7.5 7.0 6.5 6.0 5.5 5.0 4.5 4.0 3.5 3.0 2.5 2.0 1.5 1.0 0.5 0.0 -0.5
f1 (ppm)

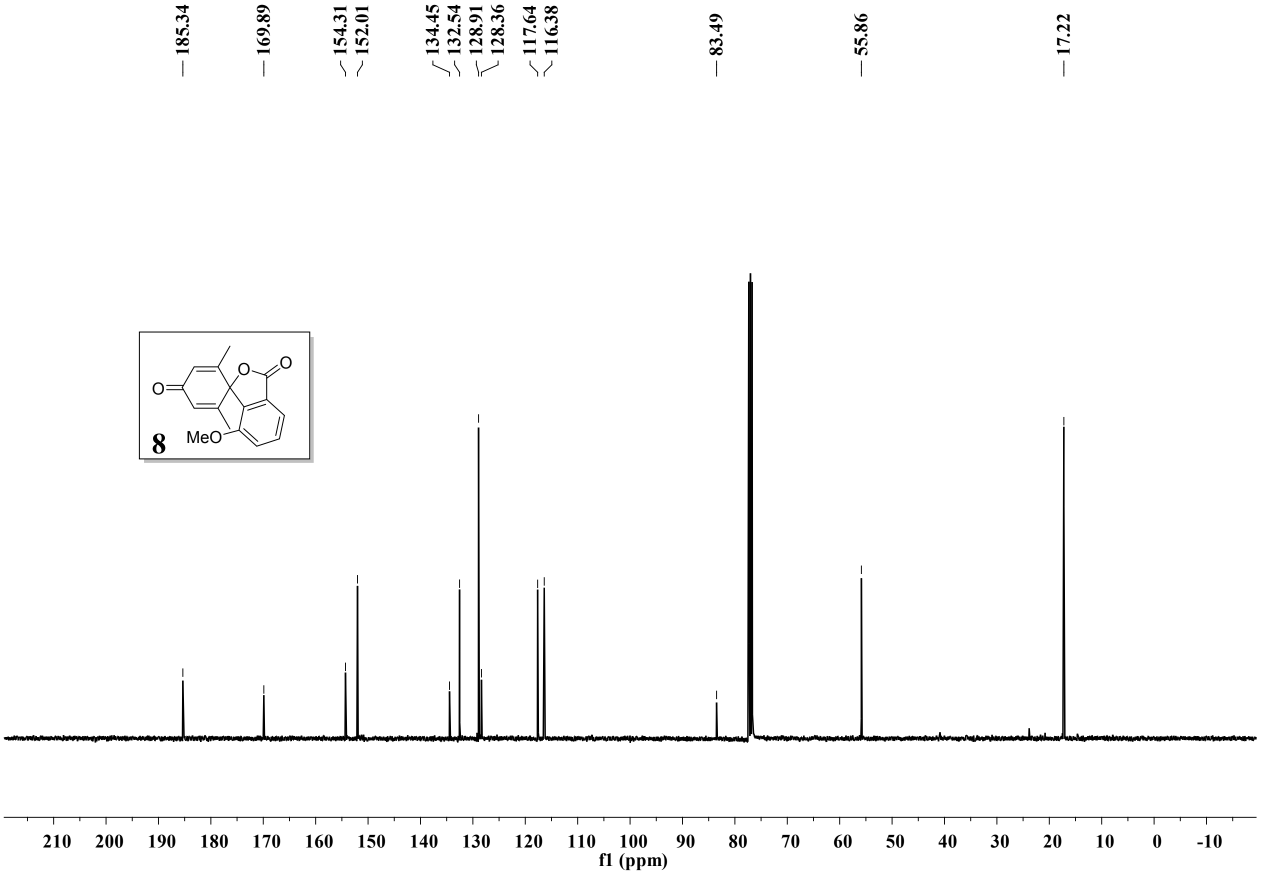
185.34
169.89
154.31
152.01
134.45
132.54
128.91
128.36
117.64
116.38
83.49
55.86
17.22
8
MeO
O
210 200 190 180 170 160 150 140 130 120 110 100 90 80 70 60 50 40 30 20 10 0 -10
f1 (ppm)

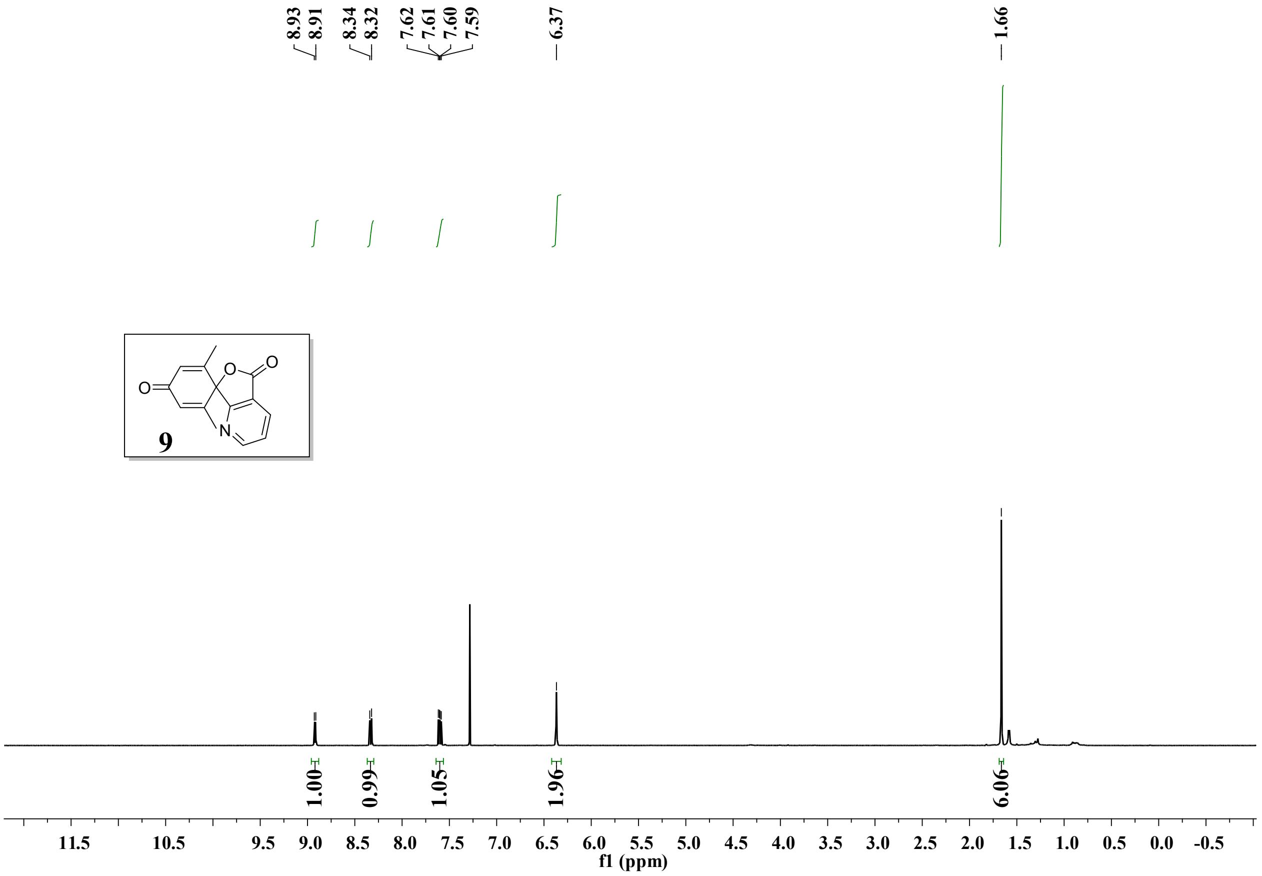

8.93
8.91
8.34
8.32
7.62
7.61
7.60
7.59
6.37
1.66
9
1.00
0.99
1.05
1.96
6.06
11.5 10.5 9.5 9.0 8.5 8.0 7.5 7.0 6.5 6.0 5.5 5.0 4.5 4.0 3.5 3.0 2.5 2.0 1.5 1.0 0.5 0.0 -0.5
f1 (ppm)

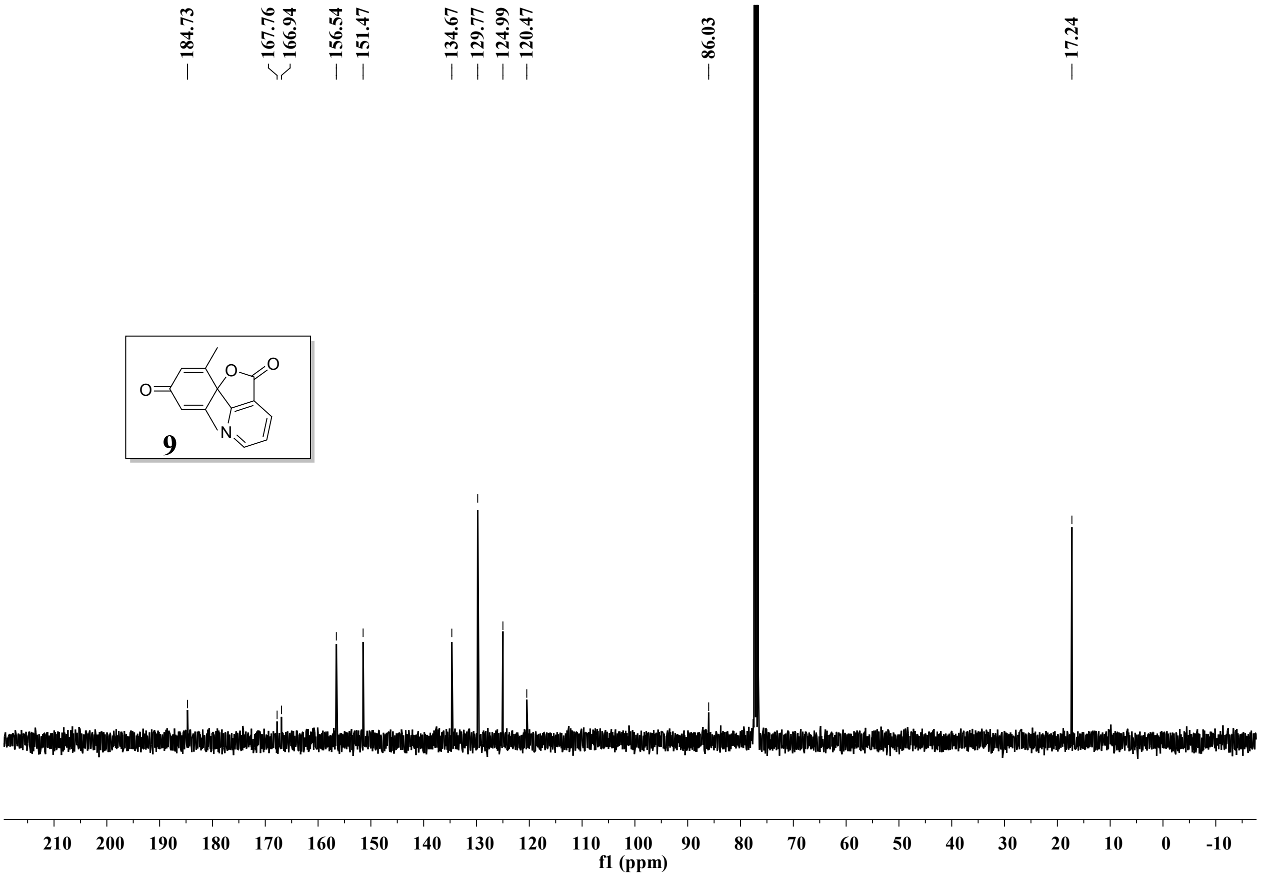

184.73
167.76
166.94
156.54
151.47
134.67
129.77
124.99
120.47
86.03
17.24
9
210 200 190 180 170 160 150 140 130 120 110 100 90 80 70 60 50 40 30 20 10 0 -10
f1 (ppm)

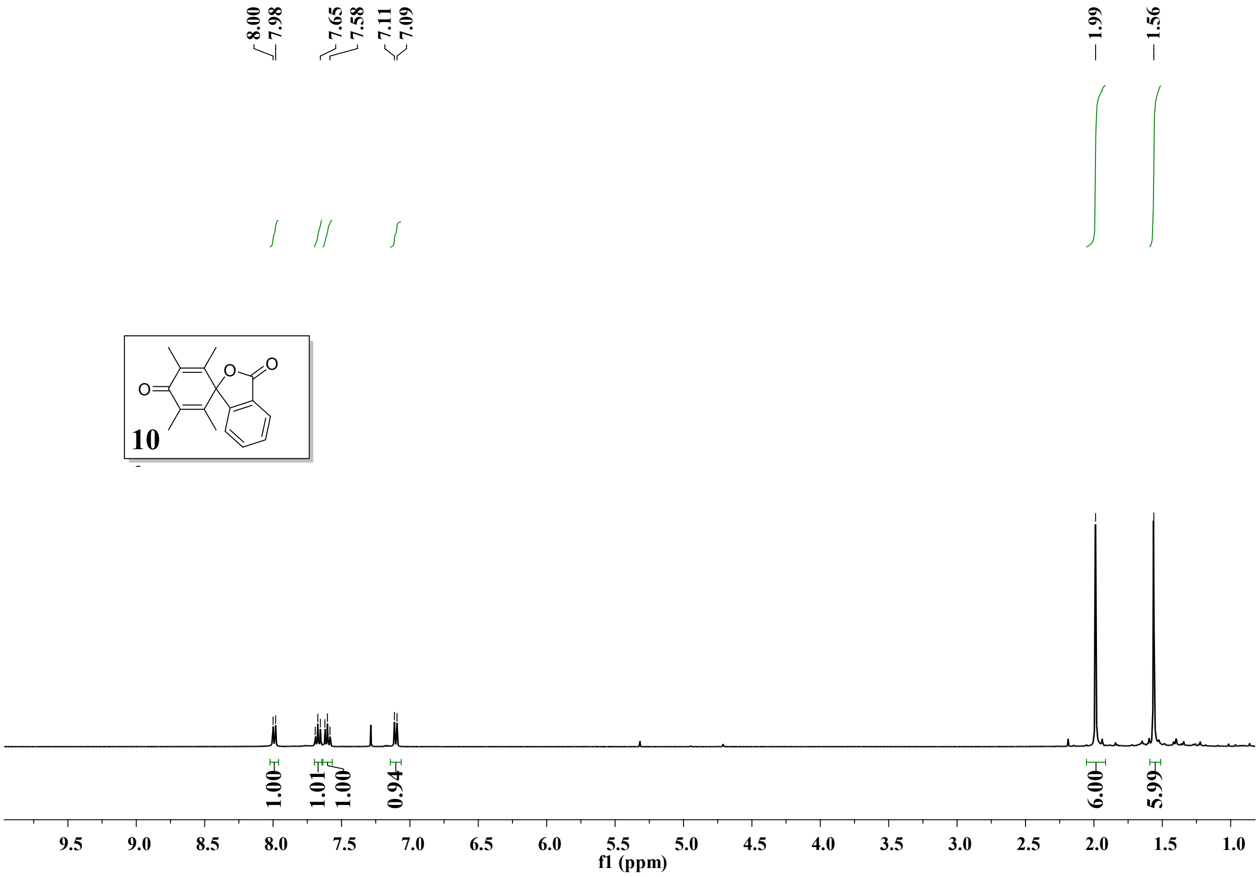
8.00
7.98
7.65
7.58
7.11
7.09
1.99
1.56
10
1.00
1.01
1.00
0.94
6.00
5.99
9.5 9.0 8.5 8.0 7.5 7.0 6.5 6.0 5.5 5.0 4.5 4.0 3.5 3.0 2.5 2.0 1.5 1.0
f1 (ppm)

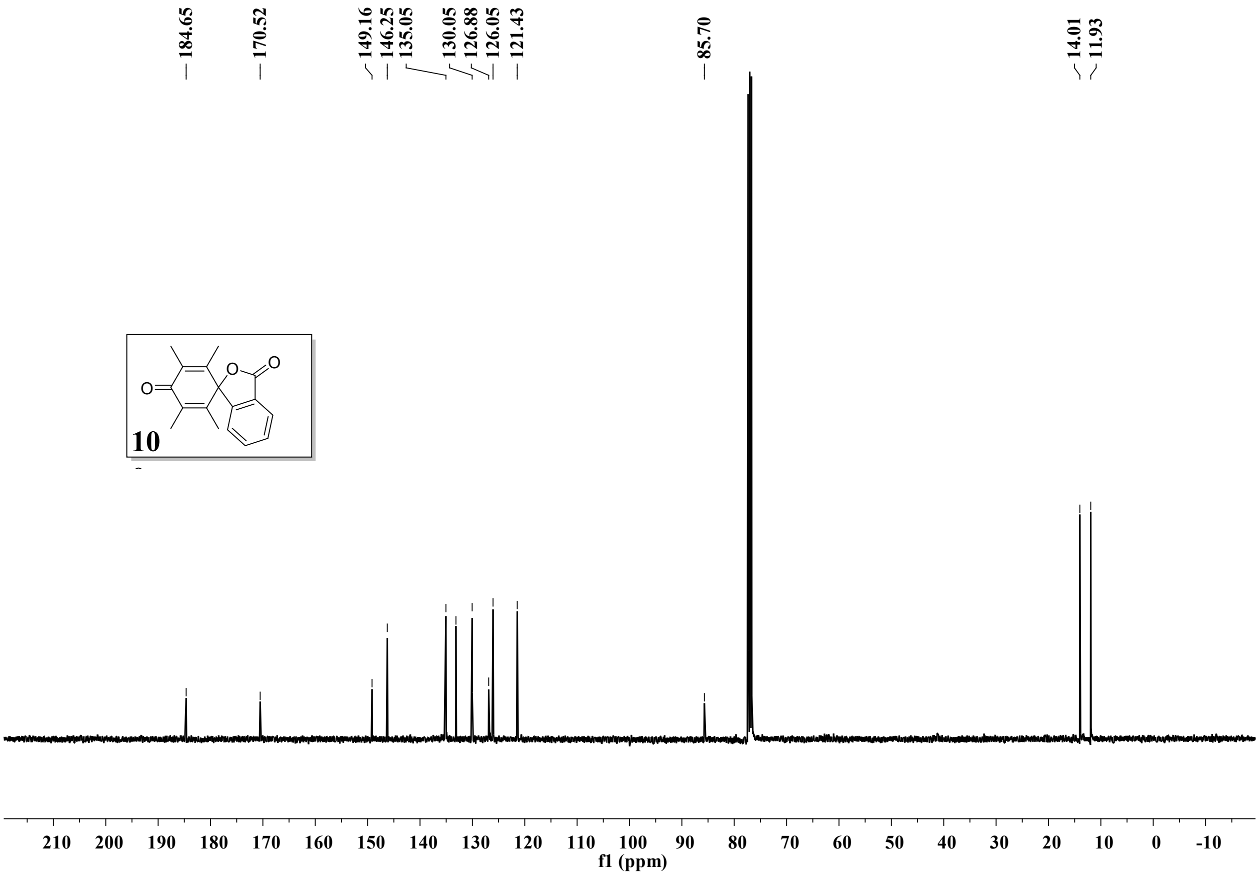
184.65
170.52
149.16
146.25
135.05
130.05
126.88
126.05
121.43
85.70
14.01
11.93
10
210 200 190 180 170 160 150 140 130 120 110 100 90 80 70 60 50 40 30 20 10 0 -10
f1 (ppm)

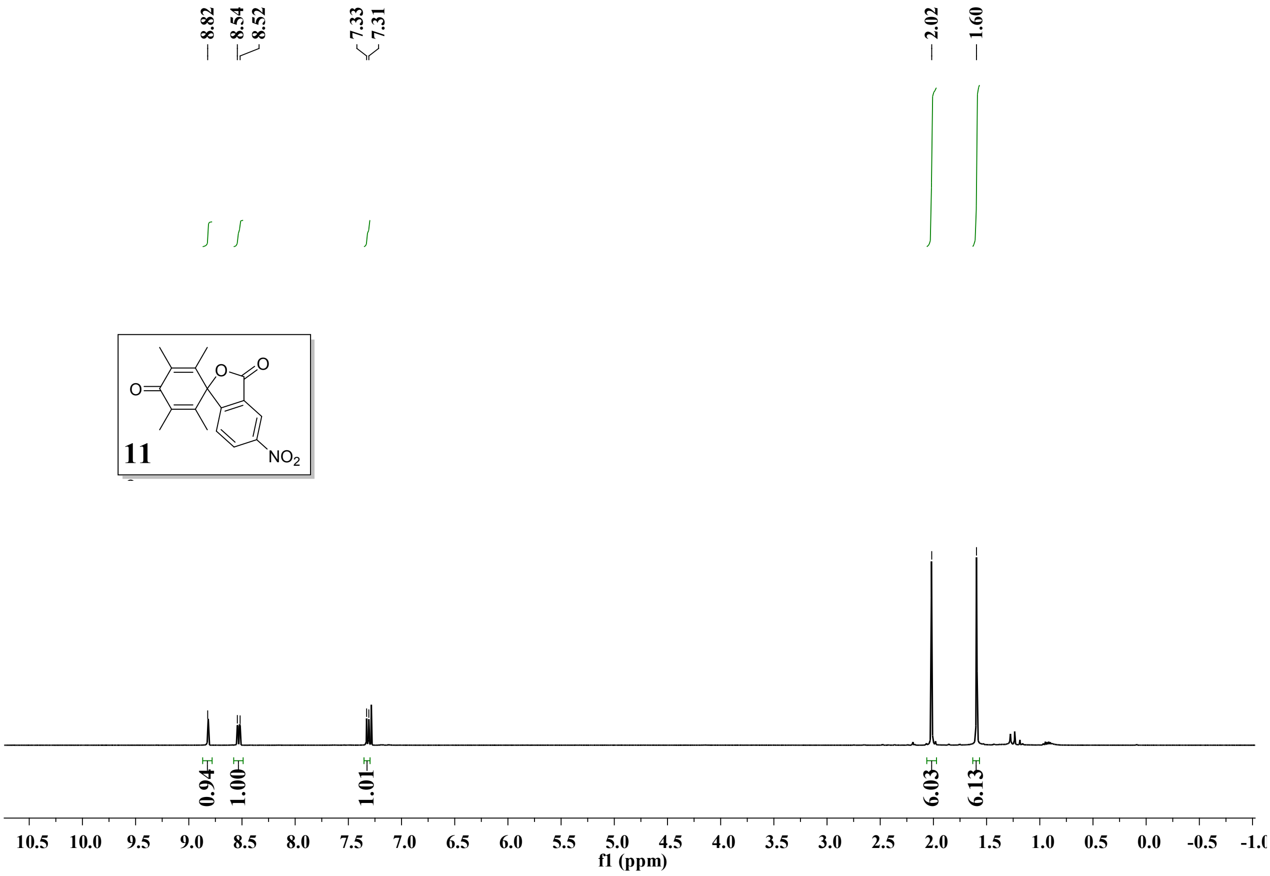

8.82
8.54
8.52
7.33
7.31
2.02
1.60
11
NO2
0.94
1.00
1.01
6.03
6.13
10.5 10.0 9.5 9.0 8.5 8.0 7.5 7.0 6.5 6.0 5.5 5.0 4.5 4.0 3.5 3.0 2.5 2.0 1.5 1.0 0.5 0.0 -0.5
f1 (ppm)

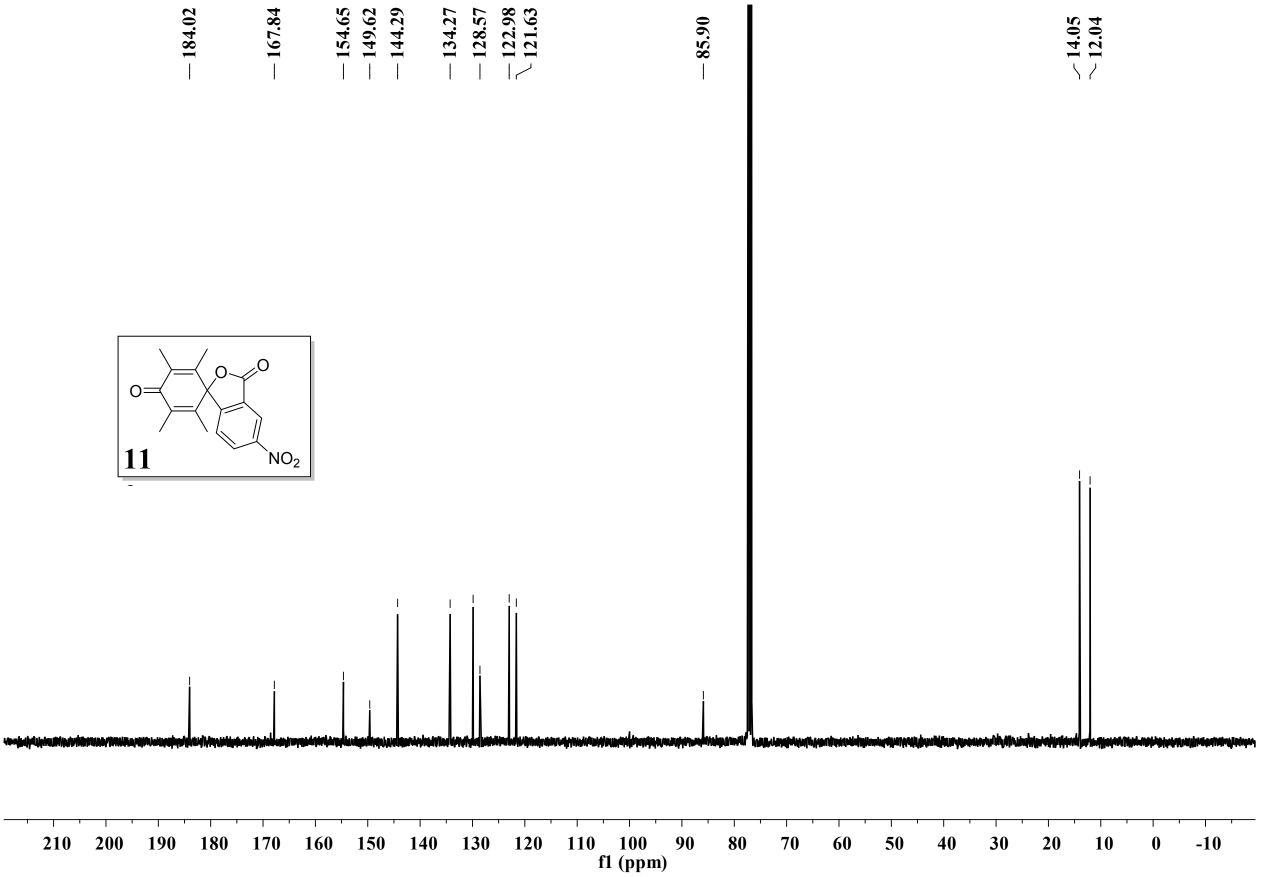

184.02
167.84
154.65
149.62
144.29
134.27
128.57
122.98
121.63
85.90
14.05
12.04
11
NO2
210 200 190 180 170 160 150 140 130 120 110 100 90 80 70 60 50 40 30 20 10 0 -10
f1 (ppm)

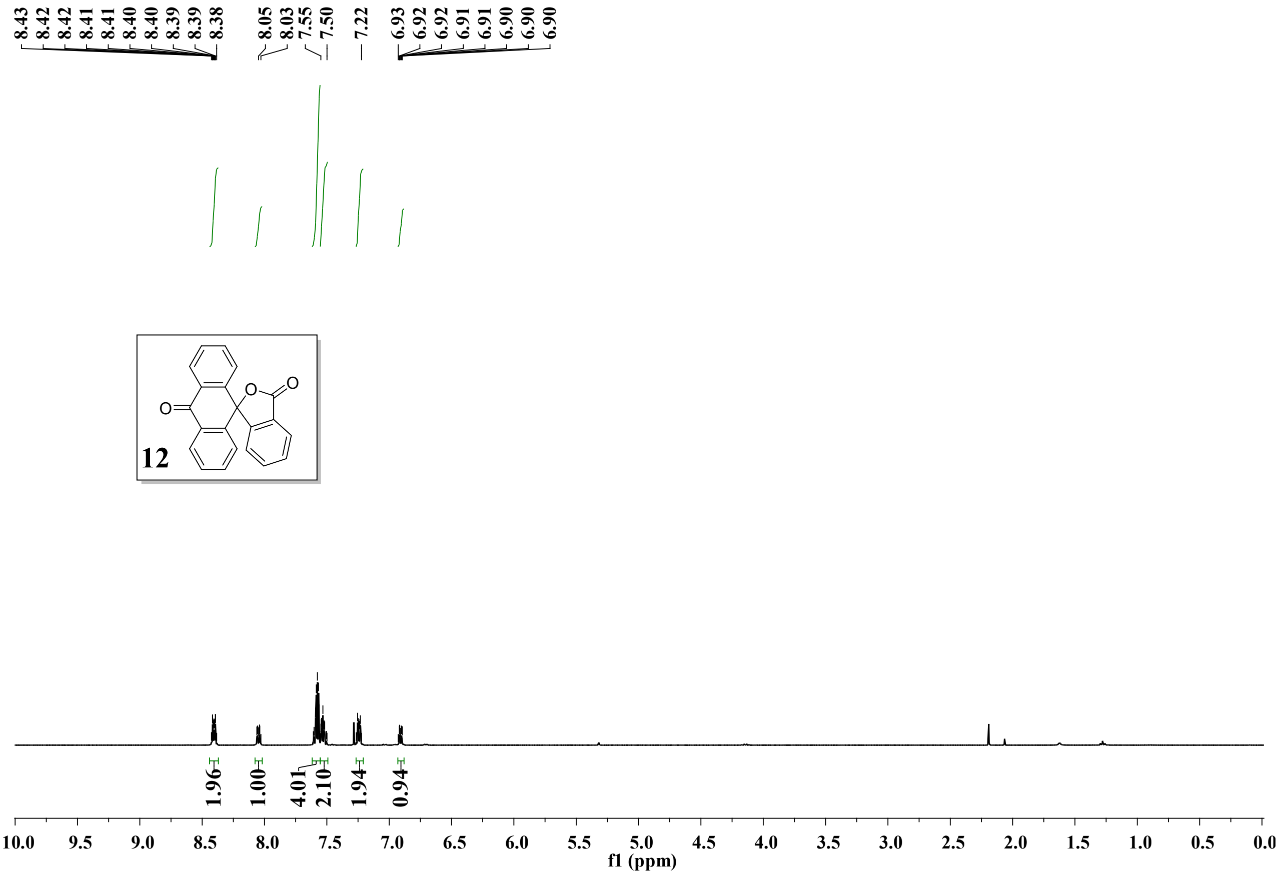
8.43
8.42
8.42
8.41
8.41
8.40
8.40
8.39
8.39
8.38
8.05
8.03
7.55
7.50
7.22
6.93
6.92
6.92
6.91
6.91
6.90
6.90
6.90
12
1.96
1.00
4.01
2.10
1.94
0.94
10.0 9.5 9.0 8.5 8.0 7.5 7.0 6.5 6.0 5.5 5.0 4.5 4.0 3.5 3.0 2.5 2.0 1.5 1.0 0.5 0.0
f1 (ppm)

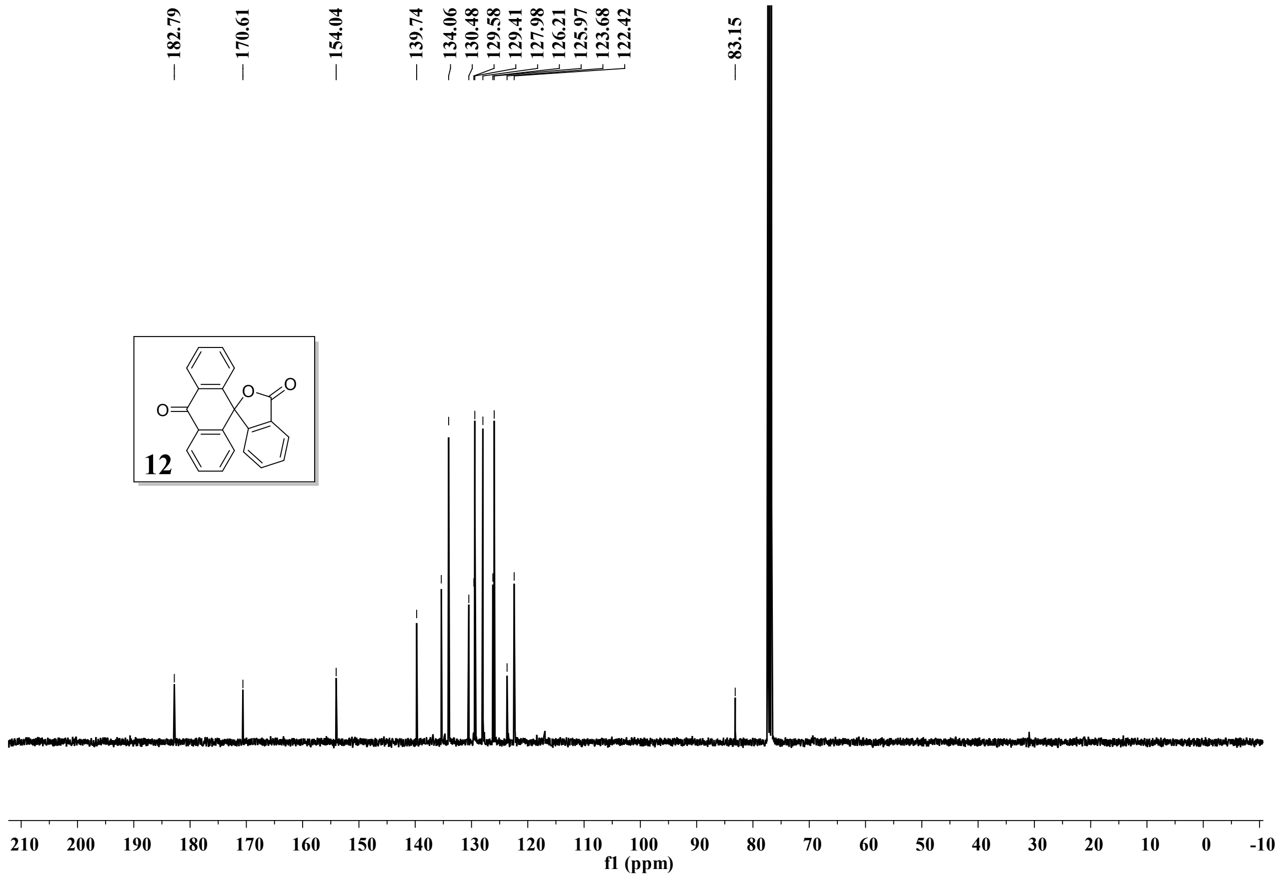
182.79
170.61
154.04
139.74
134.06
130.48
129.58
129.41
127.98
126.21
125.97
123.68
122.42
83.15
12
210 200 190 180 170 160 150 140 130 120 110 100 90 80 70 60 50 40 30 20 10 0 -10
f1 (ppm)

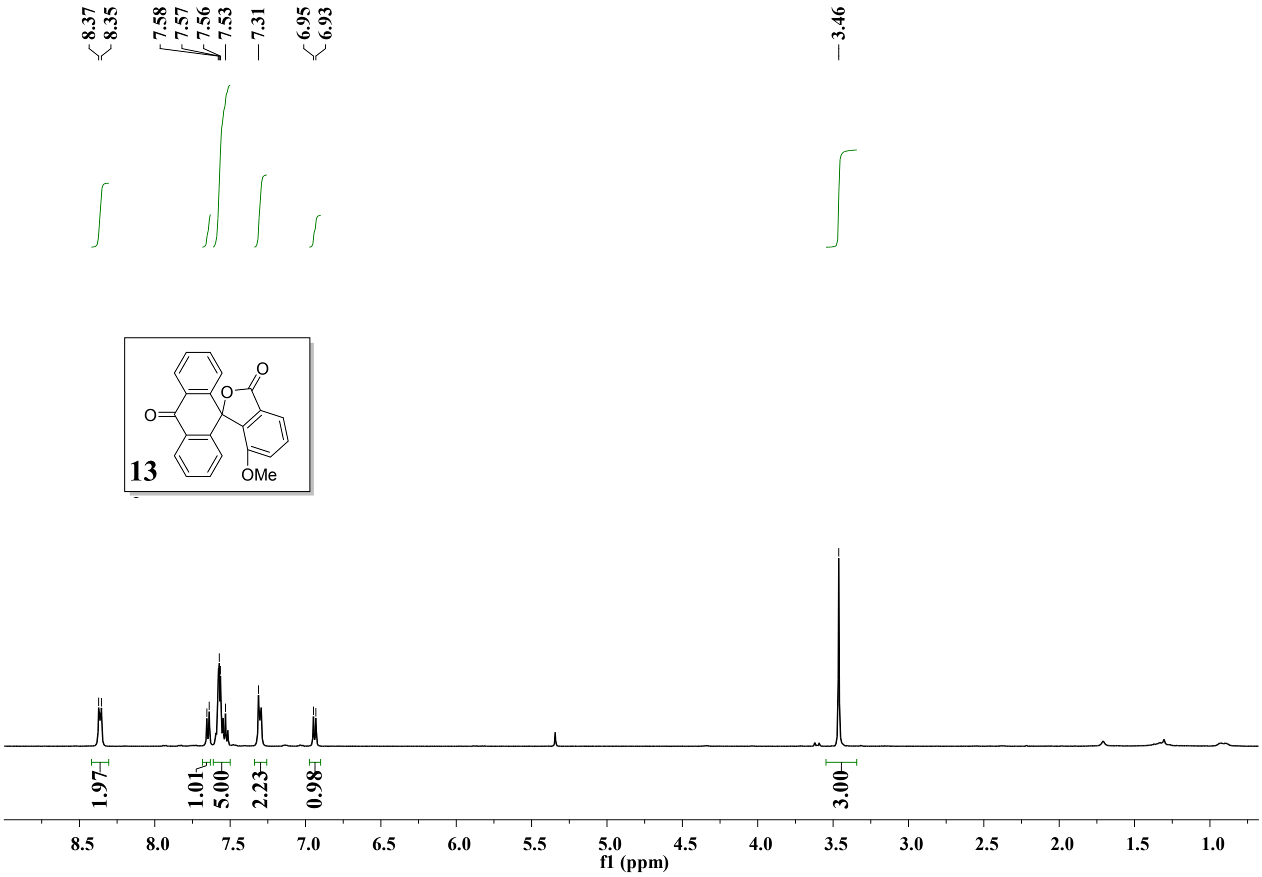
8.37
8.35
7.58
7.57
7.56
7.53
7.31
6.95
6.93
3.46
O
O
O
OMe
13
1.97
1.01
5.00
2.23
0.98
3.00
8.5 8.0 7.5 7.0 6.5 6.0 5.5 5.0 4.5 4.0 3.5 3.0 2.5 2.0 1.5 1.0
f1 (ppm)

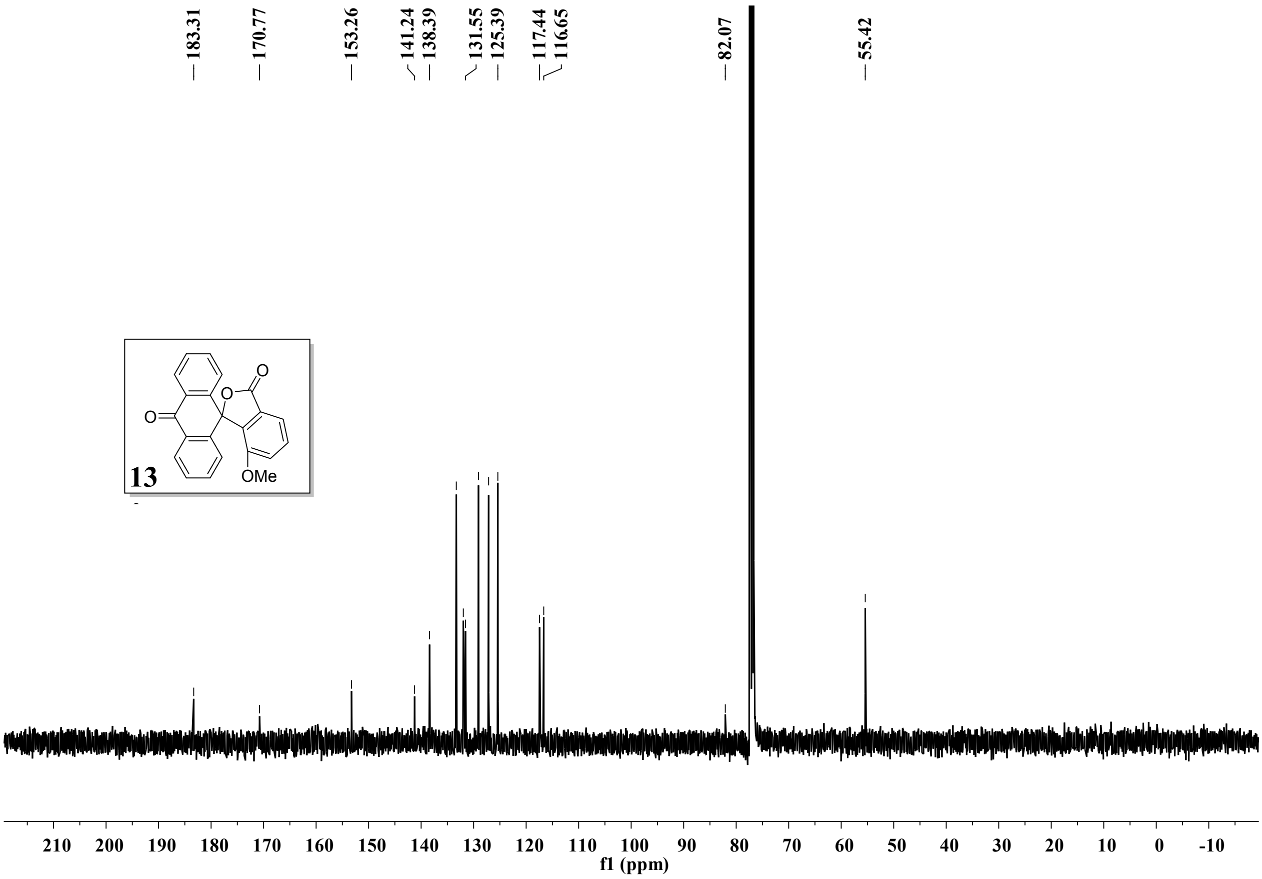
183.31
170.77
153.26
141.24
138.39
131.55
125.39
117.44
116.65
82.07
55.42
O
O
O
OMe
13
210 200 190 180 170 160 150 140 130 120 110 100 90 80 70 60 50 40 30 20 10 0 -10
f1 (ppm)

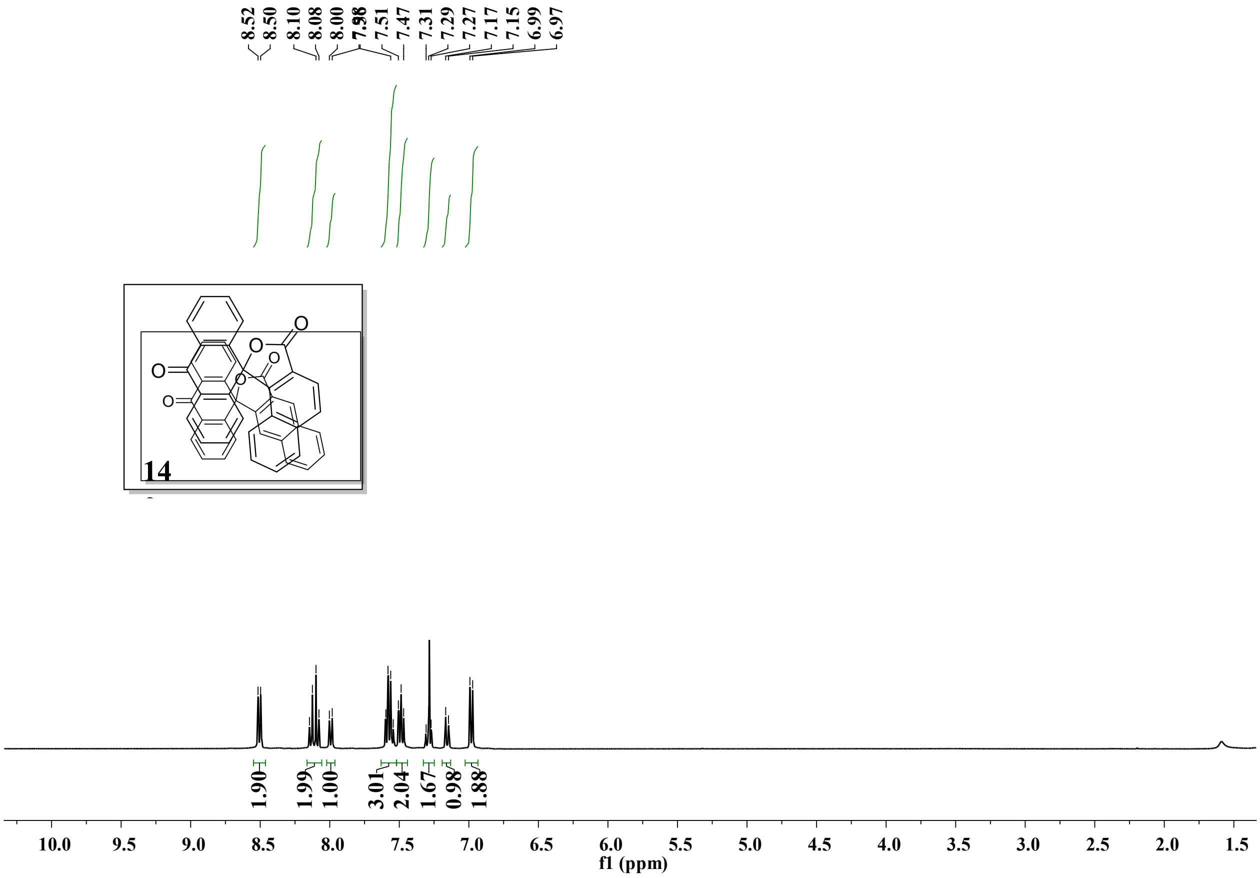

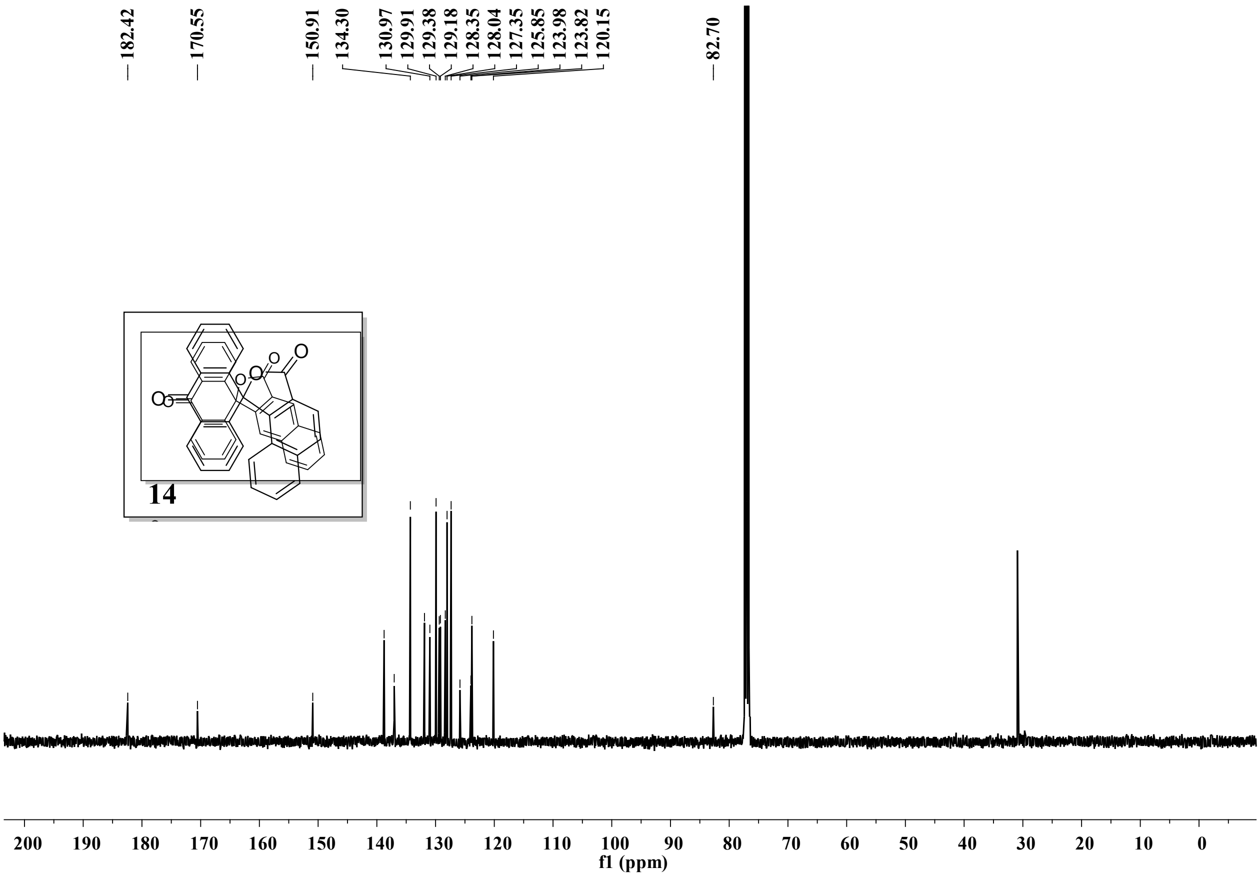

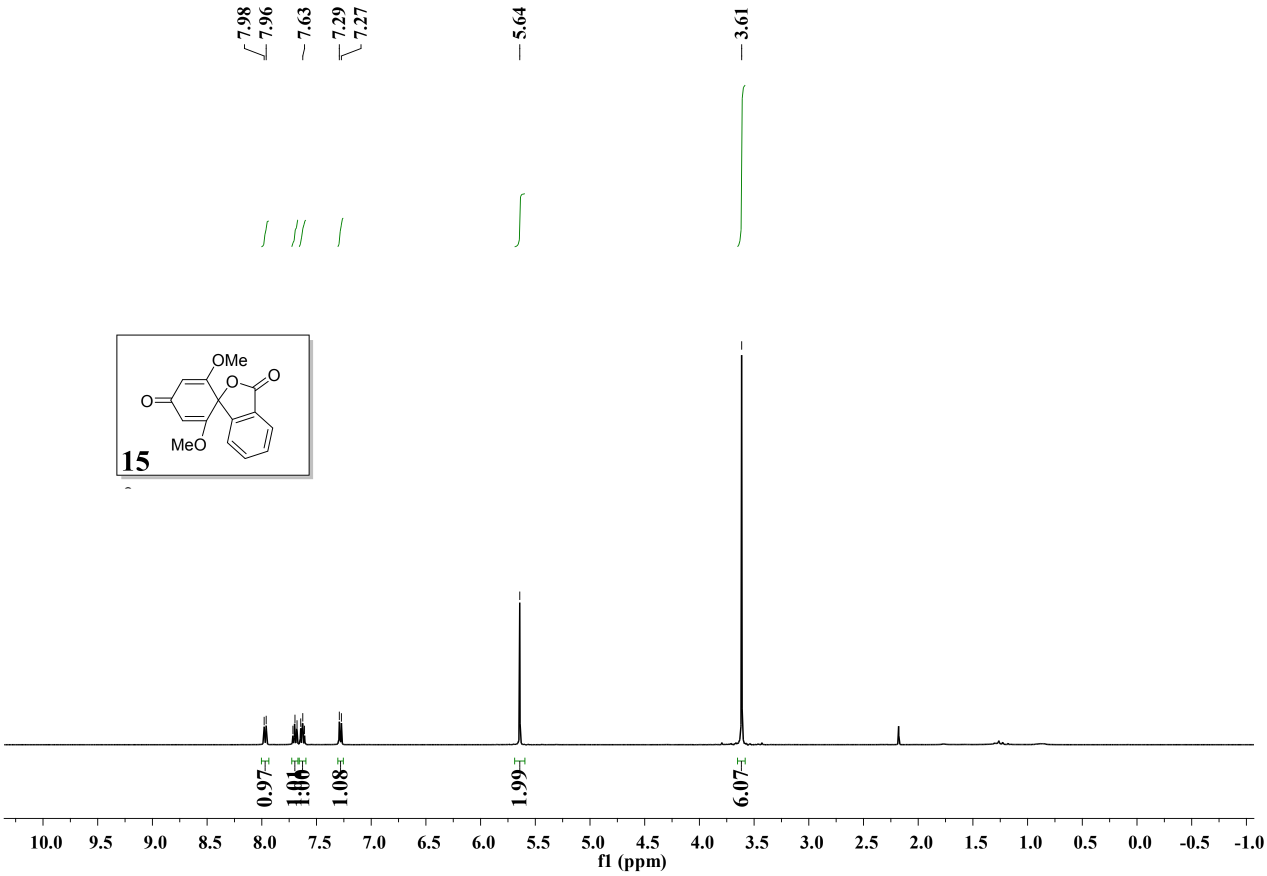
7.98
7.96
7.63
7.29
7.27
5.64
3.61
OMe
MeO
15
0.97
1.01
1.06
1.08
1.99
6.07
10.0 9.5 9.0 8.5 8.0 7.5 7.0 6.5 6.0 5.5 5.0 4.5 4.0 3.5 3.0 2.5 2.0 1.5 1.0 0.5 0.0 -0.5 -1.0
f1 (ppm)

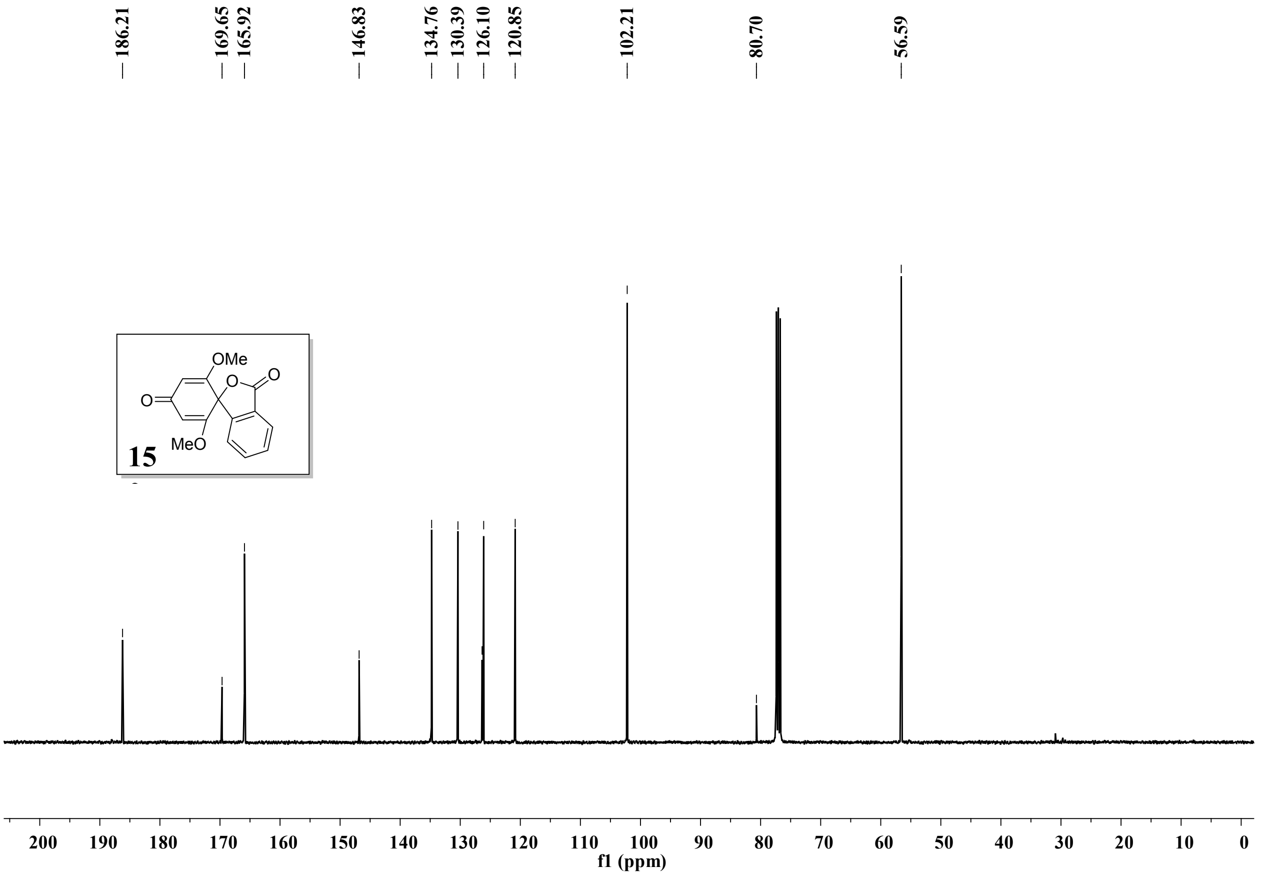
186.21
169.65
165.92
146.83
134.76
130.39
126.10
120.85
102.21
80.70
56.59
OMe
MeO
15
200 190 180 170 160 150 140 130 120 110 100 90 80 70 60 50 40 30 20 10 0
f1 (ppm)

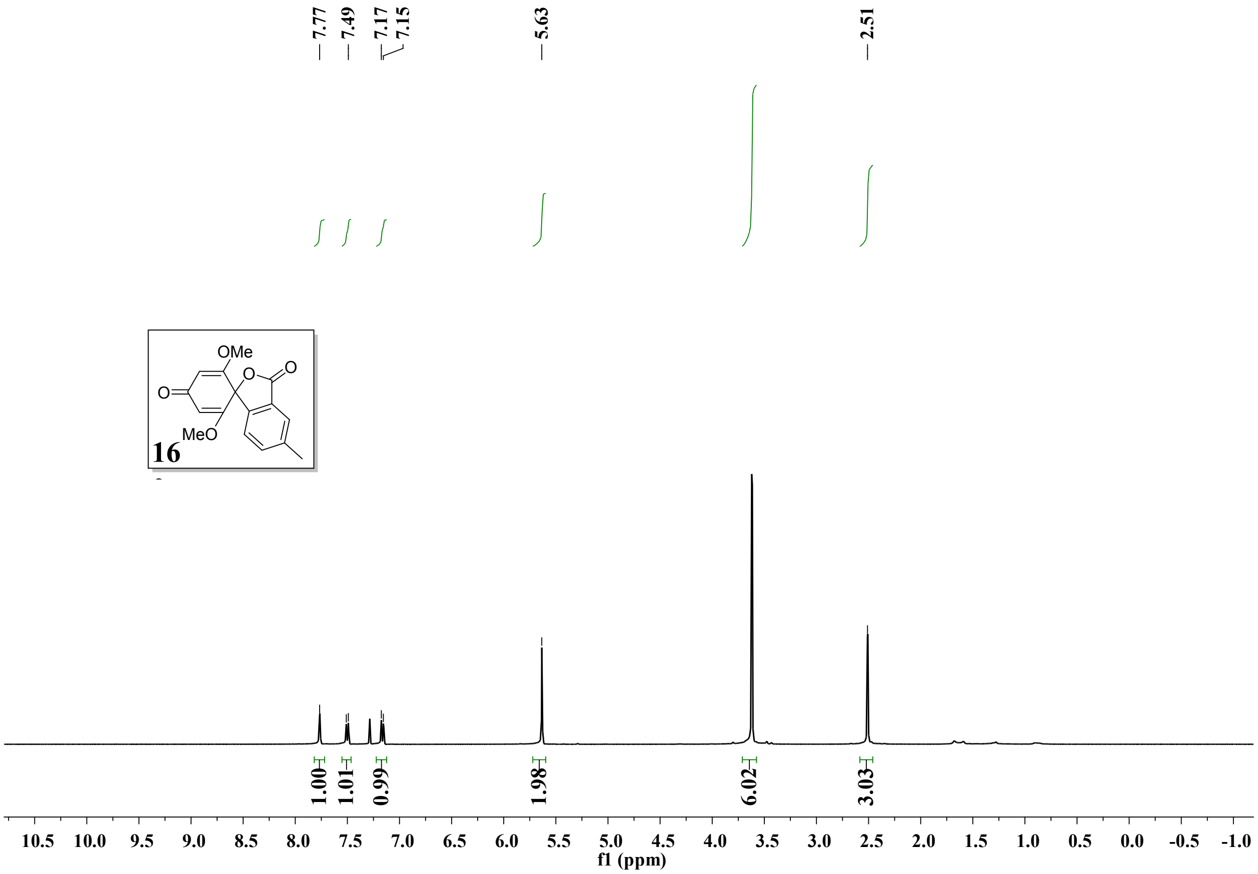
7.77
7.49
7.17
7.15
5.63
2.51
OMe
O
O
O
MeO
16
1.00
1.01
0.99
1.98
6.02
3.03
10.5 10.0 9.5 9.0 8.5 8.0 7.5 7.0 6.5 6.0 5.5 5.0 4.5 4.0 3.5 3.0 2.5 2.0 1.5 1.0 0.5 0.0 -0.5 -1.0
f1 (ppm)

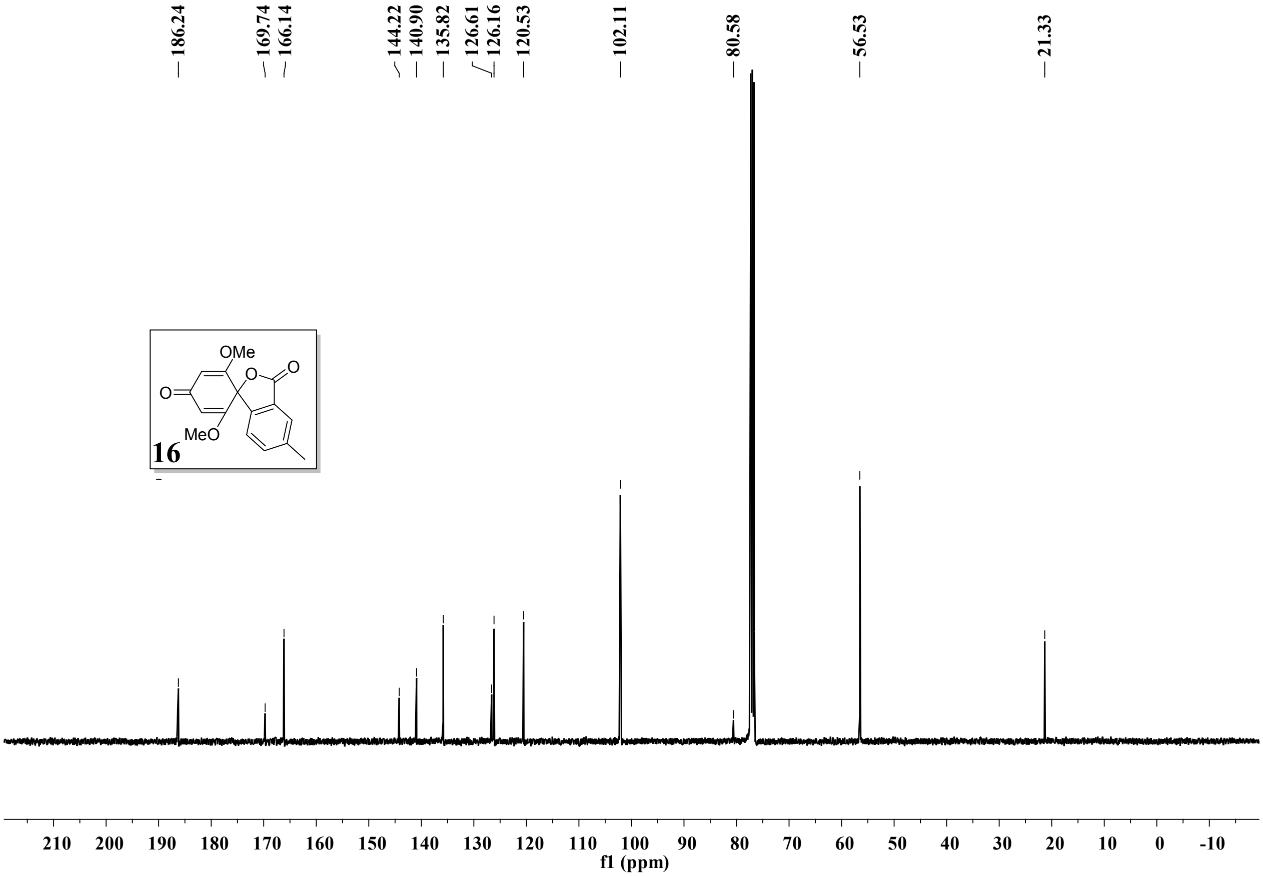
186.24
169.74
166.14
144.22
140.90
135.82
126.61
126.16
120.53
102.11
80.58
56.53
21.33
OMe
O
O
O
MeO
16
210 200 190 180 170 160 150 140 130 120 110 100 90 80 70 60 50 40 30 20 10 0 -10
f1 (ppm)

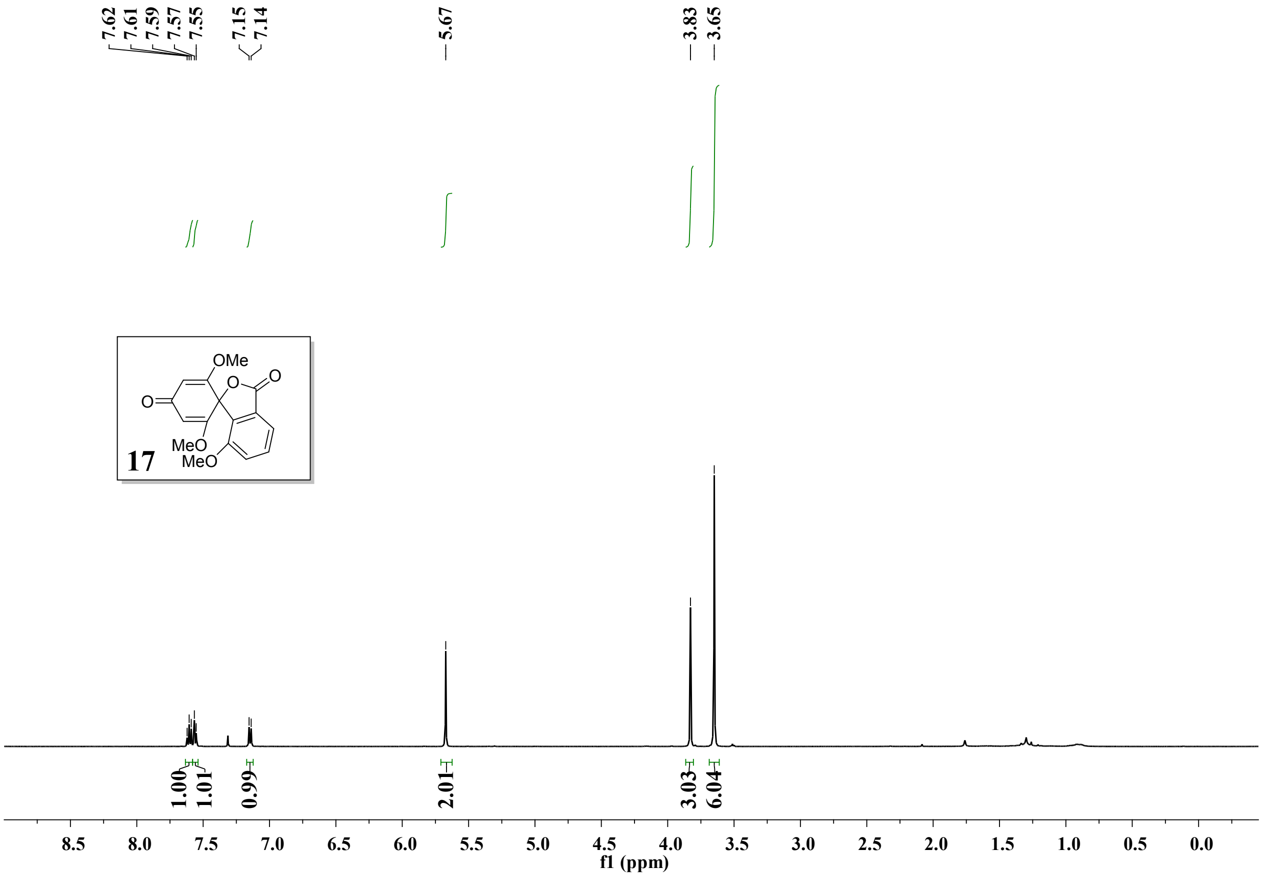
7.62
7.61
7.59
7.57
7.55
7.15
7.14
5.67
3.83
3.65
OMe
O
O
O
MeO
MeO
17
1.00
1.01
0.99
2.01
3.03
6.04
8.5 8.0 7.5 7.0 6.5 6.0 5.5 5.0 4.5 4.0 3.5 3.0 2.5 2.0 1.5 1.0 0.5 0.0
f1 (ppm)

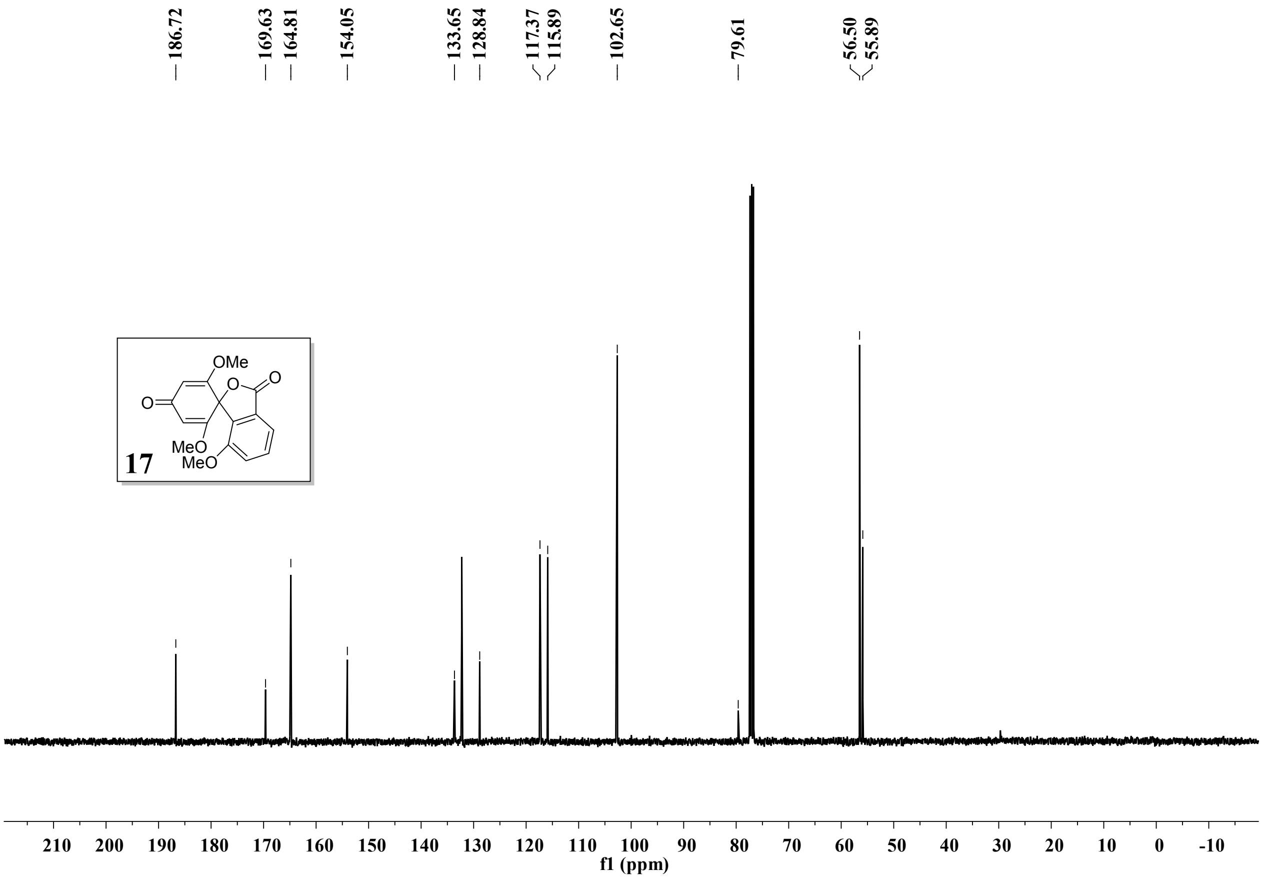
186.72
169.63
164.81
154.05
133.65
128.84
117.37
115.89
102.65
79.61
56.50
55.89
OMe
O
O
O
MeO
MeO
17
210 200 190 180 170 160 150 140 130 120 110 100 90 80 70 60 50 40 30 20 10 0 -10
f1 (ppm)

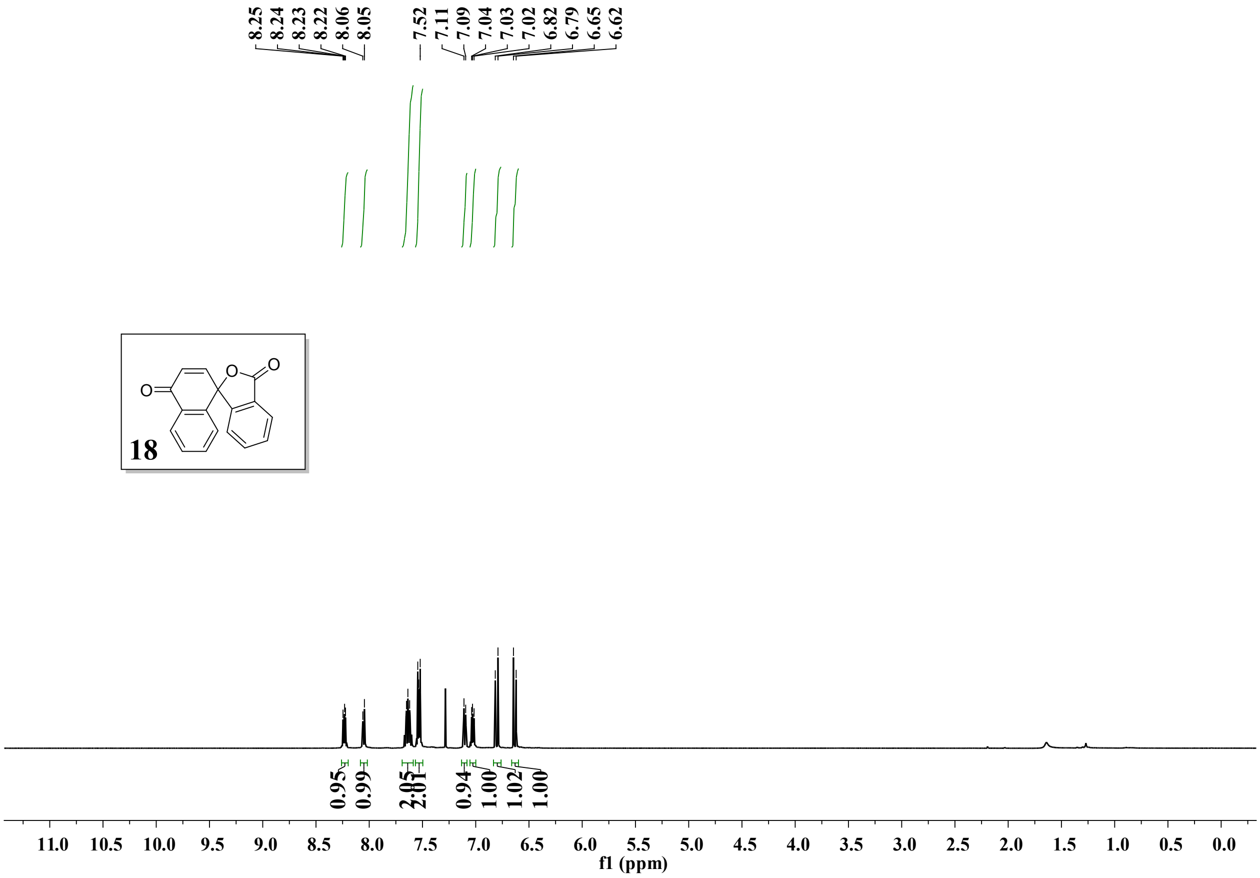
8.25
8.24
8.23
8.22
8.06
8.05
7.52
7.11
7.09
7.04
7.03
7.02
6.82
6.79
6.65
6.62
18
0.95
0.99
2.05
2.01
0.94
1.00
1.02
1.00
11.0 10.5 10.0 9.5 9.0 8.5 8.0 7.5 7.0 6.5 6.0 5.5 5.0 4.5 4.0 3.5 3.0 2.5 2.0 1.5 1.0 0.5 0.0
f1 (ppm)

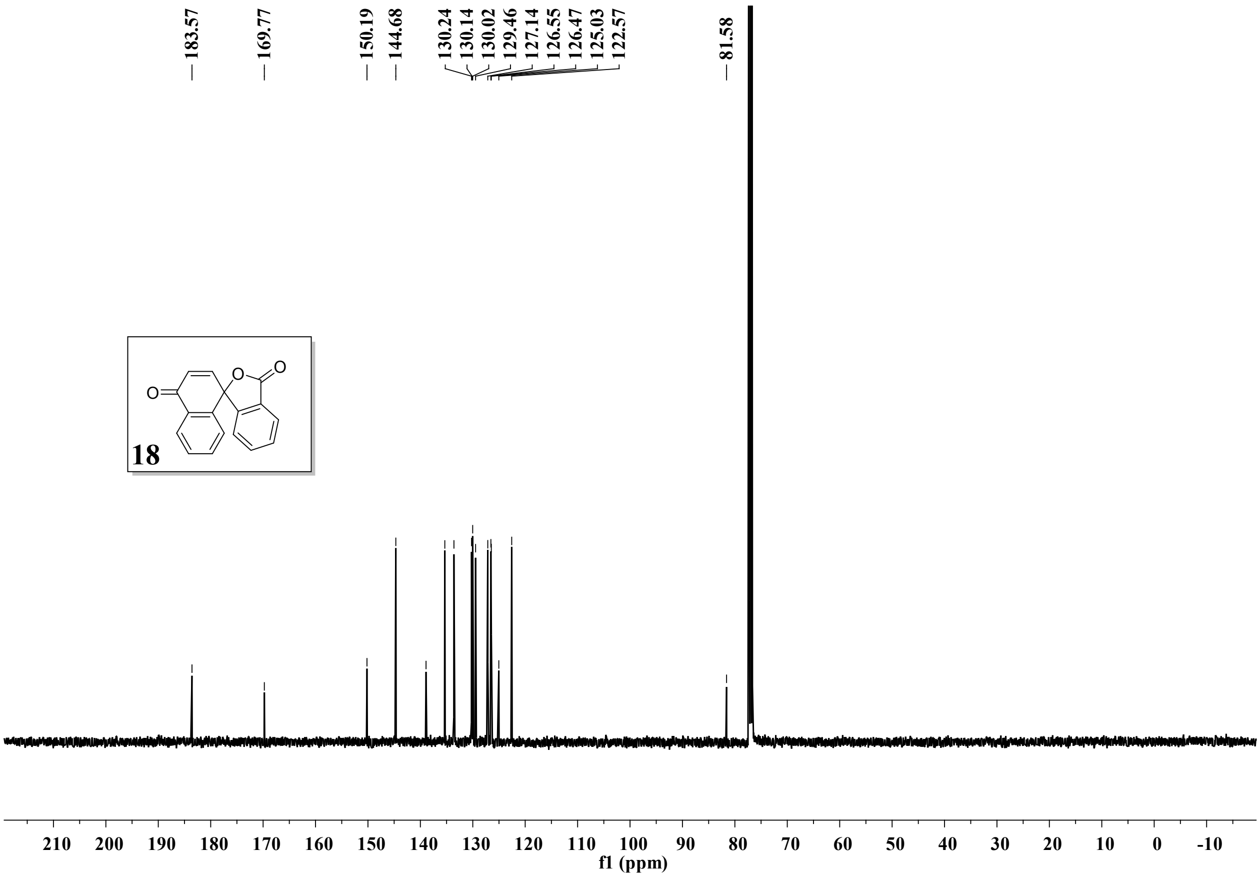
183.57
169.77
150.19
144.68
130.24
130.14
130.02
129.46
127.14
126.55
126.47
125.03
122.57
81.58
18
210 200 190 180 170 160 150 140 130 120 110 100 90 80 70 60 50 40 30 20 10 0 -10
f1 (ppm)

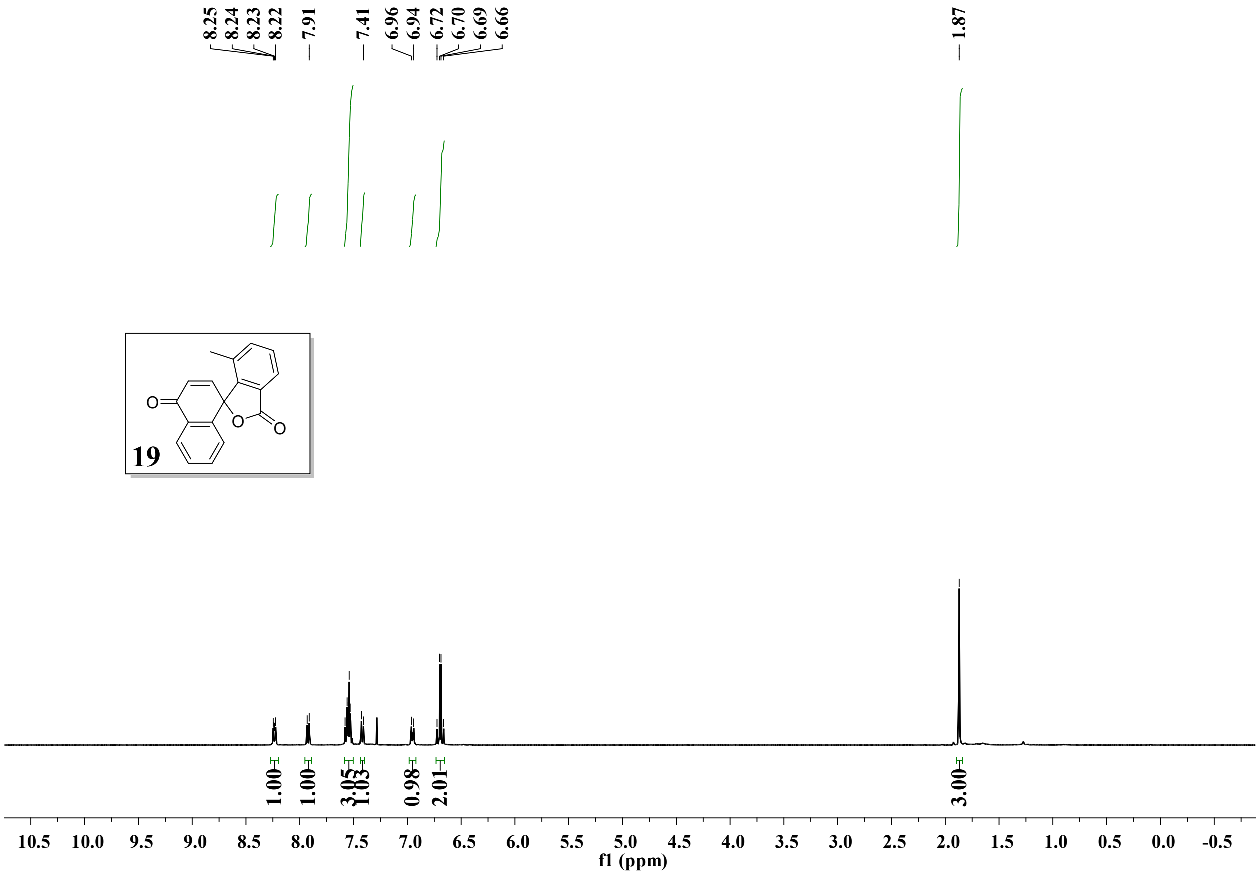
8.25
8.24
8.23
8.22
7.91
7.41
6.96
6.94
6.72
6.70
6.69
6.66
1.87
19
1.00
1.00
3.05
1.03
0.98
2.01
3.00
10.5 10.0 9.5 9.0 8.5 8.0 7.5 7.0 6.5 6.0 5.5 5.0 4.5 4.0 3.5 3.0 2.5 2.0 1.5 1.0 0.5 0.0 -0.5
f1 (ppm)

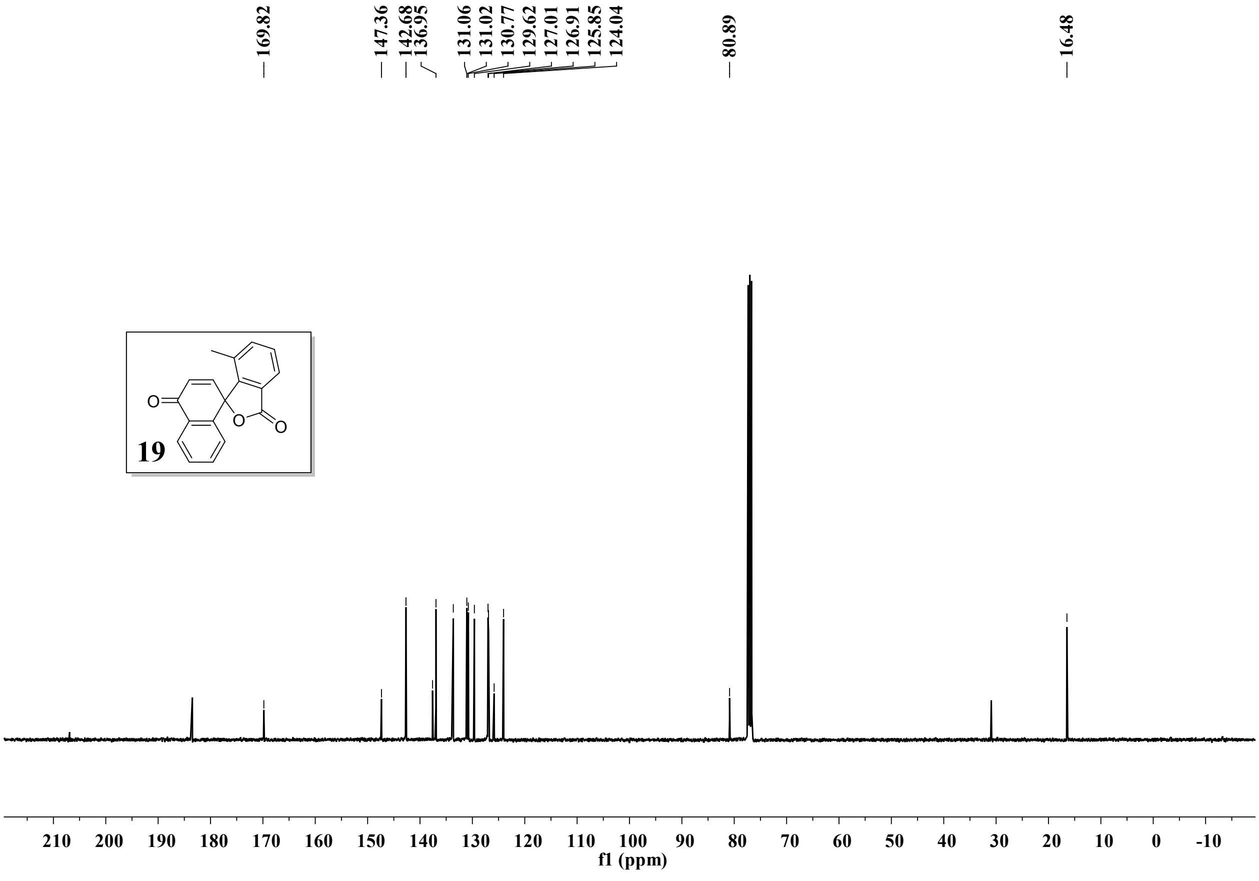
169.82
147.36
142.68
136.95
131.06
131.02
130.77
129.62
127.01
126.91
125.85
124.04
80.89
16.48
19
210 200 190 180 170 160 150 140 130 120 110 100 90 80 70 60 50 40 30 20 10 0 -10
f1 (ppm)

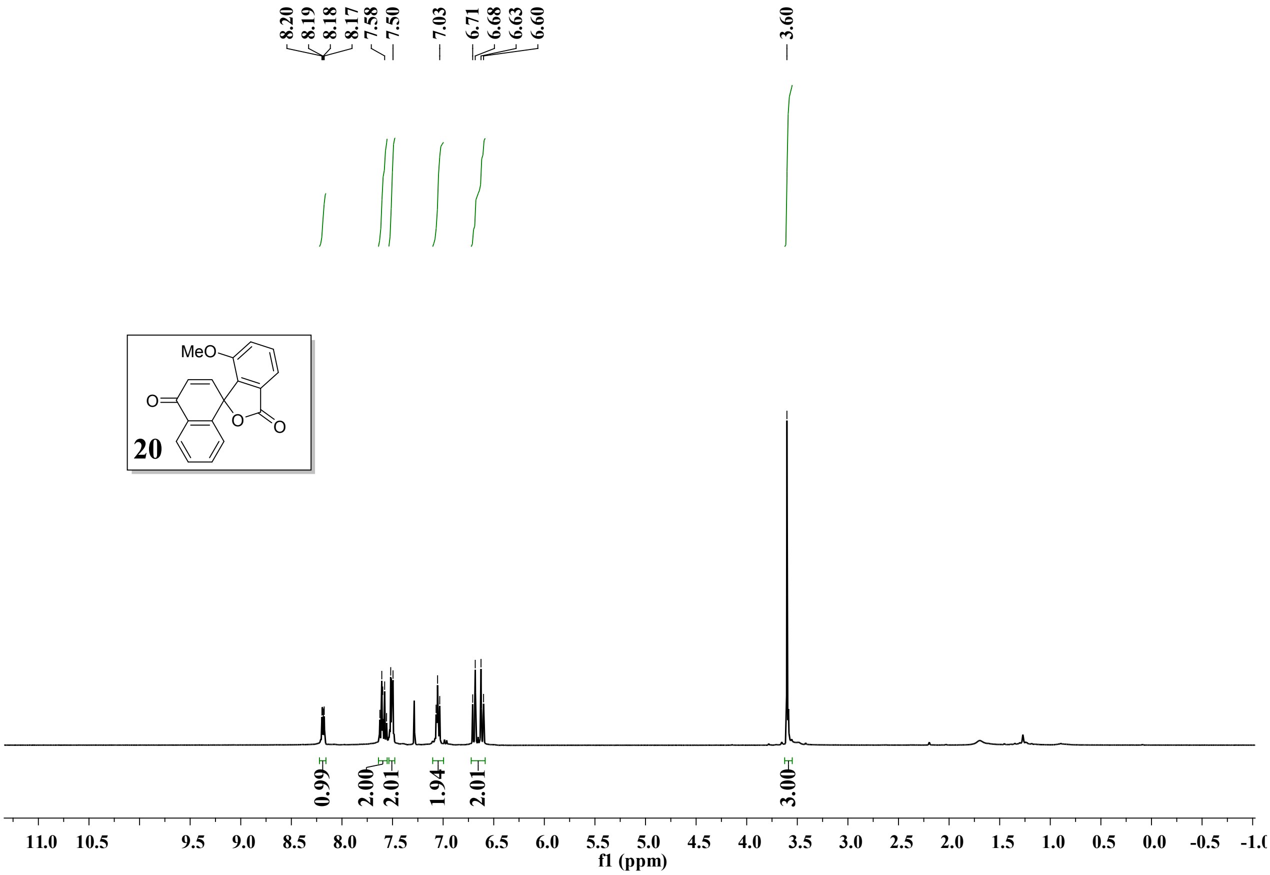
8.20
8.19
8.18
8.17
7.58
7.50
7.03
6.71
6.68
6.63
6.60
3.60
MeO
20
0.99
2.00
2.01
1.94
2.01
3.00
f1 (ppm)

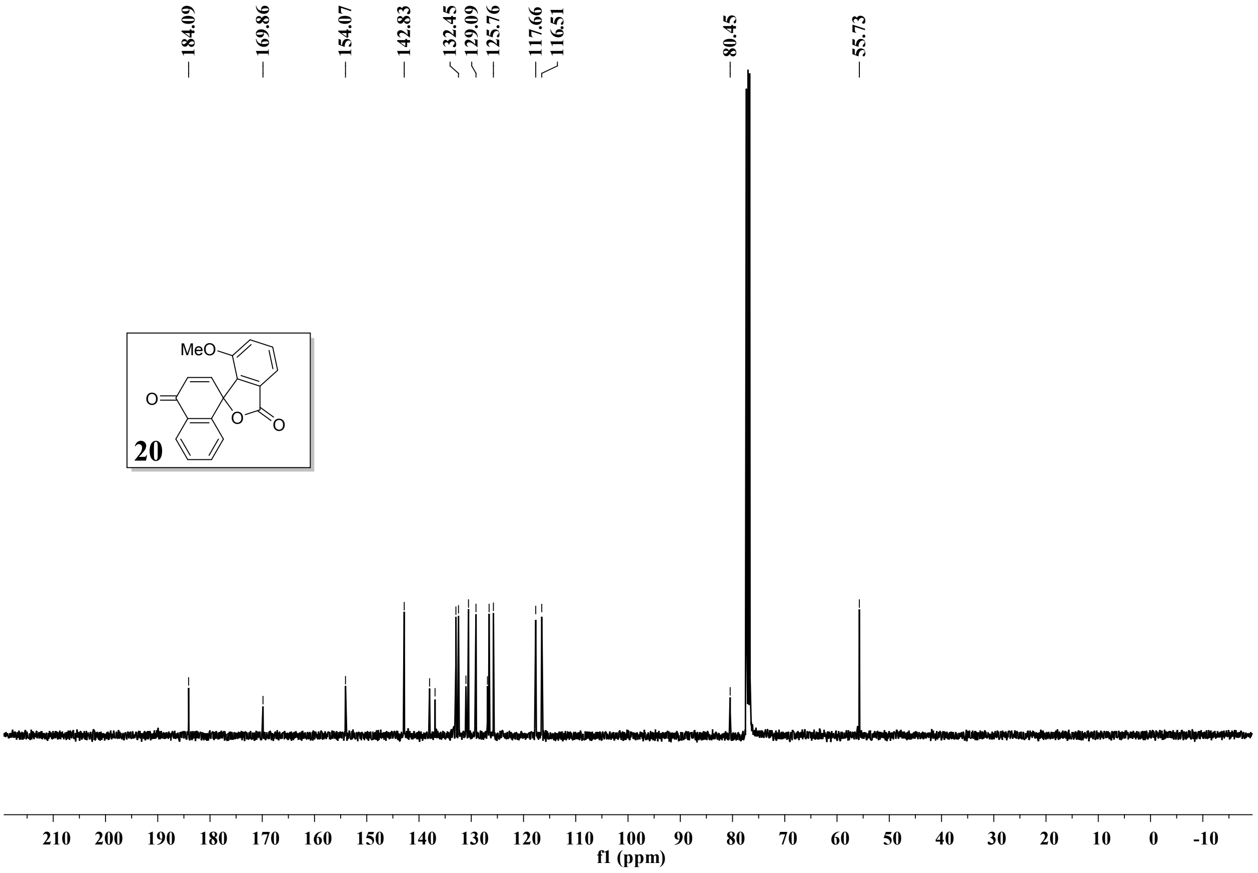
184.09
169.86
154.07
142.83
132.45
129.09
125.76
117.66
116.51
80.45
55.73
MeO
20
f1 (ppm)

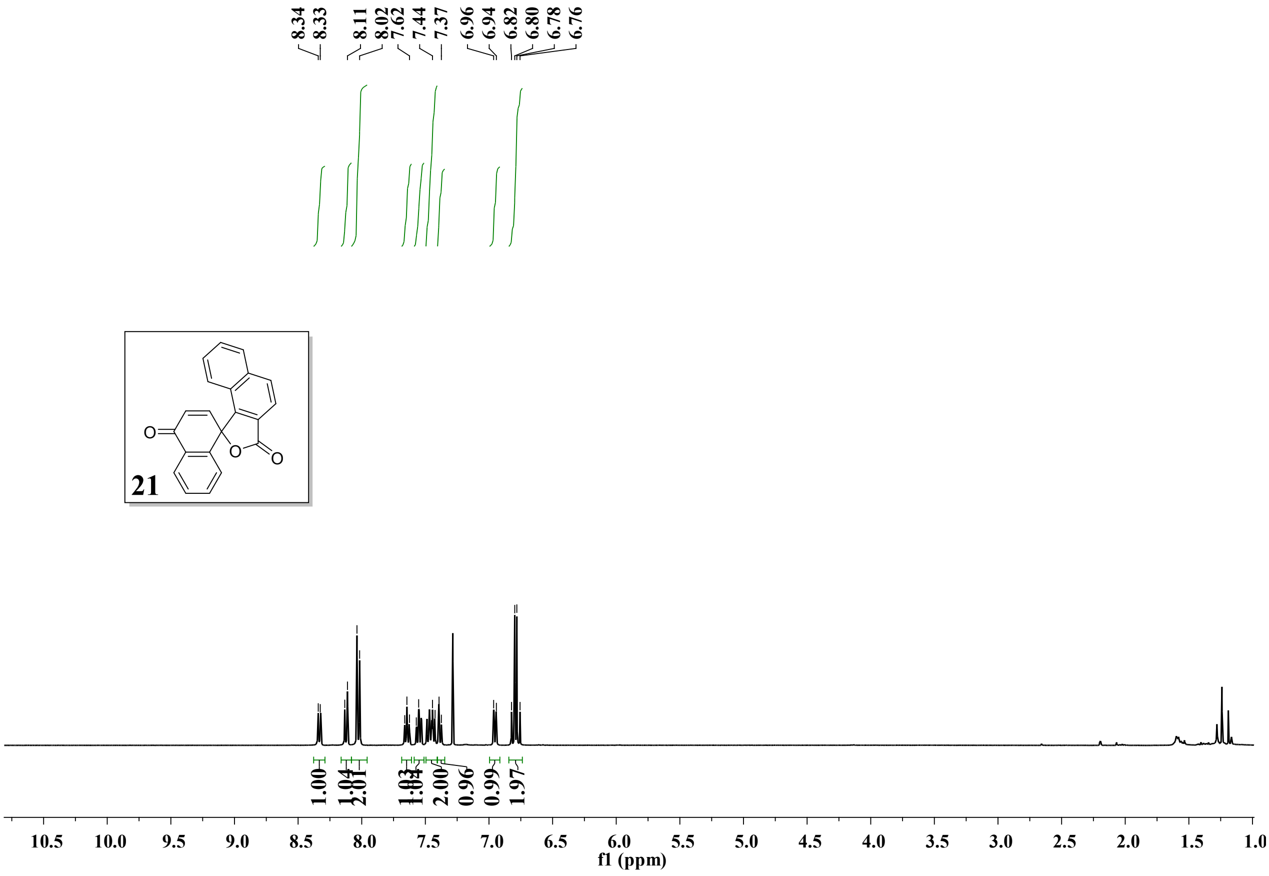
8.34
8.33
8.11
8.02
7.62
7.44
7.37
6.96
6.94
6.82
6.80
6.78
6.76
21
1.00
1.04
2.01
1.03
1.04
2.00
0.96
0.99
1.97
10.5 10.0 9.5 9.0 8.5 8.0 7.5 7.0 6.5 6.0 5.5 5.0 4.5 4.0 3.5 3.0 2.5 2.0 1.5 1.0
f1 (ppm)

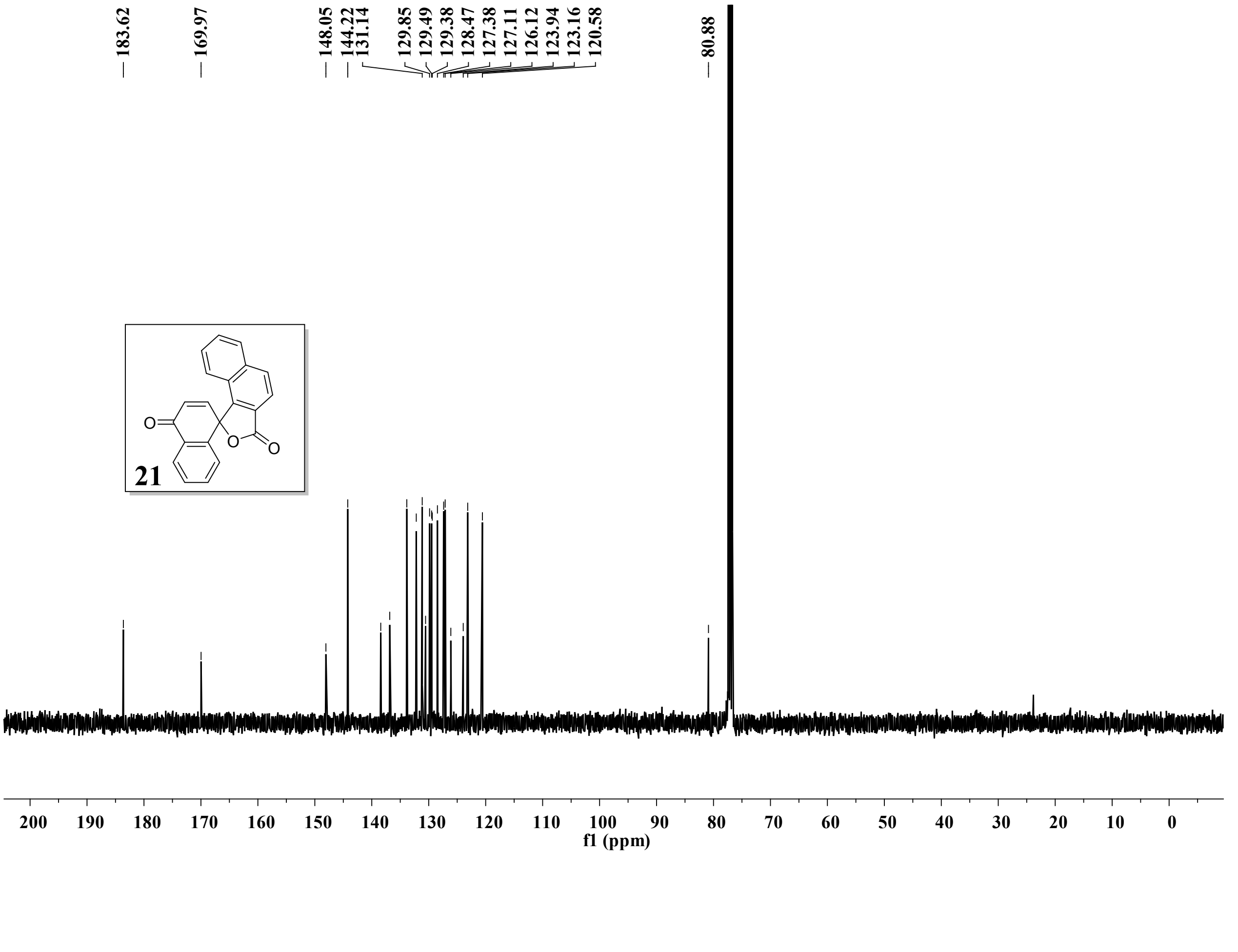
183.62
169.97
148.05
144.22
131.14
129.85
129.49
129.38
128.47
127.38
127.11
126.12
123.94
123.16
120.58
80.88
21
200 190 180 170 160 150 140 130 120 110 100 90 80 70 60 50 40 30 20 10 0
f1 (ppm)

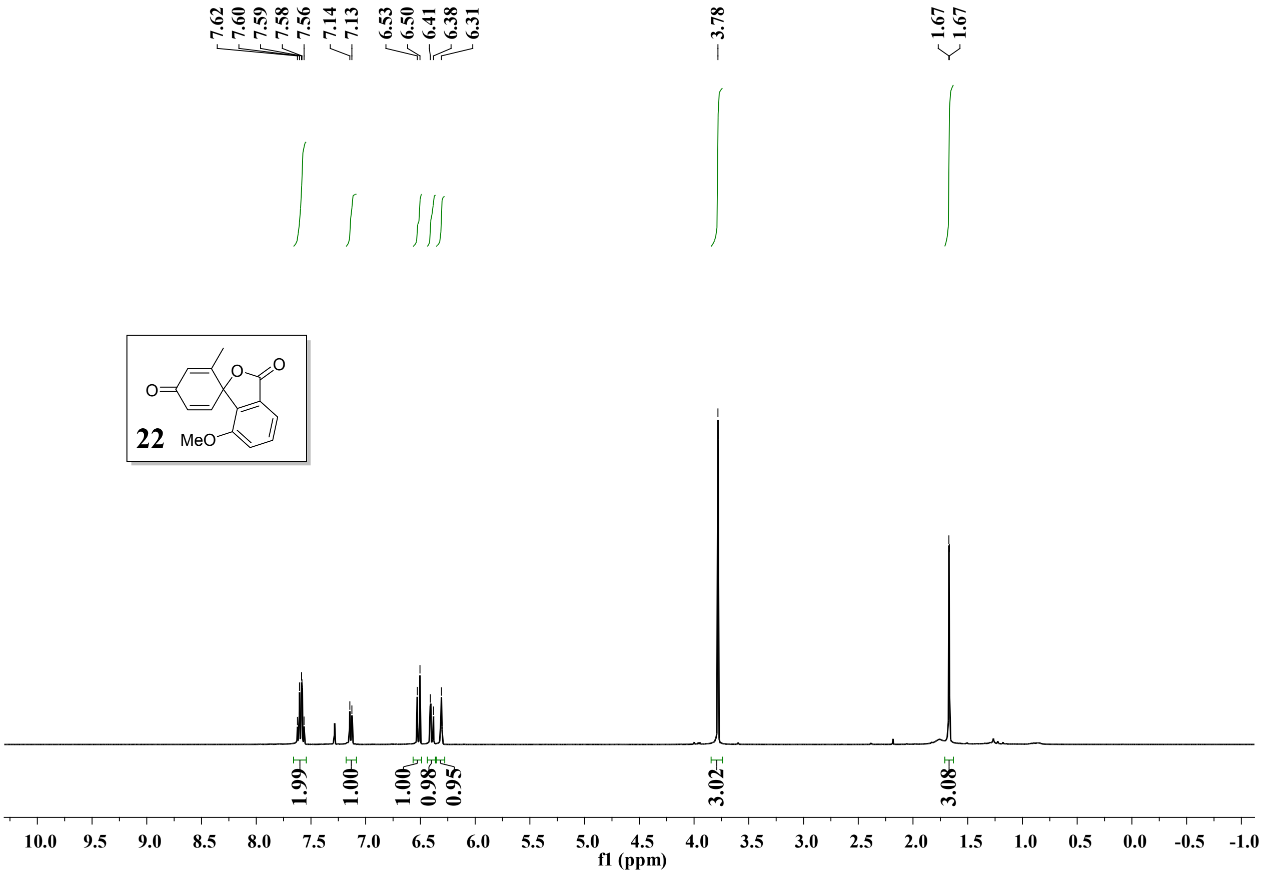
7.62
7.60
7.59
7.58
7.56
7.14
7.13
6.53
6.50
6.41
6.38
6.31
3.78
1.67
1.67
22
MeO
O
1.99
1.00
1.00
0.98
0.95
3.02
3.08
10.0 9.5 9.0 8.5 8.0 7.5 7.0 6.5 6.0 5.5 5.0 4.5 4.0 3.5 3.0 2.5 2.0 1.5 1.0 0.5 0.0 -0.5 -1.0
f1 (ppm)

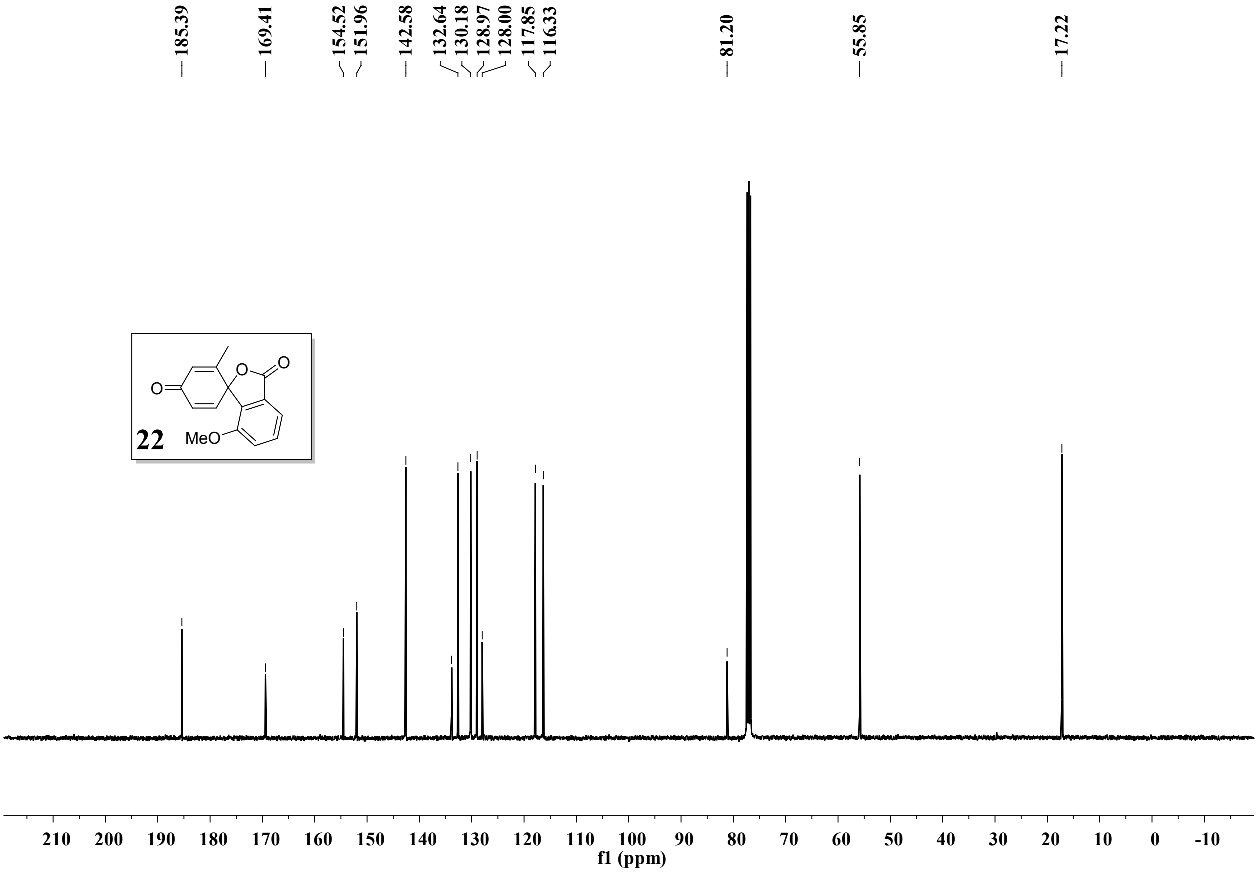
185.39
169.41
154.52
151.96
142.58
132.64
130.18
128.97
128.00
117.85
116.33
81.20
55.85
17.22
22
MeO
O
210 200 190 180 170 160 150 140 130 120 110 100 90 80 70 60 50 40 30 20 10 0 -10
f1 (ppm)

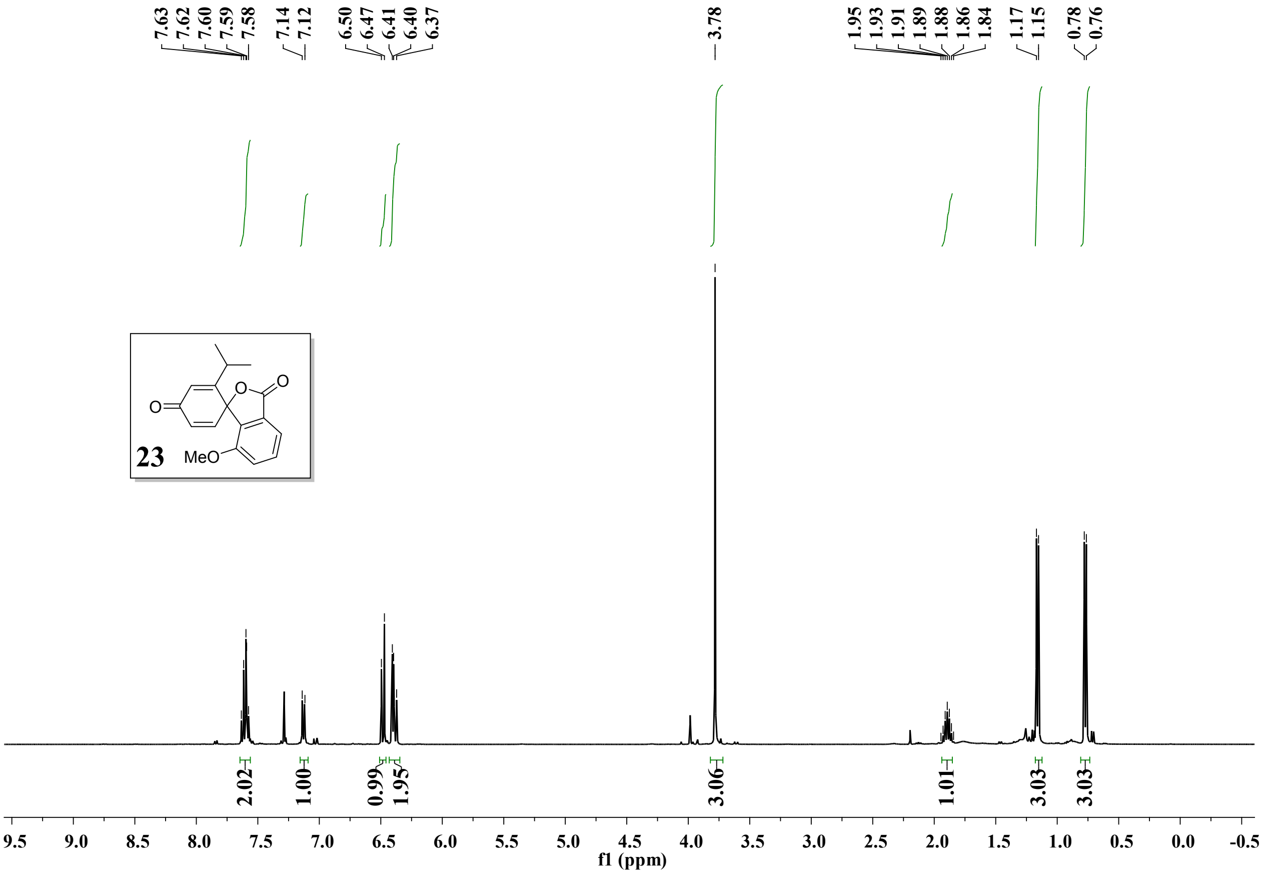
7.63
7.62
7.60
7.59
7.58
7.14
7.12
6.50
6.47
6.41
6.40
6.37
3.78
1.95
1.93
1.91
1.89
1.88
1.86
1.84
1.17
1.15
0.78
0.76
23
MeO
2.02
1.00
0.99
1.95
3.06
1.01
3.03
3.03
f1 (ppm)

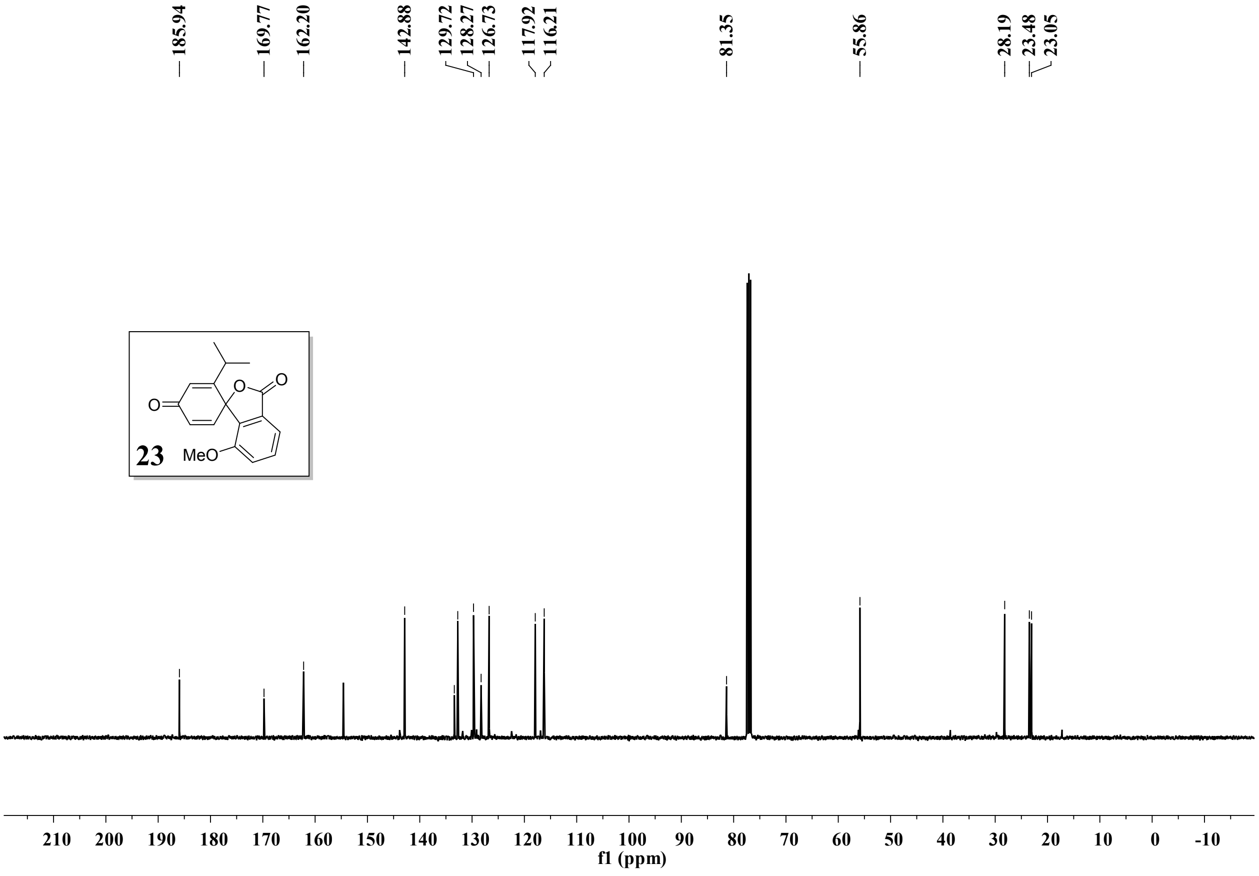
185.94
169.77
162.20
142.88
129.72
128.27
126.73
117.92
116.21
81.35
55.86
28.19
23.48
23.05
23
MeO
f1 (ppm)

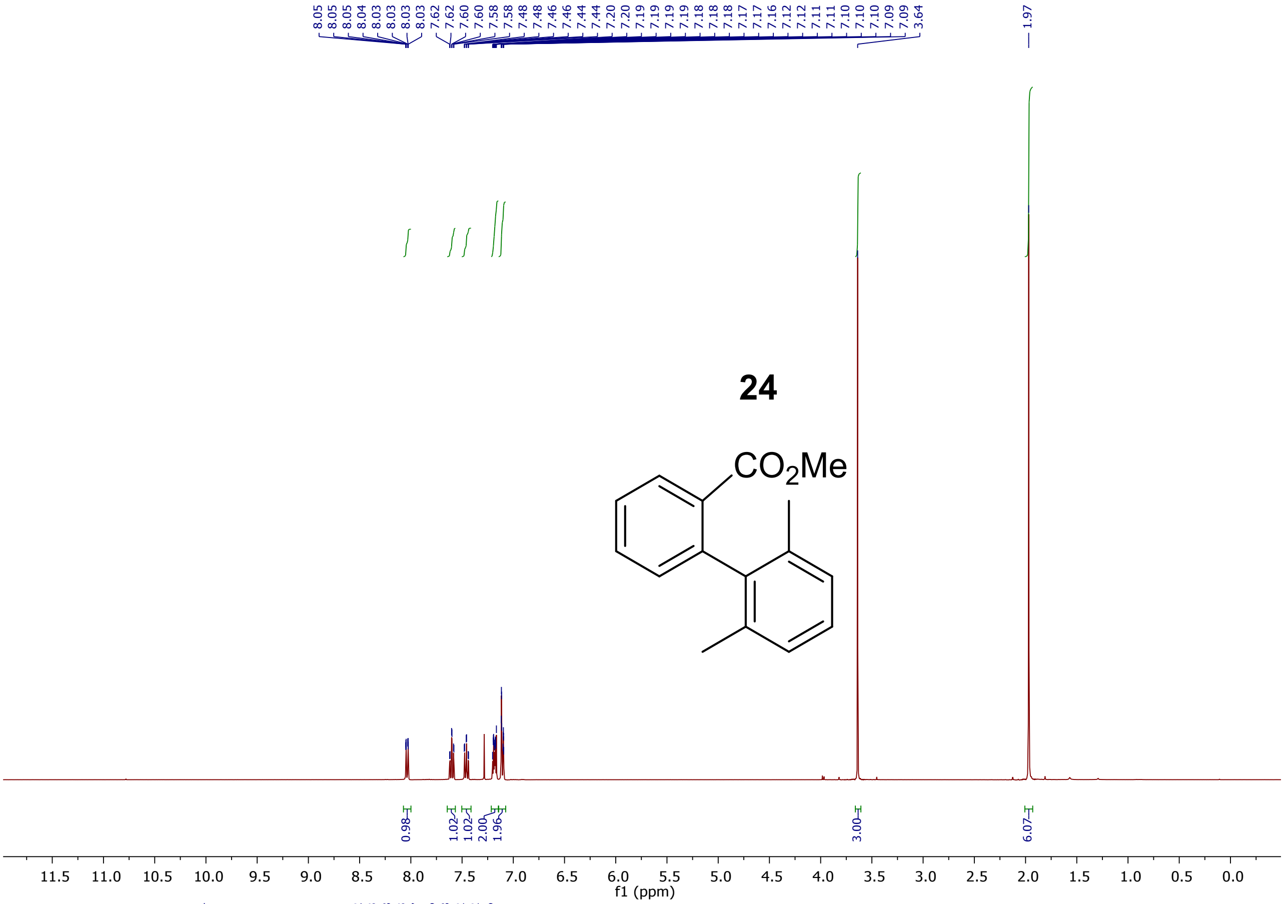
8.05 8.05 8.05 8.04 8.03 8.03 8.03 8.03 7.62 7.62 7.60 7.60 7.58 7.58 7.48 7.48 7.46 7.46 7.44 7.44 7.20 7.20 7.19 7.19 7.19 7.19 7.18 7.18 7.18 7.17 7.17 7.16 7.12 7.12 7.11 7.11 7.10 7.10 7.10 7.09 7.09 3.64
1.97
24
CO2Me
0.98 1.02 1.02 2.00 1.96
3.00
6.07
11.5 11.0 10.5 10.0 9.5 9.0 8.5 8.0 7.5 7.0 6.5 6.0 5.5 5.0 4.5 4.0 3.5 3.0 2.5 2.0 1.5 1.0 0.5 0.0
f1 (ppm)

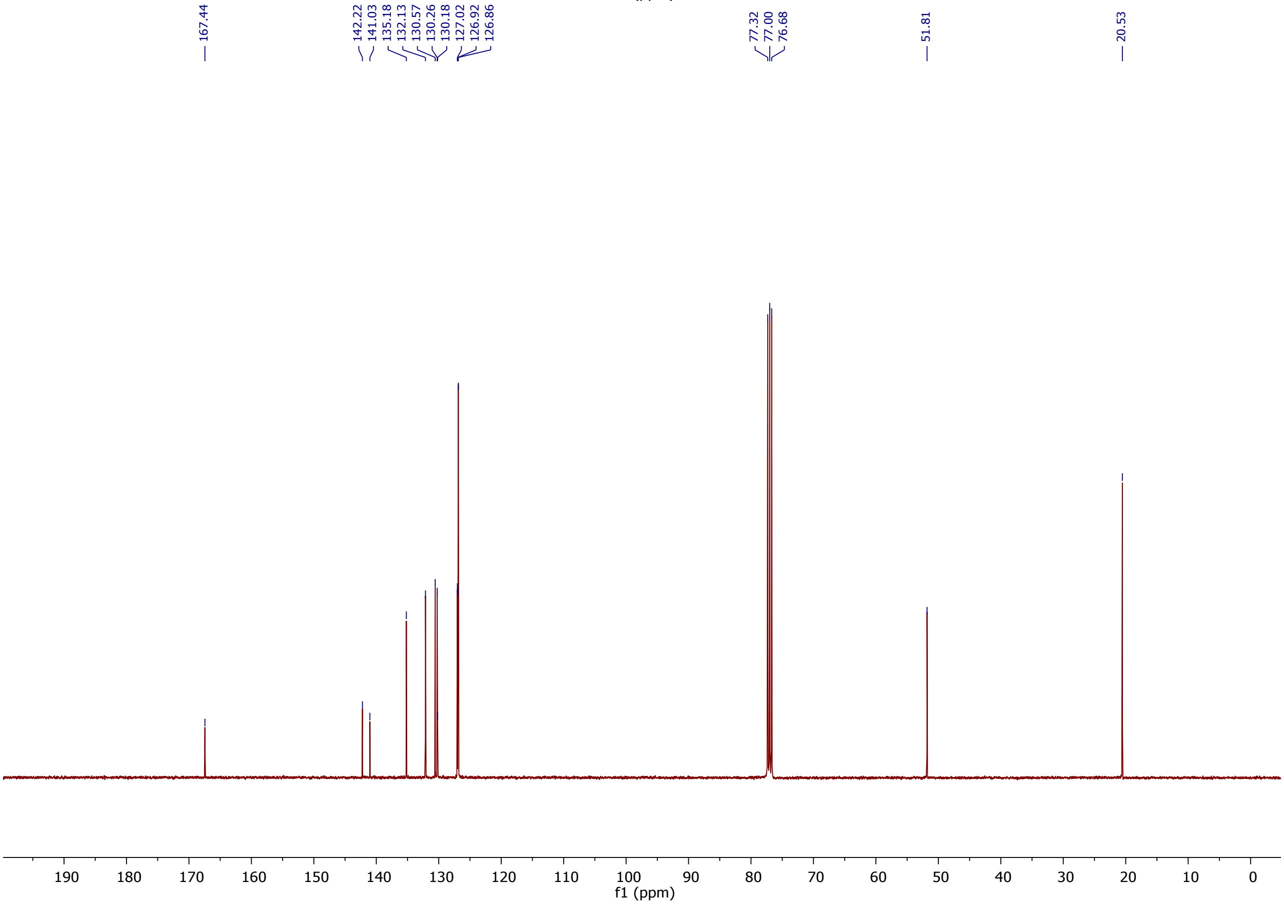
167.44
142.22 141.03 135.18 132.13 130.57 130.26 130.18 127.02 126.92 126.86
77.32 77.00 76.68
51.81
20.53
190 180 170 160 150 140 130 120 110 100 90 80 70 60 50 40 30 20 10 0
f1 (ppm)

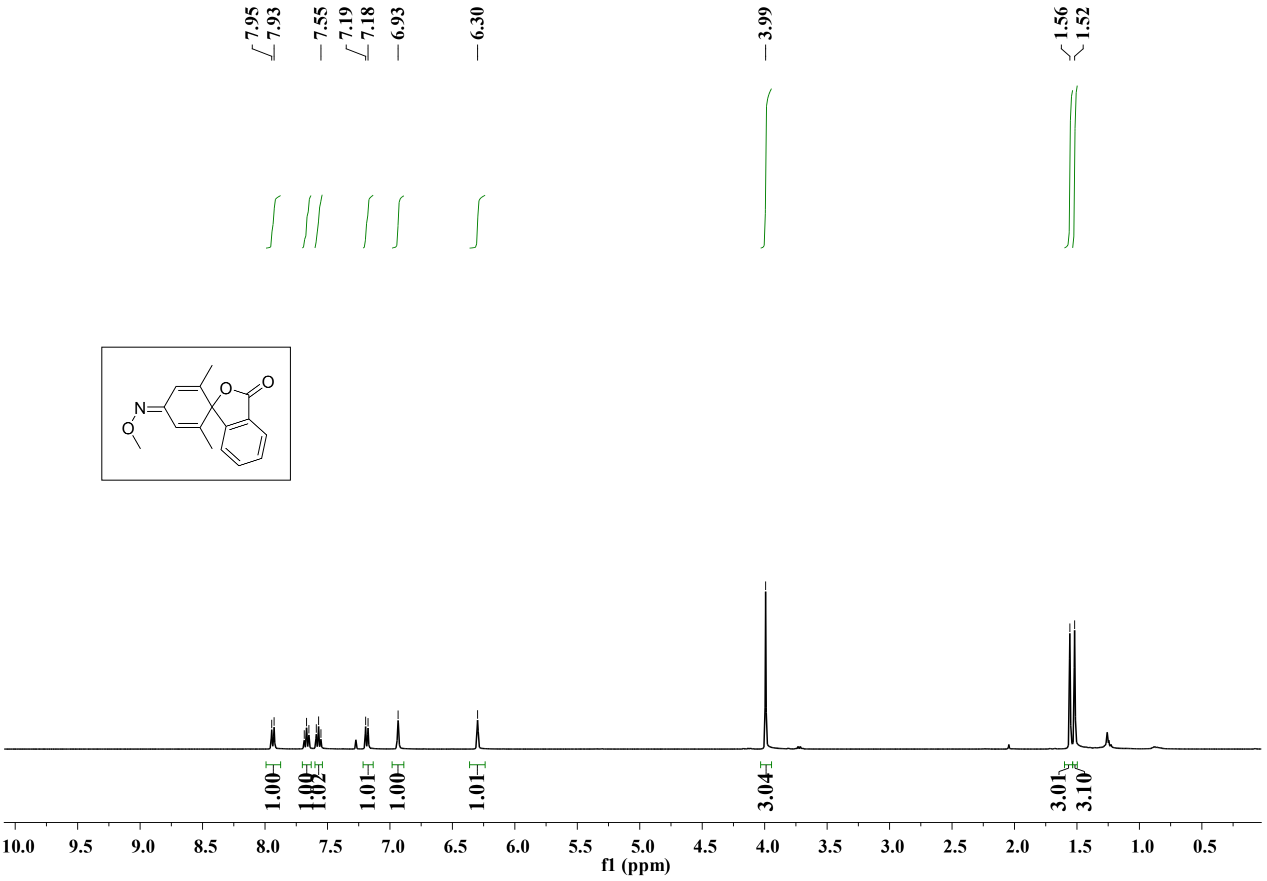
7.95
7.93
7.55
7.19
7.18
6.93
6.30
3.99
1.56
1.52
N
O
O
O
1.00
1.00
1.02
1.01
1.00
1.01
3.04
3.01
3.10
10.0 9.5 9.0 8.5 8.0 7.5 7.0 6.5 6.0 5.5 5.0 4.5 4.0 3.5 3.0 2.5 2.0 1.5 1.0 0.5
f1 (ppm)

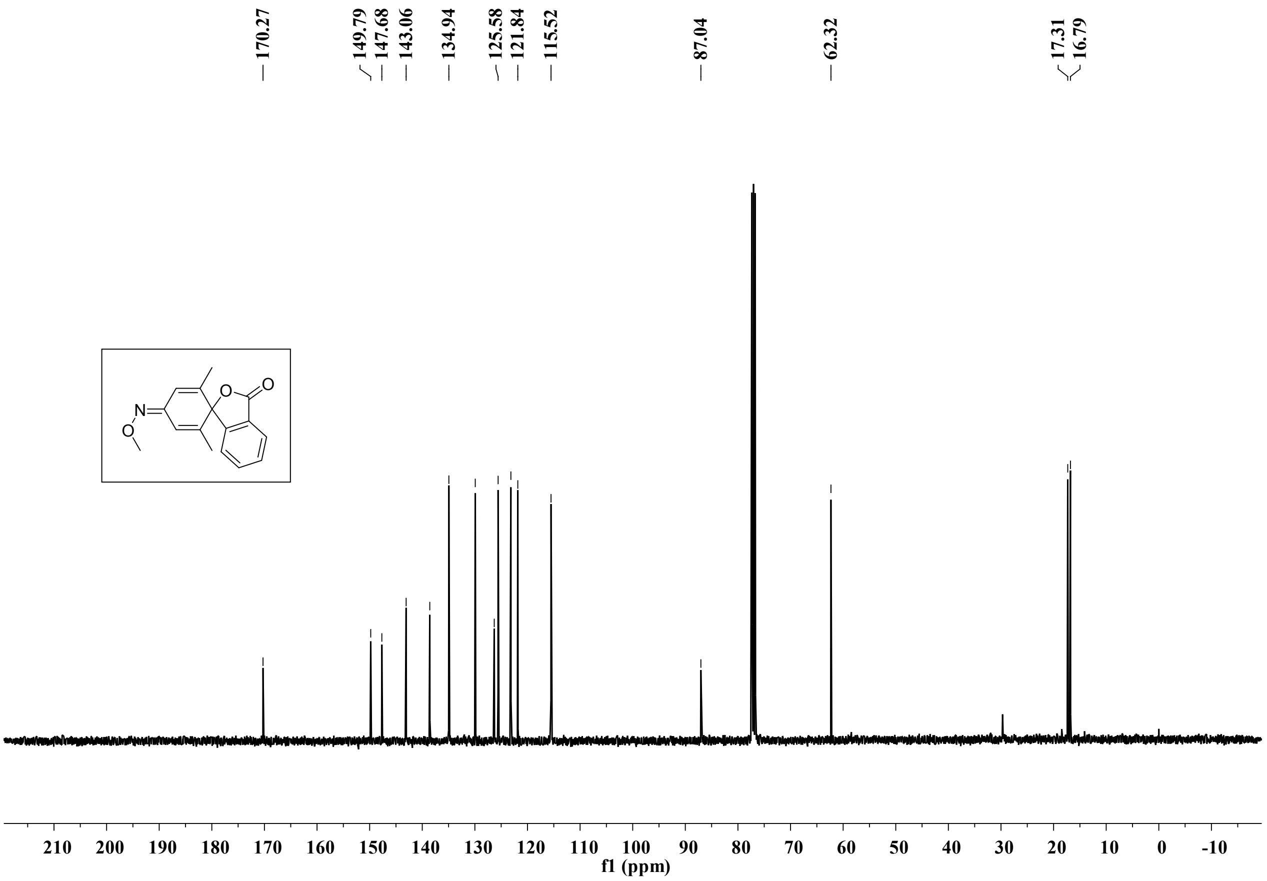
170.27
149.79
147.68
143.06
134.94
125.58
121.84
115.52
87.04
62.32
17.31
16.79
N
O
O
O
210 200 190 180 170 160 150 140 130 120 110 100 90 80 70 60 50 40 30 20 10 0 -10
f1 (ppm)

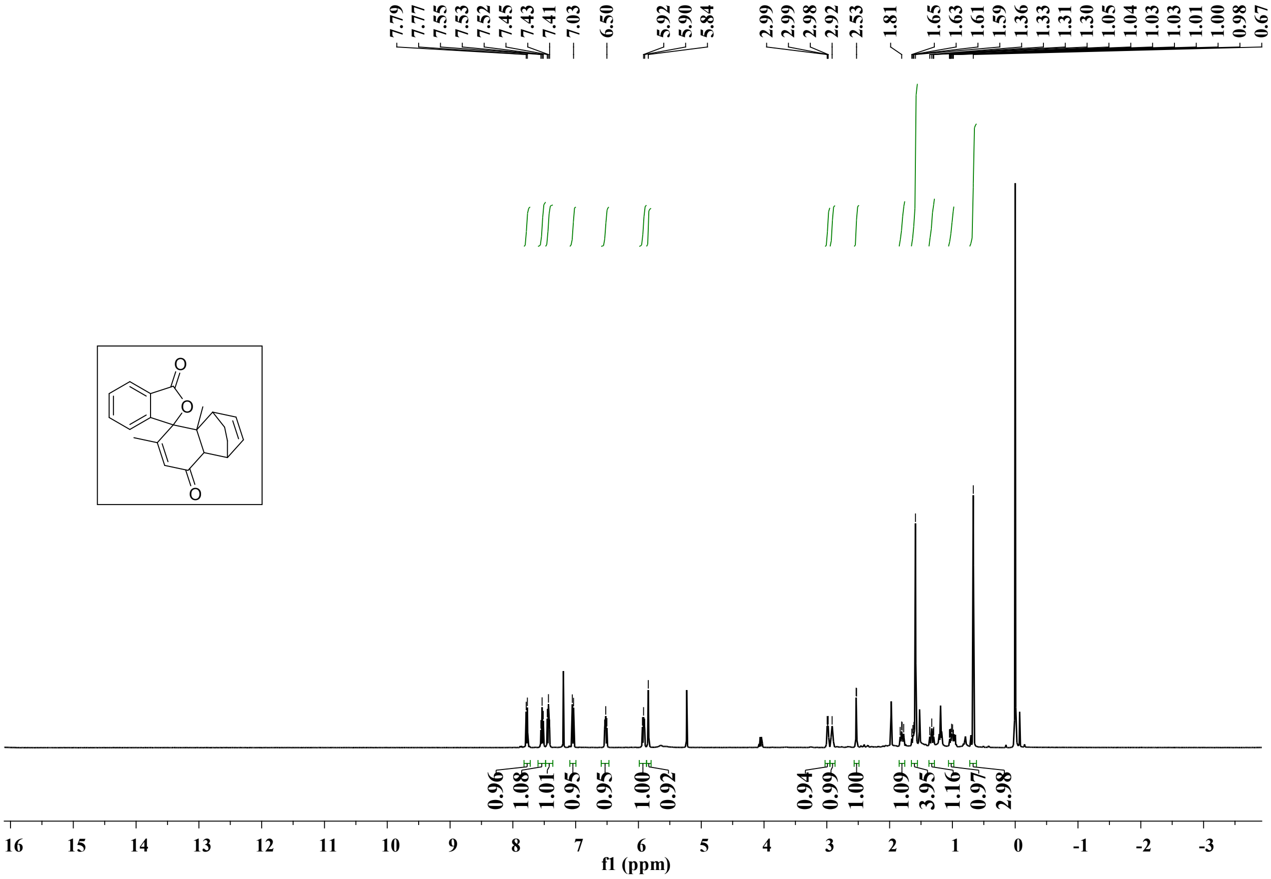
7.79 7.77 7.55 7.53 7.52 7.45 7.43 7.41 7.03 6.50 5.92 5.90 5.84 2.99 2.99 2.98 2.92 2.53 1.81 1.65 1.63 1.61 1.59 1.36 1.33 1.31 1.30 1.05 1.04 1.03 1.03 1.01 1.00 0.98 0.67
0.96 1.08 1.01 0.95 0.95 1.00 0.92 0.94 0.99 1.00 1.09 3.95 1.16 0.97 2.98
16 15 14 13 12 11 10 9 8 7 6 5 4 3 2 1 0 -1 -2 -3
f1 (ppm)

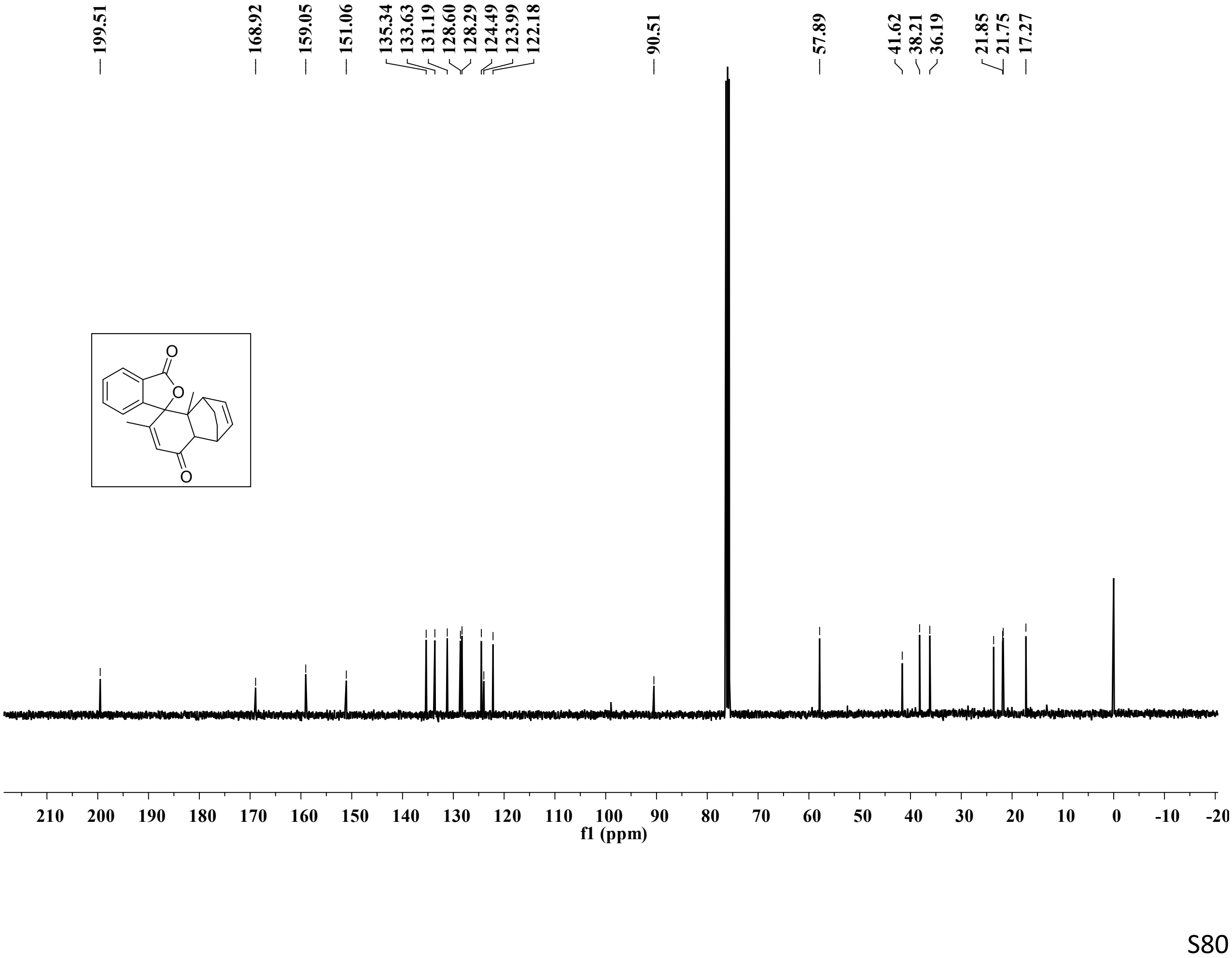
199.51 168.92 159.05 151.06 135.34 133.63 131.19 128.60 128.29 124.49 123.99 122.18 90.51 57.89 41.62 38.21 36.19 21.85 21.75 17.27
210 200 190 180 170 160 150 140 130 120 110 100 90 80 70 60 50 40 30 20 10 0 -10 -20
f1 (ppm)

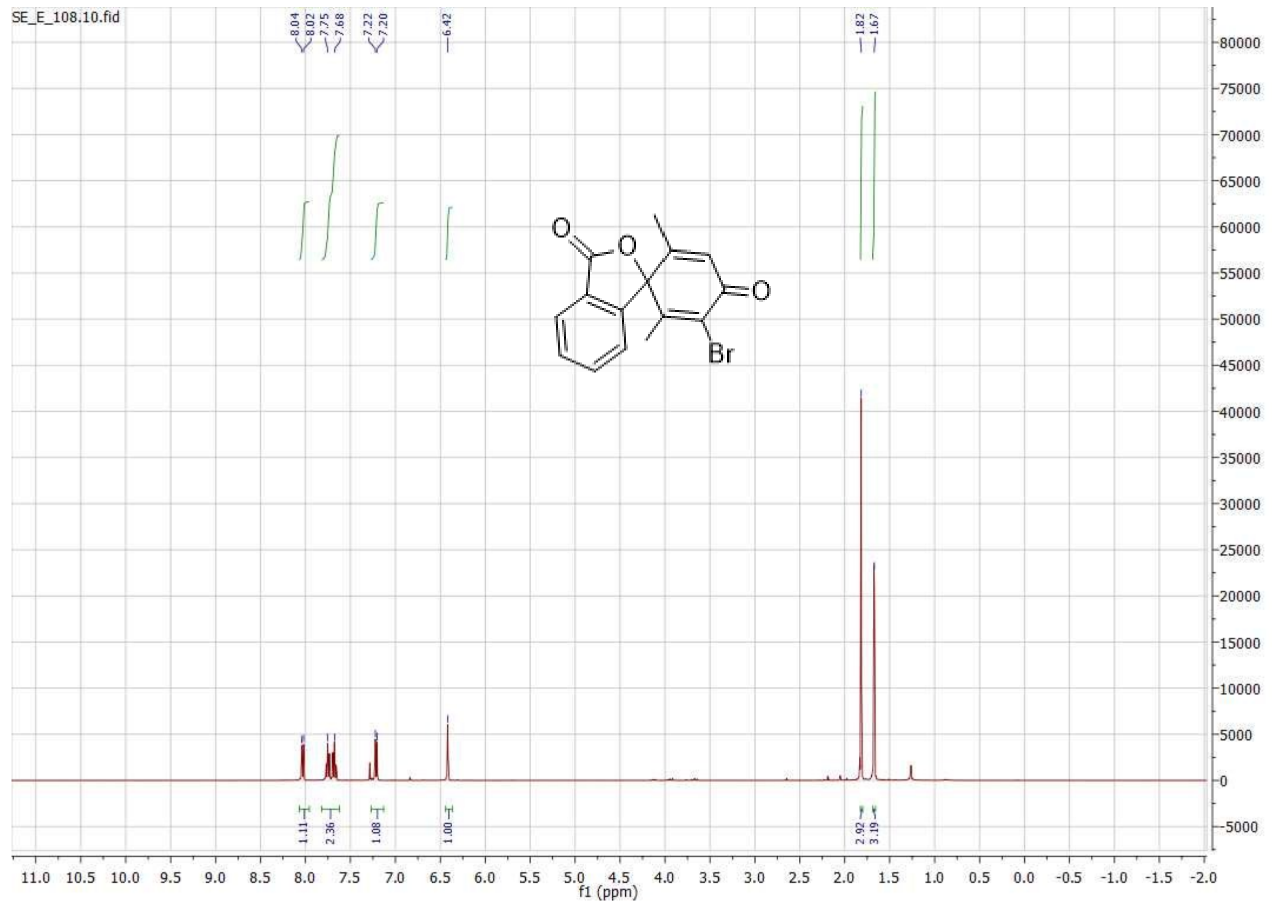
SE_E_108.10.fid
8.04
8.02
7.75
7.68
7.22
7.20
6.42
1.82
1.67
Br
1.11
2.36
1.08
1.00
2.92
3.19
f1 (ppm)

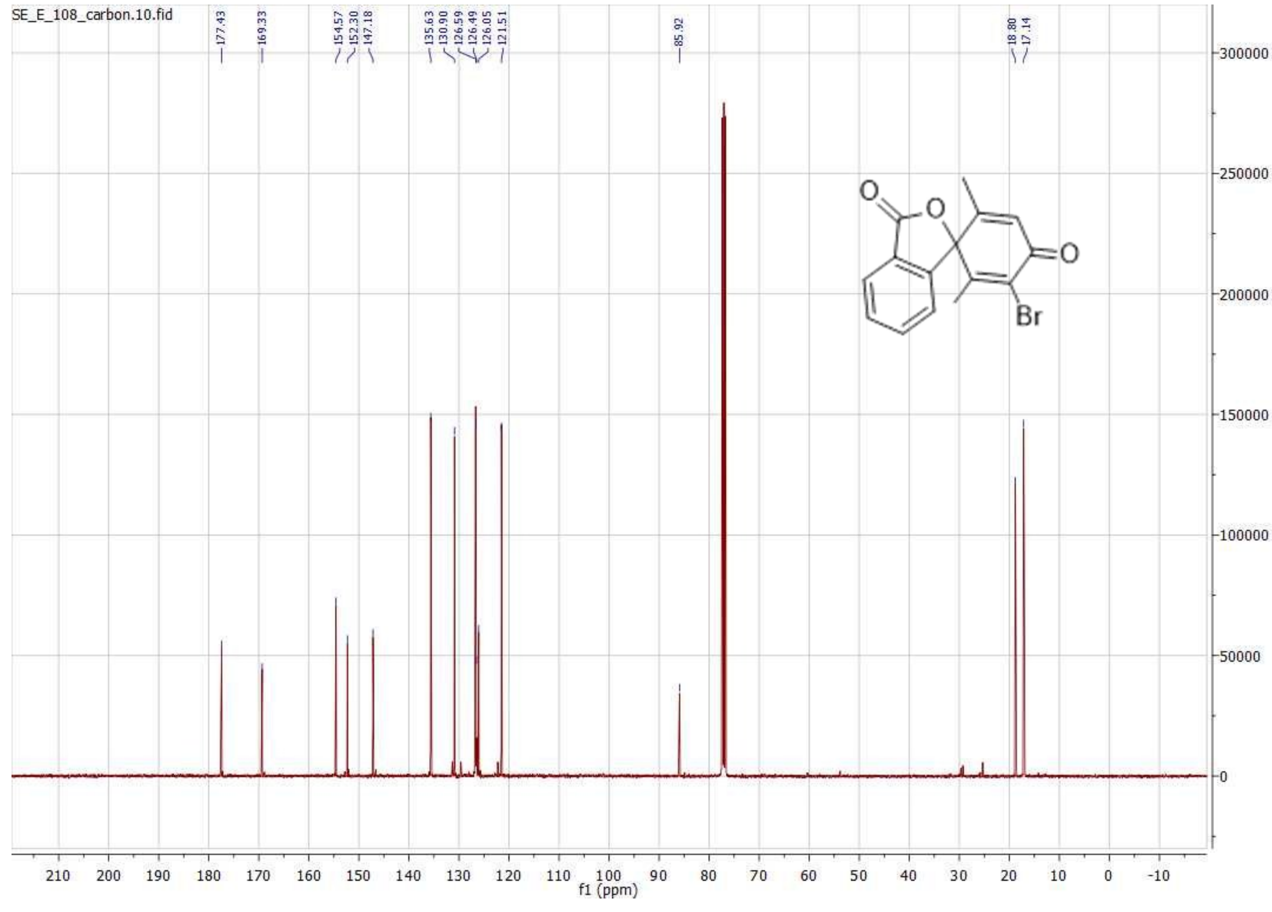
SE_E_108_carbon.10.fid
177.43
169.33
154.57
152.30
147.18
135.63
130.90
126.59
126.49
126.05
121.51
85.92
18.80
17.14
Br
f1 (ppm)

20. References

[1] Huang, Y.; Chan, G. H.; Chiba, S. *Angew. Chem. Int. Ed.* **2017**, *56*, 6544.

[2] Clar, E.; Stewart, D. G. *J. Am. Chem. Soc.*, **1952**, *74* (24), 6235.

[3] Tetsutaro, H.; Takatsugu, S.; Noriyuki, H.; Nobuyuki, K.; Sotaro, M. *Bull. Chem. Soc. Jpn.*, **1993**, *66* (10), 3034.

[4] Pi, JJ.; Lu, XY.; Liu, JH.; Lu, X.; Xiao, B.; Fu, Y.; Guimond, N. *J. Org. Chem.*, **2018**, *83* (10), 5791.

[5] Richtzenhain, H. *Chem. Ber*., **1948**, *81*, 92.

[6] Caille, J; Boukattaya, F.; Boeda, F.; Pearson-Long, M. S. M.; Ammar, H.; Bertus, P. *Org. Biomol. Chem.*, **2018**, *16*, 1519.

[7] Yamato, T; Sakaue, N. *J. Chem. Res. (S)*, **1997**, 440.

[8] Terao, Y.; Wakui, H.; Nomoto, M.; Satoh, T.; Miura, M.; Nomura, M *J. Org. Chem.*, **2003**, *68* (13), 5236.
